# Direct Synthesis of Enamides via Electrophilic Activation of Amides

**DOI:** 10.1021/jacs.1c04363

**Published:** 2021-07-07

**Authors:** Philipp Spieß, Martin Berger, Daniel Kaiser, Nuno Maulide

**Affiliations:** † Institute of Organic Chemistry, University of Vienna, Währinger Straße 38, 1090 Vienna, Austria

## Abstract

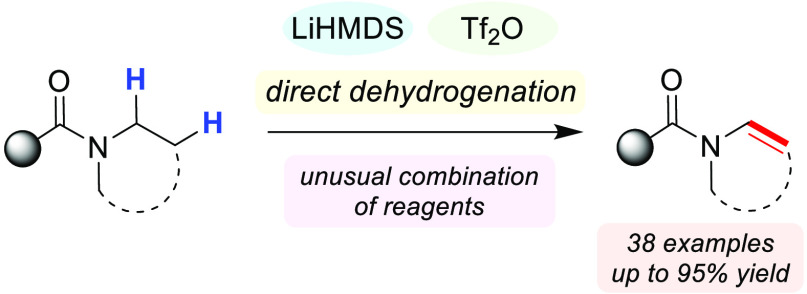

A novel, one-step N-dehydrogenation of amides to enamides is reported. This reaction employs the unlikely combination of LiHMDS and triflic anhydride, which serves as both the electrophilic activator and the oxidant, and is characterized by its simple setup and broad substrate scope. The synthetic utility of the formed enamides was readily demonstrated in a range of downstream transformations.

The chemistry of enamines is a fundamental cornerstone of the organic synthetic toolbox, driven by this compound class’s exceptional nucleophilicity. Nevertheless, the unique reactivity of enamines is accompanied by a high propensity to undergo hydrolysis, leading to considerable difficulties in the handling of these compounds.^1^ Enamides, long regarded as sluggishly reacting surrogates, have recently experienced a renaissance, occupying a niche position at the intersection of desirable resistance to hydrolysis and tunable reactivity. While tempering the nitrogen center with an electron-withdrawing group leads to a reactivity profile more akin to that of classical olefins, enamides are versatile reactants, used in a number of settings, such as transition-metal catalysis, photochemistry, or asymmetric catalysis.^2^ Several approaches for the preparation of enamides have been reported, typically starting from prefunctionalized substrates.^3^ However, the most straightforward approach to access enamides is arguably the direct N-dehydrogenation of the corresponding amides. Whereas routes for the direct desaturation to form enecarbamates have recently become well established,^4^ pathways leading from carboxamides to enamides remain elusive (Scheme 1A). Gevorgyan reported a photoinduced palladium-catalyzed dehydrogenation protocol enabled by hydrogen abstraction and starting from prefunctionalized 2-iodobenzamides,^5^ while Morandi *et al.* recently published a ruthenium-catalyzed variant of this reaction.^6^ Additionally, an electrochemical approach for such an oxidation has been developed. Therein, amides mostly derived from cyclic amines are transformed into hemiaminal methyl ethers that collapse in a second step under acidic conditions.^7^ A further appealing approach is the light-mediated one-step, direct oxidation of *N*-acetyl-pyrrolidine in the presence of titanium dioxide and a copper(II) salt.^8^ However, the single enamide reported was merely detected spectroscopically. It thus appears that the development of novel dehydrogenation methods that do not require prefunctionalization and can accommodate N-cyclic and -acyclic amide substrates is in high demand.

**Scheme 1:**
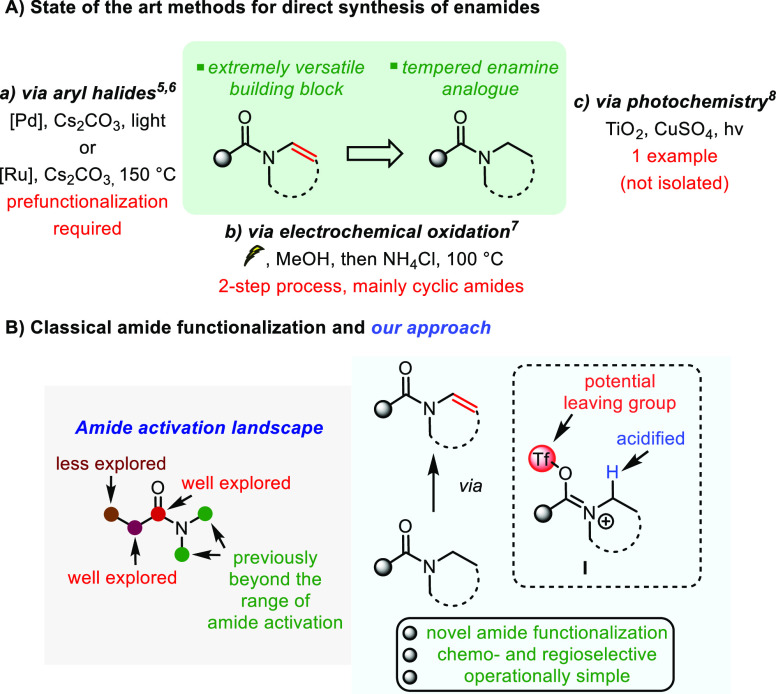
Previous Approaches to the Synthesis of Enamides by N-Dehydrogenation of Amides and Our Proposal

In recent years, electrophilic amide activation has emerged as a powerful tool to overcome the intrinsic low electrophilicity of amides.^9^ In particular, the combination of triflic anhydride and suitable pyridine bases^10^ has enabled a plethora of methods to functionalize the carbonyl portion, as well as the α-position (Scheme 1B).^11^ However, N-functionalization of amides has remained virtually uncharted territory. Our long-standing interest in the field of amide activation prompted us to speculate whether the initially formed iminium triflate **I** (Scheme 1B) might offer a pathway for N-dehydrogenation.

We hypothesized this intermediate to exhibit enhanced acidity of the proton α to nitrogen and became intrigued by the possibility of activating it in the presence of a strong, non-nucleophilic base.^12^ Herein, we report the development of a new protocol that allows access to enamides from amides *via* electrophilic amide activation.

Extensive optimization was necessary to unlock N-dehydrogenation reactivity on model substrate **1a**,^13^ with the unprecedented combination of LiHMDS, triflic anhydride (Tf_2_O) and diethyl ether as the solvent proving optimal and affording product **2a** in 89% isolated yield (Table 1, entry 1). It is noteworthy that KHMDS and NaHMDS performed considerably worse, and other bases showed marginal to no reactivity altogether (entries 2–5). When diethyl ether was replaced by tetrahydrofuran or when the temperature was elevated, a slightly lower conversion was observed (entries 6 and 7). These conditions are all the more surprising as it is well known (and confirmed by our experience) that ethereal solvents are generally incompatible with Tf_2_O, as they undergo swift polymerization at noncryogenic temperatures. To our surprise, the counterintuitive preaddition of LiHMDS played a significant role in the success of this process: a considerable decrease in yield (from 94% to 52%) was observed when Tf_2_O was added first (entry 8).

**Table 1:**
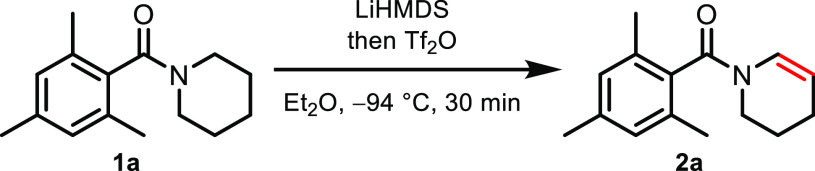
Optimization of the Reaction Conditions^a^

entry	deviation from standard conditions	yield (%)^b^
1	none	94 (89^c^)
2	NaHMDS	15
3	KHMDS	traces
4	2-I-pyr^d^	0
5	LDA	11
6	THF	84
7	–78 °C	87
8	inverse addition^e^	52

a Reaction conditions: **1a** (0.30 mmol), LiHMDS (1.44 mmol, 1 M solution in THF, 4.8 equiv), Tf_2_O (0.72 mmol, 2.4 equiv), Et_2_O (1.5 mL).

b GC yields using decane as internal standard.

c Isolated yield.

d 2-I-pyr (2.2 equiv) followed by the addition of Tf_2_O (1.1 equiv), DCM, 0 °C to rt, 16 h.

e First addition of Tf_2_O, then LiHMDS; average result based on two runs.

With reliable reaction conditions in hand, we proceeded to investigate the scope of this reaction, initially focusing on the nitrogen substituent of the amide (Scheme 2). Good to excellent yields were obtained for enamides of different ring sizes (**2a**–**c**, **2a** gave 80% yield on gram scale). Additionally, heteroatom-substituted (**2d**), bicyclic (**2e**), and also morpholine- and piperazine-derived enamides (**2f**, **2g**), were readily synthesized in good yields. Importantly, acyclic amides, which are scarcely reported in other oxidation protocols (*vide supra*), were also amenable to this method, and *E*-enamides were obtained exclusively (**2h**–**j**).

**Scheme 2:**
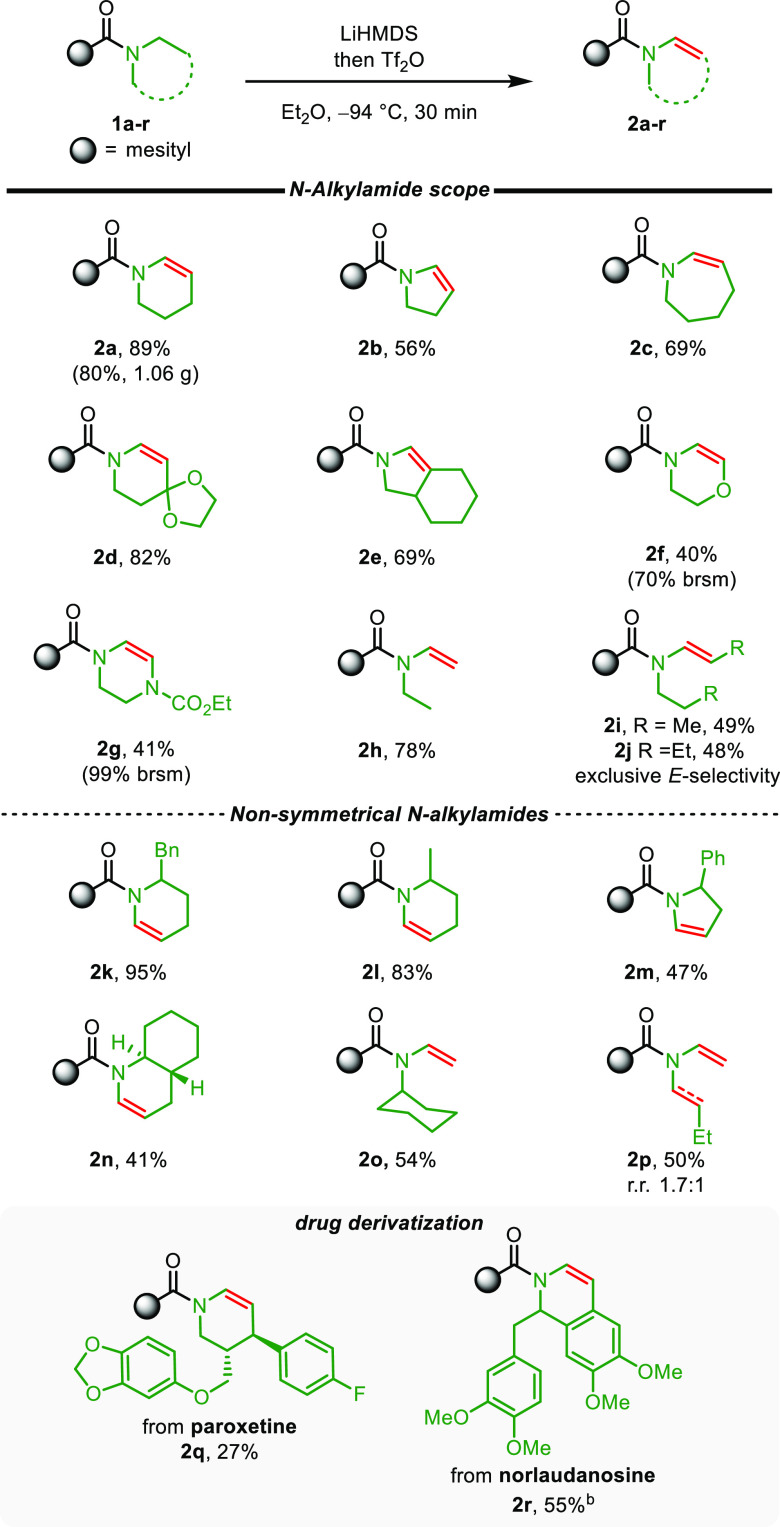
Scope of *N*-Alkylamides. Reaction conditions: amide (0.30 mmol), LiHMDS (1.44 mmol, 1 M solution in THF, 4.8 equiv), then Tf_2_O (0.72 mmol, 2.4 equiv), Et_2_O (1.5 mL). DCM was used as cosolvent.

Next, nonsymmetric amides were analyzed, showing a marked preference for N-dehydrogenation of the least encumbered nitrogen substituent (**2k**–**o**), and even modest selectivity between ethyl and butyl substituents was found (**2p**). On the basis of these auspicious results, we turned our attention to some more complex systems. An amide derivative of the drug paroxetine, bearing one β-substituent, was desaturated regioselectively (albeit in modest yield) to provide a single regioisomeric enamide **2q**, and a derivative of norlaudanosine, a dopamine metabolite, was N-dehydrogenated smoothly (**2r**).

Following the study of different nitrogen substituents, our focus shifted to the investigation of the carbon portion of the carboxamide (Scheme 3). The highly encumbered enamide **4a** and anthracenyl-derived enamide **4b** were obtained in good yields. Importantly, unsubstituted benzamides also delivered the desired enamides, albeit in lower yields (**4c**, **4d**). Again, upscaling allowed a gram-scale synthesis of the enamide **4c**.

**Scheme 3:**
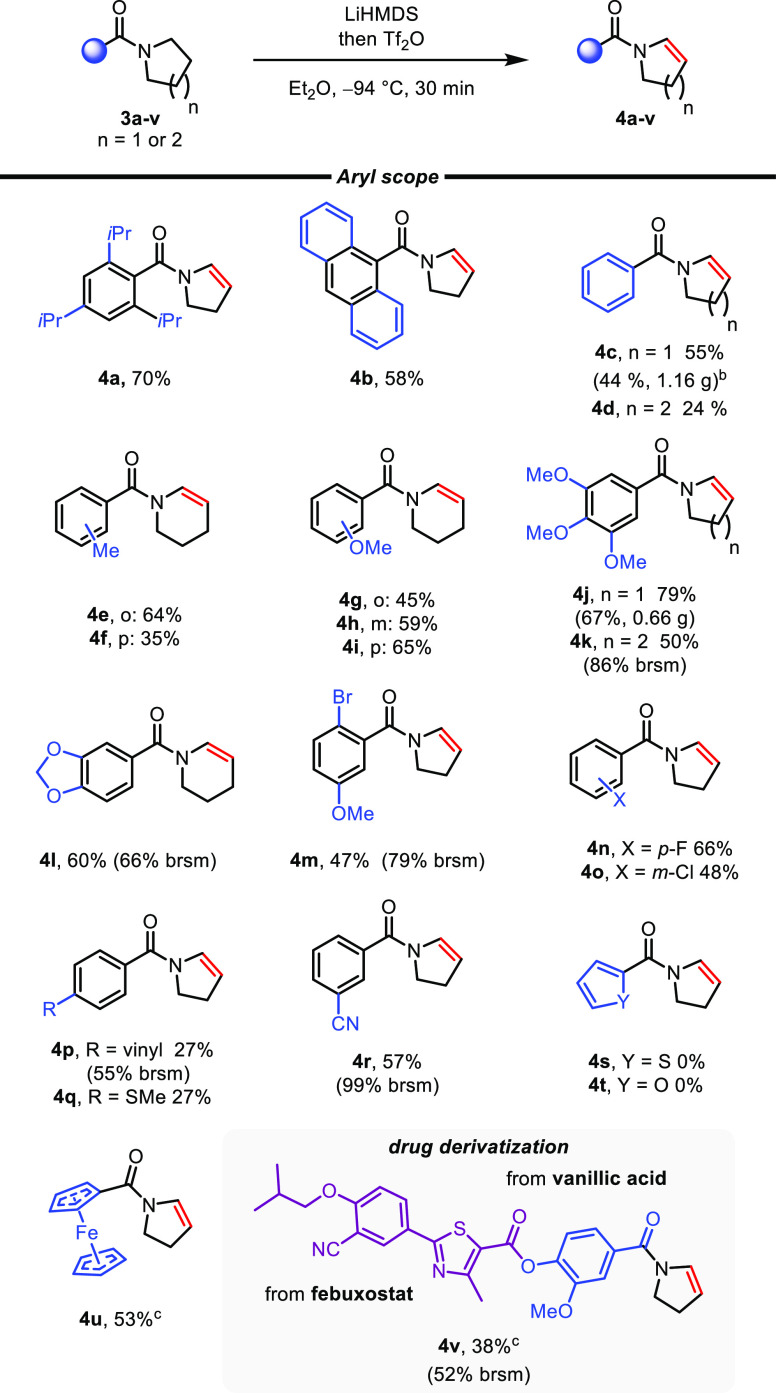
Scope of Benzamide Derivatives Reaction conditions: amide (0.30 mmol), LiHMDS (1.44 mmol, 1 M solution in THF, 4.8 equiv), then Tf_2_O (0.72 mmol, 2.4 equiv), Et_2_O (1.5 mL). LiHMDS (3.6 equiv) and Tf_2_O (1.8 equiv) were used. DCM was used as cosolvent.

Interestingly, while shuffling methyl (**4e**, **4f**) and methoxy (**4g**–**i**) substituents around the aromatic ring, an enhanced reactivity was observed for substrates carrying a methyl group in the *ortho* position, whereas a methoxy group was shown to be advantageous in the *meta* and *para* position.^14^

Other electron-rich aromatics (**4j**–**l**) were also amenable to the reaction, as were various aryl halides (**4m**–**o**). In addition, the process proved to be tolerant of several functional groups, including vinyl (**4p**), thiol (**4q**), and nitrile (**4r**) substituents. Unfortunately, thienoyl- and furoylamides (**3s**, **3t**) failed to react, and no conversion was observed. To our delight, a ferrocene-derived enamide (**4u**) was obtained in good yield, and we were pleased to find a functional-group-heavy conjugate of vanillic acid and febuxostat to provide the desired N-dehydrogenated product **4v**. With the exception of **3u**, all reactions with nonbenzamide substrates were unsuccessful, presumably due to a slower activation with triflic anhydride for α-tertiary amides or the generation of a keteniminium ion in the case of enolizable amides.^13^

To showcase the utility of the products, we performed several further functionalization reactions (Scheme 4). The enamides generated herein could be readily engaged in cycloaddition reactions featuring inverse electron-demand Diels–Alder reactions (**5a**, **5b**), or even a [2+2] cycloaddition with arynes (**5c**).^15−17^ Moreover, ring deconstruction was readily achieved under oxidative fluorinating conditions, allowing access to a decarbonylated fluorinated acyclic amine (**5e**).^18^ In a Fischer indole synthesis-type reaction, the carbon core again was easily deconstructed, allowing the synthesis of a phenyl-melatonin derivative (**5d**).^19^ In addition, under acidic treatment, a Nazarov-type cyclization was observed, forming tricyclic lactam **5f** in good yield.^20^ Finally, β-arylation of the enamide was readily achieved under copper catalysis, affording **5g** in modest yield.^21^ The broad spectrum of reactivity presented by these functionalizations—from cycloadditions to ring deconstructions to cyclizations—highlights the versatility of enamides as building blocks.

**Scheme 4:**
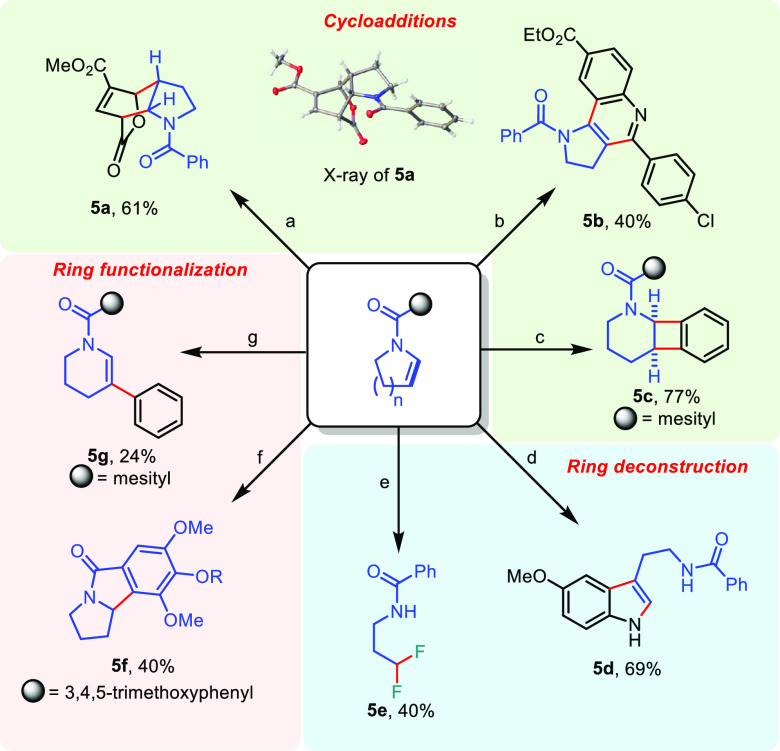
Application of Enamides **4c** (1.0 equiv), methyl coumalate (1.1 equiv), toluene, 130 °C, 15 h. **4c** (1.0 equiv), ethyl 4-aminobenzoate (1.0 equiv), *p*-chlorobenzaldehyde (1.0 equiv), Sc(OTf)_3_ (0.2 equiv), MeCN, rt, 3 d, then DDQ (2.0 equiv), CHCl_3_, rt, 16 h. **2a** (1.0 equiv), 2-(trimethylsilyl)phenyl trifluoromethanesulfonate (3.0 equiv), CsF (4.0 equiv), 1,4-dioxane, 110 °C, 16 h. **4c** (1.0 equiv), (4-methoxyphenyl)hydrazine hydrochloride (1.2 equiv), AcOH/EtOH/H_2_O, 110 °C, 1 h. **4c** (1.0 equiv), Selectfluor (4.0 equiv), AgBF_4_ (2.5 equiv), acetone/H_2_O, rt, 16 h. **4j** (1.0 equiv), CF_3_SO_3_H/CHCl_3_ (1:1), rt, 16 h. R = H/OMe (9:1). **2a** (1.0 equiv), diphenyliodonium triflate (2.0 equiv), copper(II) triflate (0.2 equiv), DCM, 80 °C, 24 h.

Mechanistic studies shed additional light on this unusual transformation (Scheme 5). Use of an ^18^O-enriched amide (**6a**) revealed conservation of the isotopic label in the obtained product (**6b**). This is a very unusual trait in electrophilic amide activation, where the carboxamide oxygen is otherwise almost always lost.^22^ Additional labeling experiments employing deuterated substrates (**6c**, **6f**) revealed kinetic isotope effects (KIE) of 4.8 for a cyclic (**6d**:**6e**) and 34.2 for an acyclic (**6g**:**6h**) substrate. Both results strongly suggest that abstraction of the proton α to nitrogen is involved in the rate-determining step of the reaction. Moreover, the latter result, which shows an unusually high primary isotope effect, leads to the conclusion that quantum tunneling might come into play. As tunneling effects are well known to gain influence at lower temperatures, and in particular when using highly sterically hindered bases,^23^ we tested the same substrate (**6f**) at a higher temperature (−41 °C), which revealed a diminished KIE of 13.2. Additionally, in the ^19^F NMR analysis of the crude reaction mixture, the presence of triflinate (**7**) was observed and analysis of the crude reaction mixture by HRMS even revealed the presence of a mixed S(IV)/S(VI) species (**8**) (see the Supporting Information for a proposed mechanism for the formation of **8**). Importantly, a deuteration experiment indicated no direct abstraction of the *N*-α-hydrogen by the base, precluding an alternative deprotonation/triflation mechanism.^13^ On the basis of these findings, we postulate a mechanism in which amide activation to an iminium triflate by Tf_2_O decisively acidifies the *N*-α-hydrogen, after which a deprotonation/elimination step takes place, leading to extrusion of a triflinate anion (**7**), thus accounting for the ^18^O label retention. A subsequent elimination leads to the observed enamide products.

**Scheme 5:**
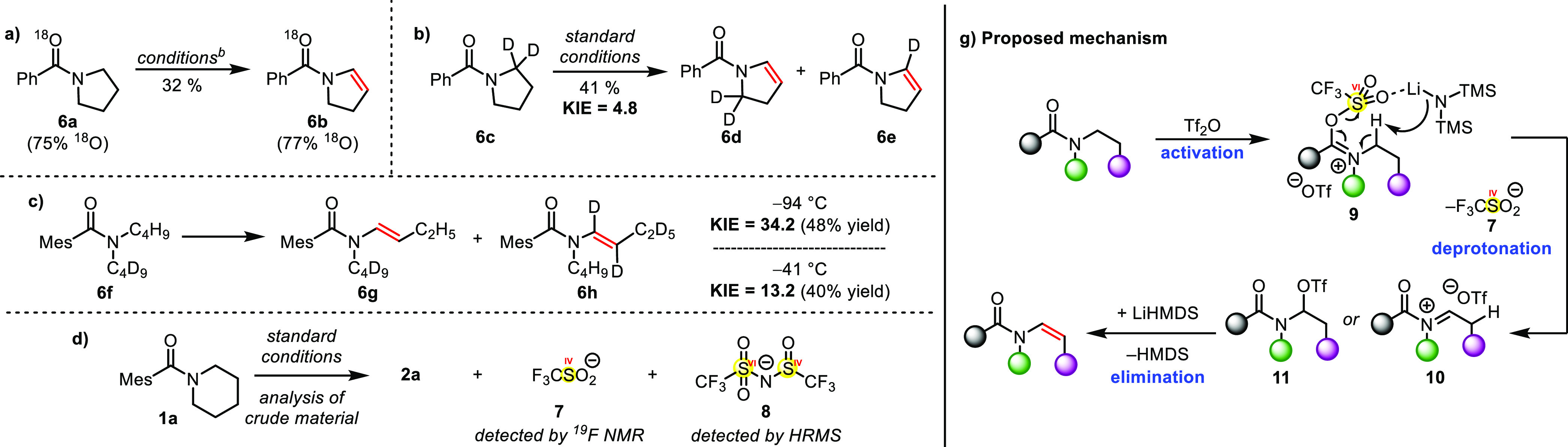
Mechanistic Studies and Our Proposal For exact reaction conditions see Table 1, entry 1. Conditions: −78 °C, THF.

In conclusion, we have described a new method to access enamides *via* an oxidation event mediated by electrophilic amide activation under unusual conditions. To the best of our knowledge, this is the first general one-step approach for the synthesis of N*-*cyclic and -acyclic enamides that does not require prefunctionalization of the substrates. Applications include modification of drug derivatives, cycloadditions, as well as ring deconstructions and emphasize the privileged position of enamides as unique building blocks. Most importantly, the unlocking of N*-*functionalization through electrophilic amide activation promises to open yet further perspectives in this chemistry.

## Supporting Information Available

Experimental procedures; spectroscopic and X-ray data (PDF)

## Accession Codes

CCDC 2075992 contains the supplementary crystallographic data for this paper. These data can be obtained free of charge via www.ccdc.cam.ac.uk/data_request/cif, or by emailing data_request@ccdc.cam.ac.uk, or by contacting The Cambridge Crystallographic Data Centre, 12 Union Road, Cambridge CB2 1EZ, UK; fax: +44 1223 336033.
