# Supplementary material for: Direct Synthesis of Enamides via Electrophilic Activation of Amides
Source: J Am Chem Soc. 2021 Jul 7;143(28):10524–9. doi: 10.1021/jacs.1c04363 (PMC8299460; doi:10.1021/jacs.1c04363)
Supplement: Supplementary file 1 — ja1c04363_si_001.pdf [file ja1c04363_si_001.pdf]

Direct Synthesis of Enamides *via* Electrophilic Activation of Amides

Philipp Spieß,[a] Martin Berger,[a] Daniel Kaiser[a] and Nuno Maulide[a]*

[a] *Institute of Organic Chemistry, University of Vienna, Währinger Straße 38, 1090 Vienna, Austria*

E-Mail: *nuno.maulide@univie.ac.at*, *Homepage:* *http://maulide.univie.ac.at*

Table of Contents

1. General Information 2

2. Optimization of the Reaction Conditions 2

3. Experimental 6

3.1. General Procedures 6
3.1.1. General Procedure 1A (GP1A): Synthesis of Amides from Acyl Chlorides 6
3.1.2. General Procedure 1B (GP1B): Synthesis of Amides from Acyl Chlorides 6
3.1.3. General Procedure 2A (GP2A): Synthesis of Amides from Carboxylic Acids 6
3.1.4. General Procedure 2B (GP2B): Synthesis of Amides from Carboxylic Acids 7
3.1.5. General Procedure 3 (GP3): Synthesis of Enamides 7

3.2. Characterization of Starting Materials 8

3.3. Characterization of Enamides 17

3.4. Unsuccessful and low-yielding Substrates 29

3.5. Application of Enamides 30

4. Mechanistic Studies 34

4.1. Isotopic-labeling Experiments: 34
4.1.1. Preparation of Isotopically-labeled Starting Materials 34
4.1.2. *N*-Dehydrogenation Reaction with Isotopically-labeled Substrates 37

4.2. Analysis of the Crude Material 41

4.3. Control Experiment 43

5. References 44

6. NMR Spectra 45

7. X-Ray Crystallographic Data 119

1. General Information

Unless otherwise stated, all glassware was flame-dried before use and all reactions were performed under an atmosphere of argon. All solvents were distilled from appropriate drying agents prior to use or directly taken from commercial sealed bottles under an atmosphere of argon. All reagents were used as received from commercial suppliers unless otherwise stated. Trifluoromethanesulfonic anhydride (Tf_2O) was distilled over P_4O_{10} prior to use and stored under inert atmosphere in the fridge for a maximum of roughly 3 weeks.[1] Reaction progress was monitored by thin layer chromatography (TLC) performed on aluminum plates coated with silica gel F254 with 0.2 mm thickness. Chromatograms were visualized by fluorescence quenching with UV light at 254 nm or by staining using potassium permanganate. Flash column chromatography was performed using silica gel 60 (230-400 mesh, Merck and co.). Neat infrared spectra were recorded using a Perkin-Elmer Spectrum 100 FT-IR spectrometer. Wavenumbers (ν_{max}) are reported in cm^{-1}. Mass spectra were obtained using a Finnigan MAT 8200 or (70 eV) or an Agilent 5973 (70 eV) spectrometer, using electrospray ionization (ESI). All ^1H NMR and ^{13}C NMR spectra were recorded using a Bruker AV-400, AV-600 spectrometer or AV-700 spectrometer at 300K. Chemical shifts are given in parts per million (ppm, δ), referenced to the solvent peak of $CDCl_3$, defined at δ = 7.26 ppm (^1H NMR) and δ = 77.16 (^{13}C NMR). Coupling constants are quoted in Hz (*J*). ^1H NMR splitting patterns are designated as singlet (s), doublet (d), triplet (t), quartet (q) and quintet (quint) as they appeared in the spectrum. If the appearance of a signal differs from the expected splitting pattern, the observed pattern is designated as apparent (app). Splitting patterns that could not be interpreted or easily visualized are designated as multiplet (m) or broad (br).

2. Optimization of the Reaction Conditions

General procedure: A flame-dried Schlenk flask was loaded with amide (**1a**, 69.4 mg, 0.30 mmol, 1.00 eq.) and dissolved in the corresponding solvent. The solution was set to the desired temperature (temperature was kept until quenching) and the base was added slowly (20 s). After the addition, the resulting mixture was stirred for 10 min. Following that, Tf_2O was added dropwise over 1 min under vigorous stirring. The reaction was stirred for 30 min before remaining reagents were quenched by the addition of a saturated solution of NH_4Cl (5 mL). The yield was determined by GC FID analysis using 1-decane (58.5 µL, 42.7 mg, 0.30 mmol) as internal standard.

Table S1. Optimization of the reaction temperature.[a]

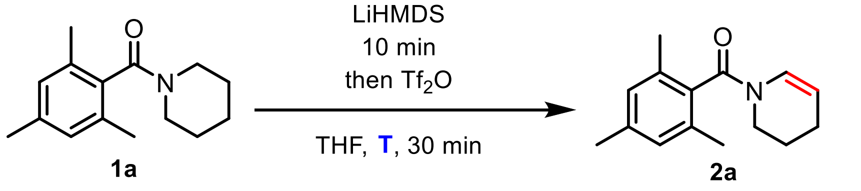

Entry	**T (°C)**	Yield (%[b])
**1**	**-94**	**90**
2	-78	67
3	-40	46
4	0	32
5	25	34

[a] **1a** (0.30 mmol), THF (1.5 mL), LiHMDS (0.96 mmol, 1 M solution in THF) with slow addition, 10 min, then Tf_2O (0.48 mmol) with addition over 1 min. [b] GC yield.

Table S2. Optimization of the base.[a]

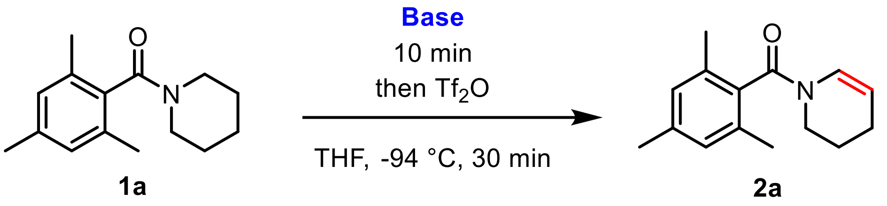

Entry	Base	Yield (%[b])
**1**	**LiHMDS[c]**	**90**
2	NaHMDS[c]	33
3	KHMDS[c]	6
4	LiO*t*Bu[c]	6
5	*t*BuLi[d]	0
6	NaH[e]	traces

[a] **1a** (0.30 mmol), THF (1.5 mL), Base with slow addition, 10 min, then Tf_2O (0.48 mmol) with addition over 1 min. [b] GC yield. [c] bases were used as 1 M solutions in THF. [d] 1.7 M in pentane. [e] was added neat.

Table S4. Optimization of the used equivalents of reagents.[a]

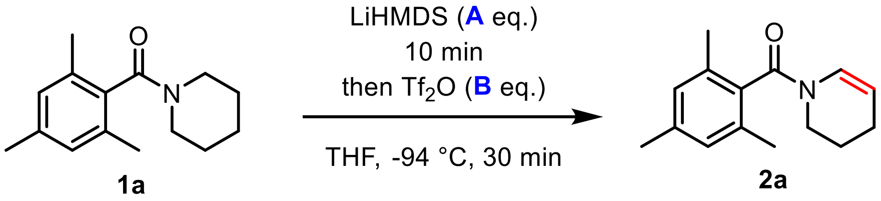

Entry	A, B (eq.)	Yield (%[b])
1	7.2, 3.6	88
**2**	**4.8, 2.4**	**90**
3	3.6, 1.8	72
4	2.4, 1.2	71
5	2.4, 2.4	67
6	7.2, 2.4	82

[a] **1a** (0.30 mmol), THF (1.5 mL), LiHMDS (1 M solution in THF) with slow addition, 10 min , then Tf_2O with addition over 1 min. [b] GC yield.

Table S5. Optimization of the addition order.[a]

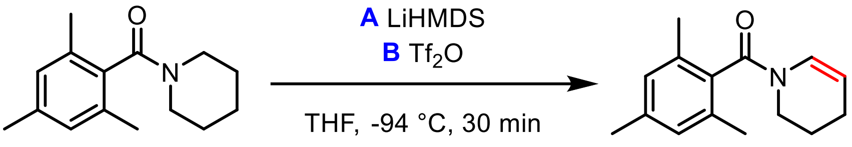

Entry	1st addition (time)	reaction time after 1st addtion	2nd addition (time)	Yield (%[b])
1	B (1 min)	3 min	A (1 min)	50
2	A (1 min)	3 min	B (1 min)	88
3	A (1min)	-	B (1 min)	64
**4**	A (1min)	10 min	B (1 min)	**90**
5	A (1min)	3 min	B (all at once)	68
6	A (1min)	3 min	B (3 min)	80
7	A (1min)	3 min	B (1 min)[c]	83
8	premixed A + B[d]	3 min	**1a** (1min)	22[e]

[a] **1a** (0.30 mmol), LiHMDS (0.96 mmol, 1 M solution in THF), 10 min, Tf_2O (0.48 mmol), 1.5 mL THF. [b] GC yield. [c] equivalents of A have been halved, addition of 2nd portion of A 10 min after the addition of B. [d] B was added to A over 1 min, 10 min, slow addition of **1a**. [e] isolated yield, major amounts of decomposition were observed.

Table S6. Optimization of the concentration.[a]

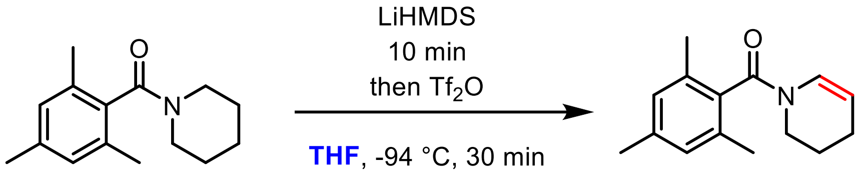

1a 2a

Entry	Total solvent volume in mL (Molarity)[b]	Yield (%[c])
1	1.5 (0.2 M)	40
2	2.25 (0.13 M)	83
**3**	**3 (0.1 M)**	**90**
4	3.75 (0.08 M)	79
5	6 (0.05 M)	22

[a] **1a** (0.30 mmol), LiHMDS (0.96 mmol, 1 M solution in THF) with slow addition, 10 min, Tf_2O (0.48 mmol) with addition over 1 min. [b] total solvent volume including 1.5 mL of the LiHMDS solution. [c] GC yield.

Table S7. Optimization of the solvent.[a]

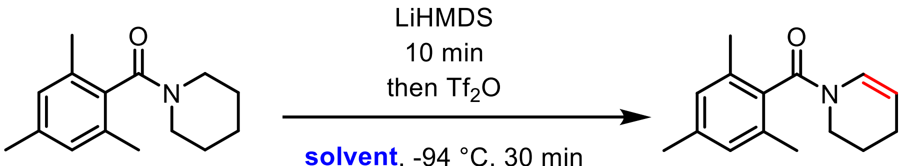

1a 2a

Entry	Solvent (mL)[b]	Yield (%)[c]
1	THF	90
2	Et_2O	70
3	2-MeTHF	36
4	2-MeTHF[d]	50
5	toluene	27
6	DCM	53
**7**	**Et_2O + THF**	**94 (89[e])**
8	2-MeTHF + THF	92
9	toluene + THF	85

[a] **1a** (0.30 mmol), solvent (1.5 mL), LiHMDS (0.96 mmol, 1 M solution in the corresponding solvent) with slow addition, 10 min, then Tf_2O (0.48 mmol) with addition over 1 min. [b] total solvent volume 3 mL, 2nd named solvent from the LiHMDS solution. [c] GC yield. [d] -116 °C instead of -94 °C. [e] isolated yield.

3. Experimental

3.1. General Procedures

3.1.1. General Procedure 1A (GP1A): Synthesis of Amides from Acyl Chlorides
(**1a-o** except for **1c, 3d-l**, **3n-o**)

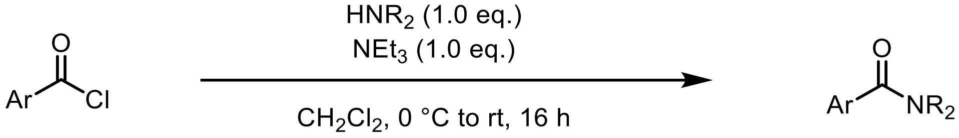

All reactions were run on a 5 mmol scale if not indicated otherwise.

The corresponding acyl chloride (1.0 eq.) was dissolved in CH_2Cl_2 (0.6 M) and the resulting mixture was cooled to 0 °C. Then, the amine (1.0 eq.) was added dropwise followed by the slow addition of triethylamine (1.0 eq.). After completion of addition, the ice bath was removed and the reaction was stirred for 16 h. The solution was transferred into a separatory funnel and sequentially washed with H_2O, 1 M NaOH, 1 M HCl and brine. The organic phase was dried over anhydrous $MgSO_4$ and the filtrate was concentrated under reduced pressure giving rise to the desired amide. Depending on the purity of the obtained material as analysed by ^{1}H NMR, additional flash column chromatography was performed (SiO_2, heptane/EtOAc).

3.1.2. General Procedure 1B (GP1B): Synthesis of Amides from Acyl Chlorides
(**1c**, **3c**)

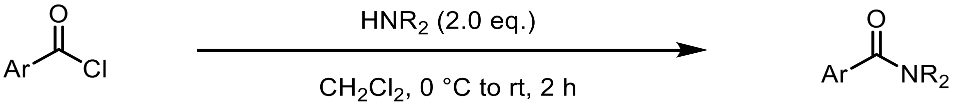

All reactions were run on a 2 mmol scale if not indicated otherwise.

The corresponding acyl chloride (1.0 eq.) was dissolved in CH_2Cl_2 (0.1 M) and the resulting mixture was cooled to 0 °C. Then, the amine (2.0 eq.) was added dropwise. After completion of addition, the ice bath was removed and the reaction was stirred for 2 h. The solution was transferred into a separatory funnel and sequentially washed with 1 M HCl and brine. The organic phase was dried over anhydrous $MgSO_4$ and the filtrate was concentrated under reduced pressure and the resulting crude material was purified via flash column chromatography (SiO_2, heptane/EtOAc).

3.1.3. General Procedure 2A (GP2A): Synthesis of Amides from Carboxylic Acids
(**3m**, **3p**, **3q**, **3r**)

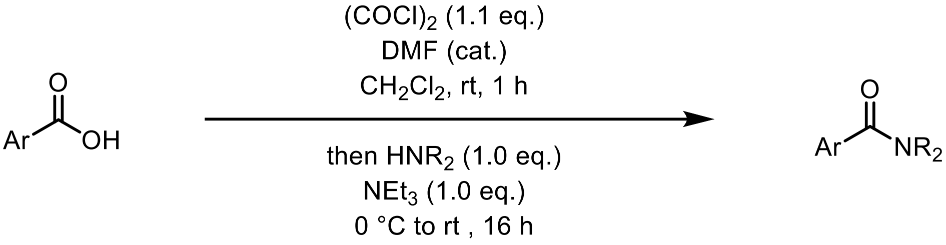

All reactions were run on a 5 mmol scale if not indicated otherwise.

The corresponding carboxylic acid (1.0 eq.) was dissolved in CH_2Cl_2 (0.6 M) and a few drops of dimethylformamide were added. Oxalyl chloride (1.1 eq.) was added dropwise whereby a strong gas evolution was observed. After 1 h, the amine (1.0 eq.) was slowly added, followed by the addition of

triethylamine (1.0 eq.). The reaction was stirred for 16 h. The solution was transferred into a separatory funnel and sequentially washed with H_2O, 1 M NaOH, 1 M HCl and finally brine. The organic phase was dried over anhydrous $MgSO_4$ and the filtrate was concentrated under reduced pressure giving rise to the desired amide. Depending on the purity of the obtained material as analysed by ^1H NMR, additional flash column chromatography was performed (SiO_2, heptane/EtOAc).

3.1.4. General Procedure 2B (GP2B): Synthesis of Amides from Carboxylic Acids (**3a**, **3b, 3u**)

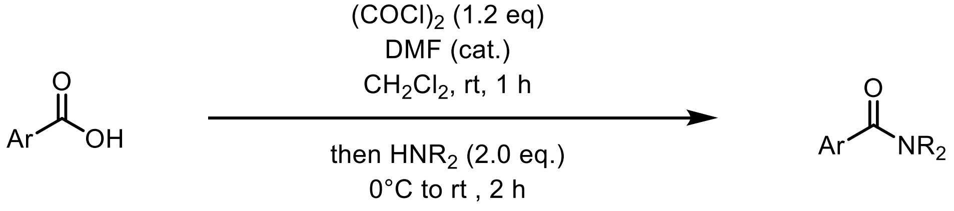

The corresponding carboxylic acid (1.0 eq.) was dissolved in CH_2Cl_2 (0.2 M) and a few drops of DMF were added. Oxalyl chloride (1.2 eq.) was added dropwise whereby a strong gas evolution was observed. After 1 h, the amine (2.0 eq.) was slowly added. The reaction was stirred for 2 h. The solution was transferred into a separatory funnel and sequentially washed with 1 M HCl and brine. The organic phase was dried over anhydrous $MgSO_4$ and the filtrate was concentrated under reduced pressure giving rise to the desired amide. The crude material was additionally purified via flash column chromatography (SiO_2, heptanes/EtOAc).

3.1.5. General Procedure 3 (GP3): Synthesis of Enamides

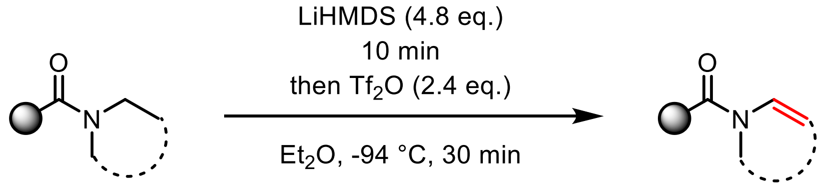

A flame-dried Schlenk flask was loaded with the corresponding amide (0.30 mmol, 1.00 eq.) which was dissolved in anhydrous Et_2O (1.5 mL). The solution was cooled down to -94 °C (dry ice/acetone mixture). Then LiHMDS (1 M in THF, 1.44 mL, 1.44 mmol, 4.80 eq.) was added slowly (ca. 20 s). After the addition, the resulting mixture was stirred for 10 min. Following Tf_2O (121 µL, 0.72 mmol, 2.40 eq.) was added dropwise over 1 min with vigorous stirring (very important). The reaction was stirred for 30 min before remaining reagents were quenched by the addition of a saturated solution of NH_4Cl (10 mL). The solution was transferred into a separatory funnel and extracted with CH_2Cl_2 (3 x 10 mL). The combined organic phases were dried over anhydrous $MgSO_4$ and the solvent was removed under reduced pressure. The crude material was purified by column chromatography (SiO_2, heptanes/EtOAc). Importantly, a noticeable instability of enamides on silica gel has been observed. Therefore, the silica gel was treated with NEt_3/Et_2O (1:9), followed by drying in the air prior to use in the flash column chromatography.

Large scale:
For a large scale (5.5 or 15.2 mmol) the addition time of Tf_2O was extended (0.28 or 0.76 mmol/min) and also the time before the addition of Tf_2O was prolonged (20 min, both cases) to ensure a satisfying cooling.

3.2. Characterization of Starting Materials

Mesityl(piperidin-1-yl)methanone (1a)

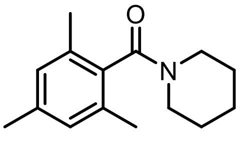

Following the GP1 using 2,4,6-trimethylbenzoyl chloride and piperidine afforded the desired amide (1.15 g, 99%) as yellowish oil. **^{1}H NMR (400 MHz, $CDCl_3$):** δ 6.83 (s, 2H), 3.76 (app s, 2H), 3.16 – 3.11 (m, 2H), 2.26 (s, 3H), 2.21 (s, 6H), 1.67 – 1.64 (m, 4H), 1.46 (app s, 2H) ppm. Spectral data was found in agreement with the literature.[2]

Mesityl(pyrrolidin-1-yl)methanone (1b)

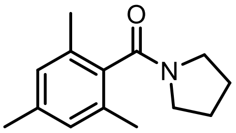

Following the GP1A using 2,4,6-trimethylbenzoyl chloride and pyrrolidine afforded the desired amide (1.06 g, 98%) as yellowish oil. **^{1}H NMR (400 MHz, $CDCl_3$):** 6.83 (s, 2H), 3.67 (t, *J* = 6.9 Hz, 2H), 3.03 (t, *J* = 6.7 Hz, 2H), 2.26 (s, 3H), 2.21 (s, 6H), 2.00 – 1.91 (m, 2H), 1.89 – 1.81 (m, 2H) ppm. Spectral data was found in agreement with the literature.[3]

Azepan-1-yl(mesityl)methanone (1c)

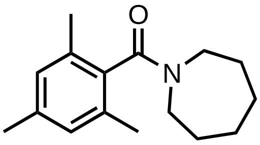

Following the GP1B using 2,4,6-trimethylbenzoyl chloride and pyrrolidine afforded the desired amide (476 mg, 97%) as yellowish oil. **^{1}H NMR (600 MHz, $CDCl_3$)**: δ 6.83 (s, 2H), 3.74 – 3.68 (m, 2H), 3.20 – 3.15 (m, 2H), 2.26 (s, 3H), 2.22 (s, 6H), 1.87 – 1.78 (m, 2H), 1.68 – 1.61 (m, 2H), 1.60 – 1.54 (m, 4H) ppm. **^{13}C NMR (101 MHz, $CDCl_3$)**: δ 171.4, 137.8, 134.5, 133.4, 128.4, 48.6, 44.7, 29.0, 28.4, 28.1, 26.9, 21.2, 19.2 ppm. **IR (neat) ν_{max}:** 2923, 2856, 1628, 1438, 1416, 1377, 1305, 1278, 852, 764, 750. **HRMS (ESI$^+$):** exact mass calculated for $[M+Na]^+$ ($C_{16}H_{23}NNaO^+$) requires m/z 268.1672, found m/z 268.1673.

Mesityl(1,4-dioxa-8-azaspiro[4.5]decan-8-yl)methanone (1d)

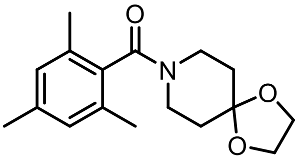

Following the GP1A using 2,4,6-trimethylbenzoyl chloride and 1,4-dioxa-8-azaspiro[4.5]decane afforded the desired amide (1.3 g, 90%) as white solid. **^{1}H NMR (400 MHz, $CDCl_3$):** δ 6.83 (s, 2H), 4.03 – 3.87 (m, 6H), 3.30 – 3.25 (m, 2H), 2.26 (s, 3H), 2.20 (s, 6H), 1.81 – 1.76 (m, 2H), 1.61 – 1.58 (m, 2H) ppm. **^{13}C NMR (101 MHz, $CDCl_3$):** δ 169.9, 138.1, 133.7, 133.5, 128.4, 107.1, 64.6, 44.2, 39.1, 35.9, 35.0, 21.2, 19.1 ppm. **IR (neat) ν_{max}:** 2954, 2918, 2882, 2361, 2342, 1623, 1611, 1466, 1435, 1356, 1245, 1183, 1146, 1114, 1077, 1029, 944, 913, 863. **HRMS (ESI$^+$):** exact mass calculated for $[M+H]^+$ ($C_{17}H_{24}NO_3^+$) requires m/z 290.1751 found m/z 290.1738.

Mesityl(octahydro-2H-isoindol-2-yl)methanone (1e)

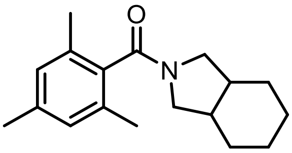

Following the GP1A using 2,4,6-trimethylbenzoyl chloride and octahydro-1H-isoindole afforded the desired amide (1.19 g, 88%) as colorless oil. **^{1}H NMR (400 MHz, $CDCl_3$):** δ 6.84 (s, 2H), 3.63 – 3.56 (m, 2H), 3.09 – 3.03 (m, 1H), 2.91 – 2.85 (m, 1H), 2.33 – 2.16 (m, 11H), 1.69 – 1.32 (m, 8H) ppm. **^{13}C NMR (101 MHz, $CDCl_3$):** δ 170.9, 138.0, 135.4, 133.2, 133.0, 129.0, 128.4, 51.5, 49.0, 37.5, 36.5, 26.1, 25.8, 22.9, 22.7, 21.3, 19.2, 19.0 ppm. **IR (neat) ν_{max}:** 2923, 2874, 2855, 1624, 1439, 1409, 1378, 851, 732. **HRMS (ESI$^+$):** exact mass calculated for $[M+H]^+$ ($C_{18}H_{26}NO^+$) requires 272.2009 m/z found 272.1996 m/z.

Mesityl(morpholino)methanone (1f)

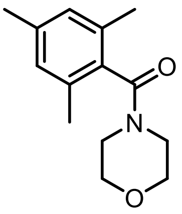

Following the GP1B using 2,4,6-trimethylbenzoyl chloride and morpholine afforded the desired amide (412 mg, 35%) as colorless oil. **^{1}H NMR (400 MHz, CDCl$_3$):** δ 6.85 (s, 2H), 3.86 – 3.81 (m, 2H), 3.79 – 3.72 (m, 2H), 3.59 – 3.54 (m, 2H), 3.21 – 3.15 (m, 2H), 2.27 (s, 3H), 2.22 (s, 6H) ppm. **^{13}C NMR (101 MHz, CDCl$_3$):** δ 170.1, 138.4, 133.7, 132.9, 128.5, 67.2, 67.1, 46.6, 41.6, 21.2, 19.2 ppm. **IR (neat) ν_{max}:** 2916, 2854, 1634, 1456, 1436, 1277, 1255, 1178, 1114, 1010, 841, 751. **HRMS (ESI$^+$):** exact mass calculated for $[M+Na]^+$ ($C_{14}H_{19}NNaO_2^+$) requires m/z 256.1308, found m/z 256.1308.

Ethyl 4-(2,4,6-trimethylbenzoyl)piperazine-1-carboxylate (1g)

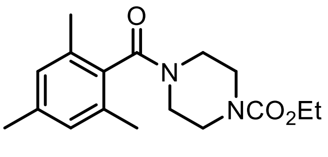

Following the GP1A using 2,4,6-trimethylbenzoyl chloride and ethyl piperazine-1-carboxylate afforded the desired amide (1.47 g, 97%) as white solid. **^{1}H NMR (400 MHz, CDCl$_3$):** δ 6.85 (s, 2H), 4.15 (q, *J* = 7.0 Hz, 2H), 3.81 (t, *J* = 5.1 Hz, 2H), 3.58 – 3.53 (m, 2H), 3.37 (t, *J* = 4.8 Hz, 2H), 3.17 (t, *J* = 4.4 Hz, 2H), 2.27 (s, 3H), 2.20 (s, 6H), 1.26 (t, *J* = 6.5 Hz, 3H) ppm.**^{13}C NMR (101 MHz, CDCl$_3$):** δ 170.2, 155.5, 138.5, 133.6, 132.9, 128.5, 61.9, 45.9, 41.0, 21.2, 19.1, 14.7 ppm. **IR (neat) ν_{max}:** 2984, 2917, 2853, 1697, 1638, 1422, 1382, 1227, 1178, 1114, 1004, 990, 852, 766. **HRMS (ESI$^+$):** exact mass calculated for $[M+H]^+$ ($C_{17}H_{25}N_2O_3^+$) requires 305.1860 m/z found 305.1844 m/z.

N,N-Diethyl-2,4,6-trimethylbenzamide (1h)

Following the GP1A using 2,4,6-trimethylbenzoyl chloride and diethylamine afforded the desired

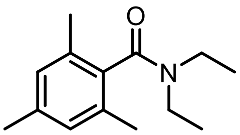

amide (1.06 g, 97%) as white solid. **^{1}H NMR (400 MHz, CDCl$_3$):** δ 6.83 (s, 2H), 3.60 (q, *J* = 6.9 Hz, 2H), 3.11 (q, *J* = 7.0 Hz, 2H), 2.27 (s, 3H), 2.20 (s, 6H), 1.26 (t, *J* = 7.0 Hz, 3H), 1.02 (t, *J* = 7.0 Hz, 3H) ppm. Spectral data was found in agreement with the literature.[4]

2,4,6-Trimethyl-N,N-dipropylbenzamide (1i)

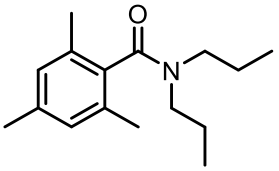

Following the GP1A using 2,4,6-trimethylbenzoyl chloride and dipropylamine afforded the desired amide (1.17 g, 95%) as white solid. **^{1}H NMR (400 MHz, CDCl$_3$):** δ 6.82 (s, 2H), 3.52 – 3.44 (m, 2H), 3.05 – 2.93 (m, 2H), 2.20 (s, 6H), 1.78 – 1.64 (m, 2H), 1.49 – 1.37 (m, 2H), 0.98 (t, *J* = 7.4 Hz, 3H), 0.73 (t, *J* = 7.4 Hz, 3H) ppm. **^{13}C NMR (101 MHz, CDCl$_3$):** δ 171.2, 137.7, 134.3, 133.5, 128.4, 50.0, 46.1, 21.7, 21.2, 20.8, 19.2, 11.8, 11.5 ppm. **IR (neat) ν_{max}:** 2969, 2932, 2874, 1618, 1464, 1416, 1378, 1309, 1255, 1091, 896, 855, 755, 595. **HRMS (ESI$^+$):** exact mass calculated for $[M+Na]^+$ ($C_{16}H_{25}NNaO^+$) requires m/z 270.1828 found m/z 270.1830.

N,N-Dibutyl-2,4,6-trimethylbenzamide (1j)

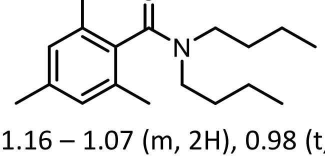

Following the GP1A using 2,4,6-trimethylbenzoyl chloride and dibutylamine afforded the desired amide (1.12 g, 82%) as colorless liquid. **^{1}H NMR (400 MHz, CDCl$_3$):** δ 6.81 (s, 2H), 3.53 – 3.48 (m, 2H), 3.03 – 2.99 (m, 2H), 2.26 (s, 3H), 2.19 (s, 6H), 1.71 – 1.59 (m, 2H), 1.44 – 1.34 (m, 4H), 1.16 – 1.07 (m, 2H), 0.98 (t, *J* = 7.3 Hz, 3H), 0.77 (t, *J* = 7.3 Hz, 3H) ppm. **^{13}C NMR (101 MHz, CDCl$_3$)**: δ 171.1, 137.7, 134.3, 133.5, 128.3, 48.0, 44.1, 30.7, 29.6, 21.2, 20.7, 20.1, 19.2, 14.1, 13.8 ppm. **IR (neat) ν_{max}:** 2957, 2930, 2871, 1628, 1612, 1457, 1417, 1377, 1295, 1259, 1234, 1090, 850, 751, 596. **HRMS (ESI$^+$):** exact mass calculated for $[M+H]^+$ ($C_{18}H_{30}NO^+$) requires m/z 276.2327 found m/z 276.2331.

(2-Benzylpiperidin-1-yl)(mesityl)methanone (1k)

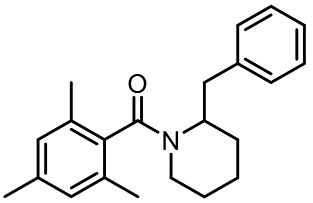

Following the GP1A (1.37 mmol scale) using 2,4,6-trimethylbenzoyl chloride and 2-benzylpiperidine afforded the desired amide (277 mg, 63%) as colorless oil. *Via* NMR spectroscopy rotameric effects were detected with a rotameric ratio of 2.9:1. Both rotamers are described together: **^{1}H NMR (600 MHz, CDCl$_3$):** δ 7.40 – 7.29 (m, 2.8H), 7.22 – 7.13 (m, 1.2H), 6.94 (s, 0.2H), 6.85 – 6.83 (m, 0.8H), 6.76 (s, 1H), 5.35 (dd, *J* = 13.7, 7.3 Hz, 0.7H), 4.88 (d, *J* = 13.3 Hz, 0.3H), 3.70 – 3.64 (m, 0.3H), 3.24 (d, *J* = 11.5 Hz, 0.7H), 3.11 (t, *J* = 12.8 Hz, 1H), 3.03 – 2.99 (m, *J* = 7.6 Hz, 1.4H), 2.96 – 2.89 (m, 0.3H), 2.66 – 2.62 (m, *J* = 13.1, 2.7 Hz, 0.3H), 2.32 – 2.24 (m, 6.4H), 2.17 (s, 0.6H), 1.87 (s, 2H), 1.82 – 1.34 (m, 6H) ppm. **^{13}C NMR (151 MHz, CDCl$_3$):** δ 169.9, 169.8, 138.3 (2C), 137.8, 137.5, 134.0, 133.8, 133.5, 133.4, 133.3, 129.4, 129.0, 128.5, 128.4, 128.3 128.2, 128.0, 126.4, 126.4, 56.2, 48.6, 42.0, 37.0 (2C), 35.6, 30.9, 26.4, 26.2, 25.6, 21.1, 21.0 , 19.5, 19.2, 19.1, 19.0, 18.9, 18.4 ppm. **IR (neat) ν_{max}:** 2934, 2856, 1623, 1436, 1416, 1287, 1032, 852, 738, 698. **HRMS (ESI$^+$):** exact mass calculated for [M+H]$^+$ ($C_{22}H_{28}NO^+$) requires 322.2171 m/z found 322.2181 m/z.

Mesityl(2-methylpiperidin-1-yl)methanone (1l)

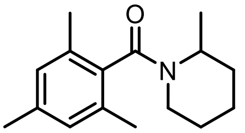

Following the GP1A using 2,4,6-trimethylbenzoyl chloride and 2-methylpiperidine afforded the desired amide (1.02 g, 83%) as colorless oil. The desired amide was obtained in a rotameric mixture with a ratio of around 2:1. Major isomer: **^{1}H NMR (600 MHz, CDCl$_3$):** δ 6.84 (s, 2H), 5.20 – 5.14 (m, 1H), 3.19 (dd, J = 13.5, 2.5 Hz, 1H), 3.04 (td, J = 13.3, 3.0 Hz, 1H), 2.26 (s, 6H), 2.14 (s, 3H), 1.75 – 1.46 (m, 6H), 1.28 (d, J = 7.0 Hz, 3H) ppm. Minor isomer: **^{1}H NMR (600 MHz, CDCl$_3$):** δ 6.81 (s, 2H), 4.80 – 4.69 (m, 1H), 3.76 – 3.67 (m, 1H), 2.86 (td, J = 13.3, 3.0 Hz, 1H), 2.27 (s, 6H), 2.23 (s, 3H), 1.75 – 1.46 (m, 6H), 1.14 (d, J = 6.9 Hz, 3H) ppm. The ^{13} C carbon signals of both rotamers could not be clearly differentiated. **^{13}C NMR (151 MHz, CDCl$_3$):** δ 169.8, 169.7, 137.7 (2C), 134.3, 134.2, 133.6 (2C), 133.5, 133.4, 128.6, 128.4, 128.2 (2C), 50.0, 43.4, 41.6, 36.2, 31.5, 30.1, 26.8, 26.0, 21.2, 19.5, 19.4, 19.2, 19.1 (2C), 18.8, 17.0, 15.7 ppm. **IR (neat) ν_{max}:** 2969, 2934, 2856, 1624, 1611, 1437, 1416, 1273, 1266, 1107, 1032, 850, 733. **HRMS (ESI$^+$):** exact mass calculated for [M+H]$^+$ ($C_{16}H_{24}NO^+$) requires 246.1858 m/z found 246.1842 m/z.

Mesityl(2-phenylpyrrolidin-1-yl)methanone (1m)

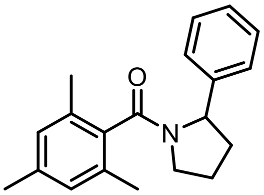

Following the GP1A using 2,4,6-trimethylbenzoyl chloride and 2-phenylpyrrolidine afforded the desired amide (1.21 g, 83%) as colorless oil. NMR spectroscopy analysis revealed two isomers in a ratio of around 1:1. Both isomers are described together: **^{1}H NMR (600 MHz, CDCl$_3$):** δ 7.36 – 7.33 (m, 2H), 7.26 – 7.23 (m, 0.5H), 7.18 – 7.13 (m, 1.5 H), 6.85 (s, 1H), 6.83 – 6.80 (m, 1H), 6.80 (s, 0.5H), 6.48 (s, 0.5H), 5.40 (dd, *J* = 8.1, 3.4 Hz, 0.5H), 4.45 (dd, *J* = 7.9, 3.4 Hz, 0.5 H), 4.09 – 4.04 (m, 0.5H), 3.90 – 3.83 (m, 0.5H), 3.37 – 3.32 (m, 1H), 3.25 – 3.20 (m, 0.5H), 2.39 – 2.30 (m, 1H), 2.28 (s, 3H), 2.23 (s, 3H), 2.20 (s, 1.5H), 2.05 – 1.83 (m, 3H), 1.56 (s, 1.5H) ppm. **^{13}C NMR (151 MHz, CDCl$_3$):** δ 171.3, 170.3, 143.6, 143.4, 138.0, 137.9, 135.2, 134.9, 134.6, 133.5, 133.0, 132.2, 128.6, 128.5, 128.4 (2C), 128.3, 127.8, 127.2, 127.0, 126.3, 126.1, 63.1, 60.40, 48.7, 46.6, 36.0, 34.7, 24.2, 22.8, 21.2 (2C), 19.3, 19.2, 19.1, 18.9 ppm. **IR (neat) ν_{max}:** 2971, 2923, 2868, 1620, 1611, 1447, 1437, 1401, 845, 701. **HRMS (ESI$^+$):** exact mass calculated for [M+H]$^+$ ($C_{20}H_{24}NO^+$) requires 294.1858 m/z found m/z 294.1866.

Mesityl((4aS,8aR)-octahydroquinolin-1(2H)-yl)methanone (1n)

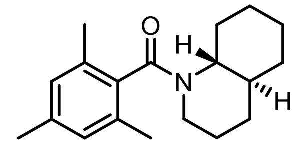

Following the GP1A using 2,4,6-trimethylbenzoyl chloride and (4aS,8aR)-decahydroquinoline afforded the desired amide (842 mg, 59%) as colorless crystals. **^{1}H NMR (600 MHz, CDCl$_3$):** δ 6.85 – 6.78 (m, 2H), 3.67 (td, *J* = 11.0, 3.0 Hz, 1H), 3.24 – 3.15 (m, 1H), 3.05 (dd, *J* = 13.5, 6.9 Hz, 1H), 2.51 – 2.42 (m, 1H), 2.24 (d, *J* = 2.7 Hz, 6H), 2.16 (s, 3H), 1.84 – 1.48 (m, 8H), 1.35 – 1.05 (m, 4H) ppm. **^{13}C NMR (151 MHz, CDCl$_3$):** δ 170.6, 137.6, 134.7, 133.7, 133.6, 128.4, 128.1, 60.5, 40.0, 38.3, 33.0, 29.9, 26.4, 25.9, 25.4, 23.5, 21.1, 19.2, 18.6 ppm. **IR (neat) ν_{max}:** 2923, 3856, 620, 1440, 1416, 1360, 1266, 1251, 1170, 1096, 1010, 851, 732, 701, 565. **HRMS (ESI$^+$):** exact mass calculated for [M+H]$^+$ ($C_{19}H_{28}NO^+$) requires m/z 286.2165 found m/z 286.2176.

N-Cyclohexyl-N-ethyl-2,4,6-trimethylbenzamide (1o)

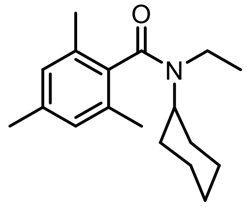

Following the GP1A using 2,4,6-trimethylbenzoyl chloride and N-ethylcyclohexanamine afforded the desired amide (1.05 g, 77%) as colorless oil. NMR spectroscopy analysis revealed two rotamers in a ratio of 2:1. Both rotamers are described together: **^{1}H NMR (400 MHz, CDCl$_3$):** δ 6.81 (s, 2H), 4.48 (t, *J* = 12.0 Hz, 0.4H), 3.49 (q, *J* = 6.8 Hz, 1.2H), 3.18 (t, *J* = 11.8 Hz, 0.6 H), 3.06 (q, *J* = 7.0 Hz, 0.8H), 2.27 (s, 3H), 2.21 (s, 6H), 1.95 – 1.76 (m, 2H), 1.75 – 1.40 (m, 6H), 1.31 (t, *J* = 6.9 Hz, 2H), 1.08 – 0.85 (m, 4H) ppm. **^{13}C NMR (151 MHz, CDCl$_3$):** δ 171.2, 170.8, 137.7, 137.4, 135.0, 134.5, 133.5, 133.4, 128.4, 59.0, 53.8, 39.5, 36.2, 32.3, 31.1, 26.1, 25.9, 25.8, 25.4, 21.2, 19.2 (2C), 16.1, 14.9 ppm. **IR (neat) ν_{max}:** 2971, 2928, 2855, 1626, 1612, 1466, 1450, 1413, 1369, 1307, 1153, 1076, 850. **HRMS (ESI$^+$):** exact mass calculated for [M+H]$^+$ ($C_{18}H_{28}NO^+$) requires 274.2171 m/z found 274.2175 m/z.

N-Butyl-N-ethyl-2,4,6-trimethylbenzamide (1p)

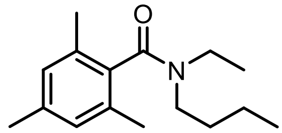

Following the GP1A using 2,4,6-trimethylbenzoyl chloride and N-ethylbutan-1-amine afforded the desired amide (1.16 g, 94%) as colorless oil. NMR spectroscopy analysis revealed two rotamers in a ratio of 1.2:1. Both rotamers are described together: **^{1}H NMR (600 MHz, CDCl$_3$):** δ 6.82 (d, 2H), 3.59 (q, *J* = 7.1 Hz, 0.9H), 3.54 – 3.48 (m, 1.1H), 3.10 (q, *J* = 7.1 Hz, 1.1H), 3.04 – 3.00 (m, 0.9H), 2.26 (d, 3.0H), 2.20 (d, 6.0H), 1.70 – 1.62 (tt, *J* = 7.8, 6.6 Hz, 1H), 1.45 – 1.36 (m, 2H), 1.25 (t, *J* = 7.1 Hz, 1.5H), 1.17 – 1.07 (m, 1H), 1.04 – 0.97 (m, 3.0H), 0.77 (t, *J* = 7.4 Hz, 1.5H) ppm. **^{13}C NMR (151 MHz, CDCl$_3$):** δ 171.0, 170.9, 137.7, 134.4, 134.2, 133.5, 128.3 (2C), 47.6, 43.6, 42.7, 38.9, 30.7, 29.7, 21.2, 20.7, 20.1, 19.2, 19.1, 14.1, 13.9, 13.8, 12.8 ppm. **IR (neat) ν_{max}:** 2959, 2931, 1627, 1612, 1455, 1417, 1377, 1287, 1099, 850, 748, 597, 584. **HRMS (ESI$^+$):** exact mass calculated for [M+H]$^+$ ($C_{16}H_{26}NO^+$) requires 248.2014 m/z found 248.2016 m/z.

((3S,4R)-3-((Benzo[d][1,3]dioxol-5-yloxy)methyl)-4-(4-fluorophenyl)piperidin-1-yl)(mesityl)methanone (1q)

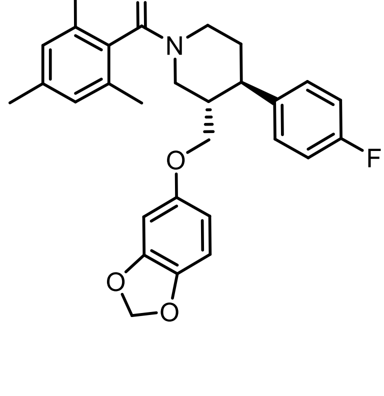

Paroxetine hydrochloride hemihydrate (329 mg, 1 mmol, 1.0 eq.) was dissolved together with triethylamine (418 µL, 3.0 eq.) in dichloromethane (3 mL). The mixture was cooled down to 0 °C and 2,4,6-trimethylbenzoyl chloride (500 µL, 3.0 eq.) was added dropwise. The reaction was allowed to come up to room temperature and was stirred for 16 h. Then, the organic phase was washed with a 1M HCl solution (10 mL) and brine solution (10 mL). The organic phase was dried over anhydrous $MgSO_4$, concentrated and the crude material was subjected to column chromatography giving rise to the title compound as a white solid (152 mg, 32%). NMR spectroscopy analysis revealed two rotamers in a ratio of

around 1:1. Both rotamers are described together: **^{1}H NMR (400 MHz, $CDCl_3$)**: δ 7.16 – 7.09 (m, 2H), 7.03 – 6.96 (m, 2H), 6.89 – 6.84 (m, 2H), 6.64 (d, *J* = 8.5 Hz, 0.5H), 6.58 (d, *J* = 8.5 Hz, 0.5H), 6.39 (d, *J* = 2.5 Hz, 0.5H), 6.17 (dd, *J* = 8.5, 2.5 Hz, 0.5H), 6.12 (d, *J* = 2.5 Hz, 0.5H), 5.94 (dd, *J* = 8.5, 2.5 Hz, 0.5H), 5.89 (d, *J* = 4.4 Hz, 2H), 5.18 (dd, *J* = 13.3, 2.5 Hz, 0.5H), 5.07 – 4.98 (m, 0.5H), 3.73 – 3.63 (m, 1H), 3.58 – 3.46 (m, 1.5H), 3.39 – 3.32 (m, 0.5H), 3.14 – 2.70 (m, 3H), 2.36 (s, 1.5H), 2.32 (s, 1.5H), 2.29 – 2.27 (m, 3H), 2.21 – 2.18 (s, 3H), 2.14 – 1.57 (m, 3H) ppm. **^{13}C NMR (101 MHz, $CDCl_3$):** δ 172.6, 170.2, 170.1, 163.0 (2C), 160.6 (2C), 154.4, 153.6, 148.3 (2C), 142.0, 141.9, 139.6, 138.7, 138.6 (3C), 138.2, 138.1, 135.7 (2C), 133.5 (2C), 133.3, 128.9 (2C), 128.8 (2C), 128.6 (2C), 128.5 (2C), 128.4 (2C), 115.9 (2C), 115.7 (2C), 108.0, 107.9, 105.8, 105.7, 101.3, 101.2, 98.2 (2C), 68.7, 68.3, 49.5, 46.7, 44.5, 44.2, 43.9, 43.0, 42.4, 41.8, 34.8, 33.9, 21.3, 21.2 (2C), 19.5, 19.4, 19.1 (2C) ppm. **^{19}F NMR (376 MHz, $CDCl_3$):** δ -115.7, -115.8 ppm. **IR (neat) ν_{max}:** 2919, 1609, 1509, 1487, 1468, 1445, 1225, 1178, 1038, 832. 730. **HRMS (ESI$^+$):** exact mass calculated for $[M+H]^+$ ($C_{29}H_{31}FNO_4^+$) requires m/z 476.2232 found m/z 476.2242.

(1-(3,4-Dimethoxybenzyl)-6,7-dimethoxy-3,4-dihydroisoquinolin-2(1H)-yl)(mesityl)methanone (1r)

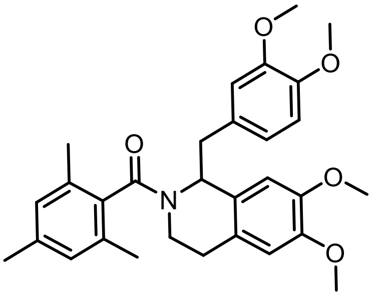

Norlaudanosine hydrochloride (570 mg, 1.50 mmol, 1.00 eq.) was dissolved in 5 mL of CH_2Cl_2 together with triethylamine (418 µL, 3.00 mmol, 2.00 eq.). The mixture was cooled to 0 °C and 2,4,6-trimethylbenzoyl chloride (250 µL, 1.5 mmol, 1.0 eq.) was added dropwise. The reaction was allowed to warm to room temperature and was stirred for 16 h. Then, the organic phase was sequentially washed with a 1 M HCl solution (10 mL) and brine (10 mL). The organic phase was dried over $MgSO_4$, concentrated and the crude material was subjected to column chromatography. In the column chromatography two compounds were isolated. **Compound A** (R_f = 0.27 in EtOAc/heptane, 1:1): white solid, 433 mg (59%). **Compound B** (R_f = 0.17 in EtOAc/heptane, 1:1): white solid, 174 mg (24%). Under careful analysis of spectroscopic data, we concluded that both compounds correspond to the title compound. We assume that **A** and **B** are atropisomers. This assumption is supported by the observation that, overtime in solution (in $CDCl_3$ over several hours), **A** and **B** formed an equilibrium (ratio: 2.9:1 (A:B)).

Compound A: ^{1}H NMR (400 MHz, $CDCl_3$): δ 6.87 (s, 1H), 6.84 (s, 1H), 6.80 (s, 1H), 6.75 (d, *J* = 8.1 Hz, 1H), 6.68 (d, *J* = 8.7 Hz, 1H), 6.55 (s, 1H), 6.23 (s, 1H), 5.99 (t, *J* = 7.0 Hz, 1H), 3.86 – 3.80 (m, 9H), 3.62 (s, 3H), 3.40 – 3.22 (m, 3H), 3.06 (dd, *J* = 13.5, 8.0 Hz, 1H), 2.78 – 2.69 (m, 1H), 2.56 (dt, *J* = 16.1, 3.7 Hz, 1H), 2.27 (s, 3H), 2.14 (s, 3H), 2.02 (s, 3H) ppm. **^{13}C NMR (101 MHz, $CDCl_3$):** δ 170.3, 149.0, 147.9 (2C), 147.1, 138.0, 133.8 (2C), 133.5, 130.7, 128.6, 128.4, 128.2, 125.1, 122.2, 113.0, 111.2, 111.1, 110.9, 56.1, 56.0, 55.9, 55.8, 52.9, 42.3, 40.8, 28.8, 21.2, 19.2, 18.7 ppm. **IR (neat) ν_{max}:** 1627, 1610, 1513, 1439, 1418, 1256, 1224, 1173, 1156, 1138, 1125, 1106, 1027, 853. **HRMS (ESI$^+$):** exact mass calculated for $[M+Na]^+$ ($C_{30}H_{35}NO_5Na^+$) requires m/z 512.2413, found m/z 512.2392.

Compound B: ^{1}H NMR (400 MHz, $CDCl_3$): δ 6.93 (s, 1H), 6.81 (s, 1H), 6.66 (d, *J* = 8.1 Hz, 1H), 6.62 (s, 1H), 6.29 (d, *J* = 8.1 Hz, 1H), 6.06 (s, 1H), 5.71 (s, 1H), 4.90 – 4.80 (m, 1H), 4.53 (dd, *J* = 8.8, 4.6 Hz, 1H), 3.85 (s, 3H), 3.81 (s, 3H), 3.62 (s, 4H), 3.46 (s, 3H), 3.34 – 3.25 (m, 1H), 3.10 – 2.96 (m, 2H), 2.89 – 2.74 (m, 2H), 2.29 (s, 3H), 2.20 (s, 3H), 1.87 (s, 3H) ppm. **^{13}C NMR (101 MHz, $CDCl_3$):** δ 170.1, 149.1, 148.0 (2C), 146.6, 138.1, 134.3, 134.0, 133.7, 130.4, 128.7, 128.3, 128.2, 125.9, 122.1, 111.9, 111.5, 111.1, 110.2, 60.5, 59.4, 56.1, 55.9, 55.7, 43.9, 36.1, 28.0, 21.2, 19.4, 18.7 ppm. **IR (neat) ν_{max}:** 1630, 1611, 1514, 1440, 1416, 1256, 1226, 1156, 1138, 1123, 1106, 1027, 854. **HRMS (ESI$^+$):** exact mass calculated for $[M+H]^+$ ($C_{30}H_{35}NO_5Na^+$) requires m/z 512.2413, found m/z 512.2396.

Pyrrolidin-1-yl(2,4,6-triisopropylphenyl)methanone (3a)

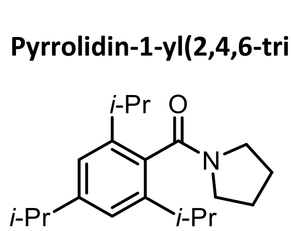

Following the GP2B (4 mmol scale) using 2,4,6-triisopropylbenzoic acid and pyrrolidine afforded the desired amide (0.99 g, 82%) as white solid. **^{1}H NMR (400 MHz, $CDCl_3$):** δ 6.98 (s, 2H), 3.67 (t, *J* = 7.1 Hz, 2H), 3.08 (t, *J* = 6.7 Hz, 2H), 2.91 – 2.77 (m, 3H), 1.95 (quint, 6.7 Hz, 2H), 1.83 (quint, *J* = 6.7 Hz, 2H), 1.24 – 1.20 (m, 18H) ppm. **^{13}C NMR (101 MHz, $CDCl_3$):** δ 170.1, 149.3, 143.9,

133.8, 121.2, 48.5, 45.0, 34.5, 31.2, 26.0, 25.4, 24.8, 24.2, 23.7 ppm. **IR (neat) ν_{max}:** 2958, 2931, 2869, 1629, 1607, 1460, 1415, 1361, 763, 749. **HRMS (ESI$^+$):** exact mass calculated for $[M+Na]^+$ ($C_{20}H_{31}NNaO^+$) requires m/z 324.2298, found m/z 324.2299.

Anthracen-9-yl(pyrrolidin-1-yl)methanone (3b)

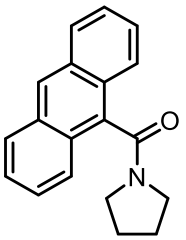

Following the GP2B (2 mmol scale) using 9-anthracenecarboxylic acid and pyrrolidine afforded the desired amide (0.36 g, 83%) as yellow solid. **^{1}H NMR (400 MHz, $CDCl_3$):** δ 8.46 (s, 1H), 8.04 – 8.00 (m, 2H), 7.93 (d, *J* = 8.5 Hz, 2H), 7.54 – 7.45 (m, 4H), 3.98 (t, *J* = 7.1 Hz, 2H), 2.93 (t, *J* = 6.8 Hz, 2H), **2.08 – 2.01** (m, 2H), 1.80 (quint, *J* = 6.8 Hz, 2H) ppm. Spectral data was found in agreement with the literature with one deviation (bold) in chemical shift.[5]

Phenyl(pyrrolidin-1-yl)methanone (3c)

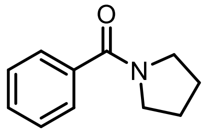

Following the GP1B (6 mmol scale) using benzoyl chloride and pyrrolidine the desired amide (919 mg, 87%) was obtained as colorless oil. ^{1}H NMR (400 MHz, $CDCl_3$) δ 7.54 – 7.48 (m, 2H), 7.43 – 7.37 (m, 3H), 3.65 (t, *J* = 6.9 Hz, 2H), 3.42 (t, *J* = 6.5 Hz, 2H), 2.03 – 1.83 (m, 4H) ppm. Spectra data was found in agreement with the literature.[6]

Phenyl(piperidin-1-yl)methanone (3d)

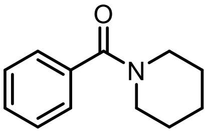

Following the GP1A using benzoyl chloride and piperidine afforded the desired amide (1.06 g, 98%) as colorless oil. **^{1}H NMR (400 MHz, $CDCl_3$):** δ 7.41 – 7.37 (m, 5H), 3.80 – 3.26 (m, 4H), 1.82 – 1.48 (m, 6H) ppm. Spectral data was found in agreement with the literature.[7]

Piperidin-1-yl(o-tolyl)methanone (3e)

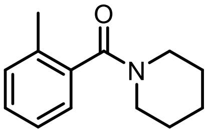

Following the GP1A using 2-methylbenzoyl chloride and piperidine afforded the desired amide (0.97 g, 95%) as colorless oil. **^{1}H NMR (400 MHz, $CDCl_3$):** δ 7.17 – 6.99 (m, 4H), 3.74 – 3.51 (m, 2H), 3.06 – 3.02 (m, 2H), 2.18 (s, 3H), 1.58 – 1.51 (m, 4H), 1.33 (app s, 2H) ppm. Spectral data was found in agreement with the literature.[8]

Piperidin-1-yl(p-tolyl)methanone (3f)

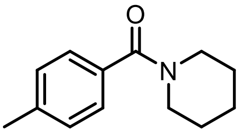

Following the GP1A using 4-methylbenzoyl chloride and piperidine afforded the desired amide (0.97 g, 95%) as colorless oil. **^{1}H NMR (400 MHz, $CDCl_3$):** δ 7.29 (d, *J* = 7.9 Hz, 2H), 7.19 (d, *J* = 7.8 Hz, 2H), 3.68 (app s, 2H), 3.36 (app s, 2H), 2.37 (s, 3H), 1.71 – 1.46 (m, 6H) ppm. Spectral data was found in agreement with the literature.[9]

(2-Methoxyphenyl)(piperidin-1-yl)methanone (3g)

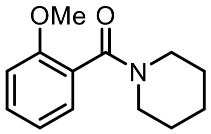

Following the GP1A using 2-methoxybenzoyl chloride and piperidine afforded the desired amide (1.18 g, 99%) as colorless oil. **^{1}H NMR (400 MHz, $CDCl_3$):** δ 7.32 (dd, *J* = 11.3, 4.5 Hz, 1H), 7.21 (dd, J = 7.4, 1.4 Hz, 1H), 6.97 (t, J = 7.5 Hz, 1H), 6.90 (d, J = 8.3 Hz, 1H), 3.83 (s, 3H), 3.80 – 3.63 (m, 2H), 3.21 – 3.15 (m, 2H), 1.72 – 1.58 (m, 6H) ppm. Spectral data was found in agreement with the literature.[10]

(3-Methoxyphenyl)(piperidin-1-yl)methanone (3h)

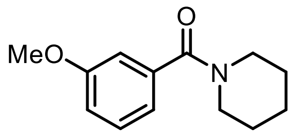

Following the GP1A using 3-methoxybenzoyl chloride and piperidine afforded the desired amide (1.00 g, 91%) as colorless oil. **^{1}H NMR (400 MHz, CDCl$_3$):** δ 7.32 – 7.27 (m, 1H), 6.96 – 6.90 (m, 3H), 3.82 (s, 2H), 3.67 (s, 2H), 3.34 (s, 3H), 1.70 –1.57 (m, 6H) ppm. Spectral data was found in agreement with the literature.[8]

(4-Methoxyphenyl)(piperidin-1-yl)methanone (3i)

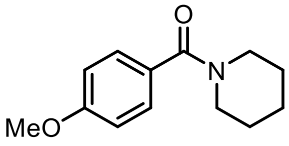

Following the GP1A using 4-methoxybenzoyl chloride and piperidine afforded the desired amide (0.97 g, 89%) as colorless oil**. ^{1}H NMR (400 MHz, CDCl$_3$)**: δ 7.37 (d, *J* = 8.6 Hz, 2H), 6.90 (d, *J* = 8.6 Hz, 2H), 3.83 (s, 3H), 3.75 – 3.28 (m, 4H), 1.74 – 1.58 (m, *J* = 37.6 Hz, 6H) ppm. Spectral data was found in agreement with the literature.[11]

Pyrrolidin-1-yl(3,4,5-trimethoxyphenyl)methanone (3j)

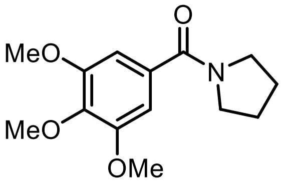

Following the GP1A using 3,4,5-trimethoxybenzoyl chloride and pyrrolidine afforded the desired amide (0.55 g, 42%) as colorless oil. **^{1}H NMR (400 MHz, CDCl$_3$):**) δ 6.76 – 6.71 (m, 2H), 3.87 – 3.83 (m, 9H), 3.66 – 3.57 (m, 2H), 3.49 – 3.40 (m, 2H), 2.02 – 1.79 (m, 4H) ppm. **^{13}C NMR (101 MHz, CDCl$_3$):** δ 169.5, 153.2, 139.4, 132.7, 104.7, 61.0, 56.4, 49.9, 46.4, 26.5, 24.6 ppm. **IR (neat) ν_{max}:** 2967, 2875, 2835, 1620, 1579, 1506, 1452, 1409, 1343, 1318, 1233, 1183, 1119, 1003, 866, 819, 761, 707, 676. **HRMS (ESI$^+$):** exact mass calculated for $[M+H]^+$ ($C_{14}H_{20}NO_4^+$) requires m/z 266.1387, found m/z 266.1389.

Piperidin-1-yl(3,4,5-trimethoxyphenyl)methanone (3k)

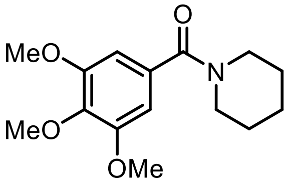

Following the GP1A using 3,4,5-trimethoxybenzoyl chloride and piperidine afforded the desired amide (0.81 g, 58%) as colorless oil. **^{1}H NMR (400 MHz, CDCl$_3$):** δ δ 6.61 (s, 2H), 3.90 – 3.80 (m, 9H), 3.74 – 3.30 (m, 4H), 1.77 – 1.50 (m, 6H) ppm. Spectral data was found in agreement with the literature.[12]

Benzo[d][1,3]dioxol-5-yl(piperidin-1-yl)methanone (3l)

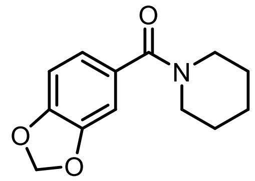

Following the GP1A using benzo[d][1,3]dioxole-5-carbonyl chloride and piperidine afforded the desired amide (1.19 g, 89%) as colorless oil. **^{1}H NMR (400 MHz, CDCl$_3$):** δ 6.93 – 6.87 (m, 2H), 6.82 – 6.79 (m, 1H), 5.99 (s, 2H), 3.76 – 3.30 (m, 4H), 1.69 – 1.53 (m, 6H) ppm. Spectral data was found in agreement with the literature.[13]

(2-Bromo-5-methoxyphenyl)(pyrrolidin-1-yl)methanone (3m)

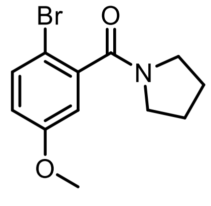

Following the GP2A using 2-bromo-5-methoxybenzoic acid and pyrrolidine afforded the desired amide (1.25 g, 86%) as yellwoish oil. **^{1}H NMR (400 MHz, CDCl$_3$):** δ 7.42 (d, *J* = 8.7 Hz, 1H,), 6.81 (s, 1H), 6.78 (d, *J* = 8.8 Hz, 1H), 3.78 (s, 3H), 3.65 (t, *J* = 6.7 Hz, 2H), 3.24 – 3.16 (m, 2H), 2.00 – 1.85 (m, 4H) ppm. **^{13}C NMR (101 MHz, CDCl$_3$):** δ 167.4, 159.3, 140.4, 133.7, 116.7, 112.8, 109.2, 55.7, 48.1, 45.7, 26.1, 24.7 ppm. **IR (neat) ν_{max}:** 2971, 2877, 2629, 1593, 1569, 1450, 1431, 1395, 1387, 1288, 1274, 1236, 1016, 820, 730, 639, 598. **HRMS (ESI$^+$):** exact mass calculated for $[M+H]^+$ ($C_{12}H_{15}BrNO_2^+$) requires m/z 284.0286, found m/z 284.0267.

(4-Fluorophenyl)(pyrrolidin-1-yl)methanone (3n)

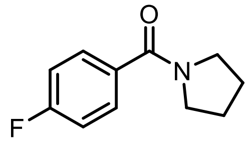

Following the GP1A using 4-fluorobenzoyl chloride and pyrrolidine afforded the desired amide (0.72 g, 75%) as white solid. **^{1}H NMR (400 MHz, CDCl$_3$):** δ 7.56 – 7.47 (m, 2H), 7.08 – 7.01 (m, 2H), 3.62 (t, *J* = 6.9 Hz, 2H), 3.41 (t, *J* = 6.5 Hz, 2H), 1.98 – 1.82 (m, 4H) ppm. Spectral data was found in agreement with the literature.[14]

(3-Chlorophenyl)(pyrrolidin-1-yl)methanone (3o)

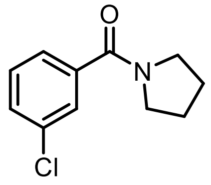

Following the GP1A using 3-chlorobenzoyl chloride and pyrrolidine afforded the desired amide (0.83 g, 79%) as colorless liquid. **^{1}H NMR (600 MHz, CDCl$_3$):** δ 7.52 – 7.48 (m, 1H), 7.41 – 7.36 (m, 2H), 7.36 – 7.31 (m, 1H), 3.64 (t, *J* = 6.9 Hz, 2H), 3.41 (t, *J* = 6.5 Hz, 2H), 2.01 – 1.84 (m, 4H) ppm. **^{13}C NMR (151 MHz, CDCl$_3$):** δ 168.3, 139.1, 134.5, 130.0, 129.8, 127.5, 125.4, 49.7, 46.4, 26.5, 24.6 ppm. **IR (neat) ν_{max}:** 2972, 2876, 1620, 1565, 1427, 1400, 743, 702. **HRMS (ESI$^+$):** exact mass calculated for $[M+H]^+$ ($C_{11}H_{13}ClNO^+$) requires m/z 210.0680 found m/z 210.684.

(2,3-Dihydro-1H-pyrrol-1-yl)(4-vinylphenyl)methanone (3p)

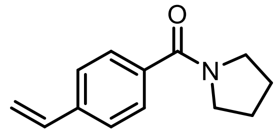

Following the GP2A (2.8 mmol scale) using 4-vinylbenzoic acid and pyrrolidine afforded the desired amide (0.39 g, 70%) as white solid. **^{1}H NMR (400 MHz, CDCl$_3$):** δ 7.49 (d, *J* = 8.2 Hz, 2H), 7.42 (d, *J* = 8.2 Hz, 2H), 6.72 (dd, *J* = 17.6, 10.9 Hz, 1H), 5.79 (d, *J* = 17.6 Hz, 1H), 5.30 (d, *J* = 10.9 Hz, 1H), 3.64 (t, *J* = 6.9 Hz, 2H), 3.43 (t, *J* = 6.5 Hz, 2H), 2.01 – 1.82 (m, 4H) ppm. **^{13}C NMR (101 MHz, CDCl$_3$)**: δ 169.5, 139.1, 136.6, 136.3, 127.6, 126.1, 115.3, 49.7, 46.3, 26.6, 24.6 ppm. **IR (neat) ν_{max}:** 2974, 2869, 1603, 1427, 999, 902, 855, 847, 772, 729. **HRMS (ESI$^+$):** exact mass calculated for $[M+H]^+$ ($C_{13}H_{16}NO^+$) requires m/z 202.1226 found m/z 202.1228.

(4-(Methylthio)phenyl)(piperidin-1-yl)methanone (3q)

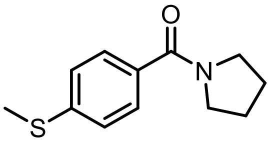

Following the GP2A (1.6 mmol scale) using 4-(methylthio)benzoic acid and pyrrolidine afforded the desired amide (0.28 g, 78%) as white solid. **^{1}H NMR (400 MHz, $CDCl_3$):** δ 7.45 (d, *J* = 8.4 Hz, 2H), 7.23 (d, *J* = 8.4 Hz, 2H), 3.62 (t, *J* = 6.8 Hz, 2H), 3.44 (t, *J* = 6.5 Hz, 2H), 2.48 (s, 3H), 2.01 – 1.76 (m, 4H) ppm. **^{13}C NMR (101 MHz, $CDCl_3$):** δ 169.3, 141.2, 133.6, 127.9, 125.6, 49.8, 46.4, 26.6, 24.6, 15.4 ppm. **IR (neat) ν_{max}:** 2969, 2866, 1593, 1550, 1420, 831, 752. **HRMS (ESI$^+$):** exact mass calculated for $[M+H]^+$ ($C_{12}H_{16}NOS^+$) requires m/z 222.0947 found m/z 222.0948.

3-(Pyrrolidine-1-carbonyl)benzonitrile (3r)

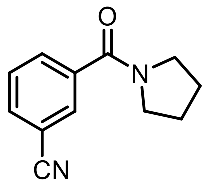

Following the GP2A using 3-cyanobenzoic acid and pyrrolidine afforded the desired amide (0.27 g, 27%) as colorless oil. **^{1}H NMR (400 MHz, $CDCl_3$):** δ 7.81 (d, *J* = 1.2 Hz, 1H), 7.76 (dd, *J* = 7.8, 1.3 Hz, 1H), 7.70 (dd, *J* = 7.8, 1.2 Hz, 1H), 7.53 (t, *J* = 7.8 Hz, 1H), 3.65 (t, *J* = 6.8 Hz, 2H), 3.41 (t, *J* = 6.5 Hz, 2H), 1.95 (app ddd, *J* = 19.2, 13.2, 6.7 Hz, 4H) ppm. **^{13}C NMR (101 MHz, $CDCl_3$):** δ 167.3, 138.6, 133.4, 131.6, 130.9, 129.5, 118.3, 112.8, 49.7, 46.6, 26.6, 24.5 ppm. **IR (neat) ν_{max}:** 2972, 2877, 2230, 1621, 1577, 1435, 1404, 1340, 1186, 1173, 912, 815, 797, 745, 723, 691, 628. **HRMS (ESI$^+$):** exact mass calculated for $[M+H]^+$ ($C_{12}H_{13}N_2O^+$) requires m/z 201.1028 found 201.1015.

1-(Pyrrolidine-1-carbonyl)ferrocene (3u)

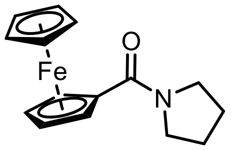

Following the GP2B (0.7 mmol scale) using ferrocenecarboxylic acid and pyrrolidine afforded the desired amide (174 mg, 88%) as orange solid. **^{1}H NMR (700 MHz, $CDCl_3$):** δ 4.73 – 4.72 (m, 2H), 4.33 – 4.31 (m, 2H), 4.20 (s, 5H), 3.69 (t, *J* = 6.2 Hz, 2H), 3.60 (t, *J* = 6.4 Hz, 2H), 1.99 – 1.94 (m, 2H), 1.92 – 1.88 (m, 2H) ppm. **^{13}C NMR (101 MHz, $CDCl_3$):** δ 169.4, 78.0, 70.4, 70.0, 69.7, 48.2, 47.2, 26.9, 24.1 ppm. Spectral data was found in agreement with the literature.[15]^[15]^[15]

(4-Hydroxy-3-methoxyphenyl)(pyrrolidin-1-yl)methanone (Precursor for **3v**)

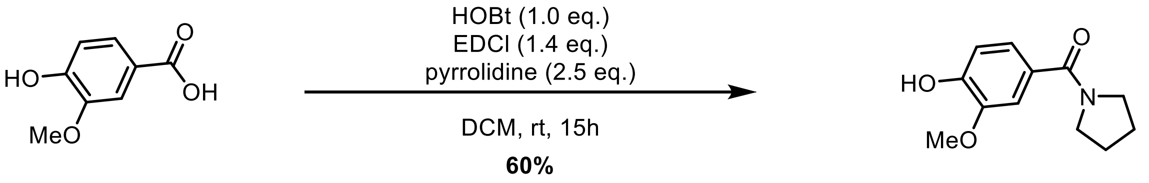

An oven-dried flask was loaded with (4-hydroxy-3-methoxyphenyl)(pyrrolidin-1-yl)methanone (1.68 g, 10 mmol, 1.00 eq.) and 1-hydroxybenzotriazole hydrate (1.35 g, 10 mmol, 1.00 eq.). Anhydrous CH_2Cl_2 (10 mL) was added, followed by pyrrolidine (2.05 mL, 25 mmol, 2.50 eq.) and 1-(3-dimethylaminopropyl)-3-ethylcarbodiimide hydrochloride (2.68 g, 14 mmol, 1.40 eq.). The resulting mixture was stirred for 15 h at room temperature. Then, a saturated solution of $NaHCO_3$ (10 mL) was added and the aqueous phase was extracted with CH_2Cl_2 (3 x 30 mL). The organic phases were combined and dried over $MgSO_4$. After concentration under reduced pressure, the crude material was subjected to column chromatography (heptane/EtOAc, 2:8 – pure EtOAc) to afford the title compound (1.33 g, 6.0 mmol, 60%) as white solid. **^{1}H NMR (700 MHz, $CDCl_3$):** δ 7.15 (s, 1H), 7.06 (dd, *J* = 8.1, 1.4 Hz, 1H), 6.89 (d, *J* = 8.1 Hz, 1H), 5.91 (s, 1H), 3.91 (s, 3H), 3.63 (t, *J* = 6.5 Hz, 2H), 3.50 (t, *J* = 6.0 Hz, 2H), 1.97 – 1.85 (m, 4H) ppm. **^{13}C NMR (176 MHz, $CDCl_3$):** δ 169.5, 147.4, 146.5, 129.1, 121.0, 113.7, 110.8, 56.2, 50.1, 46.6, 26.7, 24.6. **IR (neat) ν_{max}:** 1564, 1438, 1419, 1283, 1256, 1224, 1123, 1032, 878, 752, 726. **HRMS (ESI$^+$):** exact mass calculated for $[M+H]^+$ ($C_{12}H_{16}NO_3^+$) requires m/z 222.1125, found m/z 222.1125.

2-Methoxy-4-(pyrrolidine-1-carbonyl)phenyl 2-(3-cyano-4-isobutoxyphenyl)-4-methylthiazole-5-carboxylate (3v)

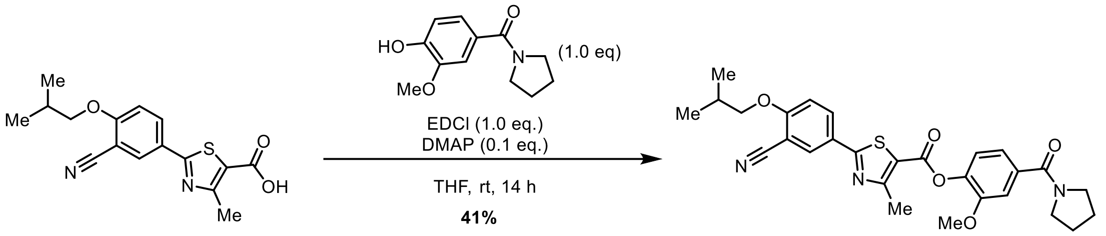

A modified procedure adopted from the literature was used.[16] An oven-dried flask was loaded with (4-hydroxy-3-methoxyphenyl)(pyrrolidin-1-yl)methanone (443 mg, 2.0 mmol, 1.00 eq.), febuxostat (633 mg, 2.0 mmol, 1.00 eq.), 1-(3-dimethylaminopropyl)-3-ethylcarbodiimide hydrochloride (383 mg, 1.0 mmol, 1.00 eq.) and 4-dimethylaminopyridine (24.4 mg, 0.10 mmol, 0.10 eq.). Anhydrous tetrahydrofuran (10 mL) was added and the mixture was stirred for 14 h at room temperature. Then, a saturated solution of $NaHCO_3$ was added and the aqueous phase was extracted with CH_2Cl_2 (3 x 10 mL). The organic phases were combined and dried over $MgSO_4$. After concentration under reduced pressure, the crude material was subjected to column chromatography (heptane/EtOAc, 8:2 – 2:8) to afford the title compound (425 mg, 0.82 mmol, 41%) as white solid. **^{1}H NMR (700 MHz, $CDCl_3$)**: δ 8.21 (d, *J* = 1.7 Hz, 1H), 8.11 (dd, *J* = 8.6, 1.8 Hz, 1H), 7.22 (s, 1H), 7.16 (d, *J* = 8.0 Hz, 1H), 7.13 – 7.10 (m, 1H), 7.02 (d, *J* = 8.9 Hz, 1H), 3.90 (d, *J* = 6.5 Hz, 2H), 3.85 (s, 3H), 3.64 (t, *J* = 6.9 Hz, 2H), 3.47 (t, *J* = 6.5 Hz, 2H), 2.80 (s, 3H), 2.24 – 2.17 (m, 1H), 1.99 – 1.94 (m, 2H), 1.92 – 1.87 (m, 2H), 1.08 (d, *J* = 6.7 Hz, 6H) ppm. **^{13}C NMR (176 MHz, $CDCl_3$)**: δ 168.9, 168.3, 163.2, 162.8, 159.8, 151.4, 140.4, 136.3, 132.8, 132.3, 126.0, 122.7, 120.4, 119.6, 115.4, 112.8, 112.1, 103.2, 75.8, 56.2, 49.9, 46.5, 28.3, 26.5, 24.5, 19.1, 17.8 ppm. **IR (neat) ν_{max}:** 2966, 2876, 2230, 1731, 1605, 1508, 1428, 1253, 1170, 1121, 1050, 908, 726. **HRMS (ESI$^+$):** exact mass calculated for $[M+H]^+$ ($C_{28}H_{30}N_3O_5S^+$) requires m/z 520.1901, found m/z 520.1901.

3.3. Characterization of Enamides

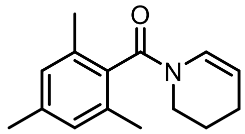

(3,4-Dihydropyridin-1(2H)-yl)(mesityl)methanone (2a)

Following GP3 using **1a** yielded the title compound (61.1 mg, 89%) as colorless oil. The desired enamide was obtained as two rotamers in a ratio of 3:1. Major isomer: **^{1}H NMR (400 MHz, $CDCl_3$):** δ 6.84 (s, 2H), 6.07 (d, *J* = 8.3 Hz, 1H), 4.86 – 4.76 (m, 1H), 3.94 – 3.84 (m, 2H), 2.27 (s, 3H), 2.17 (s, 6H), 2.13 – 2.05 (m, 2H), 1.94 – 1.85 (m, 2H) ppm. Minor isomer: **^{1}H NMR (400 MHz, $CDCl_3$):** δ 7.39 (d, *J* = 8.4 Hz, 1H), 6.84 (s, 2H), 5.23 – 5.18 (m, 1H), 3.26 – 3.20 (m, 2H), 2.27 (s, 3H), 2.19 (s, 6H), 2.13 – 2.06 (m, 2H), 1.81 – 1.58 (m, 2H) ppm. Major isomers: **^{13}C NMR (101 MHz, $CDCl_3$):** δ 169.6, 138.5, 134.0, 132.9, 128.3, 125.9, 108.5, 40.1, 22.1, 21.6, 21.2, 19.0 ppm. Minor isomer: **^{13}C NMR (101 MHz, $CDCl_3$):** δ 168.9, 138.3, 133.72, 133.4, 128.3, 123.9, 110.4, 44.5, 22.7, 22.3, 21.2, 19.0 ppm. **IR (neat) ν_{max}:** 2921, 1664, 1632, 1612, 1434, 1406, 1372, 1355, 1293, 1257, 1172, 992, 844. **HRMS (ESI$^+$):** exact mass calculated for $[M+Na]^+$ ($C_{15}H_{19}NNaO^+$) requires m/z 252.1359, found m/z 252.1355.

(2,3-Dihydro-1H-pyrrol-1-yl)(mesityl)methanone (2b)

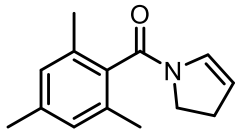

Following GP3 yielded the title compound (36.0 mg, 56%) as colorless oil. The desired enamide was obtained as 2 rotamers in a ratio of around 6:1. Major isomer: **^{1}H NMR (600 MHz, $CDCl_3$):** δ 6.84 (s, 2H), 5.90 (dt, *J* = 4.4, 2.2 Hz, 1H), 5.13 (dt, *J* = 4.4, 2.6 Hz, 1H), 4.09 – 3.97 (m, 2H), 2.73 (ddt, *J* = 11.3, 8.9, 2.4 Hz, 2), 2.27 (s, 3H), 2.19 (s, 6H) ppm. Minor isomer: **^{1}H NMR (600 MHz, $CDCl_3$):** δ 7.13 (dt, *J* = 4.4, 2.2 Hz, 1H), 6.85 (s, 2H), 5.34 (dt, *J* = 4.4, 2.6 Hz, 1H), 3.43 – 3.34 (m, 2H), 2.70 – 2.65 (m, 2H), 2.27 (s, 3H), 2.23

(s, 6H) ppm. Major isomer: **^{13}C NMR (151 MHz, $CDCl_3$):** δ 167.6, 138.6, 134.1, 133.7, 129.9, 128.4, 112.1, 44.4, 28.9, 21.2, 19.1 ppm. Minor isomer: **^{13}C NMR (151 MHz, $CDCl_3$):** δ 167.1, 138.4, 134.3, 133.3, 129.0, 128.5, 112.1, 46.4, 30.0, 21.2, 19.0 ppm. **IR (neat) ν_{max}:** 3360, 2952, 2921, 1635, 1610, 1439, 1404, 1327, 1213, 1169, 1122, 1100, 1042, 998, 651, 826, 748. **HRMS (ESI^+):** exact mass calculated for $[M+Na]^+$ ($C_{14}H_{17}NNaO^+$) requires m/z 328.1202, found m/z 328.1199.

Mesityl(2,3,4,5-tetrahydro-1H-azepin-1-yl)methanone (2c)

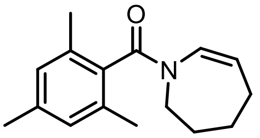

Following GP3 yielded the title compound (50.1 mg, 69%) as colorless oil. The desired enamide was obtained as two rotamers in a ratio of around 3.6:1. Major isomer: **^{1}H NMR (400 MHz, $CDCl_3$):** δ 6.84 (s, 2H), 5.90 (d, *J* = 8.5 Hz, 1H), 4.98 – 4.87 (m, 1H), 3.98 (app s, 2H), 2.31 – 2.18 (m, 11H), 1.93 (app s, 2H), 1.77 (app s, 2H) ppm. Minor isomer: **^{1}H NMR (400 MHz, $CDCl_3$):** δ 6.98 (d, *J* = 7.6 Hz, 1H), 6.85 (s, 2H), 5.33 (d, *J* = 5.2 Hz, 1H), 3.42 (app s, 2H), 2.35 – 2.15 (m, 11H), 1.77 (app s, 2H), 1.59 (app s, 2H) ppm. Major isomer: **^{13}C NMR (101 MHz, $CDCl_3$):** δ 170.6, 138.3, 134.2, 133.6, 130.5, 128.3, 117.3, 45.2, 27.8, 26.4, 25.6, 21.2, 19.1 ppm. Minor isomer: **^{13}C NMR (101 MHz, $CDCl_3$):** δ 170.9, 138.2, 133.8, 133.4, 129.5, 128.4, 118.3, 49.5, 30.4, 29.0, 26.6, 25.1, 19.1 ppm. **IR (neat) ν_{max}:** 2927, 1636, 1612, 1440, 1405, 1382, 1365, 1351, 1340, 1275, 1257, 849, 765, 750. **HRMS (ESI^+):** exact mass calculated for $[M+Na]^+$ ($C_{16}H_{21}NNaO^+$) requires m/z 266.1515, found m/z 266.1515.

Mesityl(1,4-dioxa-8-azaspiro[4.5]dec-6-en-8-yl)methanone (2d)

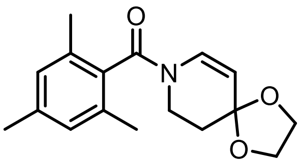

Following GP3 yielded the title compound (69.7 mg, 81%) as colorless oil. The desired enamide was obtained as two rotamers in a ratio of around 3.3:1. Major isomer: **^{1}H NMR (700 MHz, $CDCl_3$):** δ 6.85 (s, 2H), 6.25 (d, *J* = 8.5 Hz, 1H), 4.76 (d, *J* = 8.4 Hz, 1H), 4.09 – 4.04 (m, 2H), 4.04 – 3.97 (m, 4H), 2.27 (s, 3H), 2.15 (s, 6H), 2.06 – 2.00 (m, 2H) ppm. Minor isomer: **^{1}H NMR (700 MHz, $CDCl_3$):** δ 7.56 (d, *J* = 8.6 Hz, 1H), 6.85 (s, 2H), 5.14 (d, *J* = 8.6 Hz, 1H), 3.98 – 3.93 (m, 4H), 3.42 – 3.36 (m, 2H), 2.27 (s, 3H), 2.18 (s, 6H), 1.91 – 1.87 (m, 2H) ppm. Major isomer: **^{13}C NMR (176 MHz, $CDCl_3$)**: δ 170.0, 139.0, 134.2, 132.0, 129.8, 128.5, 108.0, 103.6, 64.7, 38.6, 32.4, 21.2, 19.0 ppm. Minor isomer: **^{13}C NMR (176 MHz, $CDCl_3$)**: δ 169.2, 138.7, 133.7, 132.7, 128.4, 127.3, 109.8, 103.6, 64.7, 42.7, 33.4, 21.2, 19.0 ppm. **IR (neat) ν_{max}:** 2881, 1665, 1632, 1591, 1369, 1293, 1224, 1170, 1119, 1041, 853, 745, 564. **HRMS (ESI^+):** exact mass calculated for $[M+H]^+$ ($C_{17}H_{22}NO_3{}^+$) requires 288.1600 m/z, found 288.1593 m/z.

(1,4,5,6,7,7a-Hexahydro-2H-isoindol-2-yl)(mesityl)methanone (2e)

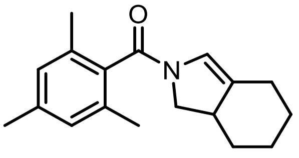

Following GP3 yielded the title compound (55.6 mg, 69%) as colorless oil. The desired enamide was obtained as two rotamers in a ratio of around 4.7:1. Both rotamers: **^{1}H NMR (600 MHz, $CDCl_3$):** δ 6.86 – 6.82 (m, 2H), 6.81 (t, *J* = 1.9 Hz, 0.2H), 5.56 (t, *J* = 1.8 Hz, 0.8H), 4.22 (dd, *J* = 12.8, 10.1 Hz, 0.8H), 3.56 – 3.49 (m, 1H), 2.93 (dd, *J* = 11.4, 6.6 Hz, 0.2H), 2.84 – 2.76 (m, *J* = 33.4 Hz, 1H), 2.27 – 2.18 (m, *J* = 52.9 Hz, 9H), 2.08 – 2.03 (m, *J* = 16.0 Hz, 1H), 1.94 – 1.88 (m, *J* = 34.7 Hz, 1H), 1.85 – 1.76 (m, *J* = 30.9 Hz, 2H), 1.40 – 1.10 (m, 4H) ppm. Both rotamers: **^{13}C NMR (151 MHz, $CDCl_3$):** δ 166.6, 166.2, 138.2, 138.1, 134.3, 134.0, 134.0, 133.8, 133.2,133.1, 130.2, 130.1, 128.3, 128.2, 120.2, 119.6, 53.2, 50.9, 43.1, 42.2, 34.7, 34.4, 27.4, 27.2, 25.9, 25.7, 25.3, 25.2, 21.1, 19.0, 18.9 (3C) ppm. **IR (neat) ν_{max}:** 2927, 2853, 1630, 1580, 1445, 1333, 1319, 1211, 1173, 851, 811, 751. **HRMS (ESI^+):** exact mass calculated for $[M+H]^+$ ($C_{18}H_{24}NO^+$) requires 270.1858 m/z, found 270.1852 m/z.

(2,3-Dihydro-4H-1,4-oxazin-4-yl)(mesityl)methanone (2f)

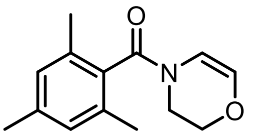

Following GP3 yielded the title compound as colorless oil (27.7 mg, 40%). In addition, remaining starting material (20.7 mg, 30%) was possible to be recovered. The desired enamide was obtained as two rotamers in a ratio of around 2.7:1. Major isomer: **^{1}H NMR (700 MHz, CDCl$_3$)**: δ 6.86 (s, 2H), 5.79 (d, *J* = 4.6 Hz, 1H), 5.52 (d, *J* = 4.4 Hz, 1H), 4.17 – 4.13 (m, 2H), 4.03 – 4.00 (m, 2H), 2.28 (s, 3H), 2.20 – 2.17 (m, 6H). Minor isomer: **^{1}H NMR (700 MHz, CDCl$_3$)**: δ 6.86 (s, 2H), 6.83 (d, *J* = 4.6 Hz, 1H), 6.19 (d, *J* = 4.7 Hz, 1H), 4.01 (s, 2H), 3.37 – 3.34 (m, 2H), 2.28 (s, 3H), 2.20 – 2.17 (m, 6H) ppm. Major isomer**: ^{13}C NMR (176 MHz, CDCl$_3$)**: δ 167.7, 138.9, 134.2, 131.9, 123.0, 128.4, 106.3, 64.9, 39.4, 21.3, 19.0 ppm. Minor isomer: **^{13}C NMR (176 MHz, CDCl$_3$):** δ 167.2, 138.8, 134.0, 132.5, 132.1, 128.6, 104.5, 65.1, 44.0, 21.2, 19.0 ppm. **IR (neat) ν_{max}:** 1633, 1409, 1379, 1215, 903, 852, 723, 649. **HRMS (ESI$^+$):** exact mass calculated for [M+H]$^+$ ($C_{14}H_{18}NO_2^+$) requires m/z 232.1332 found m/z 232.1328.

Ethyl 4-(2,4,6-trimethylbenzoyl)-3,4-dihydropyrazine-1(2H)-carboxylate (2g)

Following GP3 yielded the title compound (37.5 mg, 41%) as colorless oil. In addition, remaining starting material (53.0 mg, 58 %) was possible to be recovered. The desired enamide was obtained as four rotamers in a ratio of 4.8(A):4(B):1.9(C):1(D).

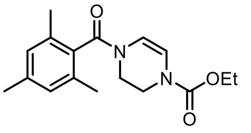
A or B

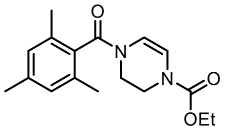
A or B

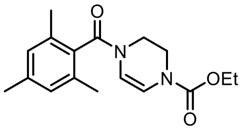
C or D

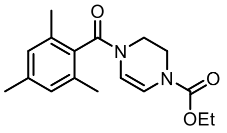
C or D

Rotamer A: **^{1}H NMR (700 MHz, CDCl$_3$):** δ 6.86 (s, 2H), 6.08 (d, *J* = 6.7 Hz, 1H), 5.49 (d, *J* = 6.7 Hz, 1H), 4.24 – 4.20 (m, 2H), 4.03 – 4.02 (m, 2H), 3.83 – 3.77 (m, 2H), 2.29 (s, 3H), 2.17 (s, 6H), 1.32 – 1.26 (m, 3H) ppm. **^{13}C NMR (176 MHz, CDCl$_3$):** δ 168.4, 152.3, 139.0, 134.2, 132.1, 128.5, 109.3, 108.2, 62.5, 41.0, 38.9, 21.3, 19.1, 14.7 ppm.
Rotamer B: **^{1}H NMR (700 MHz, CDCl$_3$):** δ 6.80 (s, 2H), 6.17 (d, *J* = 6.6 Hz, 1H), 5.51 (d, *J* = 6.6 Hz, 1H), 4.20 – 4.09 (m, 2H), 3.97 – 3.91 (m, 2H), 3.74 – 3.71 (m, 2H), 2.22 (s, 3H), 2.11 (s, 6H), 1.28 – 1.18 (m, 3H) ppm. **^{13}C NMR (176 MHz, CDCl$_3$):** δ 168.5, 152.6, 139.0, 134.2, 132.1, 128.5, 110.0, 108.9, 62.5, 41.6, 38.6, 21.3, 19.1, 14.7 ppm.
Rotamer C: **^{1}H NMR (700 MHz, CDCl$_3$):** δ 6.86 (s, 2H), 6.81 (d, *J* = 6.9 Hz, 1H), 6.48 (d, *J* = 6.9 Hz, 1H), 4.24 – 4.19 (m, 2H), 3.69 – 3.66 (m, 2H), 3.39 – 3.35 (m, 2H), 2.28 (s, 3H), 2.17 (s, 6H), 1.33 – 1.25 (m, 3H) ppm. **^{13}C NMR (176 MHz, CDCl$_3$):** only following signals could be clearly assigned to rotamer C: δ 167.9, 111.5, 106.5, 43.5, 41.6 ppm.
Rotamer D: **^{1}H NMR (700 MHz, CDCl$_3$):** δ 6.92 – 6.90 (m, 1H), 6.86 (s, 2H), 6.62 (d, *J* = 6.5 Hz, 1H), 4.24 – 4.19 (m, 2H), 3.69 – 3.66 (m, 2H), 3.39 – 3.35 (m, 2H), 2.28 (s, 3H), 2.17 (s, 6H), 1.33 – 1.25 (m, 3H) ppm. **^{13}C NMR (176 MHz, CDCl$_3$):** only following signals could be clearly assigned to rotamer D: δ 112.1, 107.3 ppm.
IR (neat) ν_{max}: 2980, 2922, 1887, 1710, 1648, 1612, 1416, 1374, 1343, 1275, 1227, 1117, 993. **HRMS (ESI$^+$):** exact mass calculated for [M+H]$^+$ ($C_{17}H_{23}N_2O_3^+$) requires 303.1709 m/z, found 303.1702 m/z.

N-Ethyl-2,4,6-trimethyl-N-vinylbenzamide (2h)

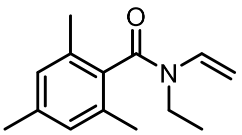

Following GP3 yielded the title compound (50.9 mg, 78%) as orange oil. The desired enamide was obtained as two rotamers in a ratio of around 9:1. Major isomer: **^{1}H NMR (400 MHz, CDCl$_3$):** δ 6.85 (s, 2H), 6.30 (dd, *J* = 15.6, 9.3 Hz, 1H), 4.49 (d, *J* = 15.7 Hz, 1H), 4.16 (d, *J* = 9.3 Hz, 1H), 3.89 (q, *J* = 7.1 Hz, 2H), 2.28 (s, 3H), 2.15 (s, 6H), 1.26 (t, *J* = 7.1 Hz, 3H) ppm. Minor isomer: **^{1}H NMR (400 MHz, CDCl$_3$):** δ 7.61 (dd, *J* = 16.3, 9.5 Hz, 1H), 6.86 (s, 2H), 4.59 (d, *J* = 10.9 Hz, 1H), 4.56 (d, *J* = 3.5 Hz, 1H), 3.39 (q, *J* = 7.1 Hz, 2H), 2.26 (s, 3H), 2.20 (s, 6H), 1.02 (t, *J* = 7.1 Hz, 3H) ppm. Major isomer: **^{13}C NMR (151 MHz, CDCl$_3$):** δ 170.5,

138.6, 134.0, 133.0, 132.9, 128.4, 93.2, 36.1, 21.2, 18.9, 11.8 ppm. Minor isomer: ^{13}C NMR could not be clearly identified. **IR (neat) ν_{max}:** 2935, 2921, 1667, 1618, 1461, 1446, 1425, 1345, 1310, 1076, 848, 607. **HRMS (ESI^{+}):** exact mass calculated for $[M+H]^+$ ($C_{14}H_{20}NO^+$) requires 218.1545 m/z, found 218.1537 m/z.

(E)-2,4,6-Trimethyl-N-(prop-1-en-1-yl)-N-propylbenzamide (2i)

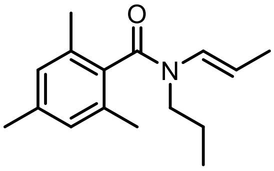

Following GP3 yielded the title compound (35.7 mg, 49%) as colorless oil. The desired enamide was obtained as two rotamers in a ratio of around 7.8:1. Major isomer: **^{1}H NMR (400 MHz, CDCl$_3$):** δ 6.84 (s, 2H), 6.05 (dd, *J* = 14.1, 1.3 Hz, 1H), 5.01 (dq, *J* = 13.4, 6.6 Hz, 1H), 3.79 – 3.69 (m, 2H), 2.29 (s, 3H), 2.14 (s, 6H), 1.75 – 1.65 (m, 2H), 1.54 (dd, *J* = 6.6, 1.2 Hz, 3H), 0.99 (t, *J* = 7.4 Hz, 3H) ppm. Minor isomer: **^{1}H NMR (400 MHz, CDCl$_3$):** δ 7.37 (d, *J* = 14.7 Hz, 1H), 6.81 (s, 2H), 5.13 (dq, J = 13.2, 6.6 Hz, 1H), 3.27 – 3.15 (m, 2H), 2.29 (s, 3H), 2.19 (s, 6H), 1.79 (dd, *J* = 6.6, 1.3 Hz, 3H), 1.74 – 1.64 (m, 2H), 0.71 (t, *J* = 7.4 Hz, 3H) ppm. Major isomer: **^{13}C NMR (101 MHz, CDCl$_3$):** δ 170.1, 138.3, 133.9, 133.5, 128.4, 127.9, 105.8, 43.8, 21.3, 20.1, 19.0, 15.6, 11.7 ppm. Minor isomer: **^{13}C NMR (101 MHz, CDCl$_3$):** δ 169.8, 138.3, 133.7, 133.6, 128.5, 125.8, 107.9, 47.9, 20.7, 19.3, 15.8, 11.5 ppm. One peak could not be detected presumably due to an overlapping with a peak of the major species. **IR (neat) ν_{max}:** 2960, 2935, 1640, 1612, 1443, 1398, 1373, 1323, 1226, 1084, 850. **HRMS (ESI^{+}):** exact mass calculated for $[M+Na]^+$ ($C_{16}H_{23}NNaO^+$) requires m/z 268.1672, found m/z 268.1668.

(E)-N-(But-1-en-1-yl)-N-butyl-2,4,6-trimethylbenzamide (2j)

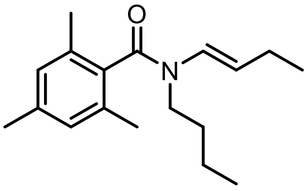

Following GP3 yielded the title compound (29.4 mg, 48%) as colorless oil. The desired enamide was obtained as two rotamers in a ratio of around 7.4:1. Both rotamers are described together: **^{1}H NMR (600 MHz, CDCl$_3$):** δ 7.37 (d, *J* = 14.7 Hz, 0.1H), 6.84 (s, 1.8H), 6.81 (s, 0.2H), 6.04 (d, *J* = 14.1 Hz, 0.9H), 5.18 – 5.13 (m, 0.1H), 5.04 (dt, *J* = 14.0, 6.9 Hz, 0.9H), 3.81 – 3.75 (m, 1.8H), 3.26 – 3.23 (m, 0.2H), 2.29 – 2.26 (m, *J* = 13.0 Hz, 3H), 2.20 – 2.14 (m, 6H), 1.92 – 1.85 (m, 2H), 1.68 – 1.63 (m, , 2H), 1.47 – 1.38 (m, 2H), 0.99 (t, *J* = 7.4 Hz, 3H), 0.88 (t, *J* = 7.4 Hz, 3H) ppm. **^{13}C NMR (151 MHz, CDCl$_3$):** δ 170.2, 138.3, 133.9, 133.5, 128.4, 126.7, 113.2, 42.0, 28.8, 23.7, 21.3, 20.6, 19.0, 15.0, 14.0 ppm. **IR (neat) ν_{max}:** 2959, 2931, 2872, 1395, 1325, 1090, 906, 729. **HRMS (ESI^{+}):** exact mass calculated for $[M+H]^+$ ($C_{18}H_{28}NO^+$) requires m/z 274.2165 found m/z 274.2162.

(2-Benzyl-3,4-dihydropyridin-1(2H)-yl)(mesityl)methanone (2k)

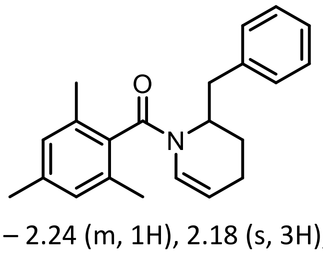

Following GP3 yielded the title compound (91.4 mg, 95%) as yellowish oil. The desired enamide was obtained as two rotamers in a ratio of around 10:1. Only the major rotamer is described: **^{1}H NMR (600 MHz, CDCl$_3$):** δ 7.36 (d, *J* = 7.1 Hz, 2H), 7.34 – 7.30 (m, 2H), 7.24 (t, *J* = 7.2 Hz, 1H), 6.85 (s, 1H), 6.08 (d, *J* = 8.4 Hz, 1H), 5.15 – 5.10 (m, 1H), 4.89 – 4.84 (m, 1H), 3.10 (dd, *J* = 13.1, 4.9 Hz, 1H), 2.76 (dd, *J* = 13.0, 10.8 Hz, 1H), 2.29 (s, 3H), 2.28 – 2.24 (m, 1H), 2.18 (s, 3H), 2.14 (s, 3H), 2.01 (dt, *J* = 18.1, 5.3 Hz, 1H), 1.79 – 1.75 (m, 1H), 1.65 – 1.58 (m, 1H) ppm. **^{13}C NMR (151 MHz, CDCl$_3$):** δ 169.2, 138.5, 138.4, 134.2, 134.1, 133.0, 129.6, 128.6, 128.4, 128.3, 126.5, 124.8, 107.7, 49.7, 36.2, 22.0, 21.3, 19.0, 18.9, 18.2 ppm. **IR (neat) ν_{max}:** 1627, 1412, 1358, 1270, 986, 852, 766, 731, 699, 514. **HRMS (ESI^{+}):** exact mass calculated for $[M+H]^+$ ($C_{22}H_{26}NO^+$) requires m/z 320.2014, found m/z 320.2006.

Mesityl(6-methyl-3,4-dihydropyridin-1(2H)-yl)methanone (2l)

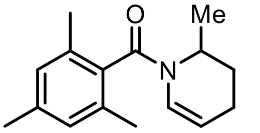

Following GP3 yielded the title compound (60.5 mg, 83%) as colorless oil. The desired enamide was obtained as two rotamers in a ratio of around 9:1. Major isomer: **^{1}H NMR (600 MHz, CDCl$_3$):** δ 6.83 (s, 2H), 6.04 – 5.95 (m, 1H), 5.03 – 4.91 (m, 1H), 4.82 – 4.72 (m, 1H), 2.29 – 2.24 (m, 3H), 2.22 – 2.12 (m, 7H), 2.05 – 1.98 (m, 1H), 1.84 – 1.76 (m, 2H), 1.24 (d, *J* = 6.7 Hz, 3H) ppm. Minor isomer: **^{1}H NMR (600 MHz, CDCl$_3$):** δ 7.32 (ddt, *J* = 8.5, 2.5, 1.3 Hz, 1H), 6.88 (s, 2H), 5.21 – 5.18 (m, 1H), 3.75 – 3.66 (m, 1H), 2.29 – 2.24 (m, 3H), 2.23 – 2.12 (m, 7H), 2.04 – 1.94 (m, 1H), 1.83 – 1.71 (m, 2H), 0.98 (d, *J* = 6.6 Hz, 3H) ppm. Major isomer: **^{13}C NMR (151 MHz, CDCl$_3$)**: δ 169.0, 138.4, 134.1, 134.0, 133.0, 128.3, 128.3, 124.4, 107.4, 44.1, 26.4, 21.2, 19.0, 18.8, 18.1, 16.5 ppm. Minor isomer: **^{13}C NMR (151 MHz, CDCl$_3$):** δ 168.6, 138.2, 134.2, 133.7, 133.1, 128.8, 128.0, 122.2, 109.6, 48.9, 27.9, 19.6, 19.0, 18.2, 17.0 ppm. One peak could not be detected presumably due to an overlapping with a peak of the major species. **IR (neat) ν_{max}:** 2972, 2923, 2850, 1664, 1629, 1411, 1357, 1281, 1039, 1012, 912, 876, 768, 720. **HRMS (ESI$^+$):** exact mass calculated for $[M+H]^+$ ($C_{16}H_{22}NO^+$) requires 244.1701 m/z, found 244.1695 m/z.

Mesityl(2-phenyl-2,3-dihydro-1H-pyrrol-1-yl)methanone (2m)

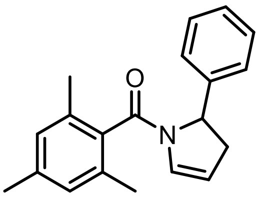

Following GP3 yielded the title compound (41.1 mg, 47%) as colorless oil. The desired enamide was obtained as two rotamers in a ratio of around 5.5:1. Both rotamers: **^{1}H NMR (400 MHz, CDCl$_3$):** δ 7.50 – 7.10 (m, 5H), 6.87 – 6.83 (m, 1.7H), 6.78 – 6.75 (m, 0.3H), 6.50 (app s, 0.2H), 6.08 (app s, 0.8H), 5.57 (dd, *J* = 10.8, 3.7 Hz, 0.8H), 5.42 (s, 0.2H), 5.18 – 5.12 (m, 0.8H), 4.58 (dd, *J* = 10.4, 3.1 Hz, 0.2H), 3.41 – 3.28 (m, 1H), 2.71 – 2.60 (m, 1H), 2.32 – 2.24 (m, 6H), 2.09 (s, 2.5H), 1.37 (s, 0.5H) ppm. Major isomer: **^{13}C NMR (101 MHz, CDCl$_3$):** δ 167.3, 143.5, 138.5, 134.4, 134.1, 133.6, 130.5, 128.8, 128.5, 128.3, 127.5, 126.1, 110.0, 59.8, 39.7, 21.2, 19.2, 18.9 ppm. Minor isomer: ^{13}C NMR could not be clearly identified. **IR (neat) ν_{max}:** 3030, 2920, 2858, 1642, 1610, 1389, 1341, 829, 756, 698. **HRMS (ESI$^+$):** exact mass calculated for $[M+H]^+$ ($C_{20}H_{22}NO^+$) requires m/z 292.1696, found m/z 292.1694.

((4aS,8aR)-4a,5,6,7,8,8a-Hexahydroquinolin-1(4H)-yl)(mesityl)methanone (2n)

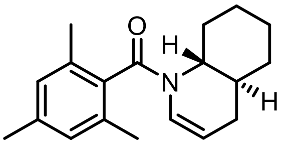

Following GP3 yielded the title compound as white solid (34.5 mg, 41%). No rotameric effects were observed *via* NMR spectroscopy. **^{1}H NMR (600 MHz, CDCl$_3$):** δ 6.82 (m, 2H), 6.01 (dd, *J* = 8.0, 2.9 Hz, 1H), 4.94 – 4.90 (m, 1H), 3.56 (td, *J* = 10.7, 2.9 Hz, 1H), 3.29 – 3.24 (m, 1H), 2.27 (s, 3H), 2.21 (s, 3H), 2.13 (s, 3H), 1.93 – 1.87 (m, 1H), 1.86 – 1.81 (m, 2H), 1.79 – 1.71 (m, 2H), 1.61 – 1.50 (m, 2H), 1.41 – 1.31 (m, 1H), 1.29 – 1.22 (m, 1H), 1.17 – 1.10 (m, 1H) ppm. **^{13}C NMR (151 MHz, CDCl$_3$)**: δ 170.8, 138.2, 134.5, 134.4, 133.1, 128.4, 128.3, 126.9, 109.3, 60.4, 41.9, 33.4, 31.5, 28.3, 26.5, 25.5, 21.2, 19.2, 18.8 ppm. **IR (neat) ν_{max}:** 2922, 2855, 1666, 1633, 1446, 1394, 1368, 1350, 1331, 1306, 1268, 1245, 1107, 1001, 850, 723. **HRMS (ESI$^+$):** exact mass calculated for $[M+H]^+$ ($C_{19}H_{26}NO^+$) requires m/z 284.2009 found m/z 284.2003.

N-Cyclohexyl-2,4,6-trimethyl-N-vinylbenzamide (2o)

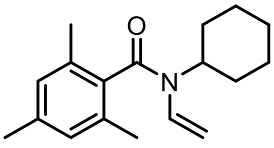

Following GP3 yielded the title compound (44.2 mg, 54%) as white solid. The desired enamide was obtained as two rotamers in a ratio of around 20:1. Only the major isomer is described: **^{1}H NMR (600 MHz, CDCl$_3$):** δ 6.82 (s, 2H), 6.18 (dd, *J* = 15.8, 9.3 Hz, 1H), 4.63 (dd, *J* = 15.8, 0.7 Hz, 1H), 4.31 – 4.28 (m, 2H), 2.27 (s, 3H), 2.25 – 2.18 (m, 2H), 2.16 (s, 6H), 1.89 – 1.85 (m, 2H), 1.81 – 1.74 (m, 2H), 1.72 – 1.65 (m, 1H), 1.44 – 1.36 (m, 2H), 1.27 – 1.20 (m, 1H) ppm. **^{13}C NMR (101 MHz, CDCl$_3$):** δ 170.9, 138.1, 134.3, 133.4, 133.2, 128.2, 98.4, 54.9, 29.2, 26.4, 25.5, 21.1, 18.8 ppm. **IR (neat)**

$\mathbf{v_{max}}$: 2930, 2856, 1659, 1611, 1435, 1342, 1316, 1218, 851, 729. **HRMS (ESI$^+$):** exact mass calculated for $[M+H]^+$ ($C_{18}H_{26}NO^+$) requires m/z 272.2014, found m/z 272.2010.

N-Butyl-2,4,6-trimethyl-N-vinylbenzamide
+ (E)-N-(but-1-en-1-yl)-N-ethyl-2,4,6-trimethylbenzamide (2p, 2p')

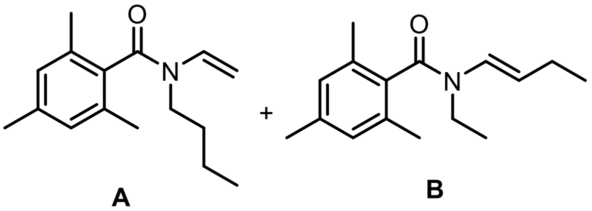

Following GP3 yielded the two title compounds (36.9 mg, 50%) as colorless oils in a ratio A:B of 1.8:1. Small amounts of each compound was possible to be isolated separately and was used for the full characterization.

Product A (2p): *Via* NMR spectroscopy two rotamers were observed in a ratio of around 12:1. Only the major isomer is described: **^{1}H NMR (600 MHz, CDCl$_3$):** δ 6.85 (s, 2H), 6.32 (dd, *J* = 15.6, 9.3 Hz, 1H), 4.47 (d, *J* = 15.6 Hz, 1H), 4.16 (d, *J* = 9.3 Hz, 1H), 3.83 – 3.77 (m, 2H), 2.28 (s, 3H), 2.15 (s, 6H), 1.70 – 1.64 (m, 2H), 1.46 – 1.41 (m, 2H), 0.99 (t, *J* = 7.4 Hz, 3H) ppm. **^{13}C NMR (151 MHz, CDCl$_3$):** δ 170.8, 138.6, 134.0, 133.5, 133.1, 128.4, 93.3, 41.2, 28.7, 21.3, 20.6, 19.0 14.0 ppm. **IR (neat) $\mathbf{v_{max}}$:** 2958, 2929, 2872, 1666, 1621, 1402, 1379, 1356, 1312, 1218, 1090, 851. **HRMS (ESI$^+$):** exact mass calculated for $[M+H]^+$ ($C_{16}H_{24}NO^+$) requires m/z 246.1852 found m/z 246.1846.

Product B (2p'): *Via* NMR spectroscopy two rotamers were observed in a ratio of around 5:1. Both rotamers are described together: **^{1}H NMR (400 MHz, CDCl$_3$):** δ 7.37 (dd, *J* = 14.5, 4.8 Hz, 0.2H), 6.84 (s, 2H), 6.03 (d, *J* = 14.1 Hz, 0.8H), 5.22 – 5.14 (m, 0.2H), 5.07 (dt, *J* = 14.0, 6.9 Hz, 0.8H), 3.87 (q, *J* = 7.1 Hz, 2H), 2.29 (s, 3H), 2.15 (s, 6H), 1.89 (quint, *J* = 6.3 Hz, 2H), 1.28 – 1.23 (m, 3H), 0.89 (t, *J* = 7.4 Hz, 3H) ppm. **^{13}C NMR (151 MHz, CDCl$_3$, major rotamer):** δ 170.0, 138.4, 133.9, 133.4, 128.4, 126.3, 113.1, 36.9, 23.7, 21.3, 19.0, 15.0, 12.1 ppm. Minor isomer: ^{13}C NMR could not be clearly identified. **IR (neat) $\mathbf{v_{max}}$:** 2961, 2922, 1641, 1612, 1451, 1396, 1323, 1082. **HRMS (ESI$^+$):** exact mass calculated for $[M+H]^+$ ($C_{16}H_{24}NO^+$) requires m/z 246.1852 found m/z 246.1846.

((3S,4R)-3-((Benzo[d][1,3]dioxol-5-yloxy)methyl)-4-(4-fluorophenyl)-3,4-dihydropyridin-1(2H)-yl)(mesityl)methanone (2q)

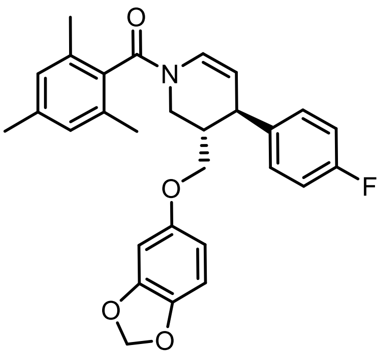

Following GP3 yielded title compound (37.2 mg, 28%) as colorless oil. The desired enamide was obtained as two rotamers in a ratio of around 4:1. Both rotamers are described together: **^{1}H NMR (600 MHz, CDCl$_3$):** δ 7.61 (dd, *J* = 8.4, 1.9 Hz, 0.2H), 7.18 – 7.12 (m, 2H), 7.03 – 6.97 (m, 2H), 6.90 – 6.84 (m, 2H), 6.69 (d, *J* = 8.4 Hz, 0.8H), 6.64 (d, *J* = 8.4 Hz, 0.2H), 6.47 (d, *J* = 2.4 Hz, 0.8H), 6.32 – 6.26 (m, 1.7H), 6.11 (dd, *J* = 8.5, 2.5 Hz, 0.3H), 5.92 – 5.90 (m, 2H), 5.20 (dd, *J* = 8.4, 3.3 Hz, 0.2H), 4.80 (dd, *J* = 8.3, 3.0 Hz, 0.8H), 4.40 (dd, *J* = 13.4, 3.6 Hz, 0.8H), 3.87 (dd, *J* = 9.3, 4.4 Hz, 0.7H), 3.79 – 3.71 (m, 1.7H), 3.66 – 3.59 (m, 1H), 3.51 – 3.49 (m, 0.3H), 3.42 (dd, *J* = 12.8, 3.2 Hz, 0.2H), 3.30 (dd, *J* = 12.8, 8.4 Hz, 0.2H), 2.33 – 2.25 (m, 6H), 2.20 – 2.16 (m, 4H) ppm. **^{13}C NMR (151 MHz, CDCl$_3$):** δ 169.9, 169.4, 162.8, 161.2, 154.4, 153.9, 148.5, 142.1, 139.0 (2C), 138.9, 138.7, 138.1, 134.2, 134.1, 133.9, 133.7, 132.8, 132.6, 129.7 (2C), 128.9, 128.6, 128.5, 128.3, 126.3, 124.5, 115.7, 115.6, 111.9, 110.6, 108.1, 108.0, 105.8, 105.4, 101.3, 98.3, 98.0, 68.7, 68.1, 44.7, 41.4, 41.1, 40.7, 40.6, 40.4, 21.3 (2C), 19.2 (2C), 19.1 (2C) ppm. **^{19}F NMR (376 MHz, CDCl$_3$):** δ -116.1, -116.2 ppm. **IR (neat) $\mathbf{v_{max}}$:** 1632, 1505, 1487, 1469, 1183, 1037, 732. **HRMS (ESI$^+$):** exact mass calculated for $[M+Na]^+$ ($C_{29}H_{28}FNO_4Na^+$) requires 496.1900 m/z found 496.1881 m/z.

(1-(3,4-Dimethoxybenzyl)-6,7-dimethoxyisoquinolin-2(1H)-yl)(mesityl)methanone (2r)

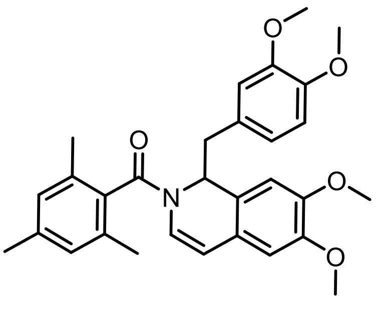

Following GP3 using Et_2O/DCM (3.5 mL, 5:1) yielded the title compound (80.9 mg, 55%) as white solid. The desired enamide was obtained as two rotamers in a ratio of around 13:1. Only the major isomer is described: **^{1}H NMR (600 MHz, $CDCl_3$):** δ 6.89 (s, 1H), 6.83 (s, 1H), 6.70 (d, *J* = 8.1 Hz, 1H), 6.65 (d, *J* = 1.6 Hz, 1H), 6.58 (s, 1H), 6.48 (dd, *J* = 8.1, 1.6 Hz, 1H), 6.04 (d, *J* = 7.8 Hz, 1H), 5.95 – 5.91 (m, 2H), 5.68 (d, *J* = 7.7 Hz, 1H), 3.85 (s, 3H), 3.83 (s, 3H), 3.80 (s, 3H), 3.53 (s, 3H), 3.03 (dd, *J* = 12.6, 4.9 Hz, 1H), 2.88 (dd, *J* = 12.5, 9.7 Hz, 1H), 2.29 (s, 3H), 2.23 (s, 3H), 2.03 (s, 3H) ppm. **^{13}C NMR (151 MHz, $CDCl_3$):** δ 169.8, 148.8, 148.3, 147.9, 147.3, 138.9, 134.6, 134.2, 132.5, 130.1, 128.5, 128.4, 124.3, 123.2, 122.8, 122.6, 113.3 (2C), 111.0, 110.1, 108.1, 56.1, 56.0 (2C), 55.8, 55.2, 40.4, 21.3, 18.9 (2C) ppm. **IR (neat) ν_{max}:** 2936, 1623, 1512, 1425, 1341, 1265, 1231, 1138, 1127, 1102, 912, 883, 728. **HRMS (ESI$^+$):** exact mass calculated for $[M+H]^+$ ($C_{30}H_{34}NO_5^+$) requires m/z 488.2431 found m/z 488.2428.

(2,3-Dihydro-1H-pyrrol-1-yl)(2,4,6-triisopropylphenyl)methanone (4a)

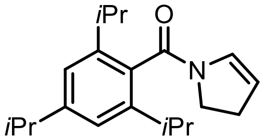

Following GP3 yielded the title compound (62.6 mg, 70%) as colorless oil. The desired enamide was obtained as two rotamers in a ratio of 7.1:1. Major isomer: **^{1}H NMR (700 MHz, $CDCl_3$):** δ 6.99 (s, 2H), 5.97 – 5.93 (m, 1H), 5.09 – 5.06 (m, 1H), 4.04 (t, *J* = 8.8 Hz, 2H), 2.97 – 2.65 (m, 5H), 1.23 (d, *J* = 6.8 Hz, 12H), 1.16 (d, *J* = 6.8 Hz, 6H) ppm. Minor isomer: **^{1}H NMR (700 MHz, $CDCl_3$):** δ 7.16 – 7.13 (m, 1H), 6.99 (s, 2H), 5.35 – 5.31 (m, 1H), 3.41 (t, *J* = 8.6 Hz, 2H), 2.97 – 2.65 (m, 5H), 1.23 (d, *J* = 6.8 Hz, 12H), 1.16 (d, *J* = 6.8 Hz, 6H) ppm. Major isomer: **^{13}C NMR (176 MHz, $CDCl_3$):** δ 167.7, 149.9, 144.7, 132.3, 130.7, 121.3, 111.2, 44.4, 34.5, 31.3, 28.9, 24.9, 24.1, 23.9 ppm. Minor isomer: ^{13}C NMR could not be clearly identified. **IR (neat) ν_{max}:** 2959, 2928, 2868, 1640, 1611, 1460, 1408, 1384, 1363, 756. **HRMS (ESI$^+$):** exact mass calculated for $[M+Na]^+$ ($C_{20}H_{29}NNaO^+$) requires m/z 322.2141, found m/z 322.2139.

Anthracen-9-yl(2,3-dihydro-1H-pyrrol-1-yl)methanone (4b)

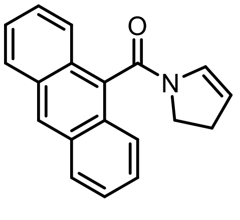

Following GP3 yielded the title compound (47.9 mg, 58%) as pale yellow solid. The desired enamide was obtained as two rotamers in a ratio of 5.3:1. Major isomer: **^{1}H NMR (600 MHz, $CDCl_3$)**: δ 8.50 (s, 1H), 8.03 (d, *J* = 7.7 Hz, 2H), 7.90 (d, *J* = 8.2 Hz, 2H), 7.56 – 7.47 (m, 4H), 5.63 (dt, *J* = 4.3, 2.2 Hz, 1H), 5.05 (dt, *J* = 4.9, 2.6 Hz, 1H), 4.40 – 4.31 (m, 2H), 2.87 – 2.80 (m, 2H) ppm. Minor isomer: **^{1}H NMR (600 MHz, $CDCl_3$):** δ 8.50 (s, 1H), 8.03 (d, *J* = 7.7 Hz, 2H), 7.97 (d, *J* = 8.4 Hz, 2H), 7.56 – 7.47 (m, 4H), 7.44 (dt, *J* = 4.3, 2.2 Hz, 1H), 5.46 (dt, *J* = 4.9, 2.6 Hz, 1H), 3.33 – 3.26 (m, 2H), 2.66 – 2.56 (m, 2H) ppm. **^{13}C NMR (151 MHz, $CDCl_3$)**: δ 166.2, 165.8, 131.4, 131.3, 130.7, 130.2, 129.1, 128.9, 128.8, 128.4, 128.2, 128.1, 127.5, 127.2, 127.1, 125.7, 125.2, 124.9, 113.0, 112.6, 46.4, 45.0, 30.0 (2C) ppm. Minor isomer: ^{13}C NMR could not be clearly identified. **IR (neat) ν_{max}:** 1632, 1611, 1435, 1400, 1328, 1276, 749. **HRMS (ESI$^+$):** exact mass calculated for $[M+Na]^+$ ($C_{19}H_{15}NNaO^+$) requires m/z 296.1046, found m/z 296.1038.

(2,3-Dihydro-1H-pyrrol-1-yl)(phenyl)methanone (4c)

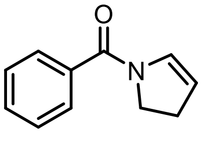

Following GP3 yielded the title compound (28.8 mg, 55%) as orange oil. The desired enamide was obtained as two rotamers in a ratio of 6.4:1. Major isomer: **^{1}H NMR (400 MHz, $CDCl_3$):** δ 7.55 – 7.38 (m, 5H), 6.47– 6.44 (m, 1H), 5.18 (app s, 1H), 4.03 (t, *J* = 8.8 Hz, 2H), 2.76 – 2.66 (m, 2H) ppm. Minor isomer: **^{1}H NMR (400 MHz, $CDCl_3$):** δ 7.55 – 7.38 (m, 5H), 7.10 (app s, 1H), 5.37 (app s, 1H), 3.86– 3.76 (m, 2H), 2.76 – 2.66 (m, 2H) ppm. Major isomer: **^{13}C NMR (176 MHz, $CDCl_3$):** δ 167.1, 136.0, 130.8, 130.4, 128.6, 127.8, 111.8, 45.8, 28.5 ppm. Minor isomer: ^{13}C NMR could not be clearly identified. **IR (neat) ν_{max}:** 2959, 2925, 1576, 1446,

1367, 830, 700. **HRMS (ESI$^+$):** exact mass calculated for $[M+H]^+$ ($C_{11}H_{12}NO^+$) requires 174.0919 m/z, found 174.0912 m/z.

(3,4-Dihydropyridin-1(2H)-yl)(phenyl)methanone (4d)

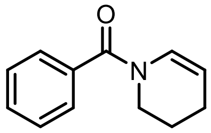

Following GP3 yielded the title compound (13.5 mg, 24%) as colorless oil. The desired enamide was obtained as two rotamers in a ratio of 3.4:1. Major isomer: **^{1}H NMR (600 MHz, CDCl$_3$):** δ 7.49 – 7.37 (m, 5H), 6.45 (d, *J* = 8.1 Hz, 1H), 4.90 – 4.80 (m, 1H), 3.89 – 3.78 (m, 2H), 2.12 (tdd, *J* = 6.1, 3.9, 2.0 Hz, 2H), 1.98 – 1.92 (m, 2H) ppm. Minor isomer: **^{1}H NMR (600 MHz, CDCl$_3$):** δ 7.49 – 7.37 (m, 5H), 7.35 – 7.27 (m, 1H), 5.29 (app s, 1H), 3.54 (app s, 1H), 2.12 (tdd, J = 6.1, 3.9, 2.0 Hz, 2H), 1.79 (br, 2H) ppm. Major isomer: **^{13}C NMR (151 MHz, CDCl$_3$):** δ 169.5, 135.3, 130.3, 128.5, 128.3, 127.7, 107.7, 41.23, 22.03, 21.81 ppm. Minor isomer: ^{13}C NMR could not be clearly identified. All spectral data were found in accordance to the literature.[17]

(3,4-Dihydropyridin-1(2H)-yl)(o-tolyl)methanone (4e)

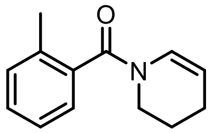

Following GP3 yielded the title compound (38.9 mg, 64%) as yellow oil. The desired enamide was obtained as two rotamers in a ratio of 3.4:1. Major isomer: **^{1}H NMR (400 MHz, CDCl$_3$):** δ 7.28 (m, 1H), 7.24 – 7.14 (m, 3H), 6.18 – 6.10 (m, 1H), 4.90 – 4.81 (m, 1H), 3.90 (app s, 2H), 2.29 (s, 3H), 2.14 – 2.07 (m, 2H), 1.91 – 1.80 (m, 2H) ppm. Minor isomer: **^{1}H NMR (400 MHz, CDCl$_3$):** δ 7.38 (d, *J* = 8.3 Hz, 1H), 7.35 – 7.25 (m, 1H), 7.24 – 7.14 (m, 3H), 5.28 – 5.20 (m, 1H), 3.32 – 3.28 (m, 2H), 2.31 (s, 3H), 2.14 – 2.07 (m, 2H), 1.78 (s, 2H) ppm. Major isomer: **^{13}C NMR (101 MHz, CDCl$_3$):** δ 169.3, 135.6, 134.6, 130.3, 129.2, 126.6, 126.4, 125.9, 108.1, 40.4, 21.9, 21.5, 19.0 ppm. Minor isomer: **^{13}C NMR (101 MHz, CDCl$_3$):** δ 168.5, 135.9, 134.3, 130.3, 129.0, 125.9, 125.8, 124.0, 110.5, 45.3, 22.3, 22.1, 18.9 ppm. **IR (neat) ν_{max}:** 2926, 1630, 1373, 1355, 1321, 1257, 992, 739, 730, 718, 621. **HRMS (ESI$^+$):** exact mass calculated for $[M+H]^+$ ($C_{13}H_{16}NO^+$) requires 202.1232 m/z, found 202.1226 m/z.

(3,4-Dihydropyridin-1(2H)-yl)(p-tolyl)methanone (4f)

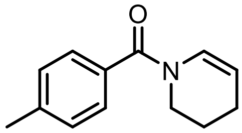

Following GP3 yielded the title compound (20.9 mg, 35%) as colorless oil. The desired enamide was obtained as two rotamers in a ratio of 3.7:1. Major isomer: **^{1}H NMR (600 MHz, CDCl$_3$):** δ 7.38 (d, *J* = 7.7 Hz, 2H), 7.21 (d, *J* = 7.9 Hz, 2H), 6.48 (d, *J* = 7.2 Hz, 1H), 4.83 (app s, 1H), 3.82 (app s, 2H), 2.38 (s, 2H), 2.15 – 2.09 (m, 2H), 1.95 (app s, 2H) ppm. Minor isomer: **^{1}H NMR (600 MHz, CDCl$_3$):** δ 7.38 (d, *J* = 7.7 Hz, 2H), 7.21 (d, *J* = 7.9 Hz, 2H), 5.21 (s, 1H), 3.58 (s, 2H), 2.38 (s, 3H), 2.15 – 2.09 (m, 3H), 1.79 (s, 2H) ppm. One peak was not detected, due to an overlapping with another signal. Major isomer: **^{13}C NMR (151 MHz, CDCl$_3$):** δ 169.6, 140.6, 132.3, 129.1, 128.5, 127.9, 107.3, 41.3, 22.1, 21.9, 21.6 ppm. Minor isomer: ^{13}C NMR could not be clearly identified. **IR (neat) ν_{max}:** 2925, 1632, 1407, 1375, 1356, 1291, 1258, 994, 753, 579. **HRMS (ESI$^+$):** exact mass calculated for $[M+H]^+$ ($C_{13}H_{16}NO^+$) requires 202.1232 m/z, found 202.1225 m/z.

(3,4-Dihydropyridin-1(2H)-yl)(2-methoxyphenyl)methanone (4g)

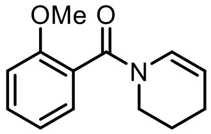

Following GP3 yielded the title compound (29.9 mg, 46%) as yellowish oil. The desired enamide was obtained as two rotamers in a ratio of 2.3:1. Major isomer: **^{1}H NMR (600 MHz, CDCl$_3$):** δ 7.39 – 7.35 (m, 1H), 7.22 (dd, *J* = 7.4, 1.7 Hz, 1H), 7.00 – 6.96 (m, 1H), 6.92 (d, *J* = 8.3 Hz, 1H), 6.19 (dt, *J* = 8.4, 1.9 Hz, 1H), 4.79 (dt, *J* = 8.1, 3.9 Hz, 1H), 3.90 – 3.85 (m, 2H), 3.81 (s, 3H), 2.14 – 2.05 (m, 2H), 1.95 – 1.87 (m, 2H) ppm. Minor isomer: **^{1}H NMR (600 MHz, CDCl$_3$):** δ 7.38 – 7.33 (m, 2H), 7.28 – 7.26 (m, 1H), 7.02 – 6.98 (m, 1H), 6.91

(d, *J* = 8.3 Hz, 1H), 5.20 (dt, *J* = 8.1, 3.9 Hz, 1H), 3.83 (s, 3H), 3.44 – 3.29 (m, 2H), 2.14 – 2.05 (m, 2H), 1.82 – 1.73 (m, 2H) ppm. Major isomer: **^{13}C NMR (151 MHz, CDCl$_3$):** δ 167.5, 155.8, 130.9, 128.5, 127.1, 125.3, 120.9, 111.3, 107.5, 55.8, 40.7, 22.1, 21.6 ppm. Minor isomer: **^{13}C NMR (151 MHz, CDCl$_3$):** δ 166.8, 155.7, 130.8, 128.4, 125.8, 125.3, 121.1, 111.0, 110.2, 55.7, 45.1, 22.3, 22.1 ppm. **IR (neat) ν_{max}:** 2930, 2840, 1629, 1599, 1370, 1356, 1288, 1245, 1109, 992, 751, 716, 618. **HRMS (ESI$^+$):** exact mass calculated for $[M+H]^+$ ($C_{13}H_{16}NO_2^+$) requires 218.1181 m/z, found 218.1172 m/z.

(3,4-Dihydropyridin-1(2H)-yl)(3-methoxyphenyl)methanone (4h)

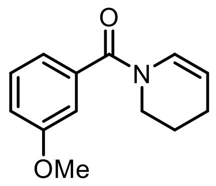

Following GP3 yielded the title compound (38.5 mg, 59%) as yellowish oil. The desired enamide was obtained as two rotamers in a ratio of 3.3:1. Major isomer: **^{1}H NMR (600 MHz, CDCl$_3$):** δ 7.31 (t, *J* = 7.9 Hz, 1H), 7.03 (d, *J* = 7.4 Hz, 1H), 7.00 (s, 1H), 6.98 – 6.94 (m, 1H), 6.45 (d, *J* = 8.1 Hz, 1H), 4.90 – 4.78 (m, 1H), 3.83 (d, *J* = 8.9 Hz, 5H), 2.17 – 2.06 (m, 2H), 1.97 – 1.88 (m, 2H). Minor isomer: **^{1}H NMR (600 MHz, CDCl$_3$):** δ 7.31 (t, *J* = 7.9 Hz, 1H), 7.27 (br, 1H), 7.03 (d, *J* = 7.4 Hz, 1H), 7.00 (s, 1H), 6.98 – 6.94 (m, 1H), 5.23 (br, 1H), 3.82 (s, 3H), 3.55 (br, 2H), 2.17 – 2.06 (m, 2H), 1.79 (br, 2H). Major isomer: **^{13}C NMR (151 MHz, CDCl$_3$):** δ 169.21, 159.7, 136.6, 129.5, 127.6, 120.5, 116.3, 113.4, 107.7, 55.5, 41.2, 22.0, 21.8. Minor isomer: ^{13}C NMR could not be clearly identified. **IR (neat) ν_{max}:** 2929, 2861, 1631, 1579, 1406, 1372, 1356, 1287, 1263, 1041, 994, 790, 746, 720, 624. **HRMS (ESI$^+$):** exact mass calculated for $[M+H]^+$ ($C_{13}H_{16}NO_2^+$) requires 218.1181 m/z, found 218.1173 m/z.

(3,4-Dihydropyridin-1(2H)-yl)(4-methoxyphenyl)methanone (4i)

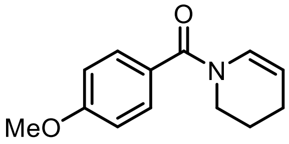

Following GP3 yielded the title compound (42.5 mg, 65%) as yellowish oil. No rotameric effects were observed *via* NMR spectroscopy. **^{1}H NMR (400 MHz, CDCl$_3$)** δ 7.47 (d, *J* = 8.7 Hz, 2H), 6.91 (d, *J* = 8.7 Hz, 2H), 6.52 (app s, 1H), 4.84 (app s, 1H), 3.86 – 3.74 (m, 5H), 2.16 – 2.05 (m, 2H), 1.94 (s, 2H). **^{13}C NMR (151 MHz, CDCl$_3$)**: δ 169.2, 161.3, 130.5, 128.1, 127.4, 113.7, 107.2, 55.5, 41.4, 22.1, 22.0. **IR (neat) ν_{max}:** 2931, 2840, 1714, 1625, 1605, 1508, 1355, 1321, 1248, 1172, 1027, 992, 839, 762, 748, 585. **HRMS (ESI$^+$):** exact mass calculated for $[M+H]^+$ ($C_{13}H_{16}NO_2^+$) requires 218.1181 m/z, found 218.1174 m/z.

(2,3-Dihydro-1H-pyrrol-1-yl)(3,4,5-trimethoxyphenyl)methanone (4j)

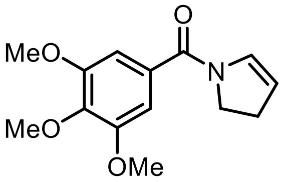

Following GP3 yielded the title compound (60.6 mg, 77%) as colorless oil. No rotameric effects were observed *via* NMR spectroscopy. **^{1}H NMR (400 MHz, CDCl$_3$):** δ 6.72 (s, 2H), 6.49 (app s, 1H), 5.19 (app s, 1H), 3.99 (t, *J* = 8.7 Hz, 2H), 3.85 – 3.84 (m, 9H). 2.76 – 2.63 (m, 2H) ppm. **^{13}C NMR (101 MHz, CDCl$_3$):** δ 166.7, 153.2, 139.8, 131.1, 130.7, 112.1, 105.1, 60.9, 56.3, 45.8, 28.4 ppm. **IR (neat) ν_{max}:** 2862, 2838, 1581, 1506, 1455, 1415, 1320, 1236, 1184, 1125, 1004, 806, 756, 718. **HRMS (ESI$^+$):** exact mass calculated for $[M+H]^+$ ($C_{14}H_{18}NO_4^+$) requires 264.1235 m/z, found 264.1231 m/z.

(3,4-Dihydropyridin-1(2H)-yl)(3,4,5-trimethoxyphenyl)methanone (4k)

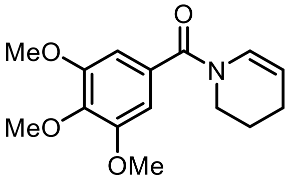

Following GP3 yielded the title compound (41.9 mg, 50%) as yellow oil. In addition remaining starting material (30.1 mg, 37 %) was recovered. The desired enamide was obtained as two rotamers in a ratio of around 4:1. Major isomer: **^{1}H NMR (600 MHz, CDCl$_3$):** δ 6.68 (s, 2H), 6.53 – 6.47 (m, 1H), 4.91 – 4.80 (m, 1H), 3.86 – 3.85 (m, 9H), 3.82 – 3.78 (m, 2H), 2.13 – 2.09 (m, 2H), 1.97 – 1.91 (m, 2H) ppm. Minor isomer: **^{1}H NMR (600 MHz, CDCl$_3$):** δ 7.23 (app s, 1H), 6.68 (s, 2H), 5.28 – 5.15 (m, 1H), 3.86 – 3.85 (m, 9H), 3.64 – 3.53 (m, 2H), 2.11 (m, 2H), 1.85 – 1.77 (m, 2H) ppm. Major isomer: **^{13}C NMR (151 MHz, CDCl$_3$):** δ 169.1, 153.2, 139.7, 130.5, 127.6, 107.7, 105.7, 61.0, 56.4, 41.3, 22.0, 21.7 ppm. Minor isomer: ^{13}C NMR could not be clearly identified. **IR (neat) ν_{max}:** 1581, 1542,

1409, 1374, 1356, 1228, 1121, 1002, 761, 735. **HRMS (ESI$^+$):** exact mass calculated for $[M+H]^+$ ($C_{15}H_{20}NO_4^+$) requires 278.1392 m/z, found 278.1388 m/z.

Benzo[d][1,3]dioxol-5-yl(3,4-dihydropyridin-1(2H)-yl)methanone (4l)

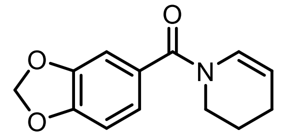

Following GP3 yielded the title compound (41.5 mg, 60%) as colorless oil and in addition remaining starting material (4.0 mg, 6 %) was recovered. The desired enamide was obtained as two rotamers in a ratio of around 3.6:1. Major isomer: **^{1}H NMR (600 MHz, CDCl$_3$):** δ 7.02 (d, *J* = 7.8 Hz, 1H), 6.98 (s, 1H), 6.82 (d, *J* = 8.0 Hz, 1H), 6.51 (app s, 1H), 6.00 (s, 2H), 4.86 (app s, 1H), 3.78 (app s, 2H), 2.12 (tdd, *J* = 6.1, 3.8, 2.0 Hz, 2H), 1.94 (app s, 2H) ppm. Minor isomer: **^{1}H NMR (600 MHz, CDCl$_3$):** δ 7.22 (app s, 1H), 7.02 (d, *J* = 7.8 Hz, 1H), 6.98 (s, 1H), 6.82 (d, *J* = 8.0 Hz, 1H), 6.00 (s, 2H), 5.21 (s, 1H), 3.63 (app s, 2H), 2.12 (tdd, *J* = 6.1, 3.8, 2.0 Hz, 2H), 1.85 (app s, 2H) ppm. Major isomer: **^{13}C NMR (151 MHz, CDCl$_3$):** δ 168.7, 149.4, 147.6, 128.9, 127.9, 123.3, 109.2, 108.1, 107.5, 101.6, 41.5, 22.1, 21.9 ppm. Minor isomer: ^{13}C NMR could not be clearly identified. **IR (neat) ν_{max}:** 2895, 2843, 1633, 1503, 1406, 1375, 1321, 1254, 1244, 1037, 996, 757. **HRMS (ESI$^+$):** exact mass calculated for $[M+H]^+$ ($C_{13}H_{14}NO_3^+$) requires 232.0974 m/z, found 232.0968 m/z.

(2-Bromo-5-methoxyphenyl)(2,3-dihydro-1H-pyrrol-1-yl)methanone (4m)

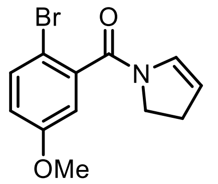

Following GP3 yielded the title compound (39.7 mg, 47%) as orange oil. The desired enamide was obtained as two rotamers in a ratio of 4:1. Major isomer: **^{1}H NMR (400 MHz, CDCl$_3$)**: δ 7.44 (d, *J* = 8.7 Hz, 1H), 6.88 – 6.78 (m, 2H), 6.01 (dt, *J* = 4.3, 2.2 Hz, 1H), 5.25 – 5.20 (m, 1H), 4.06 – 4.00 (m, 2H), 3.78 (s, 3H), 2.77 – 2.66 (m, 2H) ppm. Minor isomer: **^{1}H NMR (400 MHz, CDCl$_3$)**: δ 7.45 (d, *J* = 8.6 Hz, 1H), 7.06 (dt, *J* = 4.3, 2.2 Hz, 1H), 6.88 – 6.78 (m, 2H), 5.43 – 5.38 (m, 1H), 3.78 (s, 3H), 3.59 (t, *J* = 8.6 Hz, 2H), 2.77 – 2.66 (m, 2H) ppm. Major isomer: **^{13}C NMR (151 MHz, CDCl$_3$):** δ 164.7, 159.2, 138.7, 133.9, 129.8, 117.4, 113.6, 113.0, 109.7, 55.8, 45.0, 28.8 ppm. Minor isomer: **^{13}C NMR (151 MHz, CDCl$_3$):** δ 164.3, 159.4, 139.4, 133.9, 128.8, 117.0, 113.3, 112.9, 109.0, 55.8, 46.8, 29.9 ppm. **IR (neat) ν_{max}:** 2961, 2942, 1634, 1618, 1450, 1424, 1236, 1177, 1161, 808, 733, 599. **HRMS (ESI$^+$):** exact mass calculated for $[M+H]^+$ ($C_{12}H_{13}BrNO_2^+$) requires 282.0130 m/z, found 282.0123 m/z.

(2,3-Dihydro-1H-pyrrol-1-yl)(4-fluorophenyl)methanone (4n)

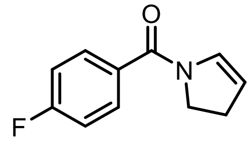

Following GP3 yielded the title compound as colorless oil (37.8 mg, 66%). The desired enamide was obtained as two rotamers in a ratio of around 6.4:1. Major isomer: **^{1}H NMR (600 MHz, CDCl$_3$):** δ 7.57 – 7.50 (m, 2H), 7.11 – 7.07 (m, 2H), 6.46 – 6.38 (m, 1H), 5.25 – 5.17 (m, 1H), 4.01 (t, *J* = 8.8 Hz, 2H), 2.73 – 2.68 (m, 2H). Minor isomer: **^{1}H NMR (600 MHz, CDCl$_3$):** δ 7.57 – 7.50 (m, 2H), 7.11 – 7.07 (m, 3H), 5.37 (app s, 1H), 3.81 (app s, 2H), 2.73 – 2.68 (m, 2H). Major isomer: **^{13}C NMR (151 MHz, CDCl$_3$):** δ 166.0, 163.9 (d, *J* = 250.8 Hz), 132.0, 130.6, 130.2 (d, *J* = 8.5 Hz), 115.6 (d, *J* = 21.8 Hz), 112.2, 45.9, 28.5 ppm. **^{19}F NMR (376 MHz, CDCl$_3$):** δ -109.3. **IR (neat) ν_{max}:** 1630, 1602, 1509, 1416, 1367, 1223, 1157, 1096, 1000, 845, 805, 756, 708, 614. **HRMS (ESI$^+$):** exact mass calculated for $[M+Na]^+$ ($C_{11}H_{10}FNONa^+$) requires 214.0644 m/z found 214.0637m/z.

(3-Chlorophenyl)(2,3-dihydro-1H-pyrrol-1-yl)methanone (4o)

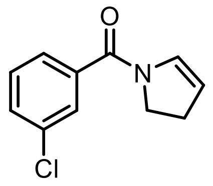

Following GP3 yielded the title compound as colorless oil (29.8 mg, 48%). The desired enamide was obtained as two rotamers in a ratio of around 6.2:1. Major isomer**: ^{1}H NMR (600 MHz, CDCl$_3$)**: δ 7.53 – 7.49 (m, 1H), 7.44 – 7.31 (m, 3H), 6.41 (dt, *J* = 4.2, 2.1 Hz, 1H), 5.25 – 5.20 (m, 1H), 4.05 – 3.97 (m, 2H), 2.75 – 2.68 (m, 2H) ppm. Minor isomer: **^{1}H NMR (600 MHz, CDCl$_3$):** δ 7.53 – 7.49 (m, 1H), 7.44 – 7.31 (m, 3H), 7.07 (app s, 1H), 5.40 (app s, 1H), 3.80 (t, *J* = 8.2 Hz, 2H), 2.75 – 2.68 (m,

2H) ppm. Major isomer: **^{13}C NMR (151 MHz, CDCl$_3$):** δ 165.4, 137.6, 134.7, 130.6, 130.3, 130.0, 128.0, 125.9, 112.6, 45.8, 28.5. Minor isomer: ^{13}C NMR could not be clearly identified. **IR (neat) ν_{max}:** 1632, 1611, 1567, 1422, 1402, 1367, 739, 719. **HRMS (ESI$^+$):** exact mass calculated for [M+Na]$^+$ ($C_{11}H_{10}ClNONa^+$) requires 230.0348 m/z found 230.0343 m/z.

(2,3-Dihydro-1H-pyrrol-1-yl)(4-vinylphenyl)methanone (4p)

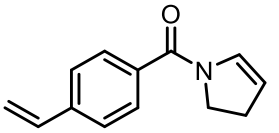

Following GP3 yielded the title compound (16.4 mg, 27%) as yellow oil. In addition, remaining starting material (14.2 mg, 24%) was possible to be recovered. The desired enamide was obtained as two rotamers in a ratio of around 5:1. Major isomer: **^{1}H NMR (400 MHz, CDCl$_3$):** δ 7.49 (d, *J* = 8.1 Hz, 2H), 7.44 (d, *J* = 8.2 Hz, 2H), 6.73 (dd, *J* = 17.6, 10.9 Hz, 1H), 6.47 (app s, 1H), 5.81 (d, *J* = 17.6 Hz, 1H), 5.33 (d, *J* = 10.9 Hz, 1H), 5.19 (app s, 1H), 4.02 (t, *J* = 8.8 Hz, 2H), 2.76 – 2.66 (m, 2H) ppm. Minor isomer: **^{1}H NMR (400 MHz, CDCl$_3$):** δ 7.49 (d, *J* = 8.1 Hz, 2H), 7.44 (d, *J* = 8.2 Hz, 2H), 7.09 (app s, 1H), 6.73 (dd, *J* = 17.6, 10.9 Hz, 1H), 5.81 (d, *J* = 17.6 Hz, 1H), 5.36 (app s, 1H), 5.33 (d, *J* = 10.9 Hz, 1H), 3.91 – 3.79 (m, 2H), 2.87 – 2.60 (m, 2H) ppm. Major isomer: **^{13}C NMR (101 MHz, CDCl$_3$)**: δ 166.8, 139.7, 136.2, 135.1, 130.8, 128.2, 126.3, 115.7, 111.9, 45.8, 28.5 ppm. Minor isomer: ^{13}C NMR could not be clearly identified. **IR (neat) ν_{max}:** 2953, 2923, 2860, 1629, 1411, 1367, 998, 917, 853, 834, 784, 772, 721. **HRMS (ESI$^+$):** exact mass calculated for [M+H]$^+$ ($C_{13}H_{14}NO^+$) requires 200.1075 m/z, found 200.1071 m/z.

(2,3-Dihydro-1H-pyrrol-1-yl)(4-(methylthio)phenyl)methanone (4q)

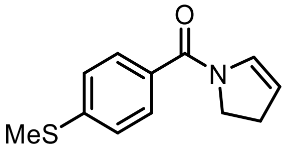

Following GP3 yielded the title compound (18.0 mg, 27%) as orange oil. No rotameric effects were observed *via* NMR spectroscopy. **^{1}H NMR (400 MHz, CDCl$_3$):** δ 7.44 (d, *J* = 7.9 Hz, 2H), 7.25 (d, *J* = 8.1 Hz, 2H), 6.47 (app s, 1H), 5.19 (app s, 1H), 4.00 (t, *J* = 8.7 Hz, 2H), 2.73 – 2.67 (m, 2H), 2.50 (s, 3H). **^{13}C NMR (101 MHz, CDCl$_3$):** δ 166.6, 142.1, 132.2, 130.8, 128.4, 125.7, 111.9, 45.9, 28.5, 15.3 ppm. **IR (neat) ν_{max}:** 2953, 2858, 1410, 1367, 1186, 1090, 831, 751, 712. **HRMS (ESI$^+$):** exact mass calculated for [M+H]$^+$ ($C_{12}H_{14}NOS^+$) requires 220.0796 m/z, found 220.0789 m/z.

3-(2,3-Dihydro-1H-pyrrole-1-carbonyl)benzonitrile (4r)

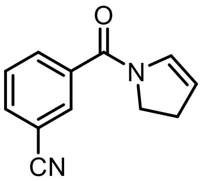

Following GP3 yielded the title compound (34.0 mg, 57%) as yellow solid. In addition, remaining starting material (26.0 mg, 43%) was possible to be recovered. The desired enamide was obtained as two rotamers in a ratio of around 6:1. Major isomer: **^{1}H NMR (600 MHz, CDCl$_3$)**: δ 7.79 (s, 1H), 7.75 – 7.70 (m, 2H), 7.55 (t, *J* = 7.8 Hz, 1H), 6.34 (dt, *J* = 4.1, 2.0 Hz, 1), 5.31 – 5.27 (m, 1H), 4.04 – 4.00 (m, 2H), 2.75 – 2.71 (m, 2H). Minor isomer: **^{1}H NMR (600 MHz, CDCl$_3$):** δ 7.79 (s, 1H), 7.75 – 7.70 (m, 2H), 7.55 (t, *J* = 7.8 Hz, 1H), 7.05 (app s, 1H), 5.44 (app s, 1H), 3.79 (t, *J* = 8.3 Hz, 2H), 2.75 – 2.71 (m, 2H). Major isomer: **^{13}C NMR (151 MHz, CDCl$_3$):** δ 164.4, 137.2, 133.8, 132.1, 131.4, 129.7, 129.6, 118.0, 113.5, 113.0, 46.0, 28.5. Minor isomer: ^{13}C NMR could not be clearly identified. **IR (neat) ν_{max}:** 2923, 2897, 2861, 1632, 1612, 1433, 1403, 1367, 1198, 1181, 804, 737, 690. **HRMS (ESI$^+$):** exact mass calculated for [M+H]$^+$ ($C_{12}H_{11}N_2O^+$) requires 199.0871 m/z, found 199.0866 m/z.

(2,3-Dihydro-1H-pyrrol-1-yl)(ferrocene)methanone (4u)

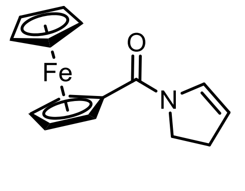

Following GP3 using as a cosolvent DCM (0.9 mL) yielded the title compound (45.3 mg, 54%) as a red solid. **^{1}H NMR (400 MHz, CDCl$_3$):** δ 7.12 (dt, *J* = 4.2, 2.1 Hz, 1H), 5.24 (app s, 1H), 4.79 – 4.60 (m, 2H), 4.35 (s, 2H), 4.23 (s, 5H), 4.09 – 3.93 (m, 2H), 2.81 – 2.59 (m, 2H) ppm. **^{13}C NMR (176 MHz, CDCl$_3$):** δ 167.0, 130.8, 111.0, 77.8, 70.3, 69.9, 69.8, 46.4, 28.0 ppm. **IR (neat) ν_{max}:** 1604, 1459, 1406,

1346, 809. **HRMS (ESI$^+$):** exact mass calculated for $[M+H]^+$ ($C_{15}H_{16}FeNO^+$) requires m/z 282.0576, found m/z 282.0576.

4-(2,3-Dihydro-1H-pyrrole-1-carbonyl)-2-methoxyphenyl 2-(3-cyano-4-isobutoxyphenyl)-4-methylthiazole-5-carboxylate (4v)

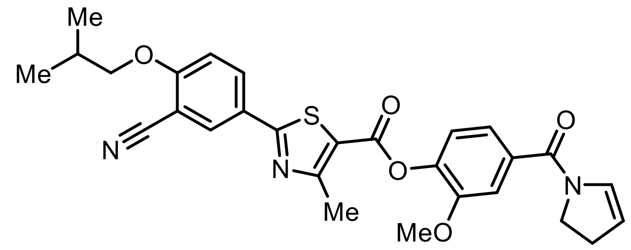

Following GP3 (0.24 mmol scale) using as a cosolvent DCM (1.1 mL) yielded the title compound (47.6 mg, 38%) as a yellow solid. In addition, remaining starting material (17.2 mg, 14%) was recovered. **^{1}H NMR (400 MHz, $CDCl_3$):** δ 8.23 (d, *J* = 2.2 Hz, 1H), 8.13 (dd, *J* = 8.8, 2.2 Hz, 1H), 7.24 – 7.18 (m, 2H), 7.12 (d, *J* = 8.0 Hz, 1H), 7.03 (d, *J* = 8.9 Hz, 1H), 6.54 (s, 1H), 5.23 (s, 1H), 4.03 (t, *J* = 8.8 Hz, 2H), 3.91 (d, *J* = 6.5 Hz, 2H), 3.87 (s, 3H), 2.82 (s, 3H), 2.77 – 2.70 (m, 2H), 2.26 – 2.17 (m, 1H), 1.10 (d, *J* = 6.7 Hz, 6H) ppm. **^{13}C NMR (101 MHz, $CDCl_3$):** δ 168.4, 166.1, 163.4, 162.8, 159.8, 151.6, 140.9, 135.0, 132.8, 132.4, 130.7, 126.0, 122.9, 120.3, 120.2, 115.5, 112.8, 112.5, 112.3, 103.2, 75.9, 56.3, 46.0, 28.5, 28.3, 19.2, 17.8 ppm. **IR (neat) ν_{max}:** 2960, 2929, 2873, 2229, 1733, 1605, 1507, 1423, 1371, 1329, 1273, 1250, 1199, 1182, 1121, 1049, 1011, 733. **HRMS (ESI$^+$):** exact mass calculated for $[M+H]^+$ ($C_{28}H_{28}N_3O_5S^+$) requires m/z 518.1744, found m/z 518.1751.

3.4. Unsuccessful and low-yielding Substrates

Table 3. Unsuccessful and poor yielding substrates.[a]

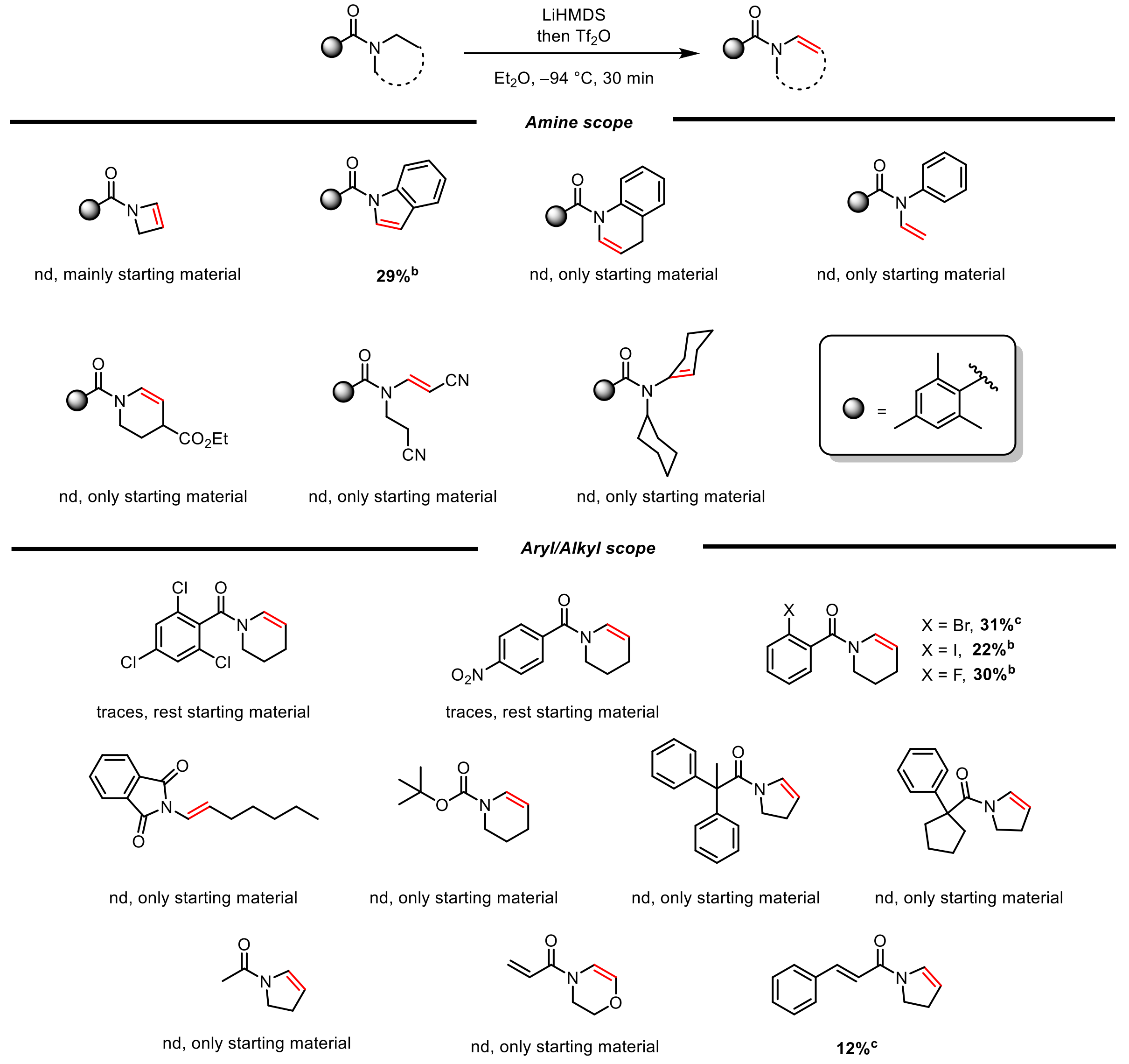

[a] amide (0.30 mmol), 1.5 mL Et_2O, LiHMDS (0.96 mmol, 1M solution in THF) with slow addition, Tf_2O (0.48 mmol) with addition over 1 min. [b] GC yield determined with decane (ratio 1:1) as internal standard. [c] Isolated yield.

3.5. Application of Enamides

Methyl 1-benzoyl-8-oxo-2,3,3a,4,7,7a-hexahydro-1H-4,7-(epoxymethano)indole-5-carboxylate (5a)

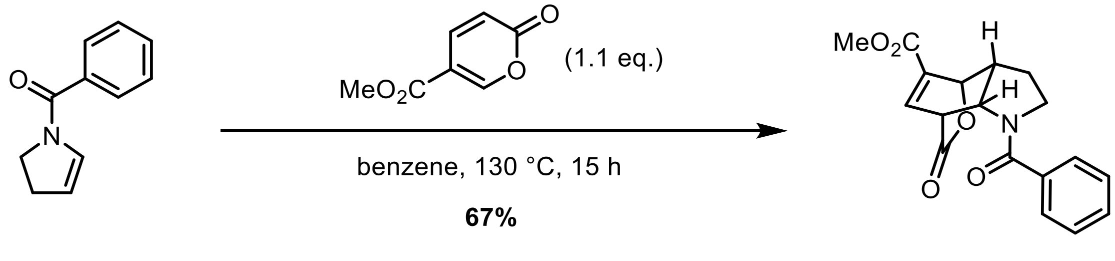

A procedure adopted from literature was used.[18] An oven-dried screw-cap vial was loaded with methyl coumalate (84.8 mg, 0.55 mmol, 1.10 eq.), followed by the addition of enamide **4c** (86.6 mg, 0.50 mmol, 1.00 eq.) dissolved in toluene (1 mL). The mixture was heated at reflux (130 °C, oil bath temperature) for 15 h, after which full conversion of the enamide was observed by TLC. The solvent was evaporated and the crude material was subjected to flash column chromatography (heptane/EtOAc, 8:2 – 2:8) to give the title compound (110 mg, 0.34 mmol, 67%) as white solid. **^{1}H NMR (400 MHz, CDCl$_3$):** δ 7.44 – 7.31 (m, 5H), 7.25 (d, *J* = 5.0 Hz, 1H), 5.56 (s, 1H), 4.74 (d, *J* = 6.2 Hz, 1H), 4.26 (s, 1H), 3.75 (s, 3H), 3.66 – 3.53 (m, 1H), 3.46 – 3.35 (m, 1H), 2.68 (t, *J* = 9.3 Hz, 1H), 2.20 – 2.00 (m, 2H) ppm. **^{13}C NMR (101 MHz, CDCl$_3$):** δ 167.6, 154.2, 134.7, 131.7, 131.5, 128.6, 127.8, 127.0, 123.1, 112.8, 112.6, 112.2, 100.5, 55.9, 40.5, 25.4 ppm. **IR (neat) ν_{max}:** 3106, 2941, 2872, 2342, 1644, 1524, 1446, 1345, 1168, 1088, 1077, 983, 856, 740, 680, 606, 558. **HRMS (ESI$^+$):** exact mass calculated for $[M+H]^+$ ($C_{18}H_{18}NO_5^+$) requires 328.1185 m/z, found 328.1176 m/z.

Ethyl 1-benzoyl-4-(4-chlorophenyl)-2,3-dihydro-1H-pyrrolo[3,2-c]quinoline-8-carboxylate (5b)

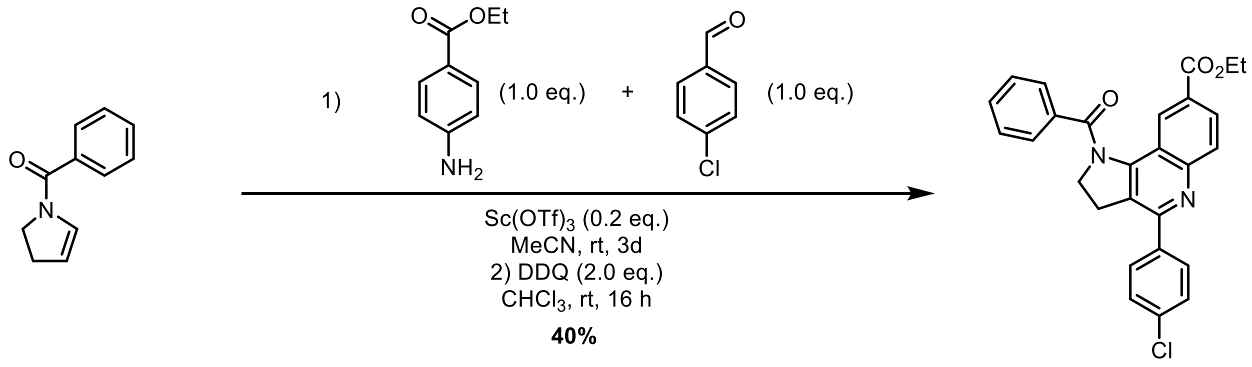

A modified procedure adopted from literature was used.[19] Ethyl 4-aminobenzoate (49.6 mg, 0.30 mmol, 1.00 eq.) and *p*-chlorobenzaldehyde (42.2 mg, 0.30 mmol, 1.00 eq.) were dissolved in anhydrous acetonitrile (0.5 mL) in an oven-dried round bottom flask. Then, molecular sieves (4 Å) were added, followed by scandium triflate (29.5 mg, 0.06 mmol, 0.20 eq.). The resulting mixture was stirred at room temperature for 5 min. Then, the enamide (52.0 mg, 0.30 mmol, 1.00 eq.) dissolved in acetonitrile (0.5 mL) was added. The mixture was stirred for 3 d at room temperature. Then, the reaction was stopped by the addition of aqueous saturated solution of $NaHCO_3$ (5 mL) and filtered. The aqueous phase was extracted with EtOAc (3x 10 mL). The combined organic phases were dried over anhydrous $MgSO_4$ and the solvent was removed under reduced pressure. The crude material was used directly in the next oxidative step and was dissolved in anhydrous $CHCl_3$ (0.5 mL). Next, 3-dichloro-5,6-dicyano-1,4-benzoquinone (136 mg, 0.60 mmol, 2.00 eq.) was added. The reaction was stirred for 16 h at room temperature. Then CH_2Cl_2 (5 mL) was added and the organic phase was washed with saturated aqueous $NaHCO_3$ (3x 5 mL), filtered over anhydrous $MgSO_4$ and concentrated under reduced pressure. The obtained material was purified by column chromatography (heptane/EtOAc, 1:9 – 1:1) yielding the title compound (55.5 mg, 0.121 mmol, 41%) as yellow solid.**^{1}H NMR (400 MHz, CDCl$_3$):** δ 8.54 (s, 1H), 8.22 (d, *J* = 8.9 Hz, 1H), 8.14 (d, *J* = 8.9 Hz, 1H), 7.81 (app t, 4H), 7.57 (t, *J* = 7.6 Hz, 1H), 7.48 (m, 4H),

4.36 (m, 4H), 3.35 (t, *J* = 7.6 Hz, 2H), 1.37 (t, *J* = 7.0 Hz, 3H) ppm. **^{13}C NMR (101 MHz, CDCl$_3$):** δ 171.1, 166.3, 156.1, 150.8, 150.3, 137.7, 135.6, 134.8, 132.3, 130.1, 130.0, 129.1, 129.0, 128.8, 128.8, 128.6, 127.4, 124.9, 118.5, 61.3, 54.9, 30.3, 14.5 ppm. **IR (neat) ν$_{max}$:** 3062, 2980, 2343, 1713, 1662, 1489, 1149, 1391, 1253, 1092, 1014, 843, 730, 706. **HRMS (ESI$^+$):** exact mass calculated for $[M+H]^+$ ($C_{27}H_{22}ClN_2O_3^+$) requires 457.1313 m/z, found m/z 457.1305.

Mesityl(3,4,4a,8b-tetrahydrobenzo[3,4]cyclobuta[1,2-b]pyridin-1(2H)-yl)methanone (5c)

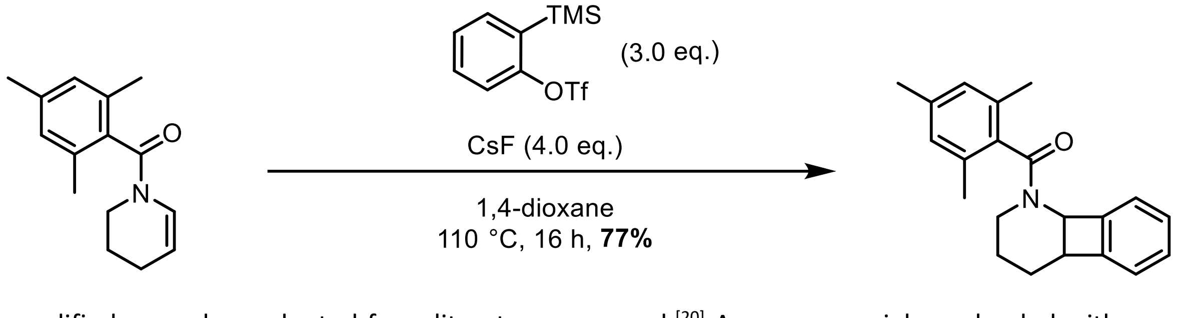

A modified procedure adopted from literature was used.[20] A screw-cap vial was loaded with oven-dried CsF (91.1 mg, 0.60 mmol, 4.00 eq.), enamide **2a** (34.3 mg, 0.15 mmol, 1.00 eq.) and anhydrous 1,4-dioxane (2 mL). Then, 2-(trimethylsilyl)phenyl trifluoro-methanesulfonate (115 µL, 0.45 mmol, 3.00 eq.) was added dropwise using a gastight syringe. The reaction mixture was heated at 110 °C for 16 h. After cooling to room temperature, the solution was filtered through a plug of Celite®, followed by three rinse cycles with EtOAc. The filtrate was concentrated *in vacuo* and subsequently purified via silica gel flash column chromatography (heptane/EtOAc, pure heptane to 6:4) giving rise to the desired amidobenzocyclobutane (35.3 mg, 0.116 mmol, 77%) as yellowish oil. The desired cyclobutane product was obtained as two isomers in a ratio of 1:1. **^{1}H NMR (600 MHz, CDCl$_3$):** δ 7.35 – 7.21 (m, 2.5H), 7.12 (dd, *J* = 7.1, 0.5 Hz, 0.5H), 7.09 – 7.06 (m, 1H), 6.88 – 6.83 (m, 2H), 5.98 (d, *J* = 5.5 Hz, 0.5H), 4.92 (d, *J* = 5.5 Hz, 0.5H), 4.28 – 4.19 (m, 0.5H), 4.15 – 4.11 (m, 0.5H), 3.95 – 3.92 (m, 0.5H), 3.30 (ddd, *J* = 13.8, 8.0, 1.7 Hz, 0.5H), 3.15 – 3.11 (m, 0.5H), 3.08 – 3.01 (m, 0.5H), 2.39 (s, 1.5H), 2.29 – 2.25 (m, 6H), 2.17 (s, 1.5H), 2.10 – 2.03 (m, 1H), 2.00 – 1.92 (m, 1H), 1.70 – 1.61 (m, 0.5H), 1.51 – 1.45 (m, 0.5H), 1.43 – 1.37 (m, 0.5H), 1.35 – 1.30 (m, 0.5H) ppm. **^{13}C NMR (151 MHz, CDCl$_3$):** δ 172.1, 171.6, 146.9, 146.3, 146.1, 145.1, 138.2, 138.0, 134.3, 134.2, 133.7, 133.5, 133.3, 133.2, 129.5, 129.0, 128.5, 128.4, 128.3, 127.9, 127.9, 123.8, 123.0, 122.9, 122.1, 56.5, 52.6, 45.2, 44.0, 43.1, 39.4, 25.7, 24.7, 19.9, 19.4, 18.9, 17.2 ppm. **IR (neat) ν$_{max}$:** 2919, 2876, 2855, 1629, 1611, 1436, 1404, 1361, 1308, 1263, 1169, 852, 756. **HRMS (ESI$^+$):** exact mass calculated for $[M+H]^+$ ($C_{21}H_{24}NO^+$) requires 306.1858 m/z, found 306.1855 m/z.

N-(2-(5-Methoxy-1H-indol-3-yl)ethyl)benzamide (5d)

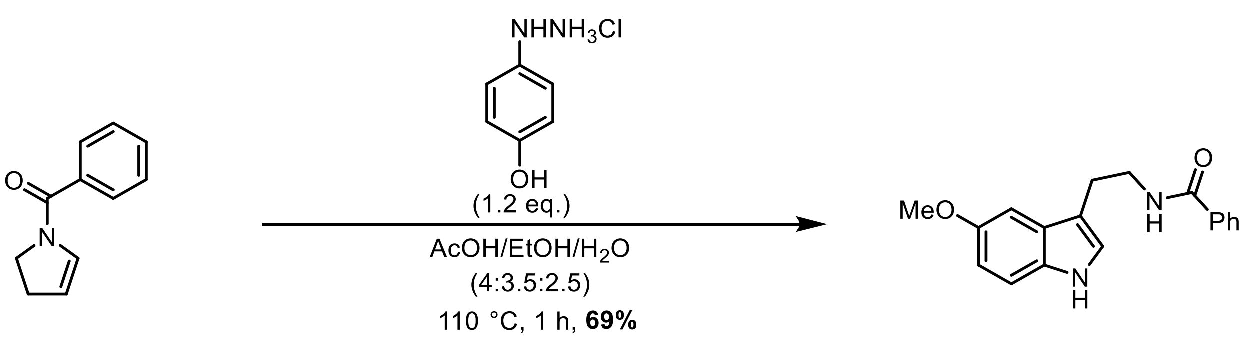

A modified procedure adopted from literature was used.[21] An oven-dried screw-cap vial was loaded with enamide **4c** (41.6 mg, 0.24 mmol, 1.00 eq.), (4-methoxyphenyl)hydrazine hydrochloride (50.6 mg, 0.29 mmol, 1.20 eq.) and a solvent mixture of AcOH/EtOH/H$_2$O (0.5 mL, 0.7 mL, 0.8 mL). The reaction mixture was heated at 110 °C for 1 h. After cooling to room temperature, CH_2Cl_2 (5 mL) was added and the organic phase was washed with saturated aqueous $NaHCO_3$ (5 mL) dried over anhydrous $MgSO_4$

and evaporated *in vacuo*. The crude material was subjected to flash column chromatography (heptane/EtOAc, 8:2 – 2:8) affording the title compound (49.1 mg, 0.167 mmol, 70%) as a red liquid. **^{1}H NMR (400 MHz, CDCl$_3$):** δ 8.21 (s, 1H), 7.59 (d, *J* = 7.5 Hz, 2H), 7.37 (t, *J* = 7.3 Hz, 1H), 7.28 (t, *J* = 7.5 Hz, 2H), 7.16 (d, *J* = 8.7 Hz, 1H), 6.94 (d, *J* = 11.7 Hz, 2H), 6.77 (dd, *J* = 8.7, 1.7 Hz, 1H), 6.26 (s, 1H), 3.71 – 3.67 (m, 5H), 2.96 (t, *J* = 6.6 Hz, 2H) ppm. **^{13}C NMR (101 MHz, CDCl$_3$):** δ 167.6, 154.2, 134.7, 131.7, 131.5, 128.6, 127.8 127.0, 123.1, 112.8, 112.6, 112.2, 100.5, 55.9, 40.5, 25.4 ppm. **IR (neat) ν_{max}:** 3406, 3296, 3058, 2933, 1635, 1577, 1529, 1484, 1453, 1439, 1293, 1214, 1173, 1072, 1030, 922, 797, 710. **HRMS (ESI$^+$):** exact mass calculated for [M+H]$^+$ ($C_{18}H_{19}N_2O_2^+$) requires 295.1447 m/z, found 295.1441 m/z.

N-(3,3-difluoropropyl)benzamide (5e)

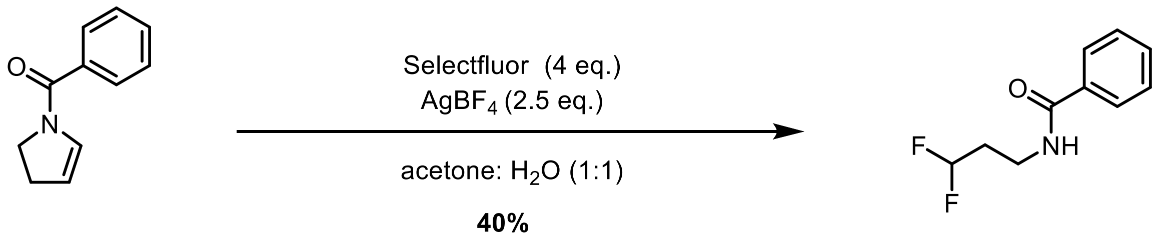

A modified procedure adopted from literature was used.[22] An oven-dried dram vial was loaded with enamide **4c** (26.3 mg, 0.15 mmol, 1.00 eq.) dissolved in acetone/H_2O (1:1, 0.5 mL). Selectfluor® (213 mg, 0.60 mmol, 4.00 eq.) was added followed by $AgBF_4$ (73 mg, 0.38 mmol, 2.5 eq). The reaction mixture was stirred at room temperature for 16 h. After this time, water (5 mL) was added and the aqueous phase was extracted with EtOAc (5x 10 mL). The combined organic layers were dried over anhydrous $MgSO_4$ and concentrated under reduced pressure. The crude material was purified via flash column chromatography (heptane/EtOAc, pure heptane to 6:4) to afford the title compound (12.0 mg, 0.06 mmol, 40%) as a colorless oil. **^{1}H NMR (600 MHz, CDCl$_3$):** δ 7.77 – 7.74 (m, 2H), 7.53 – 7.49 (m, 1H), 7.45 – 7.41 (m, 2H), 6.41 (s, 1H), 5.99 (tt, *J* = 56.1, 4.1 Hz, 1H), 3.70 – 3.65 (m, 2H), 2.26 – 2.14 (m, 2H) ppm. **^{13}C NMR (151 MHz, CDCl$_3$):** δ 167.8, 134.4, 131.8, 128.8, 127.0, 116.6 (t, *J* = 239.3 Hz), 34.1 (t, *J* = 20.6 Hz), 33.8 (t, *J* = 6.2 Hz) ppm. **^{19}F NMR (376 MHz, CDCl$_3$):** δ -116.30 ppm. **IR (neat) ν_{max}:** 3321, 3066, 2942, 1710, 1639, 1537, 1308, 1294, 1221, 1117, 1067, 966, 693, 670, 595. **HRMS (ESI$^+$):** exact mass calculated for [M+Na]$^+$ ($C_{10}H_{12}NONa^+$) requires 222.0706 m/z, found 222.0695 m/z.

(R)-8-Hydroxy-7,9-dimethoxy-1,2,3,9b-tetrahydro-5H-pyrrolo[2,1-a]isoindol-5-one (5f)

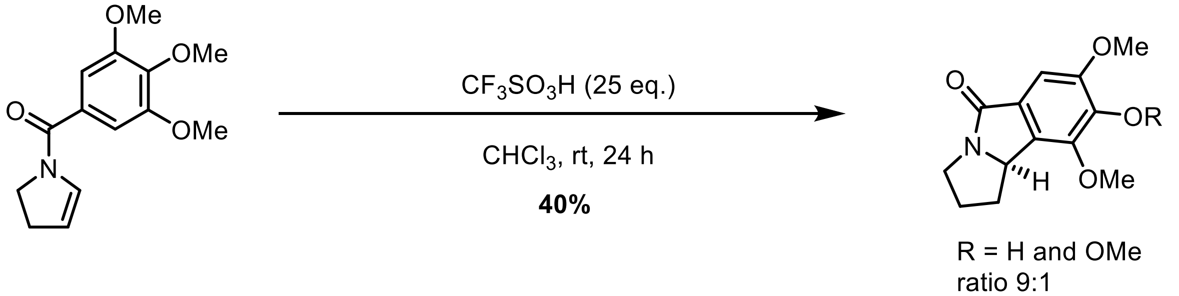

A modified procedure adopted from literature was used.[18] To a solution of the enamide (105 mg, 0.40 mmol, 1.00 eq.) dissolved in $CHCl_3$ (2 mL) trifluoromethanesulfonic acid (1.95 mL, 22.0 mmol, 25.0 eq.) was added slowly. The reaction was stirred for 16 h before it was poured into ice water (10 mL). The aqueous phase was extracted three times with $CHCl_3$ (5 mL), the organic phases were combined and dried over anhydrous $MgSO_4$. After concentration of the organic phase under reduced pressure, the crude material was subjected to flash column chromatography (heptane/EtOAc, 1:1 to pure EtOAc) affording the cyclized product (39.4 mg, 0.158 mmol, 40%) as yellow solid. The major product was that of demethylation of the para-methoxy group. **^{1}H NMR (600 MHz, CDCl$_3$):** δ 7.06 (s, 1H), 6.07 (s, 1H),

4.66 (dd, J = 10.6, 5.5 Hz, 1H), 3.98 (s, 3H), 3.92 (s, 3H), 3.69 – 3.59 (m, 1H), 3.41 – 3.35 (m, 1H), 2.39 – 2.25 (m, 3H), 1.25 – 1.16 (m, 1H) ppm. **^{13}C NMR (151 MHz, CDCl$_3$):** δ 172.2, 148.9, 141.9, 141.6, 132.3, 124.9, 101.2, 62.9, 60.6, 56.8, 42.0, 30.1, 29.4 ppm. **IR (neat) ν_{max}:** 3221, 2940, 2893, 2848, 1666, 1617, 1477, 1373, 1327, 1224, 1192, 1095, 1071, 1031. **HRMS (ESI$^+$):** exact mass calculated for $[M+H]^+$ ($C_{13}H_{16}NO_4^+$) requires 250.1079 m/z, found 250.1065 m/z.

Mesityl(5-phenyl-3,4-dihydropyridin-1(2H)-yl)methanone (5g)

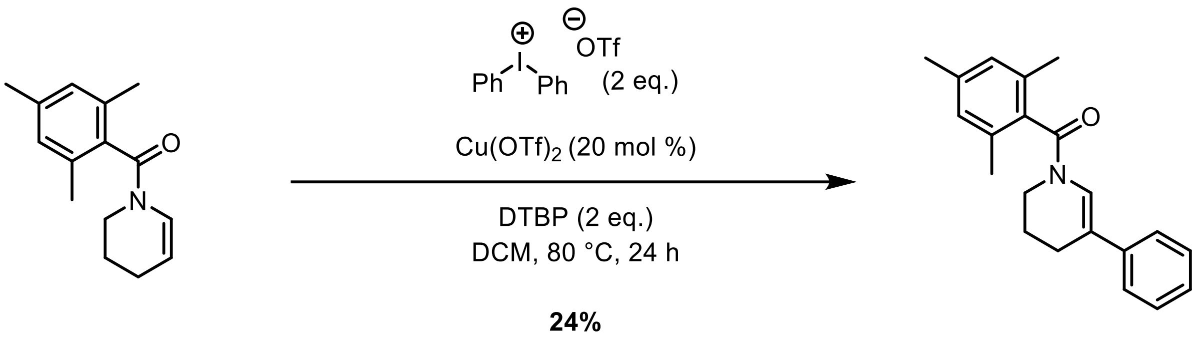

A modified procedure adopted from literature was used.[23] An oven-dried screw cap vial was loaded with diphenyliodonium trifuoromethanesulfonate (258 mg, 0.60 mmol, 2.00 eq.) and copper(II) trifluoromethanesulfonate (21.7 mg, 0.06 mmol, 0.20 eq.). Then, enamide **2a** (68.8 mg, 0.30 mmol, 1.00 eq.) dissolved in CH_2Cl_2 (0.5 mL) was added followed by the addition of 2,6-di-tert-butylpyridine (139 µL, 0.60 mmol, 2.00 eq.). The resulting mixture was heated at 80 °C for 24 h. Then, a saturated aqueous solution of $NaHCO_3$ (5 mL) was added. The aqueous phase was extracted with CH_2Cl_2 (2 x 5 mL) and the combined organic phases were washed with brine (5 mL). The organic phase was dried over anhydrous $MgSO_4$ and concentrated *in vacuo*. The crude product was purified by column chromatography (heptane/EtOAc, pure heptane to 8:2) giving rise to the title compound as colorless oil (21.6 mg, 0.07 mmol, 24%). The NMR analysis revealed the presence of two rotamers in a ratio of 1.5:1. **^{1}H NMR (400 MHz, CDCl$_3$)**: δ 7.98 (s, 0.4H), 7.54 – 7.45 (m, 1H), 7.34 (t, J = 7.6 Hz, 1H), 7.23 (d, 1H), 7.19 – 7.09 (m, 2H), 6.87 (s, 2H), 6.57 (s, 0.6H), 4.00 – 3.94 (m, 1.2H), 3.34 – 3.29 (m, 0.8H), 2.54 (t, J = 5.8 Hz, 2H), 2.30 (s, 3H), 2.23 – 2.19 (m, 6H), 2.09 – 2.03 (m, 1.2H), 1.97 – 1.92 (m, 0.8H). **^{13}C NMR (151 MHz, CDCl$_3$):** δ 170.1, 169.1, 139.9, 139.7, 138.7, 138.5, 134.1, 133.8, 133.5, 132.7, 128.6 (2C), 128.4, 126.9, 126.7, 124.84 (2C), 123.2, 121.3, 121.1, 119.7, 44.2 40.0, 25.1, 24.8, 22.7 (2C), 21.3 (2C), 19.2, 19.1. **IR (neat) ν_{max}:** 2922, 2855, 2362, 1628, 1611, 1455, 1386, 1312, 1261, 1170, 996, 851, 750, 727, 694. **HRMS (ESI$^+$):** exact mass calculated for $[M+H]^+$ ($C_{21}H_{24}NO^+$) requires 306.1858 m/z, found 306.1854 m/z.

4. Mechanistic Studies

4.1. Isotopic-labeling Experiments:

4.1.1. Preparation of Isotopically-labeled Starting Materials

Phenyl(pyrrolidin-1-yl)methanone-^{18}*O* (6a)

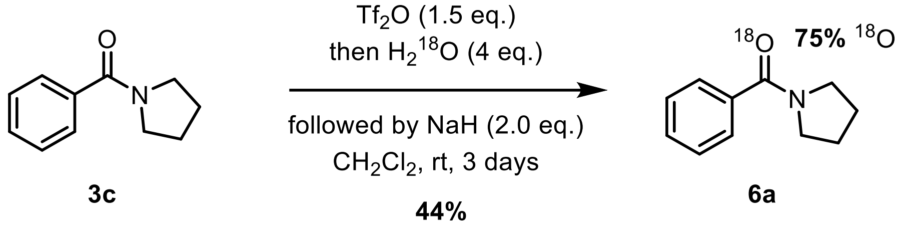

Amide (**3c**, 200 mg, 1.14 mmol, 1.00 eq) was dissolved in anhydrous CH_2Cl_2 (3.5 mL) in a flame-dried flask. The resulting solution was cooled to 0 °C and Tf_2O (288 µL, 1.71 mmol, 1.50 eq) was added. The reaction was stirred for 15 min at 0 °C. Then, ^{18}O-water (82 µL, 4.57 mmol, 4.00 eq) was added and the mixture was stirred for 24 h at room temperature. Since no conversion of the iminium triflate was observed, sodium hydride (60% in paraffine, 91.3 mg, 2.28 mmol, 2.00 eq) was added and the mixture was stirred vigorously for 3 d. The mixture was diluted with CH_2Cl_2 (30 mL), washed with NH_4Cl (saturated, 20 mL), dried over anhydrous $MgSO_4$ and concentrated under reduced pressure. The crude product was purified by column chromatography (heptane/EtOAc, 8:2 to pure EtOAc) to afford **6a** (89.9 mg, 44%) with an ^{18}O content of 75%, as determined by HRMS.

Acquisition Parameter

Source Type	ESI	Ion Polarity	Positive	Set Nebulizer	0.4 Bar
Focus	Not active	Set Capillary	4200 V	Set Dry Heater	180 °C
Scan Begin	50 m/z	Set End Plate Offset	-500 V	Set Dry Gas	4.0 l/min
Scan End	1900 m/z	Set Charging Voltage	0 V	Set Divert Valve	Source
		Set Corona	0 nA	Set APCI Heater	0 °C

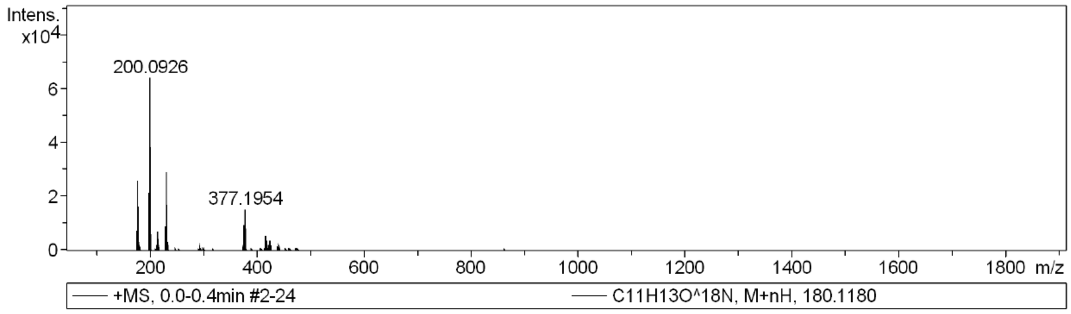

#	m/z	I
1	176.1062	7679
2	177.1098	1266
3	178.1107	26031
4	179.1140	3187
5	198.0883	21326
6	199.0917	3099
7	200.0926	63960

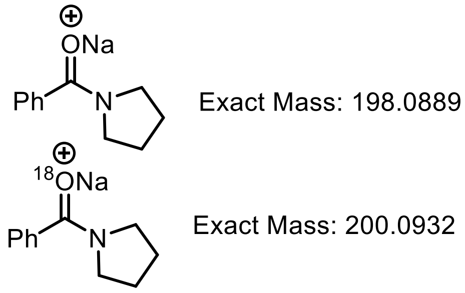

The (non-labeled) product is reported in the literature.[3]

Phenyl(pyrrolidin-1-yl-2,2-d2)methanone (6c)

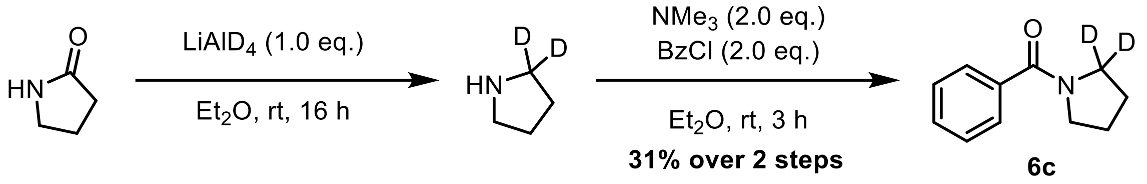

A modified procedure adopted from literature was used.[24] Lithium aluminium deuteride (703 mg, 16.7 mmol, 1.00 eq.) was suspended in anhydrous Et_2O (50 mL) in a flame-dried flask (250 mL). The resulting suspension was cooled to 0 °C and 2-pyrrolidone (1.27 mL, 1.43 g, 16.7 mmol, 1.00 eq.) was added dropwise. The reaction mixture was stirred for 16 h at room temperature. The reaction was stopped by addition of a saturated solution of sodium sulfate until evolution of hydrogen ceased. The resulting clear solution was filtered from the off-white precipitate and the filtrate was collected in a separate flame-dried flask (250 mL). Then, benzoyl chloride (3.89 mL, 33.5 mmol, 2.00 eq.) and trimethylamine (4.67 mL, 33.5 mmol, 2.00 eq.) were added to the filtrate, and the mixture was stirred for 3 h at room temperature. The mixture was transferred to a separation funnel by using Et_2O (50 mL). The organic layer was extracted with 1 N HCl (100 mL) and saturated Na_2CO_3 (100 mL), dried over anhydrous $MgSO_4$ and concentrated. The crude product was purified by column chromatography (heptane/EtOAc, 8:2 to pure EtOAc) to afford 904 mg (31%) of the deuterated product. The spectra were found to be in accordance with the literature.[24]

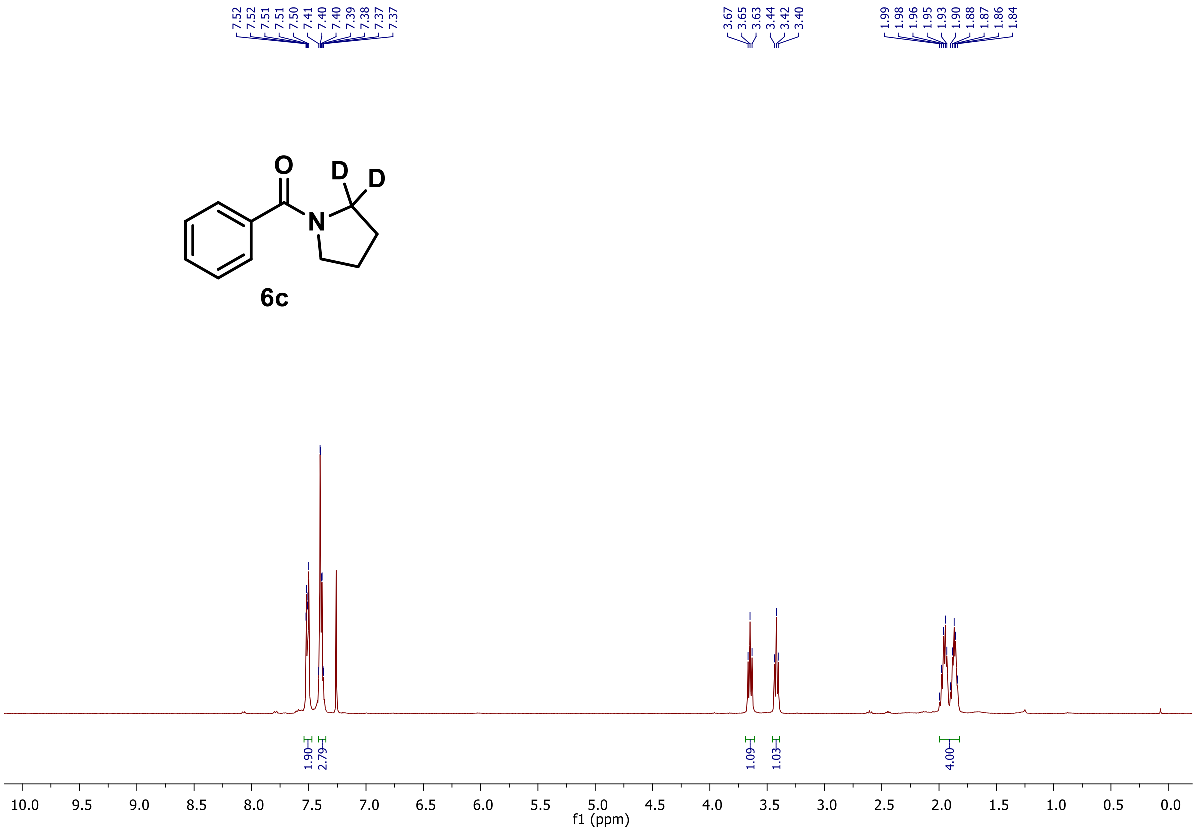

N-(4λ[12]-buta-1,3-diyn-1-yl-d_9)-N-butyl-2,4,6-trimethylbenzamide (6f)

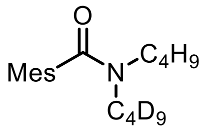

Following the GP1A (2.0 mmol) using benzoyl chloride and dibutylamine-(monobutyl-d_9) (purchased from Sigma Aldrich) afforded the desired amide (535 mg, 1.88 mmol, 94%) as colorless oil.

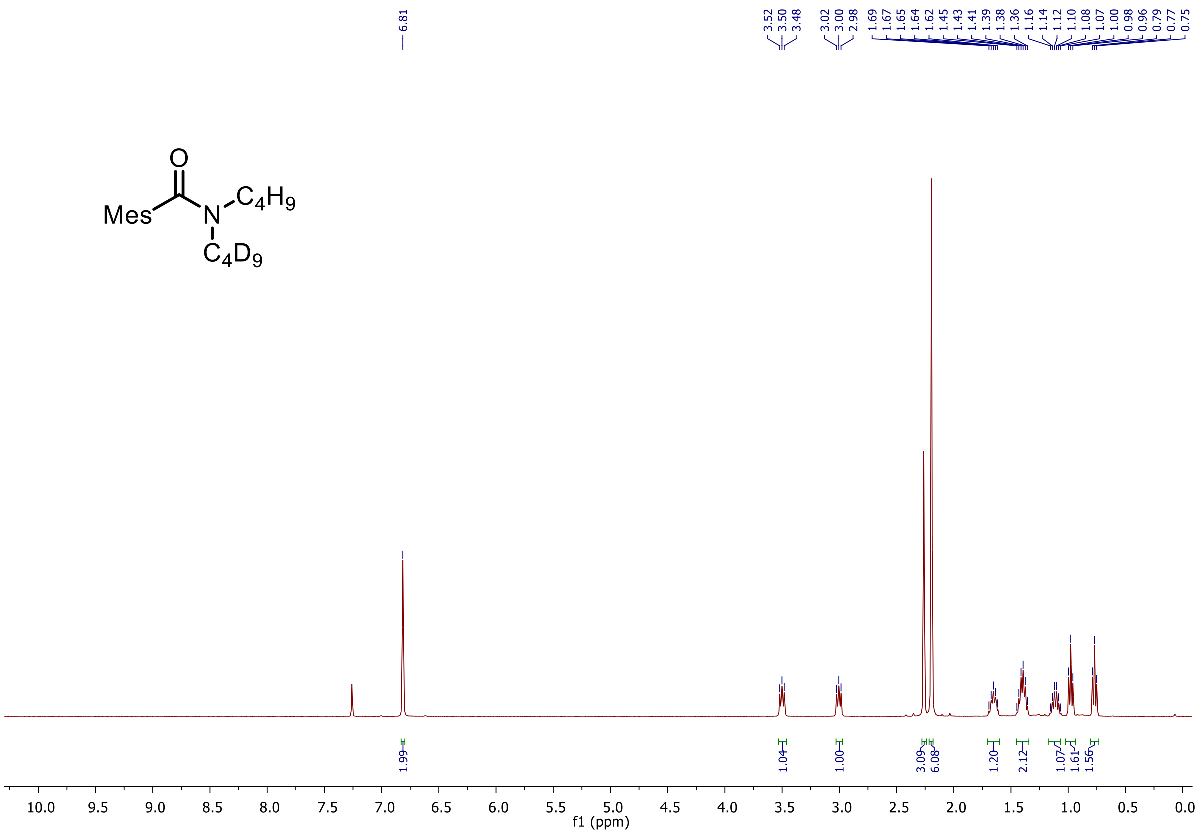

4.1.2. *N*-Dehydrogenation Reaction with Isotopically-labeled Substrates

Scheme 1. Overview of isotope-labeling experiments.

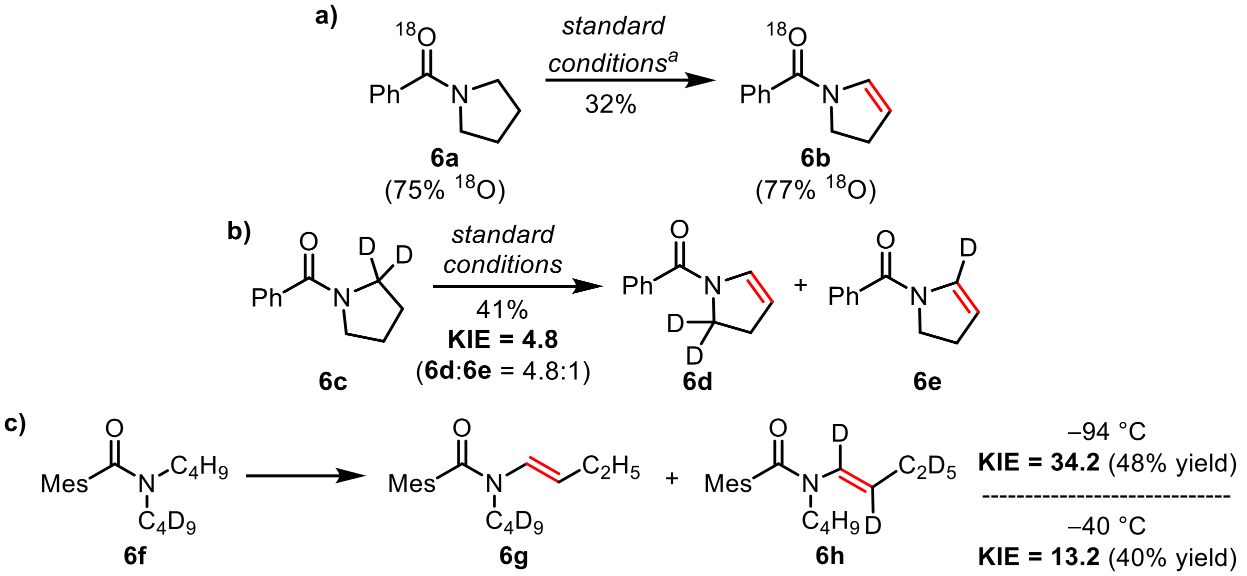

*Substrates **6a**, **6c** and **6f** have been conducted to GP3; [a] deviation from standard conditions: -78 °C and THF.*

Experiment A

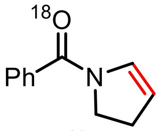

6b
(77% ^{18}O)

Following GP3 (deviation: -78 °C and THF as solvent) using **6a** yielded the desired enamide as a colorless oil (17.9 mg, 32 %). HRMS analysis revealed an ^{18}O content of 77%.

Acquisition Parameter

Source Type	ESI	Ion Polarity	Positive	Set Nebulizer	0.4 Bar
Focus	Not active	Set Capillary	4200 V	Set Dry Heater	180 °C
Scan Begin	80 m/z	Set End Plate Offset	-500 V	Set Dry Gas	4.0 l/min
Scan End	1900 m/z	Set Charging Voltage	0 V	Set Divert Valve	Source
		Set Corona	0 nA	Set APCI Heater	0 °C

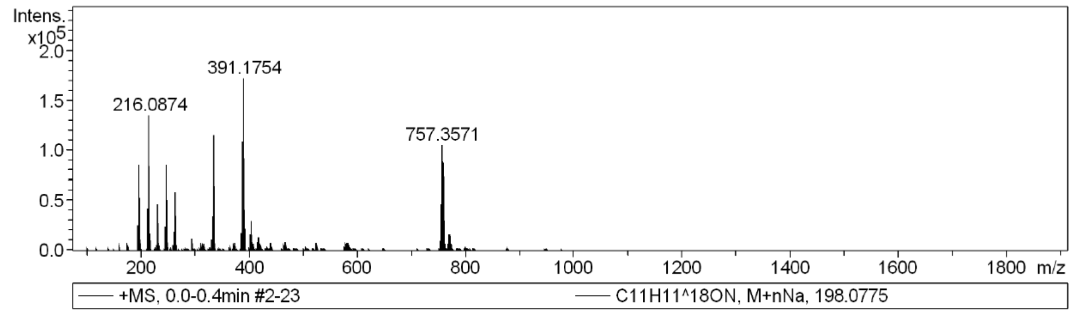

#	m/z	I
1	196.0725	26102
2	198.0770	85017

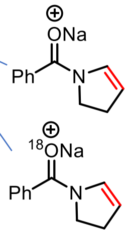

Exact Mass: 196.0733

Exact Mass: 198.0775

Experiment B

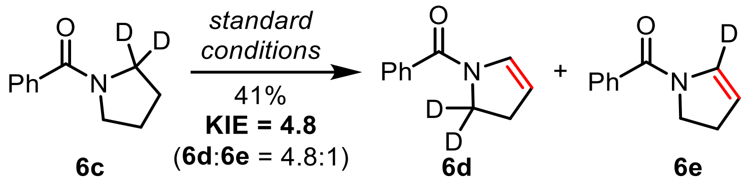

Following GP3 using **6c** yielded the desired enamide as a colorless oil (23.3 mg, 41%). For the calculation of the KIE value, the ratio of **6d** to **6e** was determined by NMR spectroscopy as well as HMRS analysis.

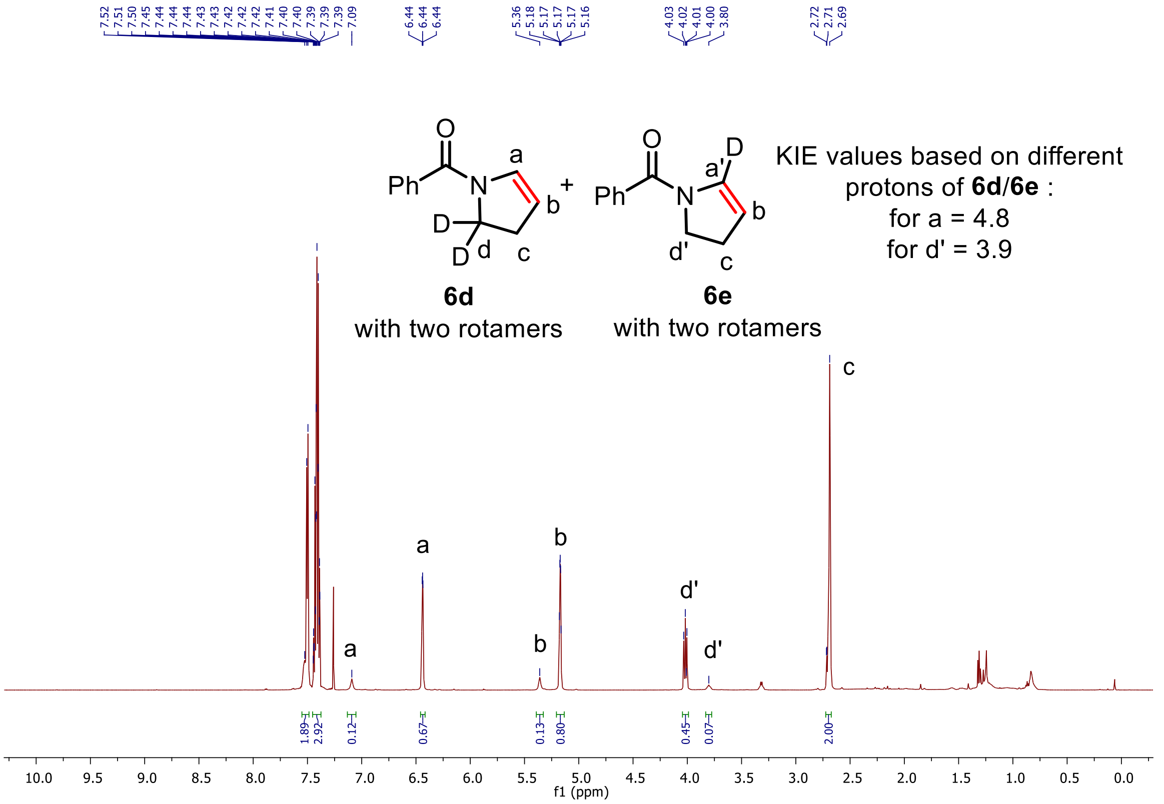

Acquisition Parameter

Source Type	ESI	Ion Polarity	Positive	Set Nebulizer	0.4 Bar
Focus	Not active	Set Capillary	4200 V	Set Dry Heater	180 °C
Scan Begin	50 m/z	Set End Plate Offset	-500 V	Set Dry Gas	4.0 l/min
Scan End	1900 m/z	Set Charging Voltage	0 V	Set Divert Valve	Source
		Set Corona	0 nA	Set APCI Heater	0 °C

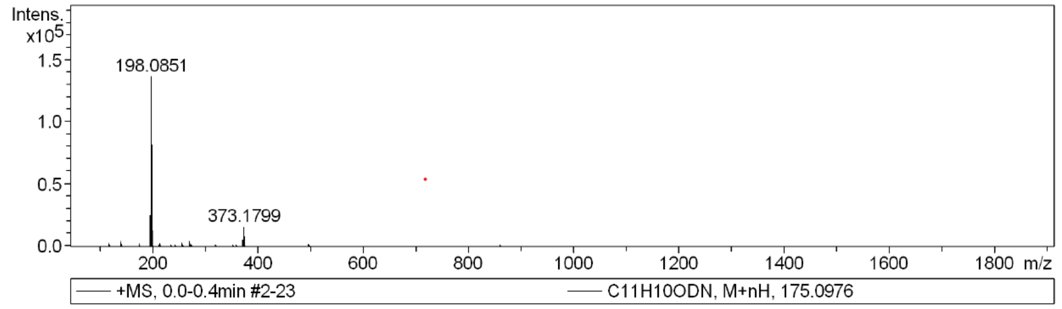

#	m/z	I
1	118.1228	2376
2	140.1046	2877
3	176.1030	576
4	196.0731	5237
5	197.0792	25286
6	198.0851	136170
7	199.0884	17580
8	200.0916	1187

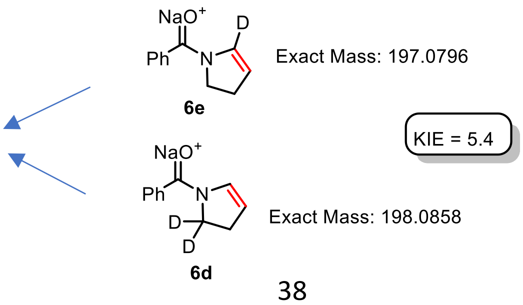

Experiment C

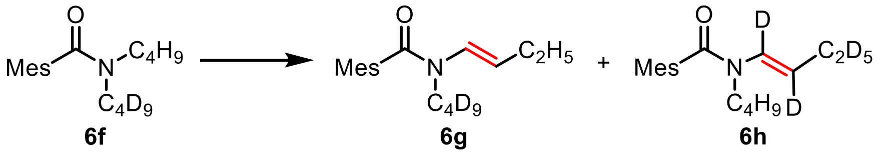

–94 °C
KIE = 34.2 (48% yield)

–40 °C
KIE = 13.2 (40% yield)

Following GP3 (–94 °C) using **6f** yielded the desired enamide as a colorless oil (40.7 mg, 48%). For the calculation of the KIE value, the ratio **6g/6f** was determined by NMR spectroscopy as well as HMRS analysis.

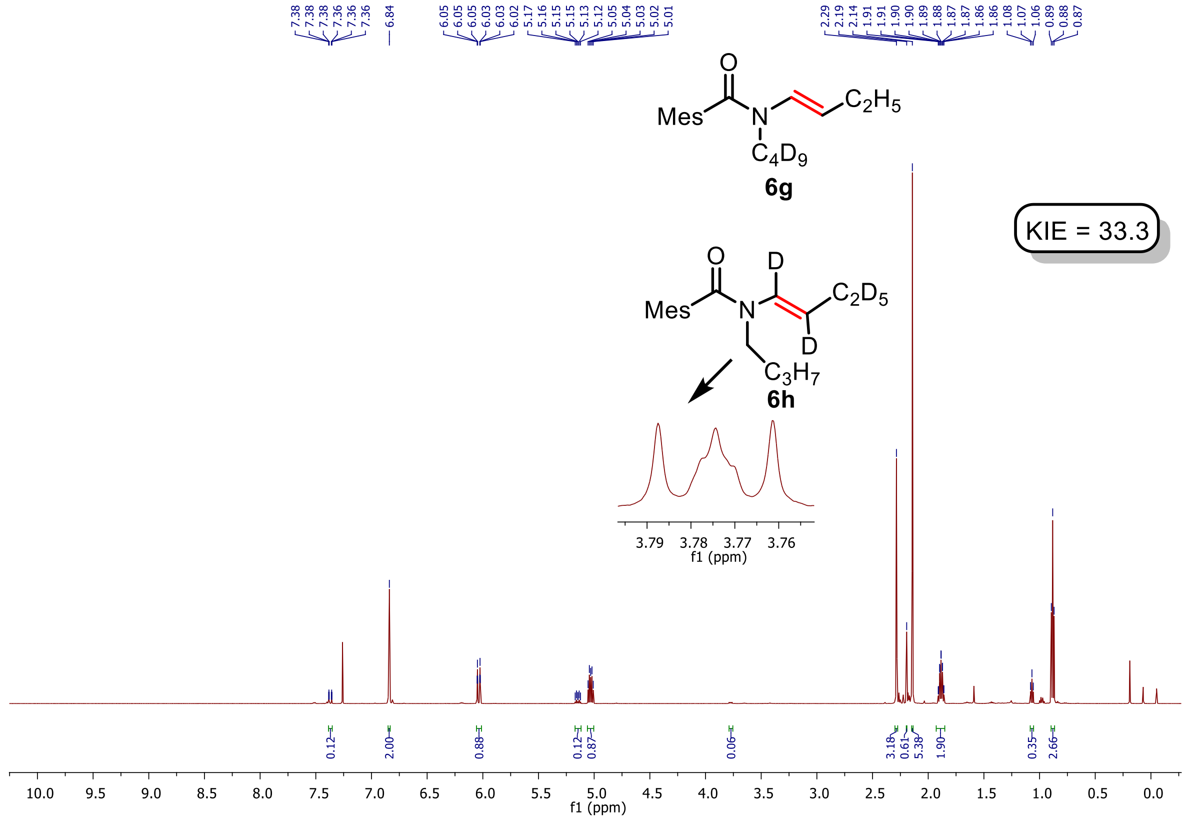

Acquisition Parameter

Source Type	ESI	Ion Polarity	Positive	Set Nebulizer	0.4 Bar
Focus	Not active	Set Capillary	4200 V	Set Dry Heater	180 °C
Scan Begin	50 m/z	Set End Plate Offset	-500 V	Set Dry Gas	4.0 l/min
Scan End	1900 m/z	Set Charging Voltage	0 V	Set Divert Valve	Source
		Set Corona	0 nA	Set APCI Heater	0 °C

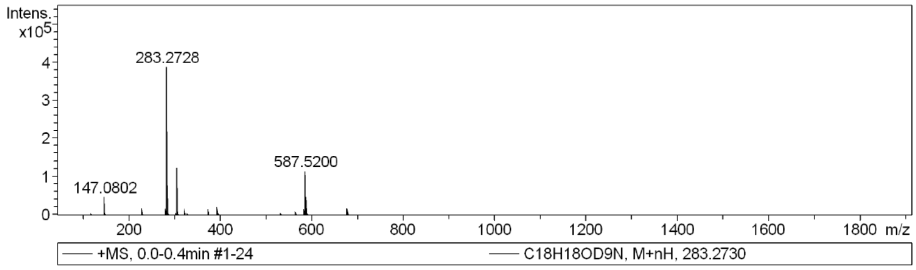

#	m/z	I
1	147.0802	47002
2	148.0835	5319
3	229.2256	11560
4	281.2598	11276
5	282.2658	14149
6	283.2728	386093
7	284.2761	78015
8	285.2857	20753

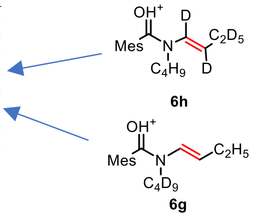

Exact Mass: 281.2605

KIE = 34.2

Exact Mass: 283.2730

In order to check the temperature effect on the KIE for substrate **6f**, the same reaction (0.2 mmol scale) was performed at –41 °C (cooling system: MeCN + dry ice) to provide **6g/6f** (33.6 mg, 40%). For the calculation of the KIE value, the ratio **6g/6f** was determined by NMR spectroscopy as well as HMRS analysis.

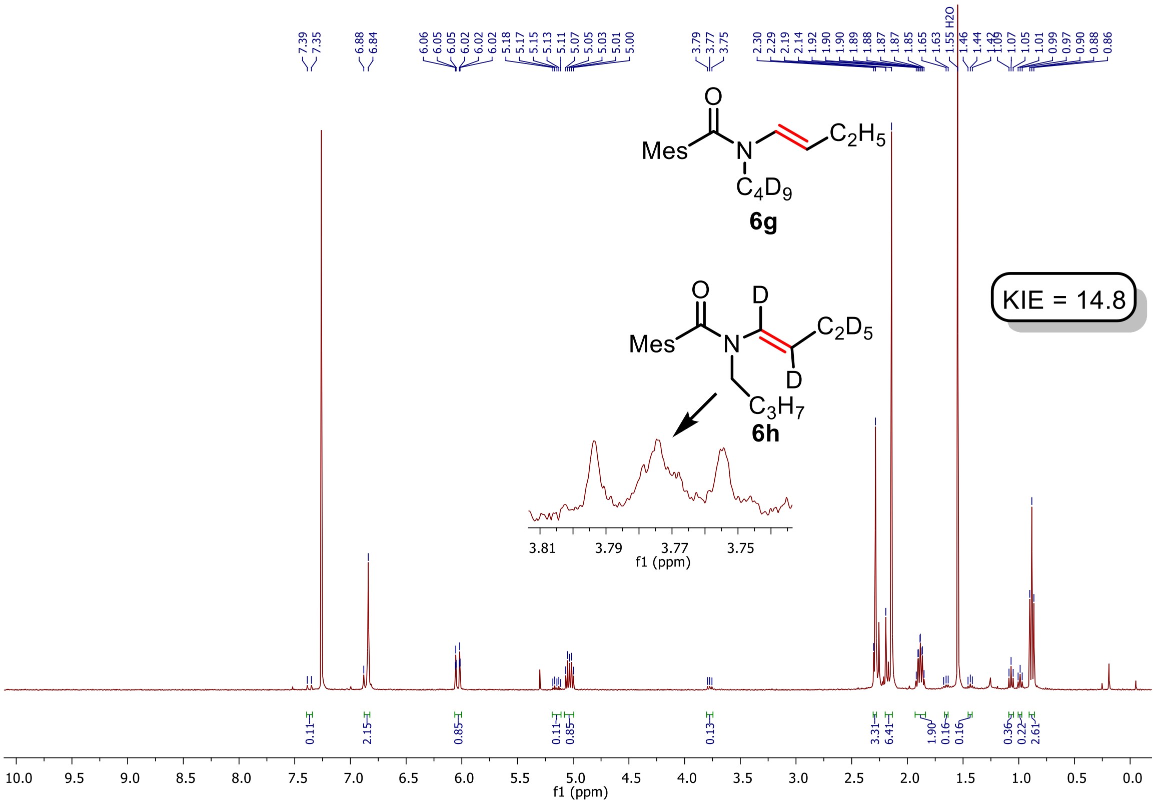

Acquisition Parameter

Source Type	ESI	Ion Polarity	Positive	Set Nebulizer	0.4 Bar
Focus	Not active	Set Capillary	4200 V	Set Dry Heater	180 °C
Scan Begin	50 m/z	Set End Plate Offset	-500 V	Set Dry Gas	4.0 l/min
Scan End	1900 m/z	Set Charging Voltage	0 V	Set Divert Valve	Source
		Set Corona	0 nA	Set APCI Heater	0 °C

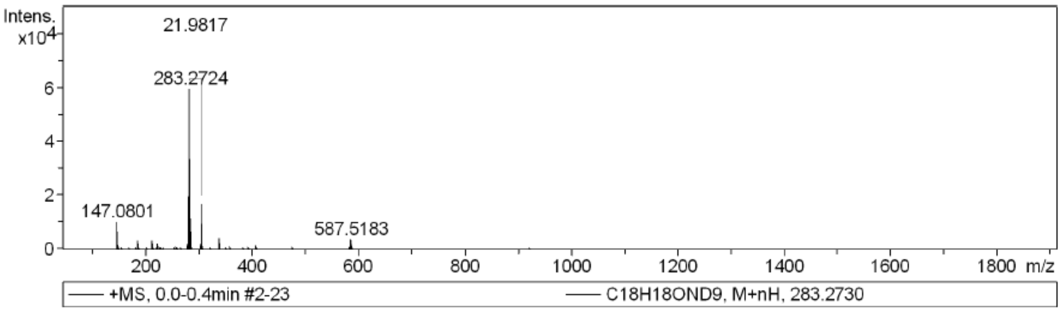

#	m/z	I
1	145.9880	729
2	147.0801	10042
3	148.0835	1198
4	186.2213	3320
5	212.9993	3205
6	222.1122	2048
7	258.9597	725
8	279.0530	1282
9	280.0599	1113
10	281.0491	19366
11	281.2594	4492
12	282.0559	17247
13	282.2650	2469
14	283.0613	4586
15	283.2724	59312

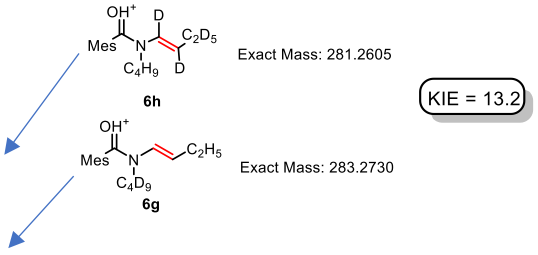

4.2. Analysis of the Crude Material

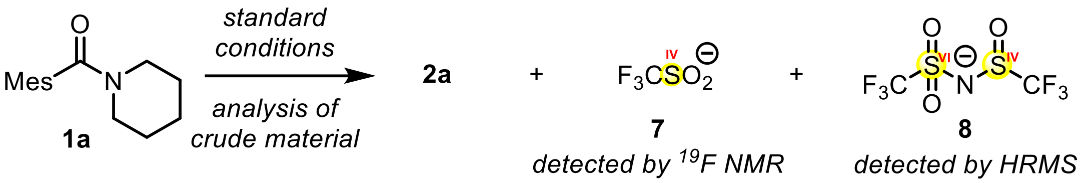

Following GP3 with substrate **1a**, the obtained crude material was analyzed (without quenching with NH_4Cl):
1) *via* ^{19}F NMR spectroscopy (d^6-DMSO was used as deuterated solvent). By the addition of 1 equivalent of Langlois salt (CF_3SO_2Na) which lead to an increase of signal **a**, the presence of **7** was proven. Additional experiments **A** and **B** were conducted to provide more insight of the additional fluorine species origin in the analyzed material.

^{19}F NMR spectroscopy:

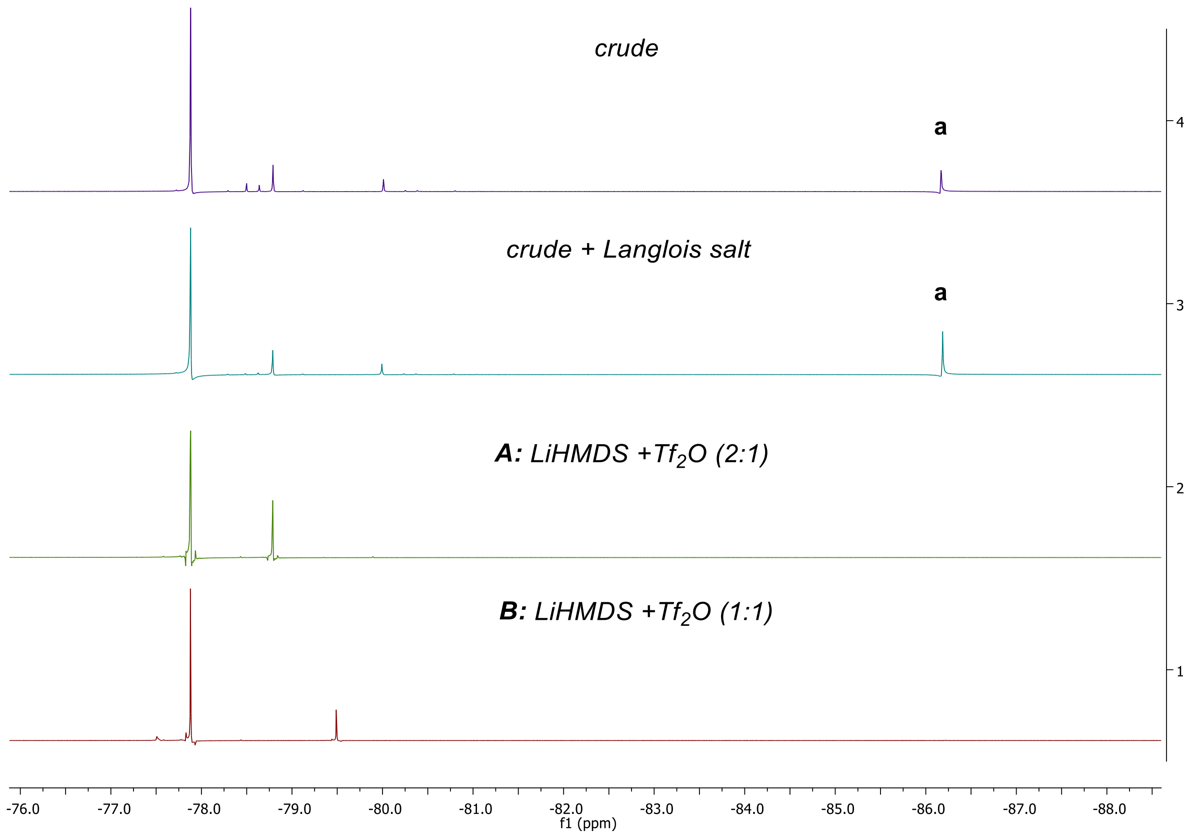

2) *via* HRMS

Species **8** was detected by HRMS:

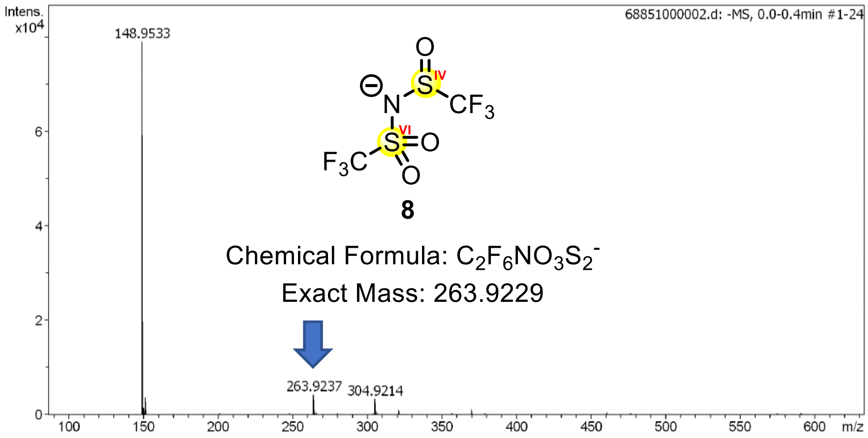

Proposed mechanism for the formation of **8**:

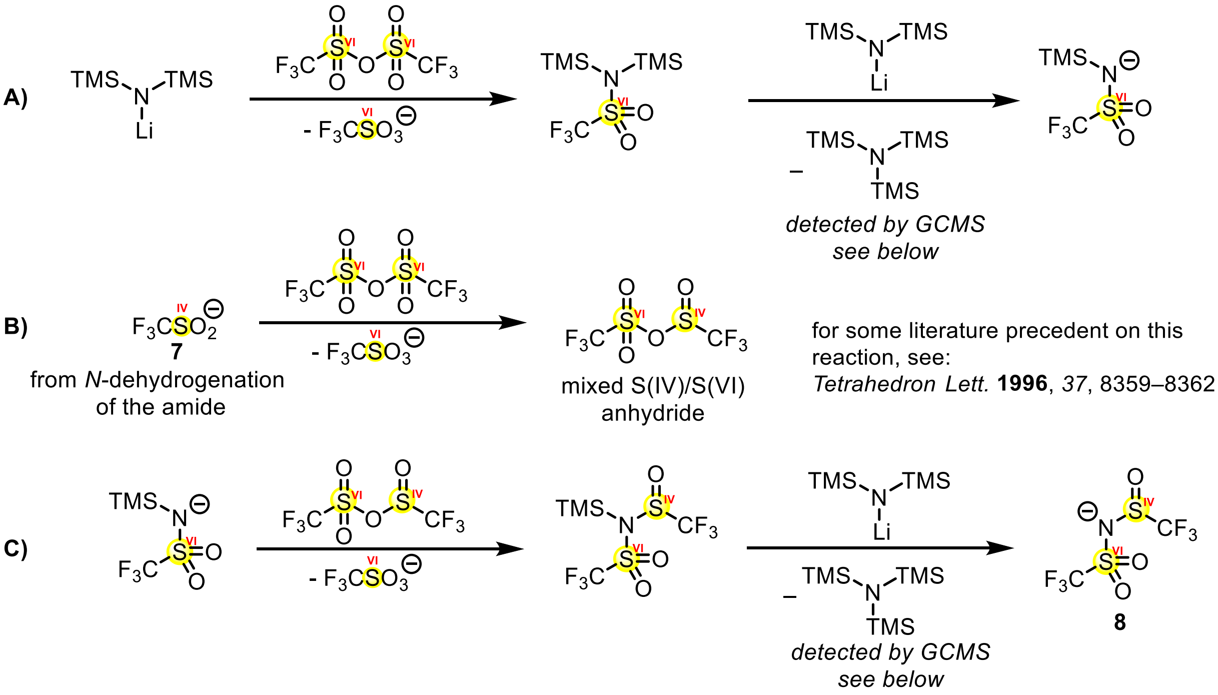

GCMS of worked-up crude (with substrate **1a**) showing the presence of $N(TMS)_3$:

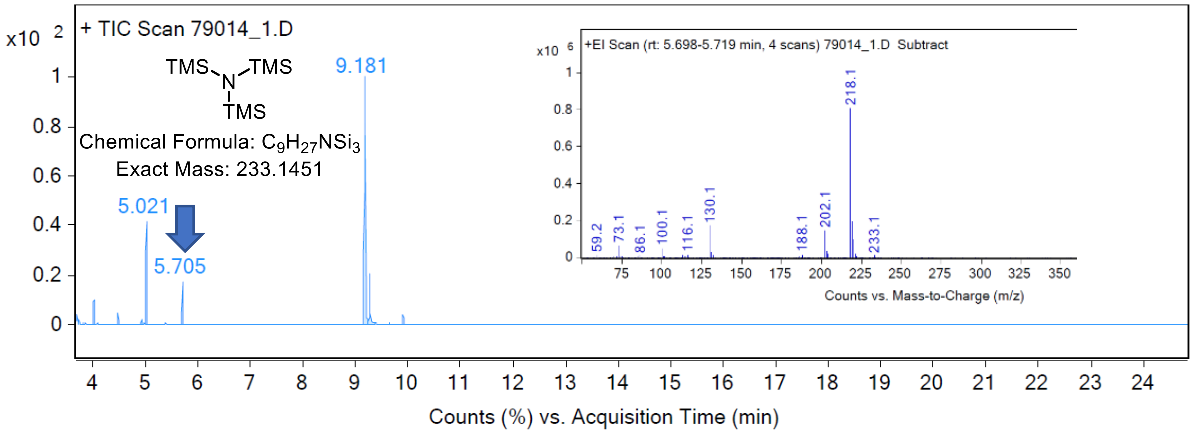

4.3. Control Experiment

Potential alternative pathway:

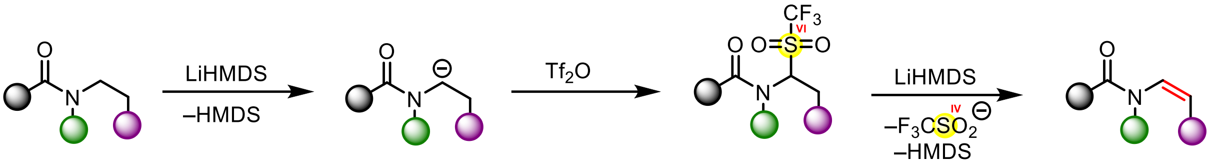

To test the possibility of the pathway shown above, following experiment was performed which did not reveal any deuterium incorporation:

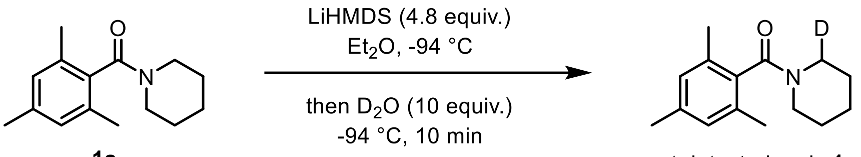

In a flame-dried Schlenk flask, **1a** (69.4 mg, 0.30 mmol, 1.00 eq.) was dissolved in anhydrous Et_2O (1.5 mL). The mixture was cooled down to -94 °C and LiHMDS (1 M in THF, 1.44 mL, 0.72 mmol, 4.80 eq., 1 M in THF) was slowly added. The mixture was stirred for 10 min, then D_2O (54.1 µL, 3.00 mmol, 10.0 eq.) was added in one portion. The reaction mixture was extracted with CH_2Cl_2 (3 x 10 mL), the organic phases were combined and dried over anhydrous $MgSO_4$. After filtration and evaporation of solvent, the crude material was analyzed by NMR spectroscopy and mass spectrometry. No incorporation of deuterium was detected by both methods, instead pure **1a**, with no signs of degradation, was recovered after extraction. Therefore, we exclude the possibility of the route shown above.

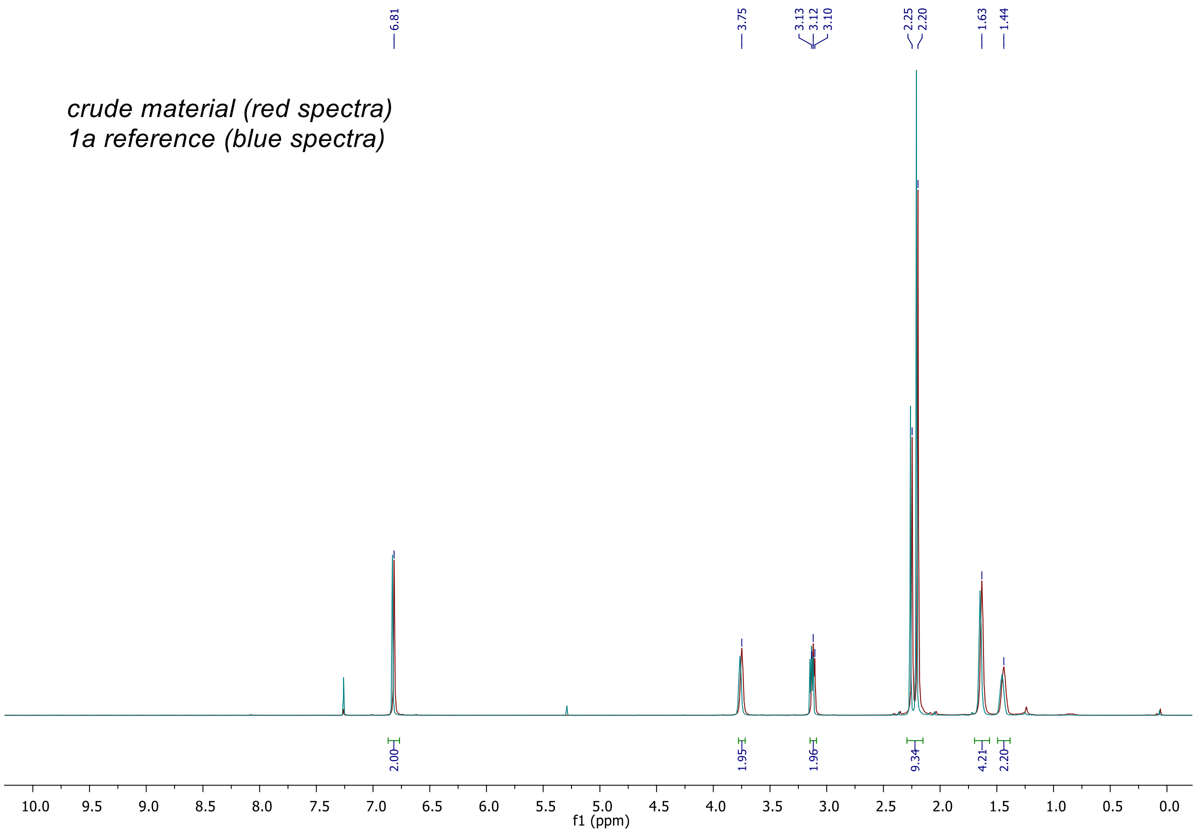

5. References

[1] T. E. Stang, P. J., Dueber, *Org. Synth.* **1974**, *54*, 79.
[2] T. Furukawa, M. Tobisu, N. Chatani, *Bull. Chem. Soc. Jpn.* **2016**, *90*, 332–342.
[3] M. Holik, A. Mannschreck, *Org. Magn. Reson.* **1979**, *12*, 223–228.
[4] J. Clayden, P. Johnson, J. H. Pink, *J. Chem. Soc. Perkin Trans. 1* **2001**, 371–375.
[5] J. Karthikeyan, N. Yoshikai, *Org. Lett.* **2014**, *16*, 4224–4227.
[6] W. Huang, M.-L. Xu, *J. Chem. Res.* **2013**, *37*, 77–79.
[7] G.-P. Yang, K. Li, W. Liu, K. Zeng, Y.-F. Liu, *Org. Biomol. Chem.* **2020**, *18*, 6958–6964.
[8] Z. Yin, Z. Wang, X.-F. Wu, *European J. Org. Chem.* **2017**, *2017*, 3992–3995.
[9] N. Sharma, G. Sekar, *Adv. Synth. Catal.* **2016**, *358*, 314–320.
[10] G. Pandey, S. Koley, R. Talukdar, P. K. Sahani, *Org. Lett.* **2018**, *20*, 5861–5865.
[11] S. Srinivas Kotha, S. Badigenchala, G. Sekar, *Adv. Synth. Catal.* **2015**, *357*, 1437–1445.
[12] W. Li, X.-F. Wu, *Org. Lett.* **2015**, *17*, 1910–1913.
[13] X. Wu, W. Fan, Y. Pan, Y. Zhai, Y. Niu, C. Li, Q. Mei, *Molecules* **2014**, *19*, 1034–1046.
[14] J. Li, F. Xu, Y. Zhang, Q. Shen, *J. Org. Chem.* **2009**, *74*, 2575–2577.
[15] D.-Y. Huang, Q.-J. Yao, S. Zhang, X.-T. Xu, K. Zhang, B.-F. Shi, *Org. Lett.* **2019**, *21*, 951–954.
[16] Y.-S. Bao, M. Baiyin, B. Agula, M. Jia, B. Zhaorigetu, *J. Org. Chem.* **2014**, *79*, 6715–6719.
[17] N. Gigant, L. Chausset-Boissarie, M.-C. Belhomme, T. Poisson, X. Pannecoucke, I. Gillaizeau, *Org. Lett.* **2013**, *15*, 278–281.
[18] M. Feng, X. Jiang, *Chem. Commun.* **2014**, *50*, 9690–9692.
[19] O. Di Pietro, E. Viayna, E. Vicente-García, M. Bartolini, R. Ramón, J. Juárez-Jiménez, M. V. Clos, B. Pérez, V. Andrisano, F. J. Luque, R. Lavilla, D. M. Muñoz-Torrero, *Eur. J. Med. Chem.* **2014**, *73*, 141–152.
[20] J. B. Feltenberger, R. Hayashi, Y. Tang, E. S. C. Babiash, R. P. Hsung, *Org. Lett.* **2009**, *11*, 3666–3669.
[21] W. Marais, C. W. Holzapfel, *Synth. Commun.* **1998**, *28*, 3681–3691.
[22] J. B. Roque, Y. Kuroda, L. T. Göttemann, R. Sarpong, *Science* **2018**, *361*, 171–174.
[23] N. Gigant, L. Chausset-Boissarie, M.-C. Belhomme, T. Poisson, X. Pannecoucke, I. Gillaizeau, *Org. Lett.* **2013**, *15*, 278–281.
[24] R. Ito, N. Umezawa, T. Higuchi, *J. Am. Chem. Soc.* **2005**, *127*, 834–835.

6. NMR Spectra

Azepan-1-yl(mesityl)methanone (1c)

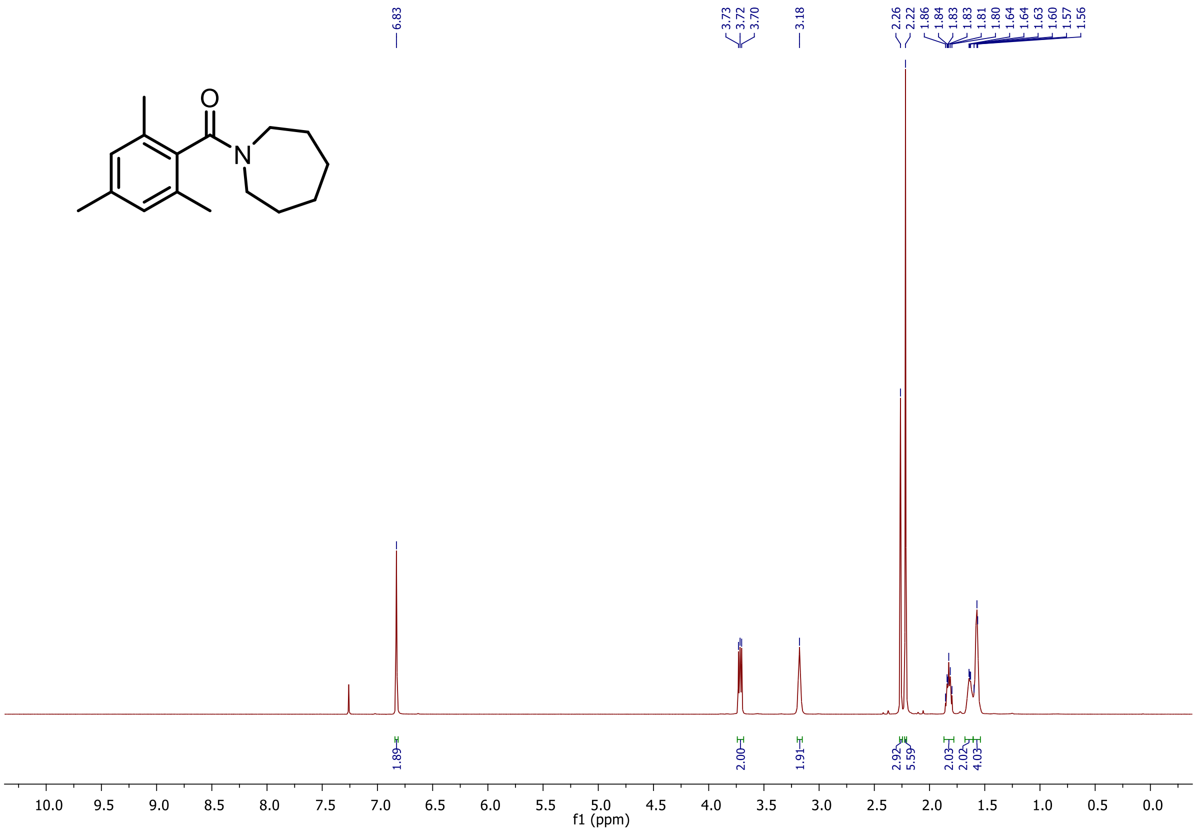

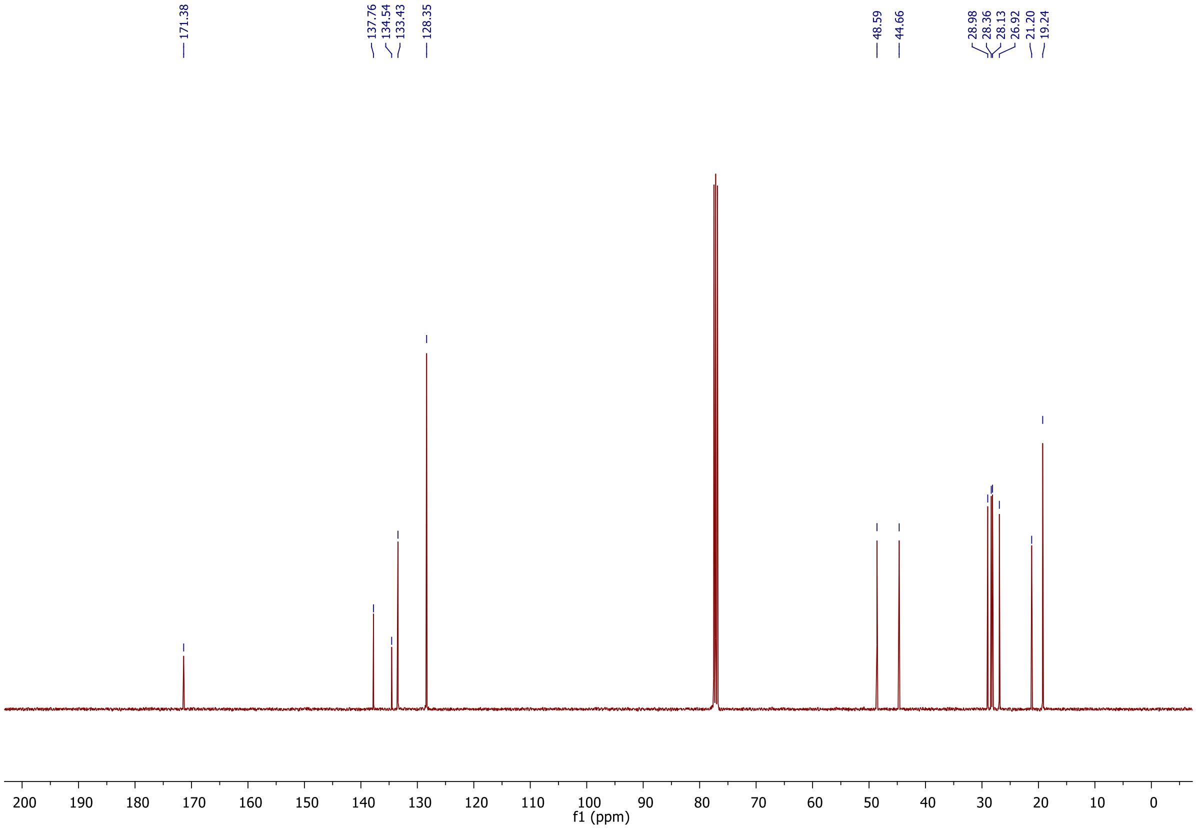

Mesityl(1,4-dioxa-8-azaspiro[4.5]decan-8-yl)methanone (1d)

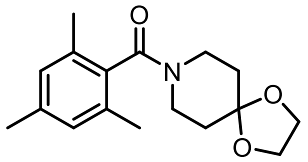

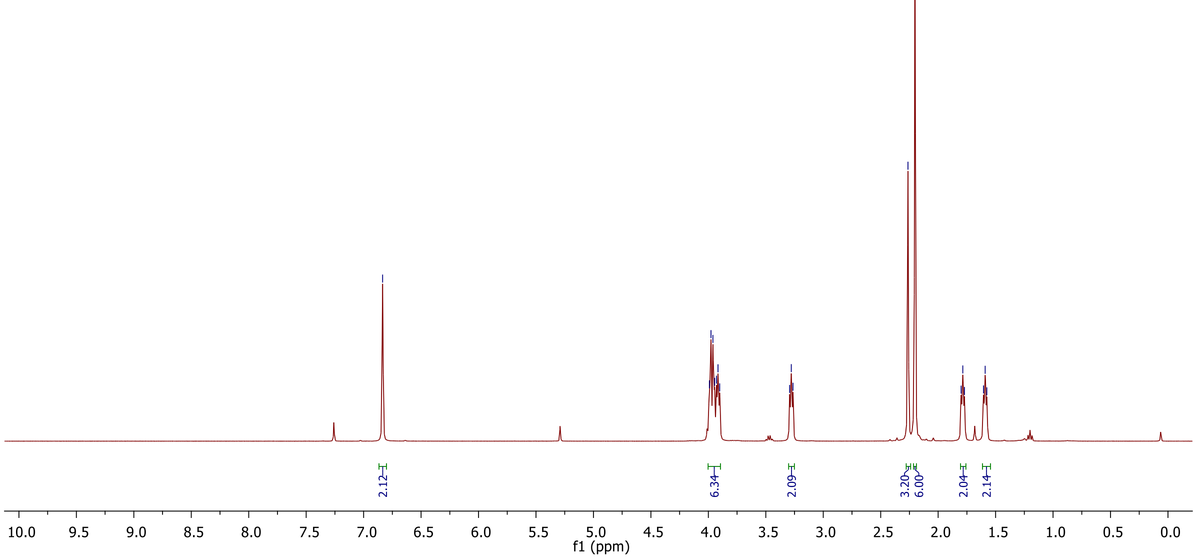

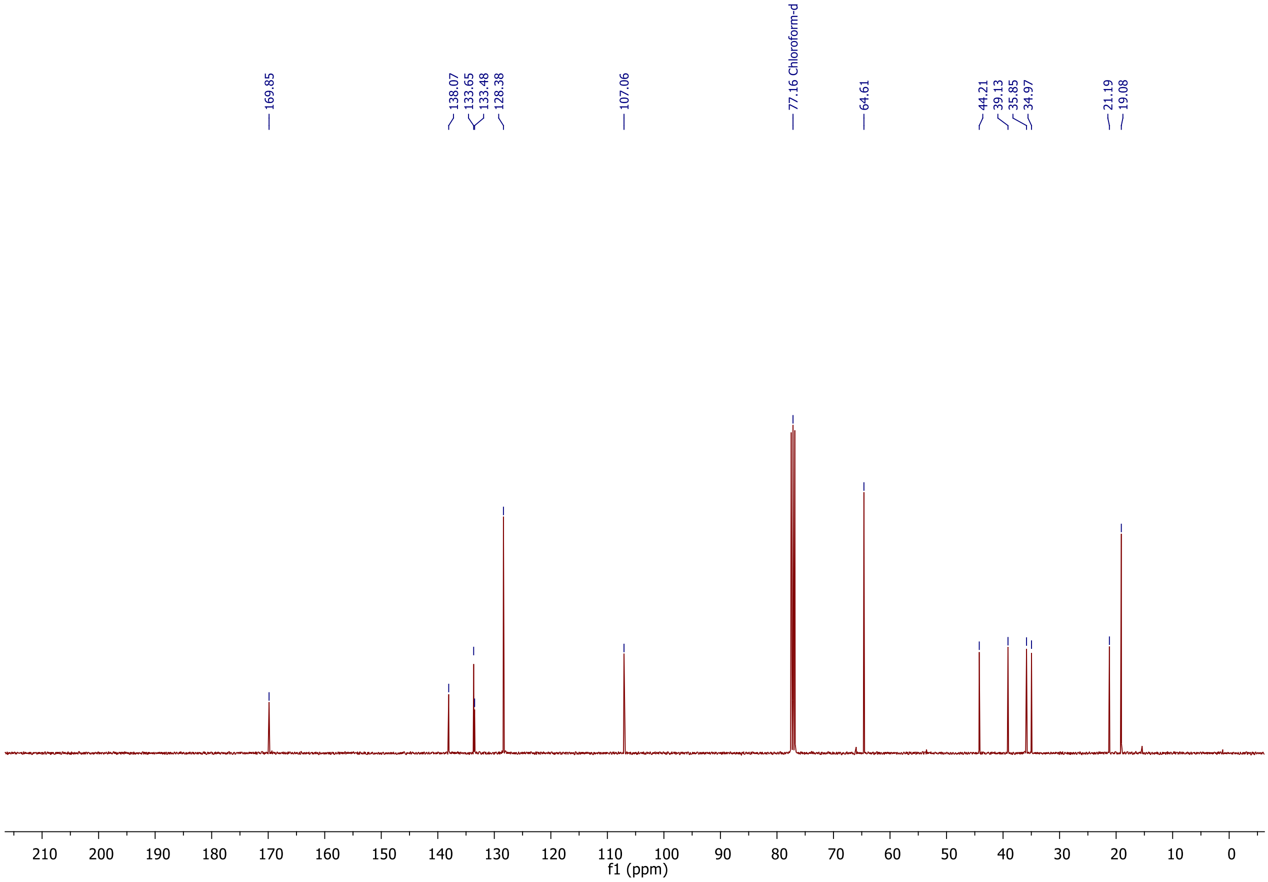

Mesityl(octahydro-2H-isoindol-2-yl)methanone (1e)

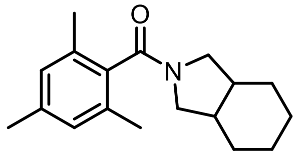

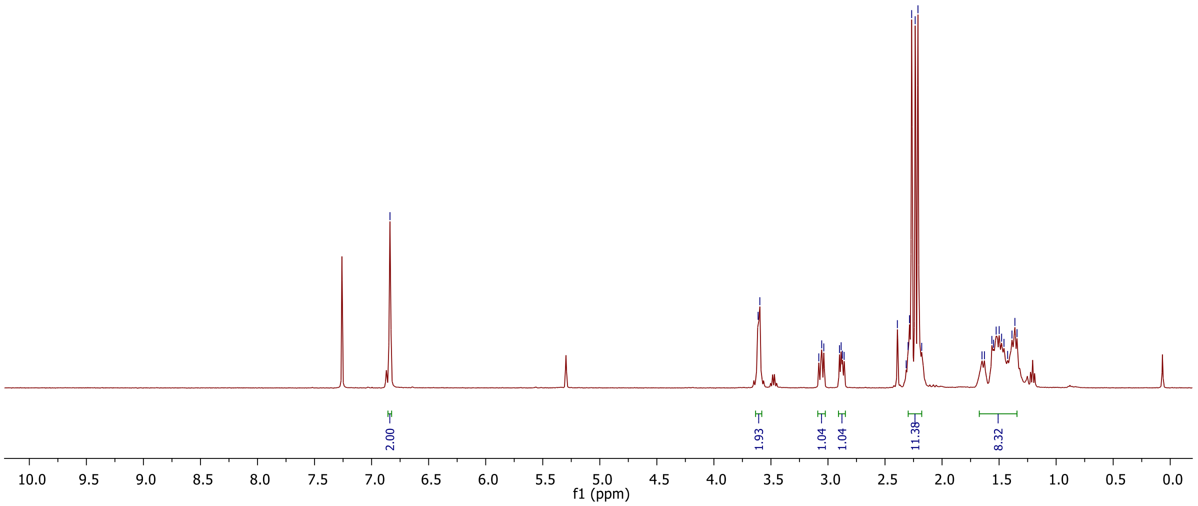

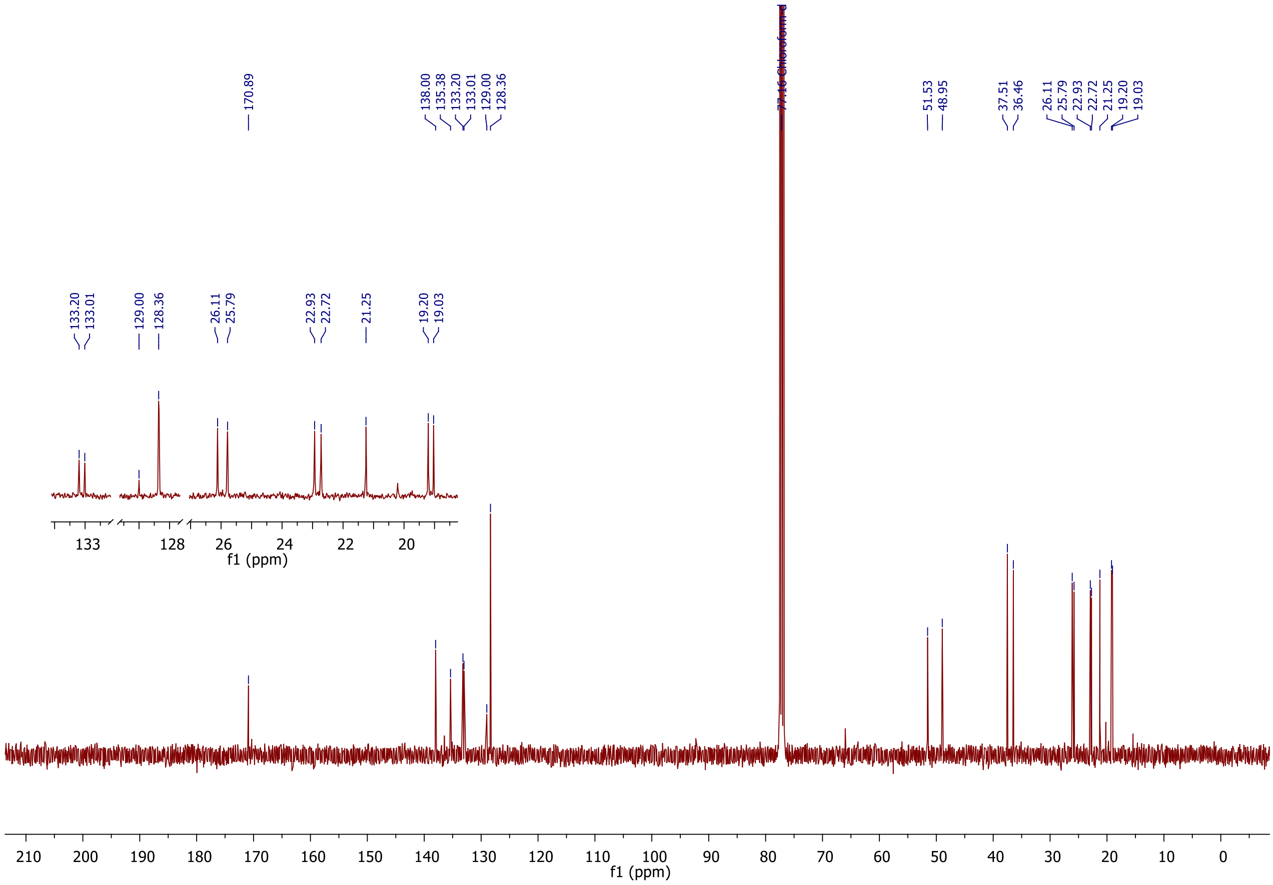

Mesityl(morpholino)methanone (1f)

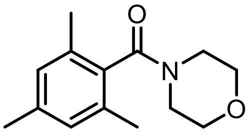

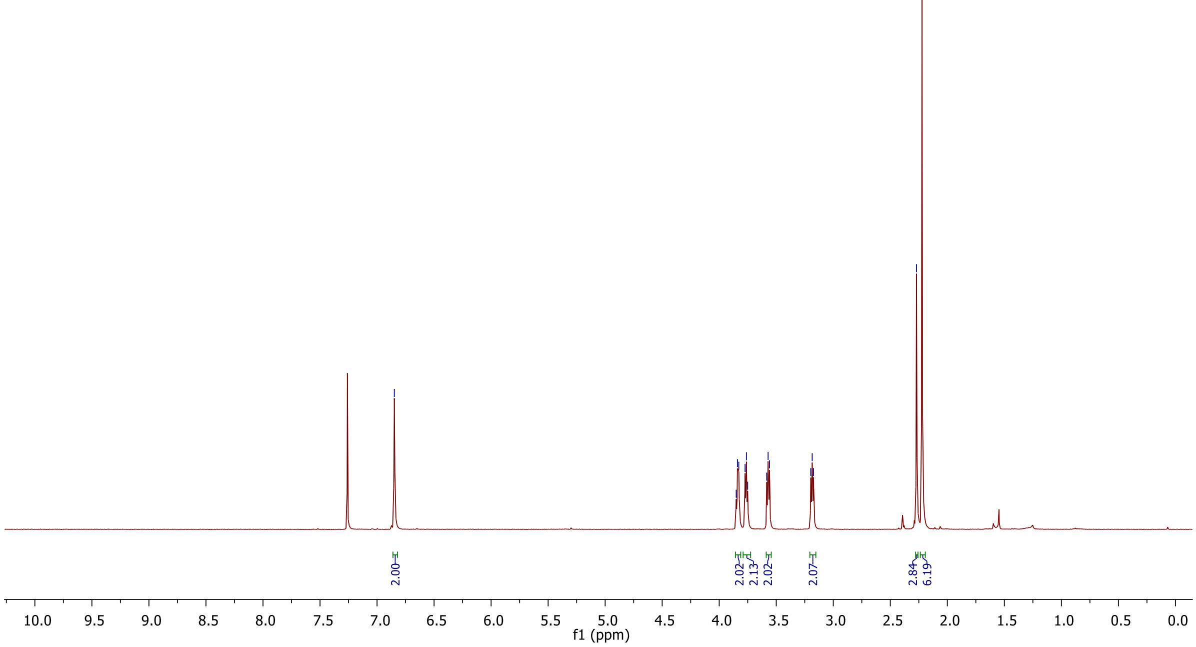

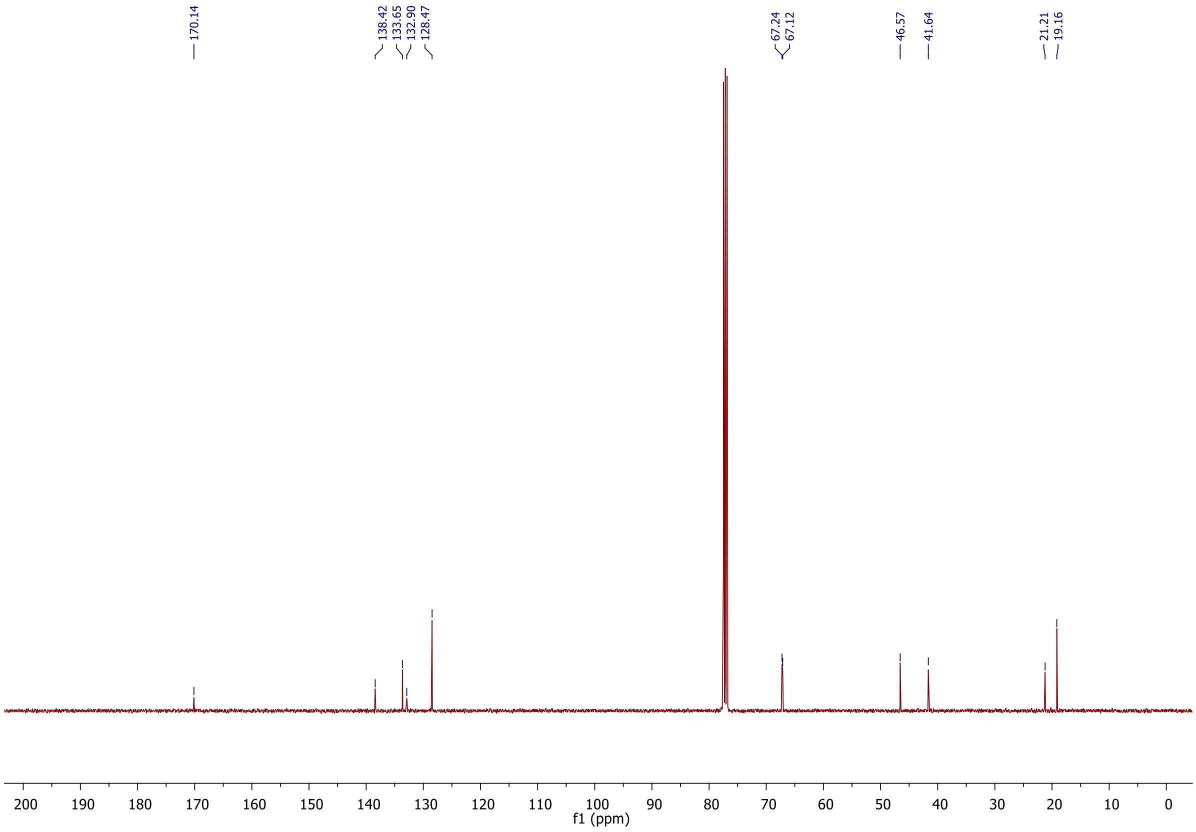

Ethyl 4-(2,4,6-trimethylbenzoyl)-3,4-dihydropyrazine-1(2H)-carboxylate (1g)

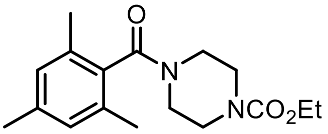

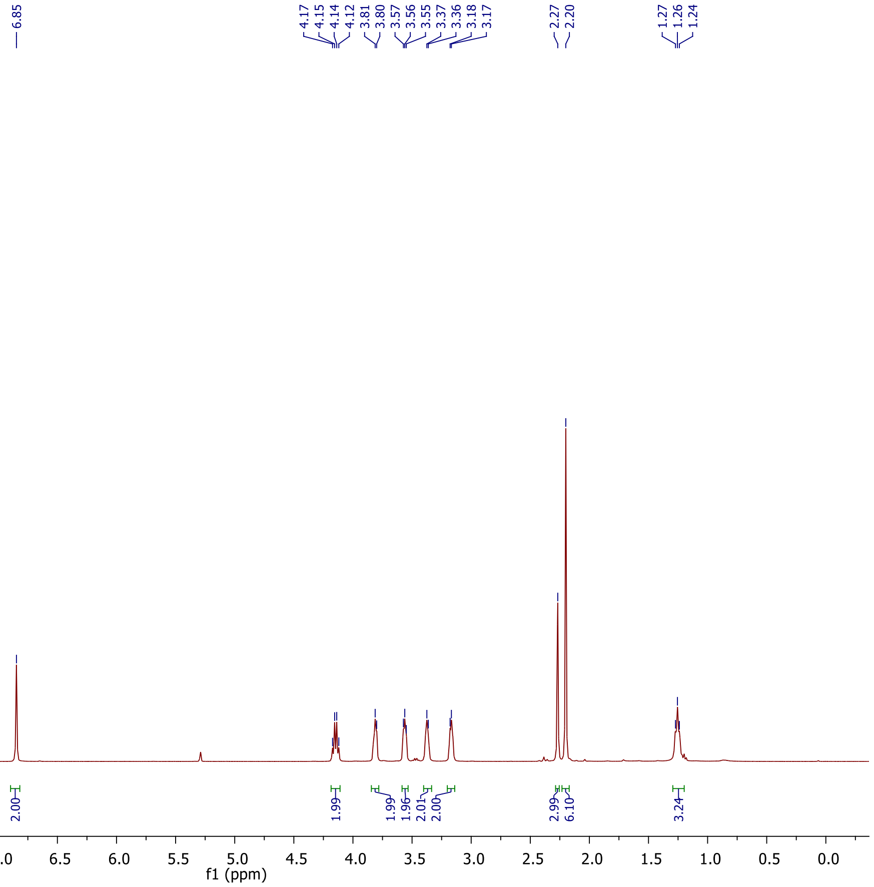

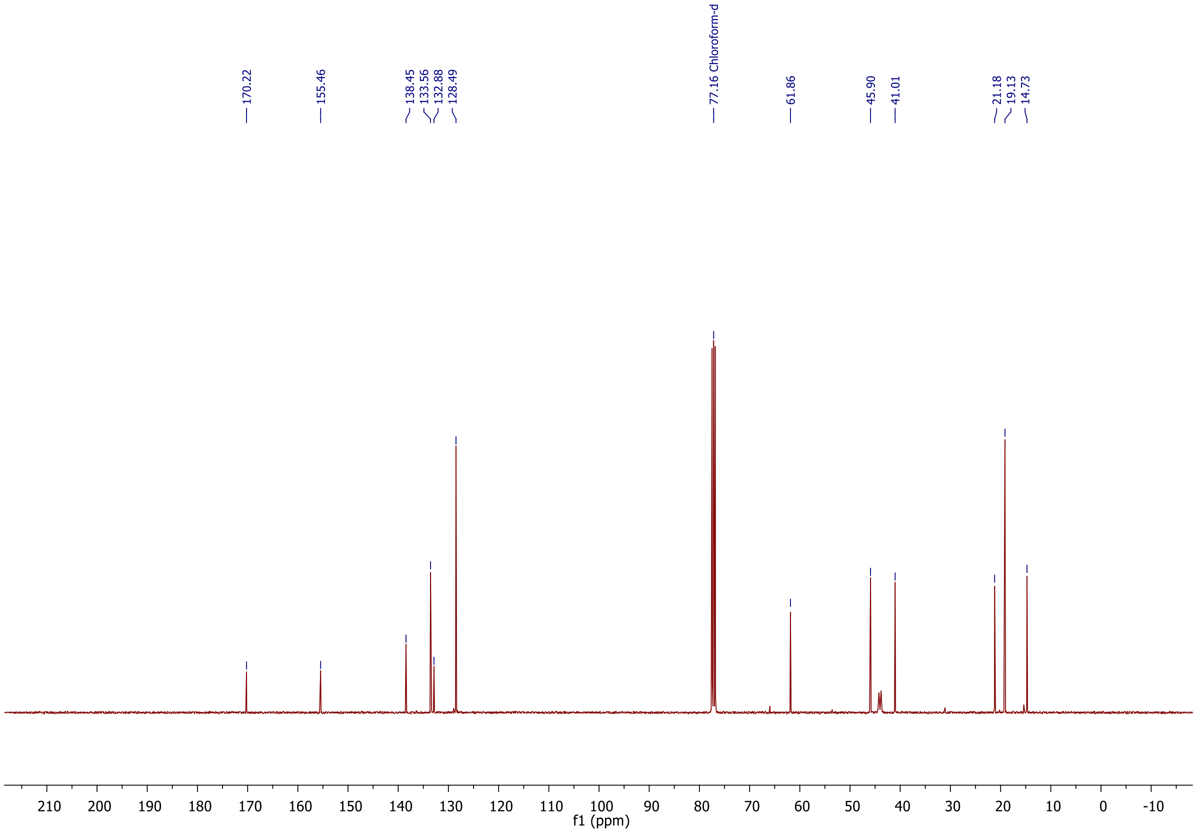

2,4,6-Trimethyl-N,N-dipropylbenzamide (1i)

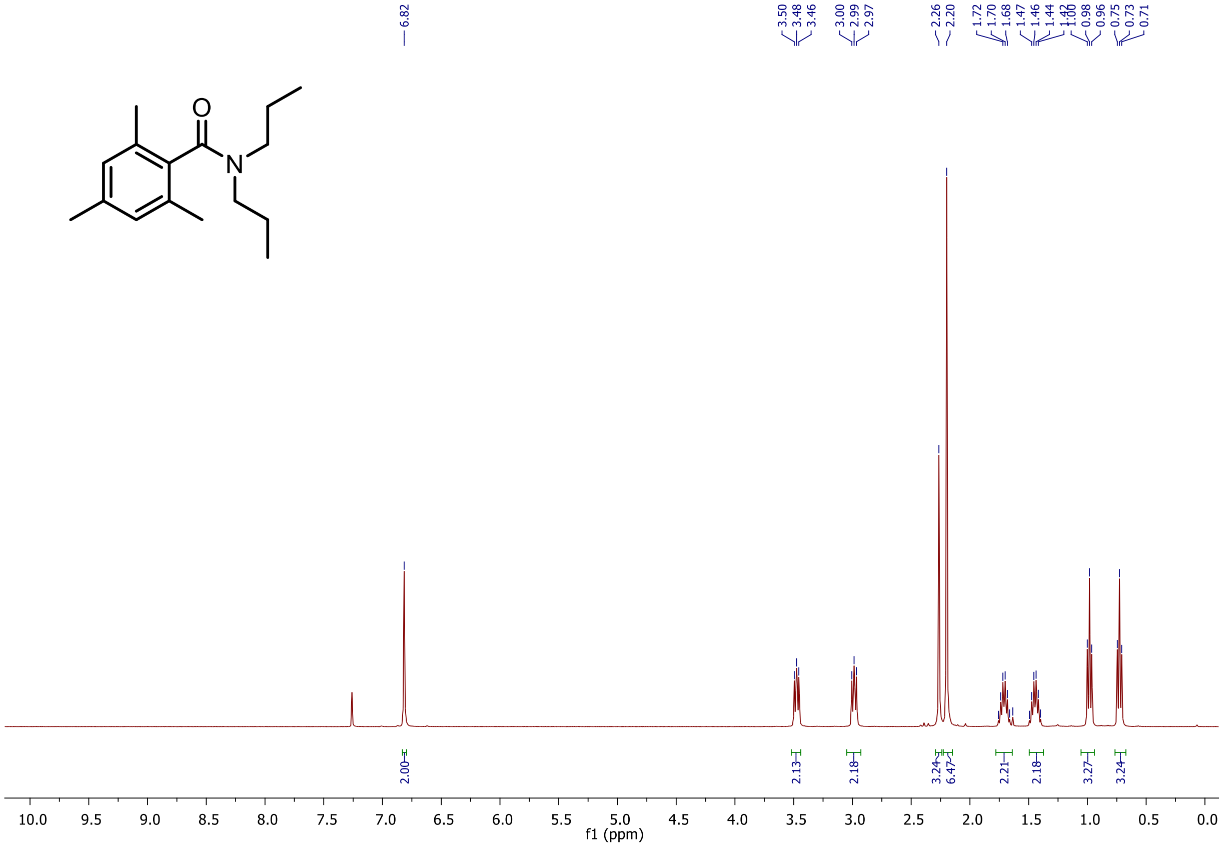

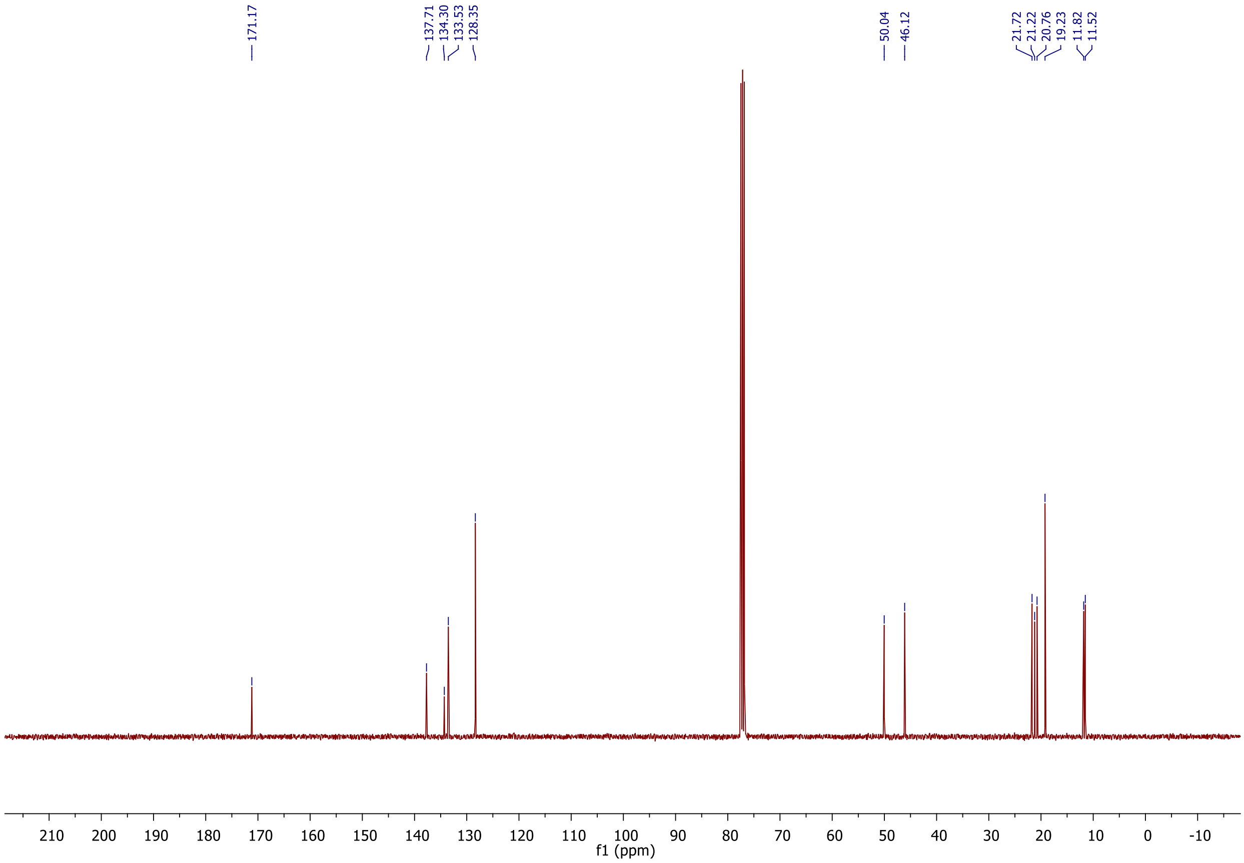

N,N-Dibutyl-2,4,6-trimethylbenzamide (1j)

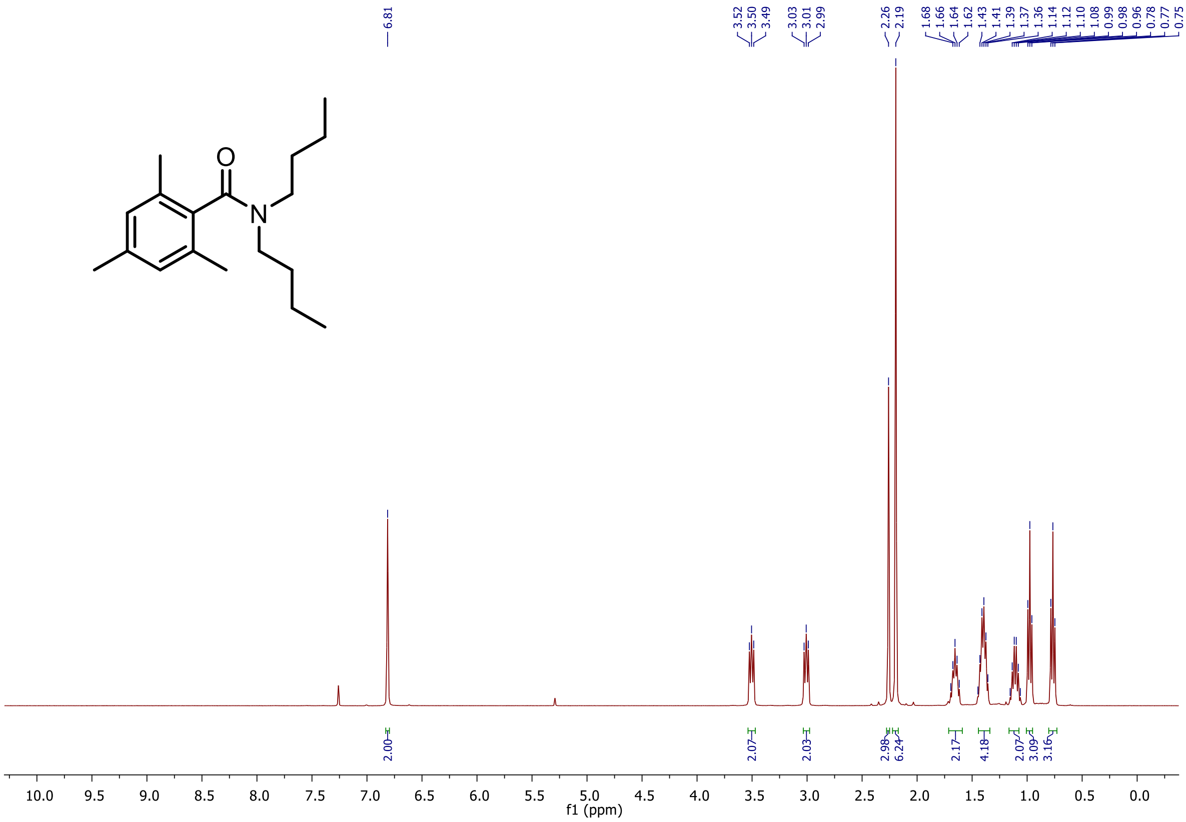

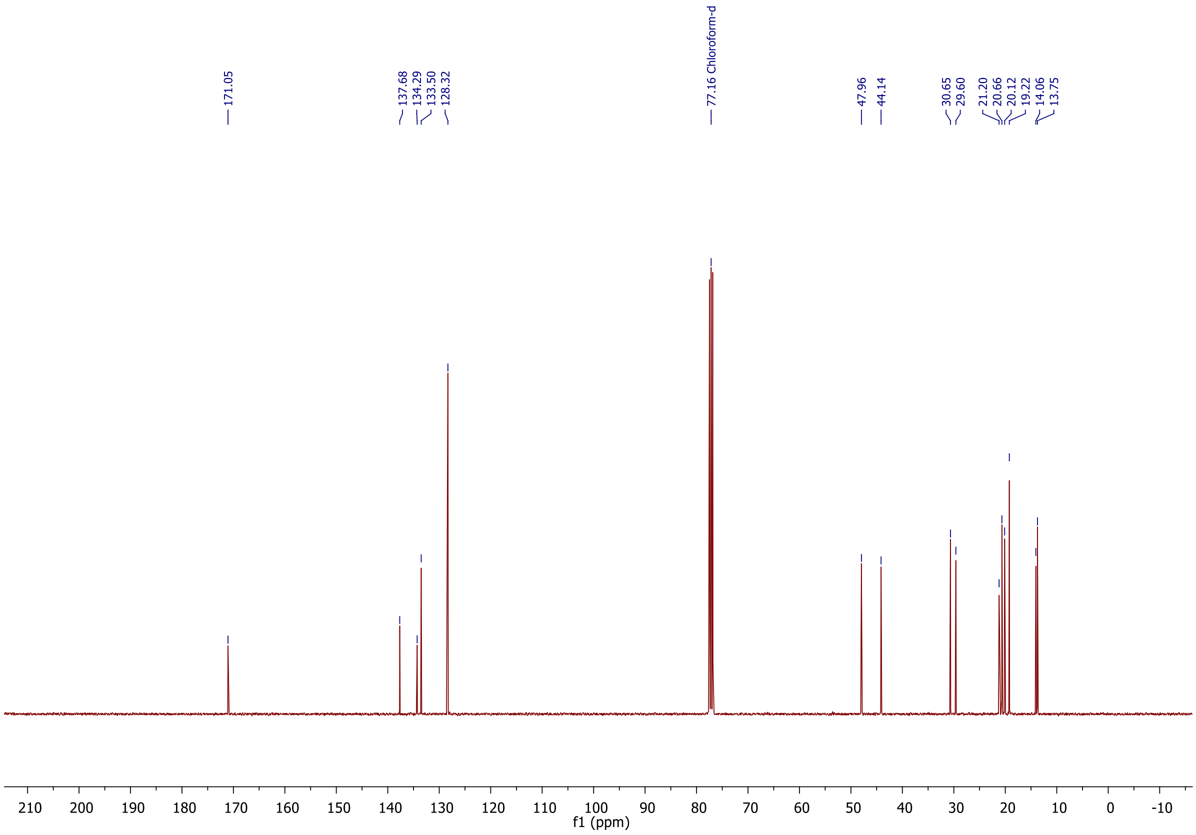

(2-Benzylpiperidin-1-yl)(mesityl)methanone (1k)

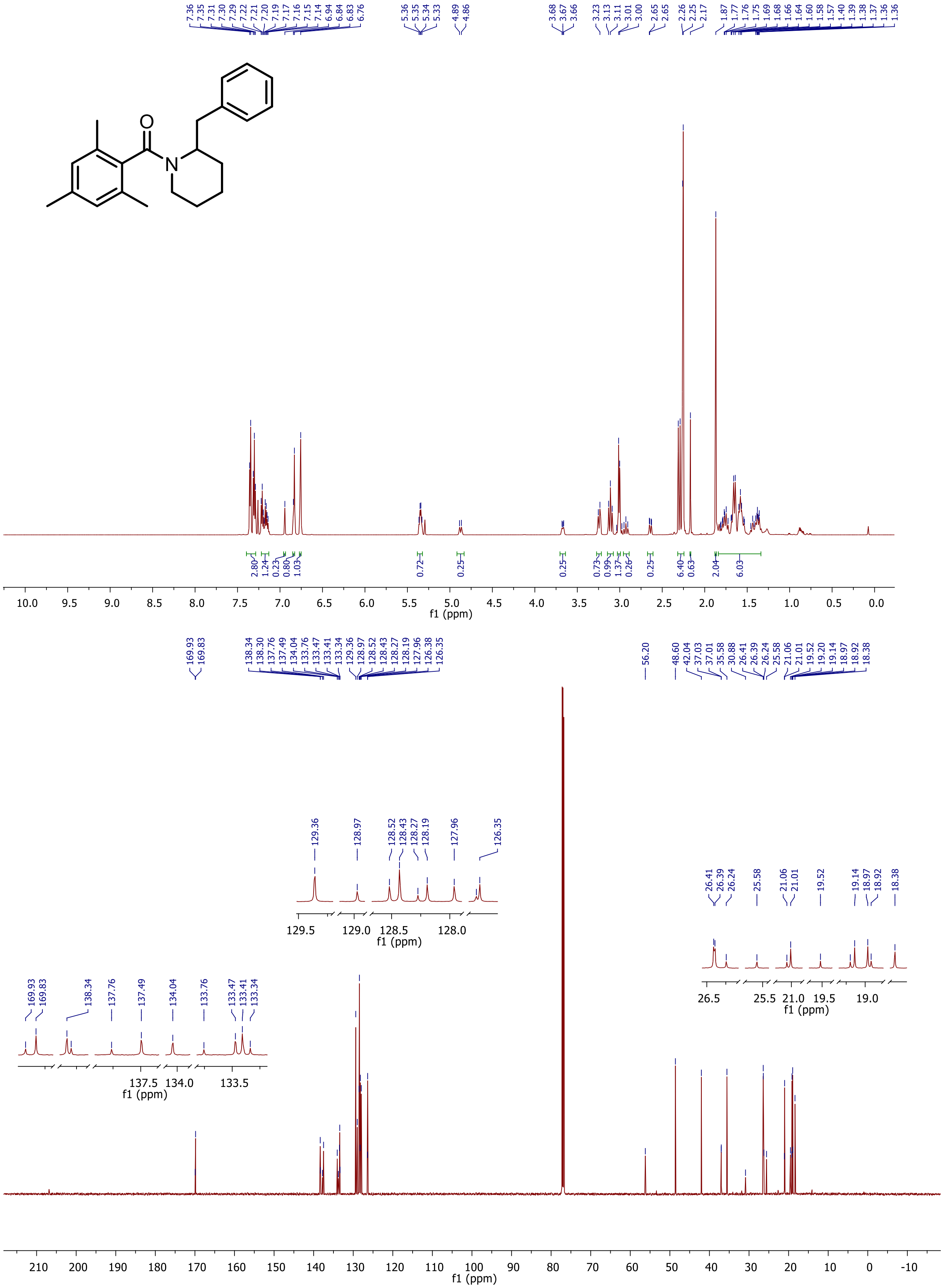

Mesityl(2-methylpiperidin-1-yl)methanone (1l)

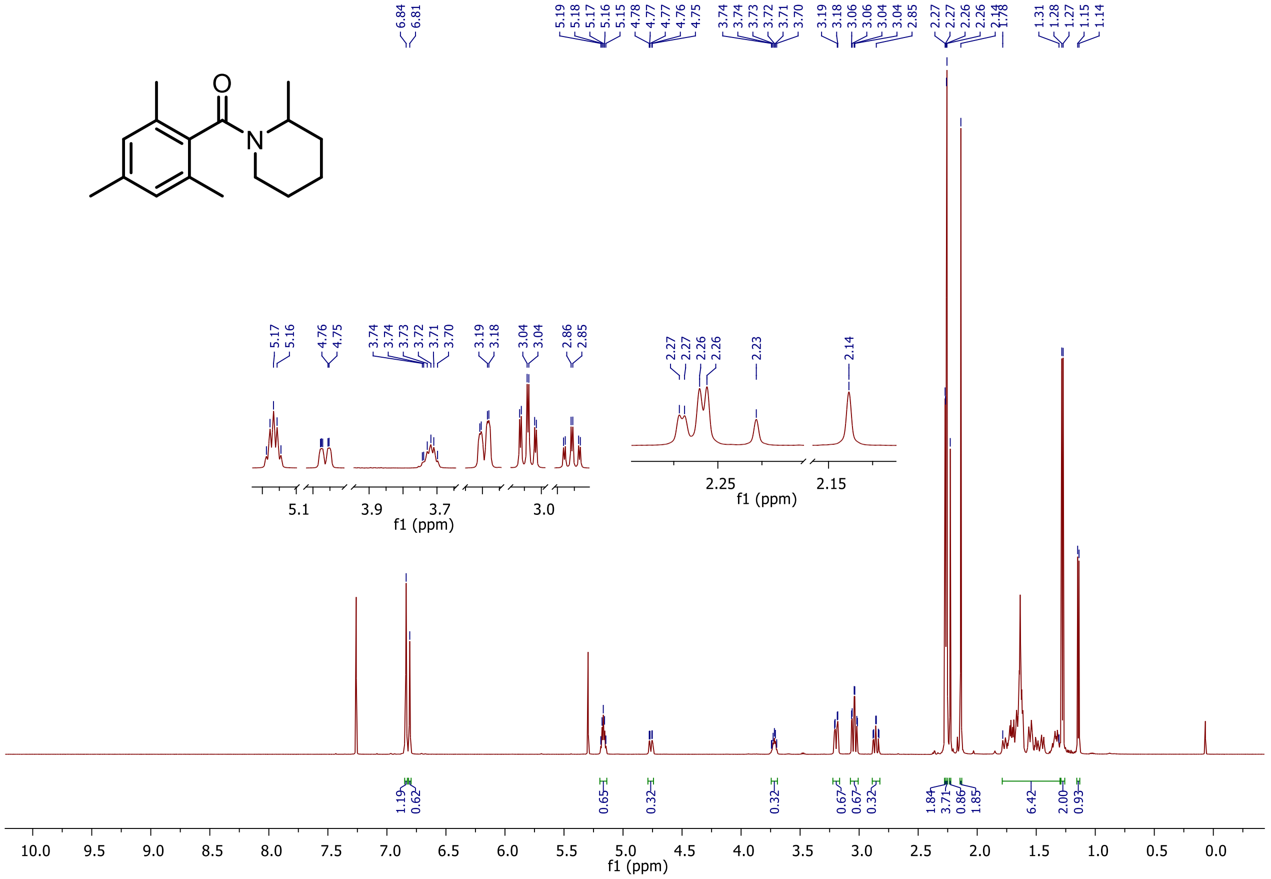

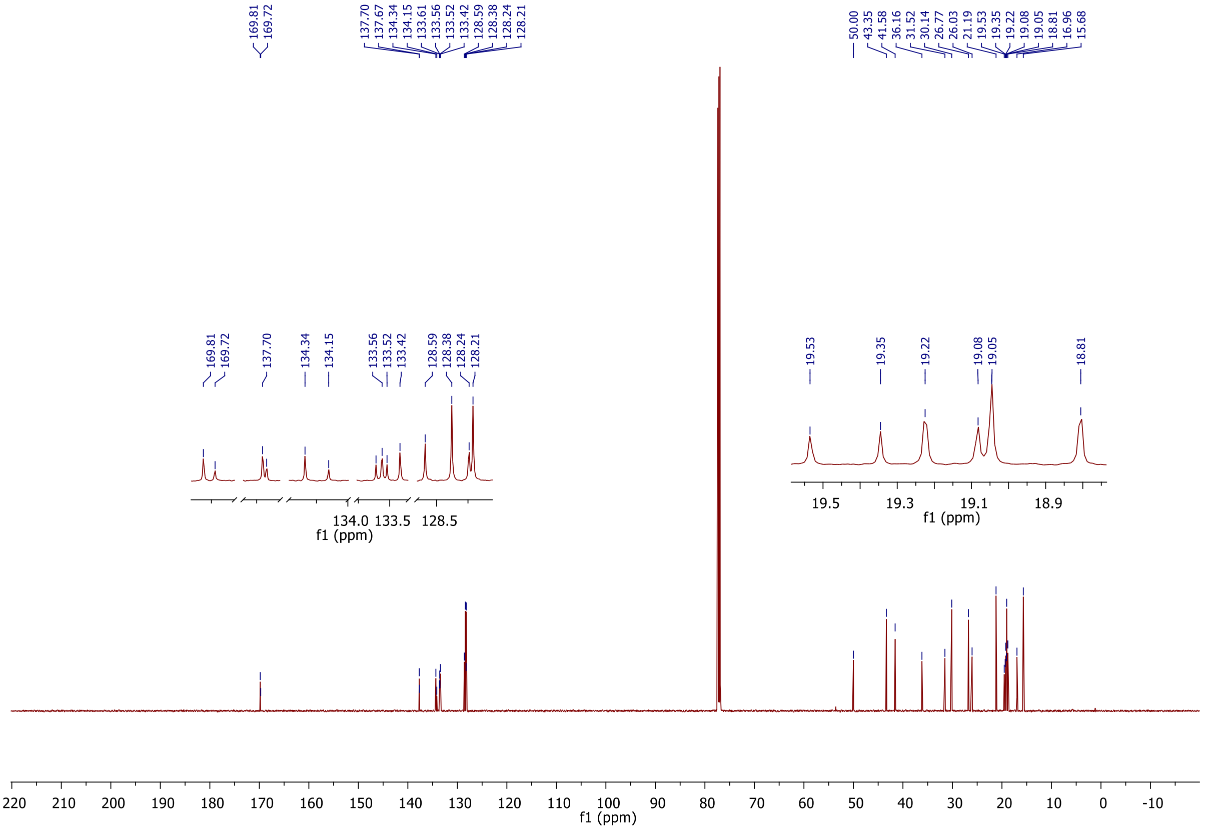

Mesityl(2-phenylpyrrolidin-1-yl)methanone (1m)

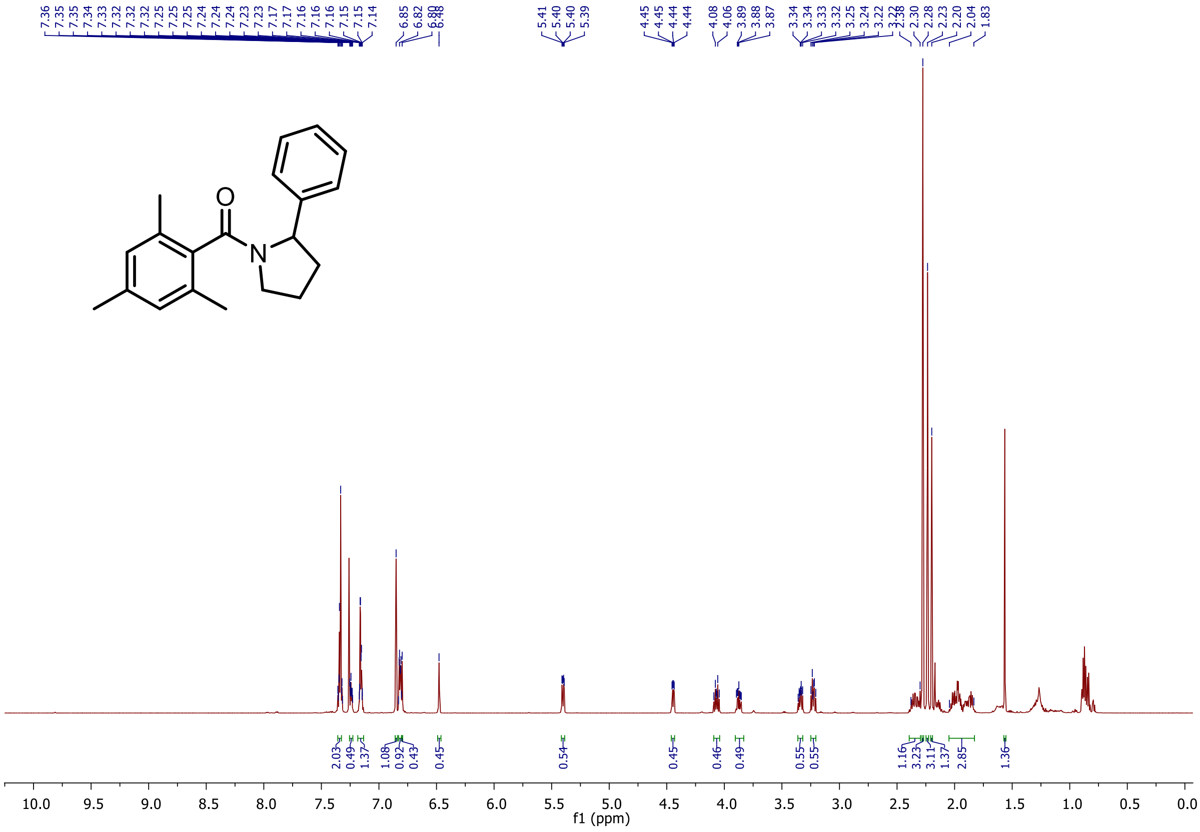

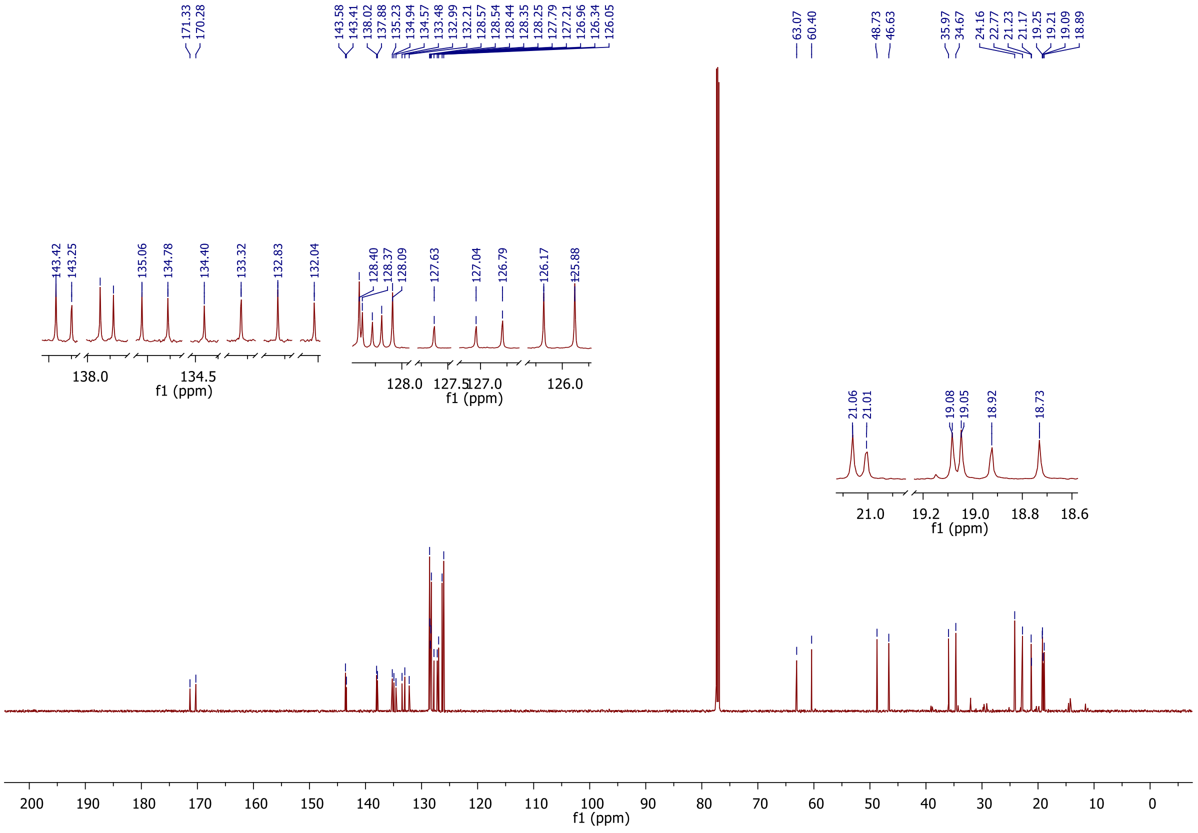

Mesityl((4aS,8aR)-octahydroquinolin-1(2H)-yl)methanone (1n)

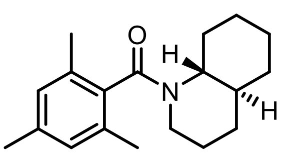

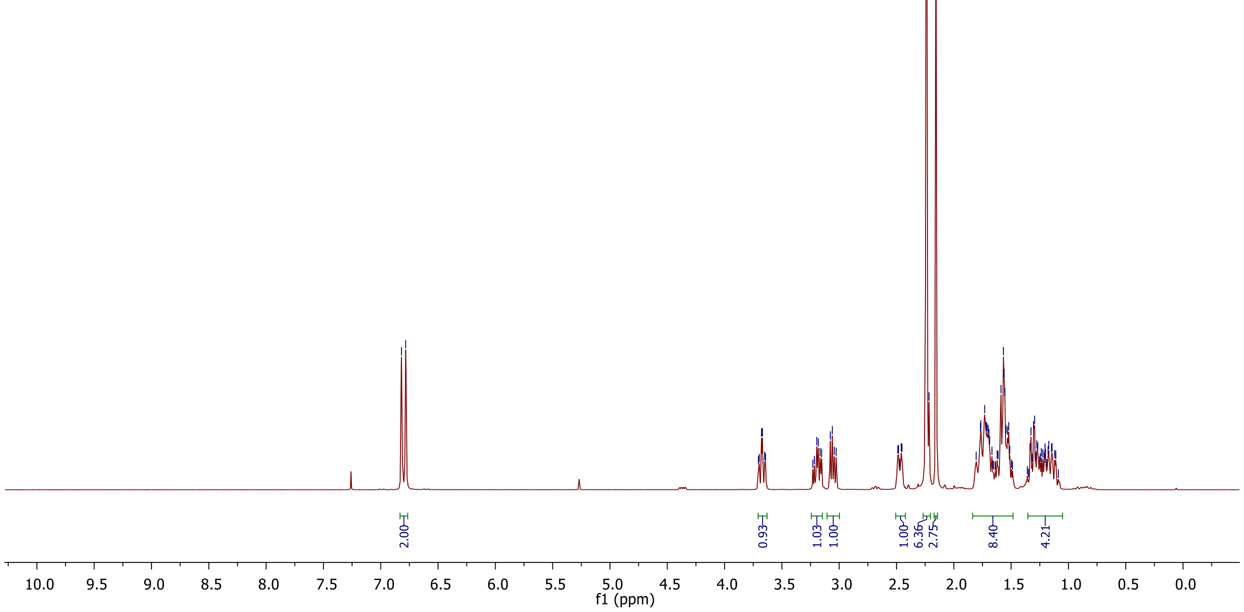

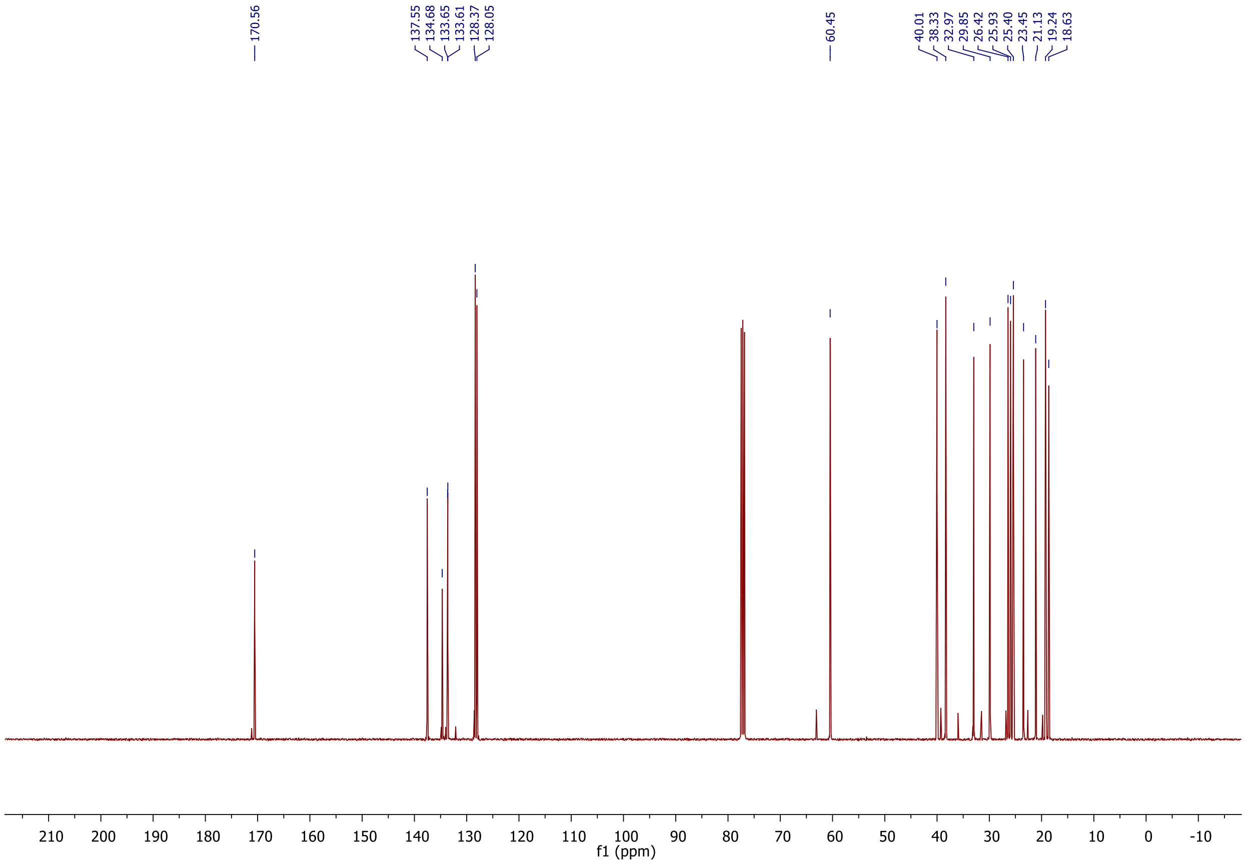

N-Cyclohexyl-N-ethyl-2,4,6-trimethylbenzamide (1o)

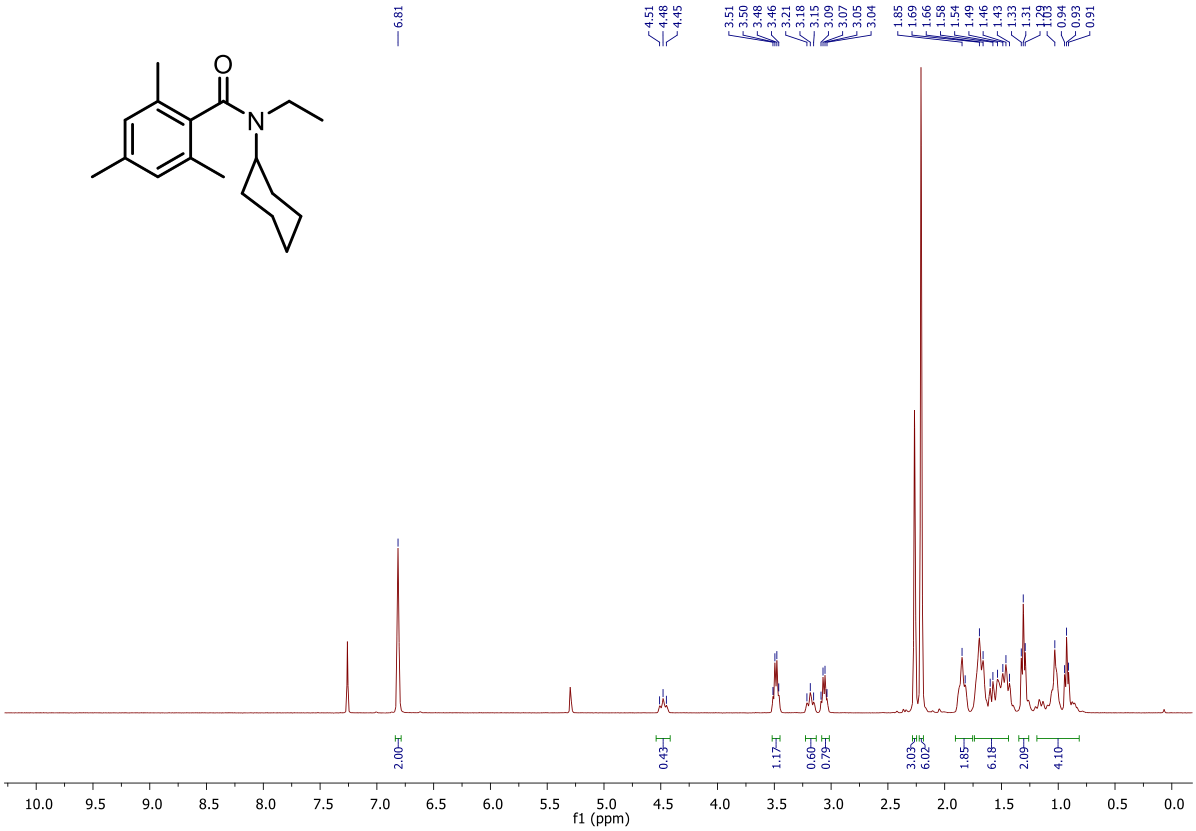

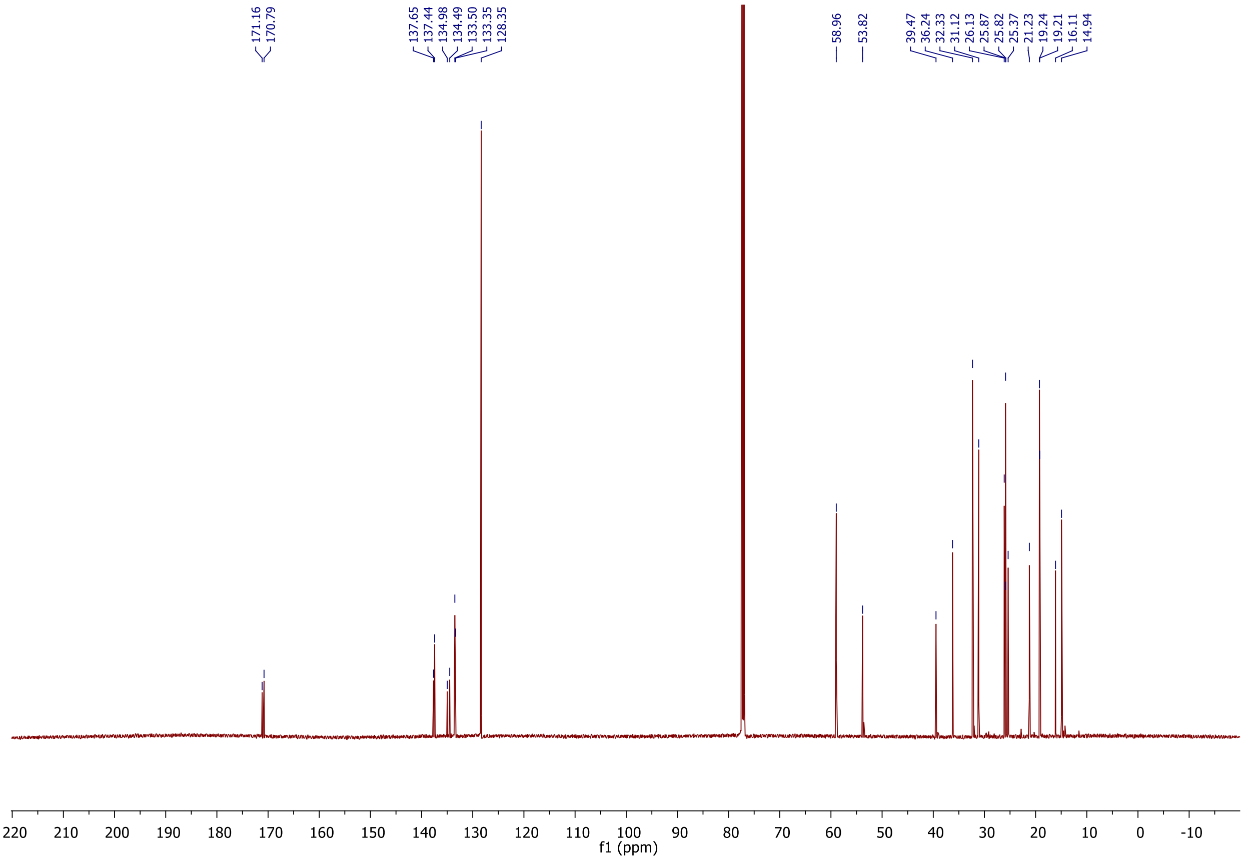

N-Butyl-N-ethyl-2,4,6-trimethylbenzamide (1p)

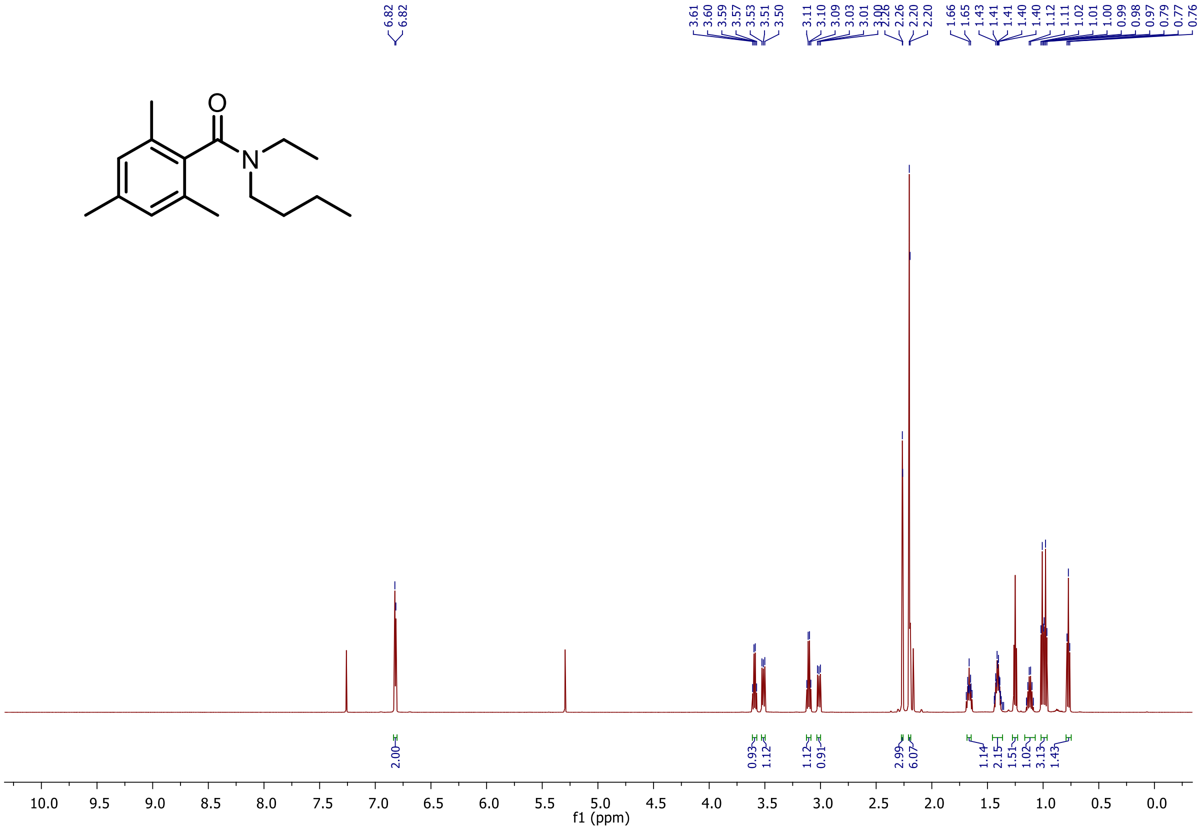

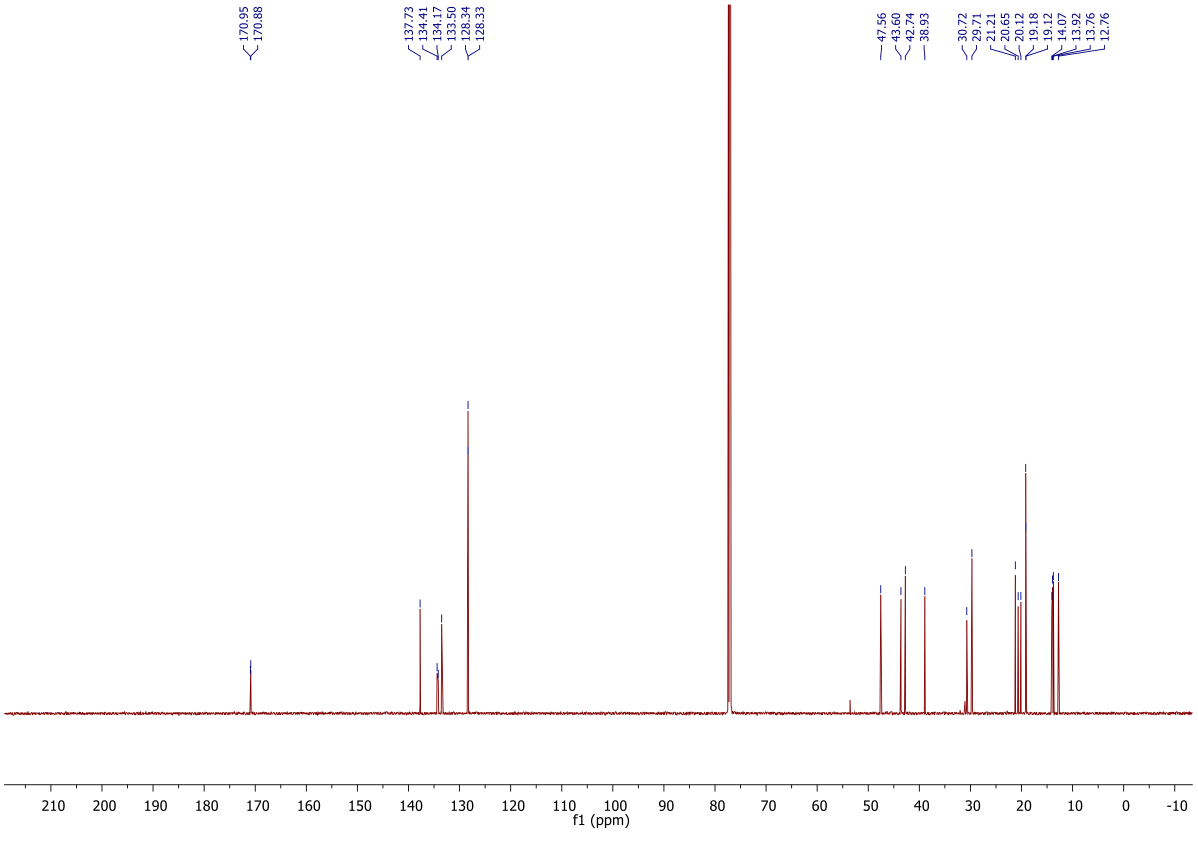

((3S,4R)-3-((Benzo[d][1,3]dioxol-5-yloxy)methyl)-4-(4-fluorophenyl)piperidin-1-yl)(mesityl)methanone (1q)

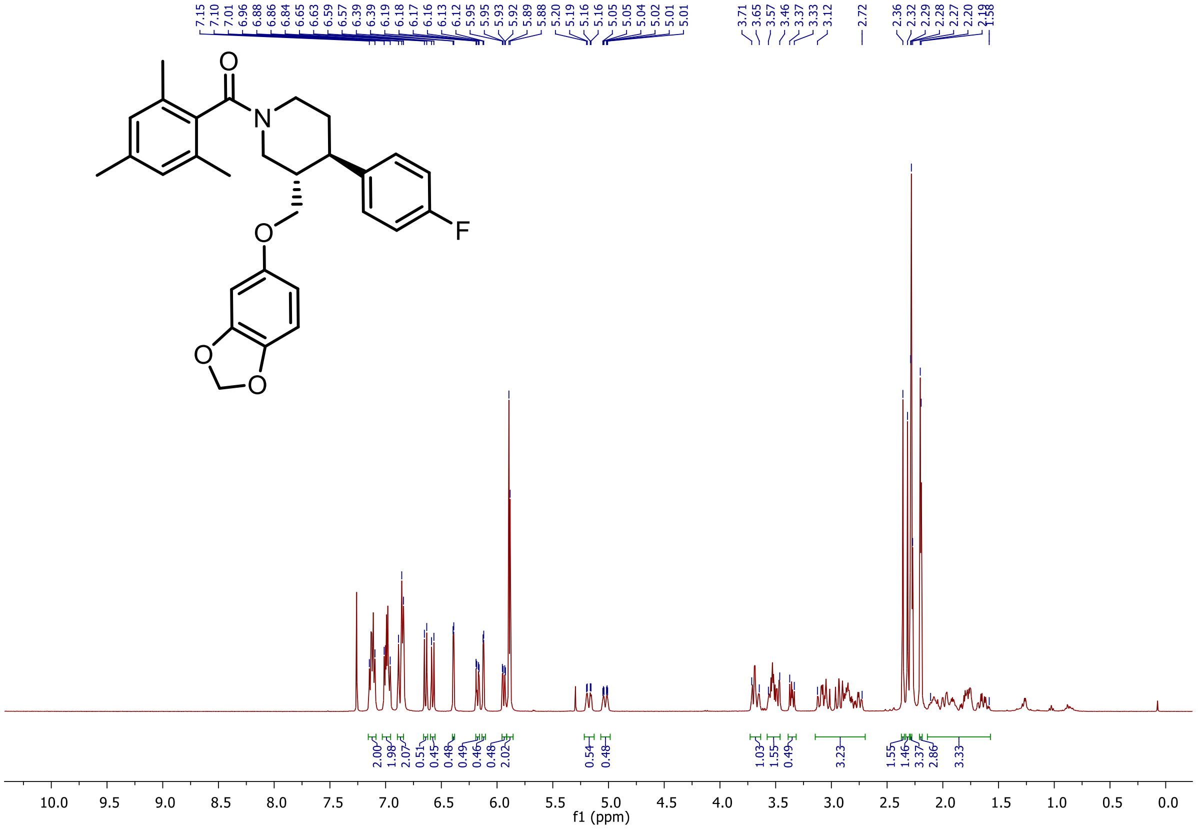

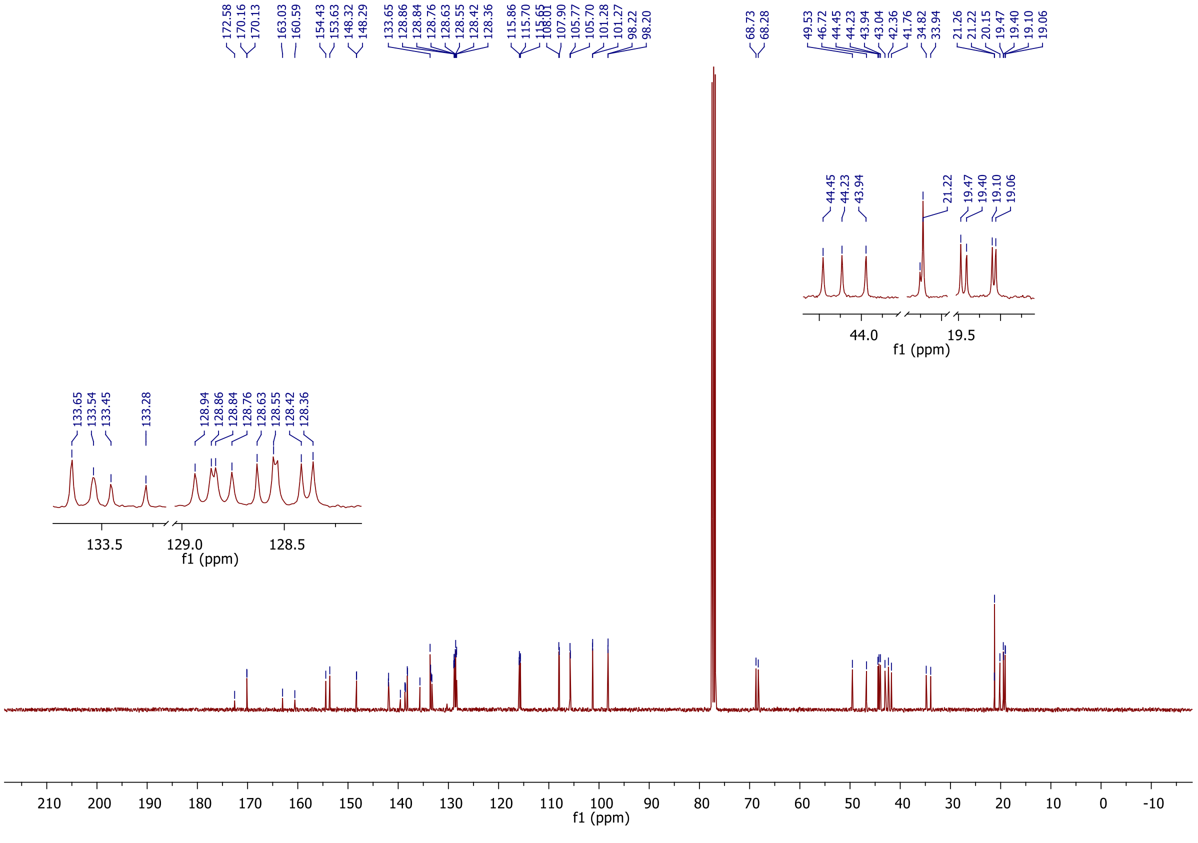

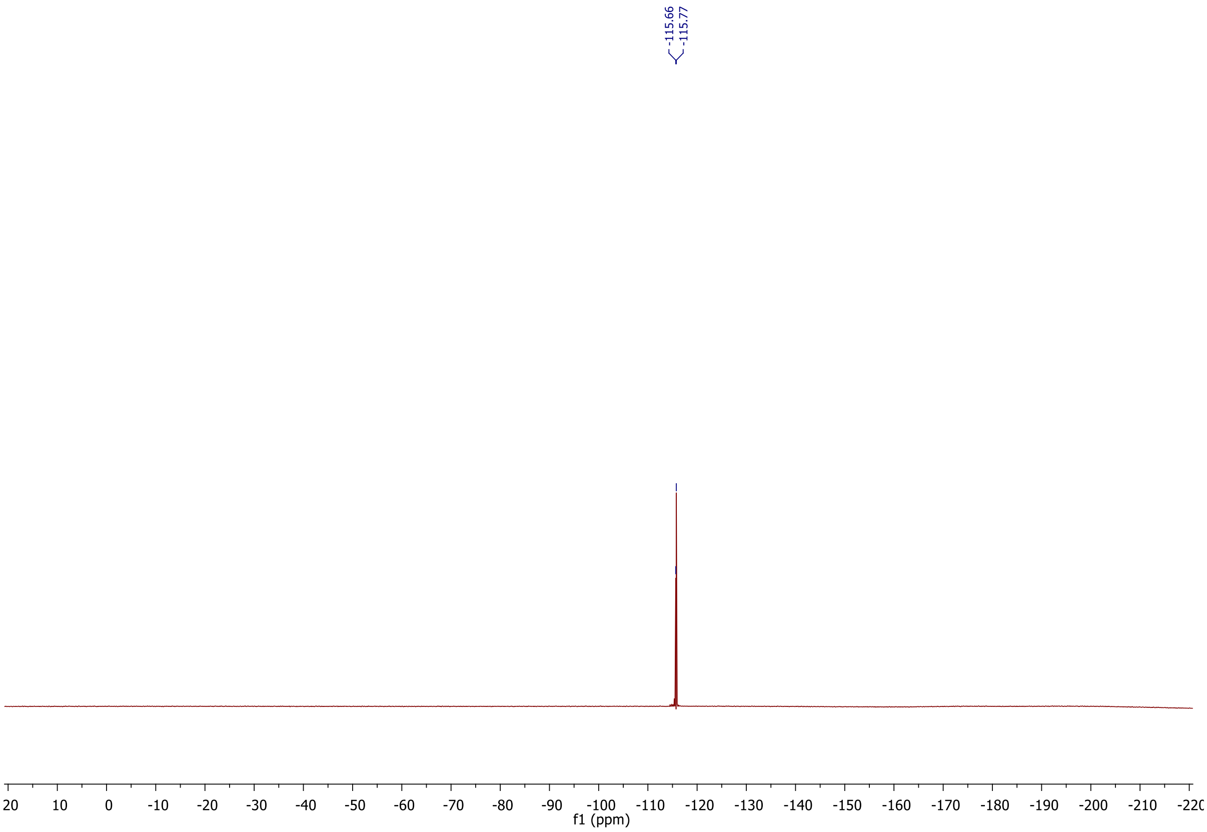
-115.66
-115.77
20 10 0 -10 -20 -30 -40 -50 -60 -70 -80 -90 -100 -110 -120 -130 -140 -150 -160 -170 -180 -190 -200 -210 -220
f1 (ppm)

(1-(3,4-Dimethoxybenzyl)-6,7-dimethoxy-3,4-dihydroisoquinolin-2(1H)-yl)(mesityl)methanone (1r)

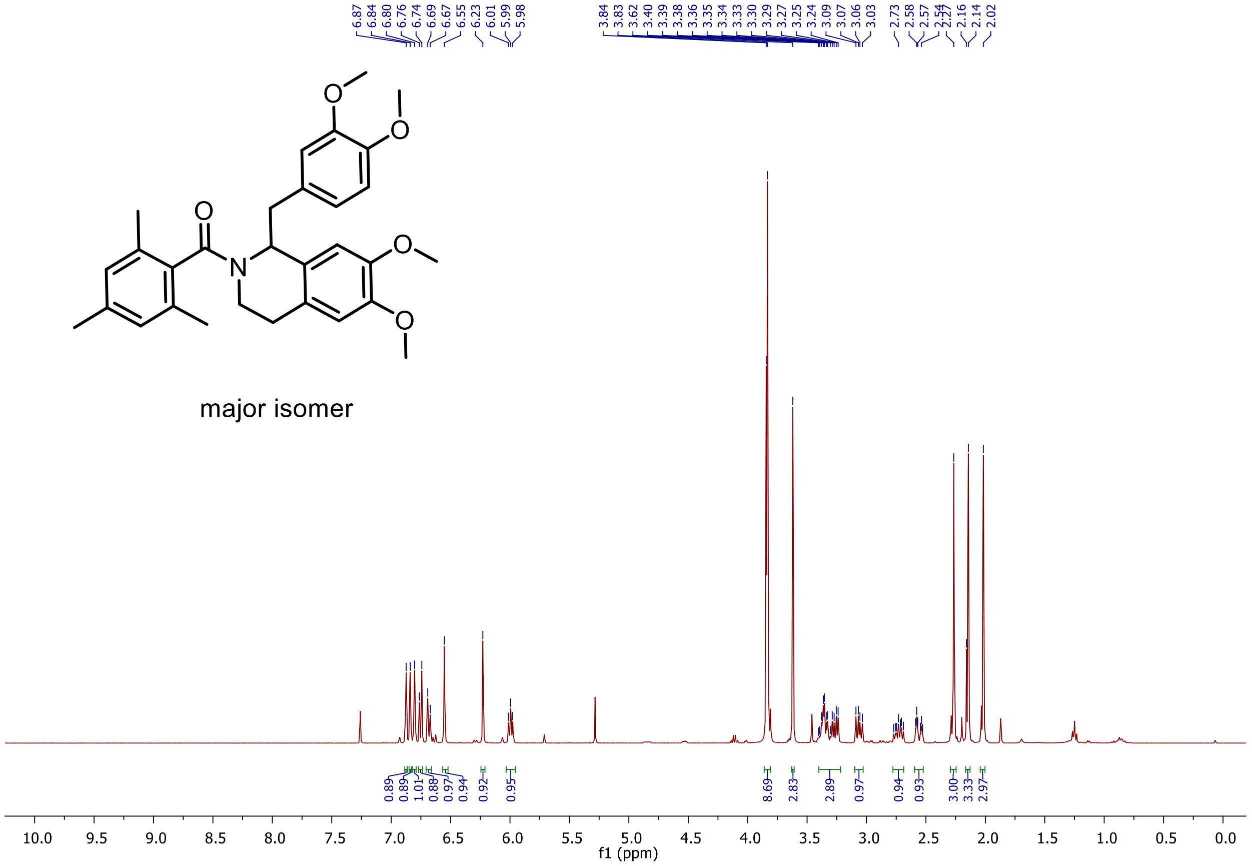

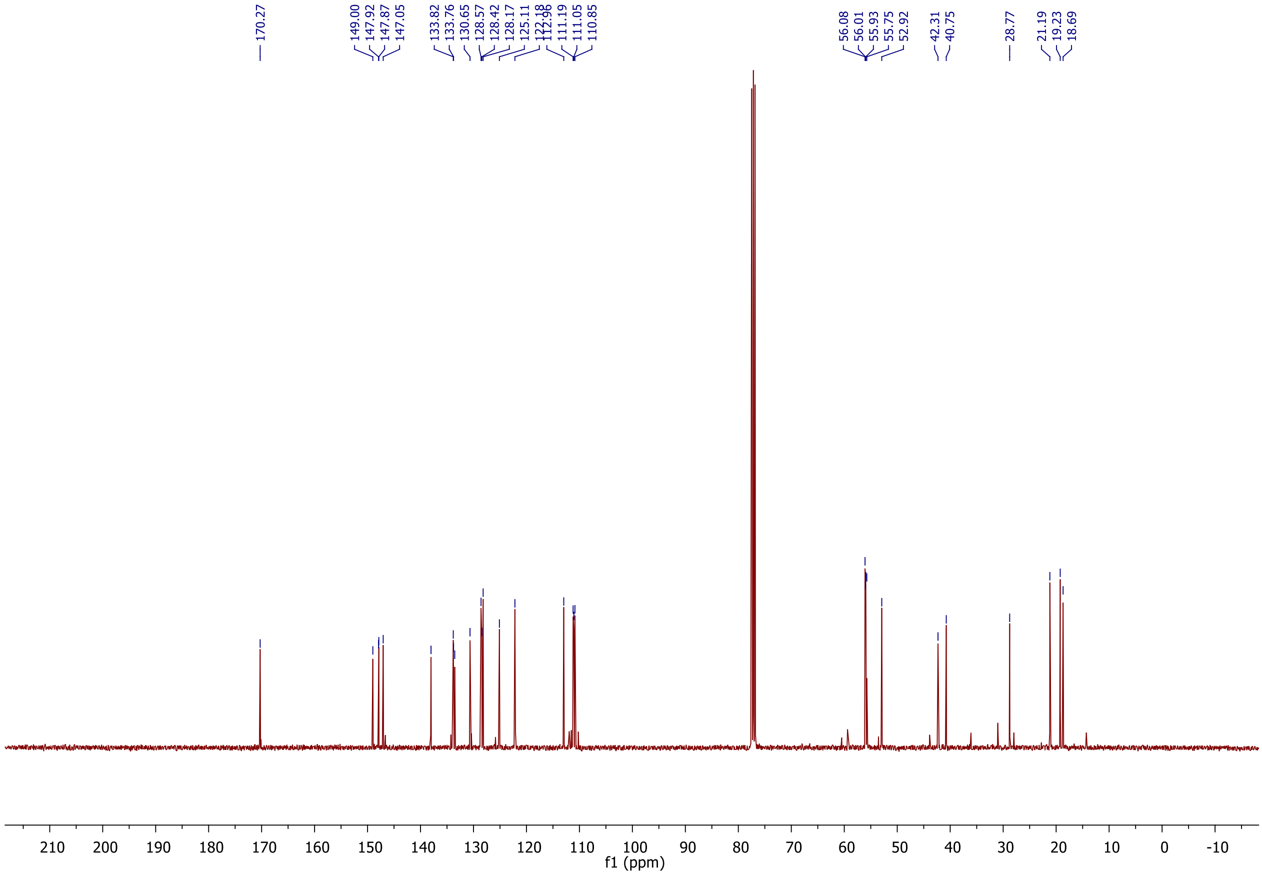

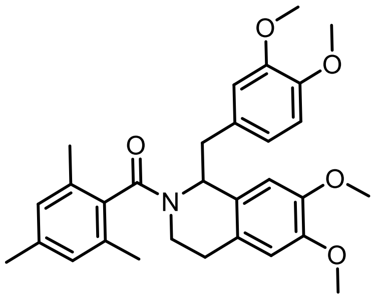

minor isomer

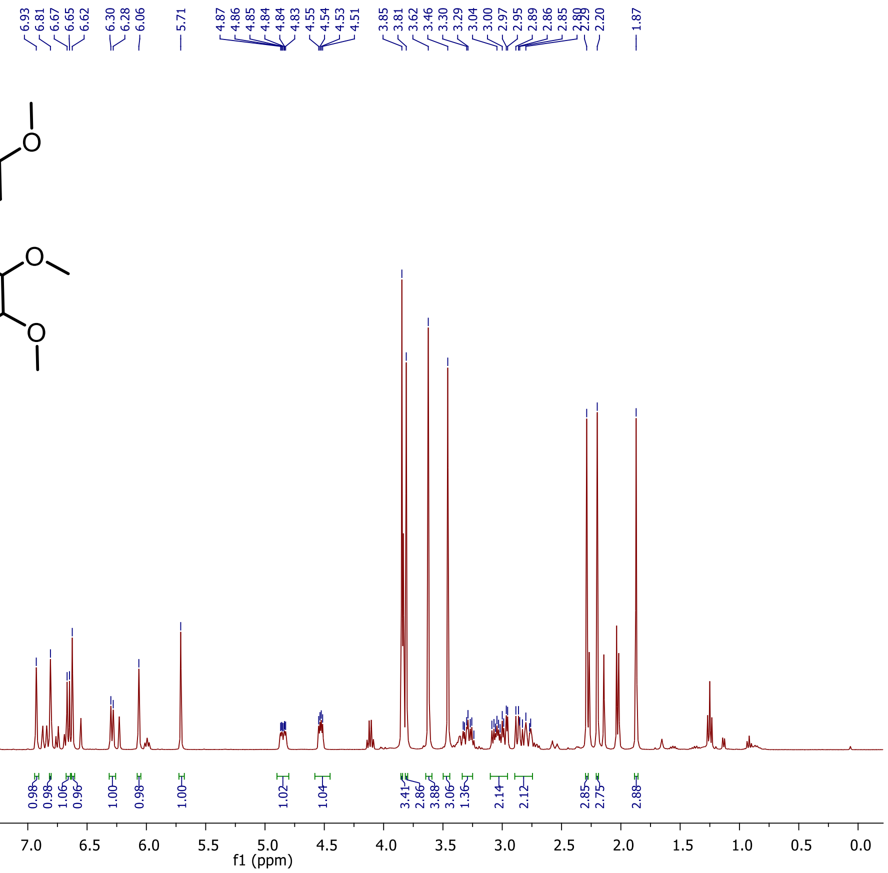

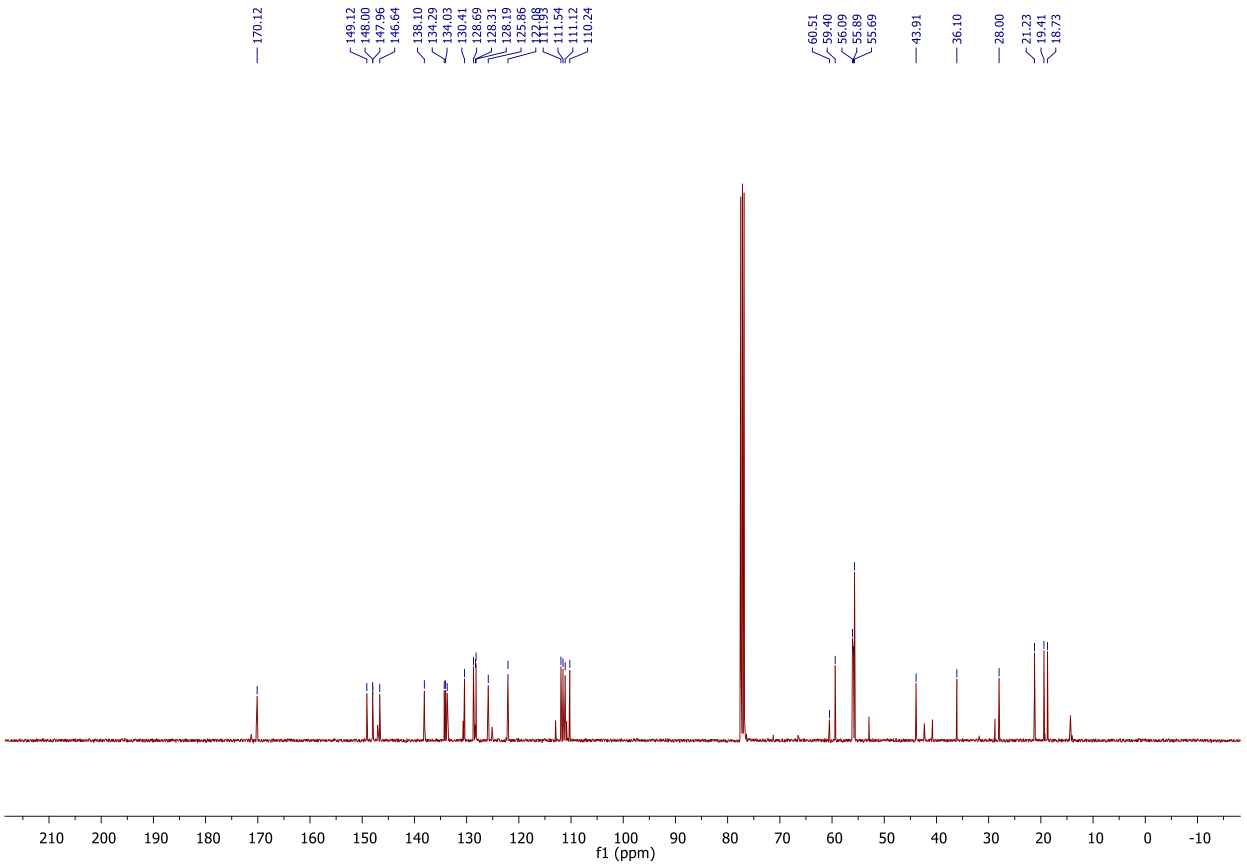

Pyrrolidin-1-yl(2,4,6-triisopropylphenyl)methanone (3a)

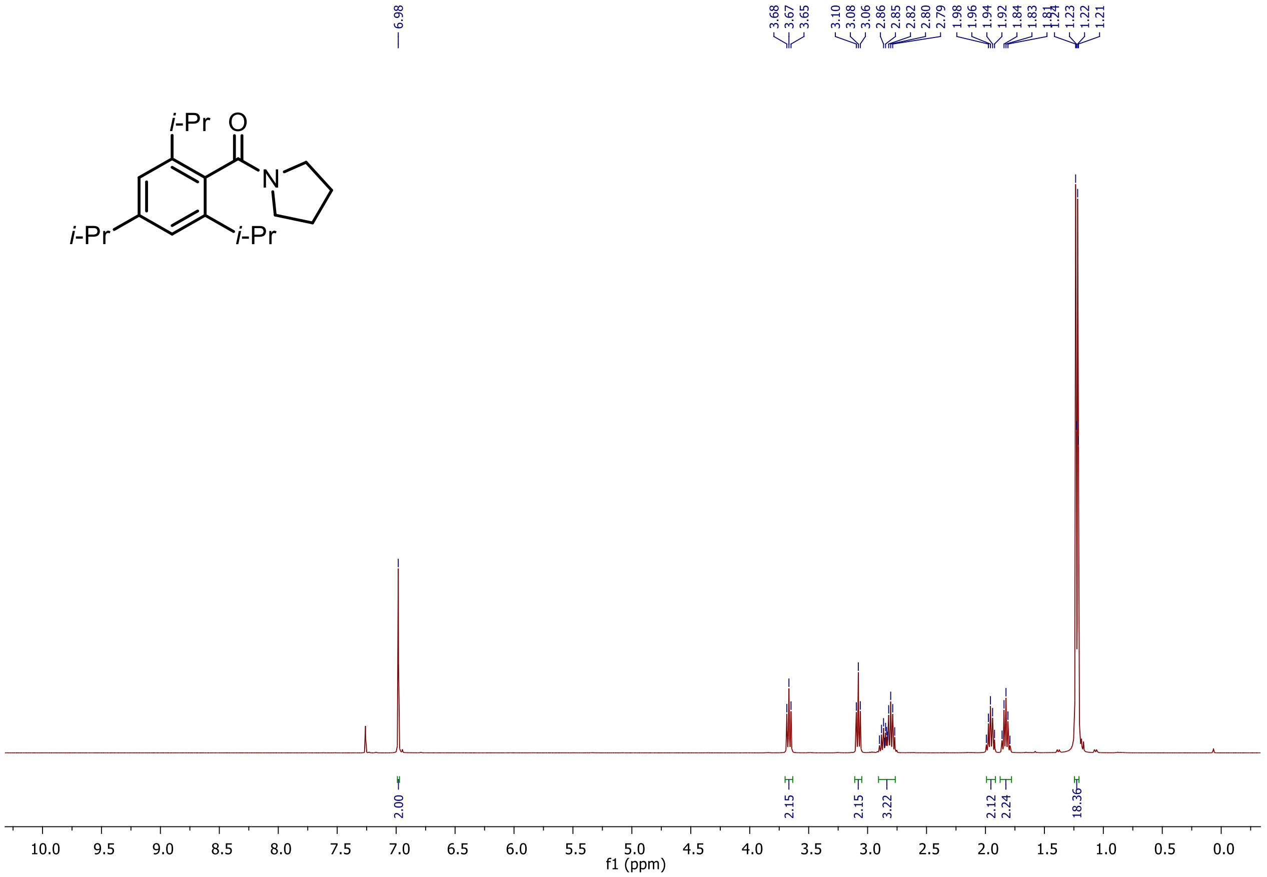

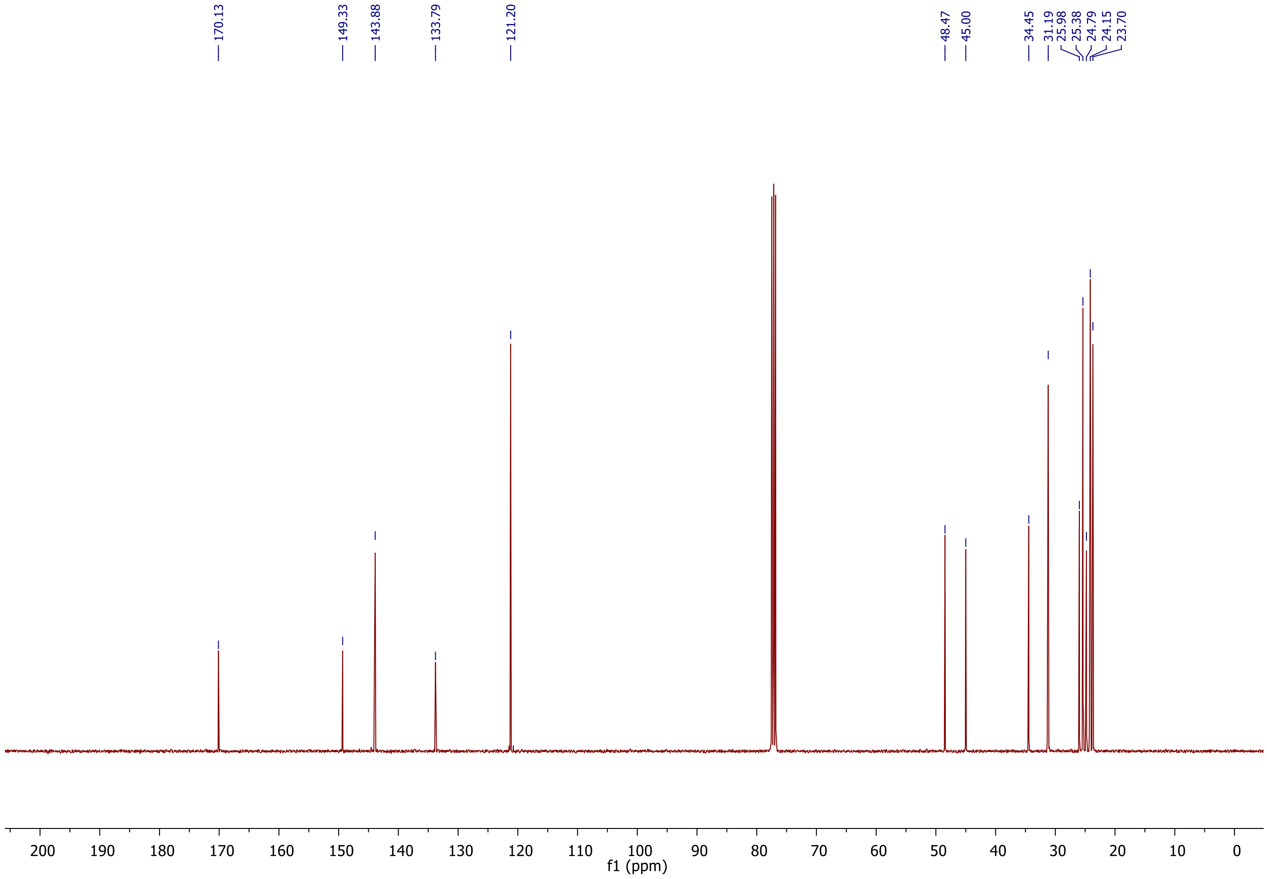

Pyrrolidin-1-yl(3,4,5-trimethoxyphenyl)methanone (3j)

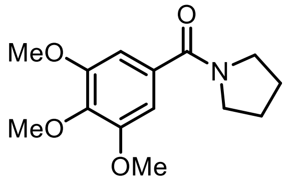

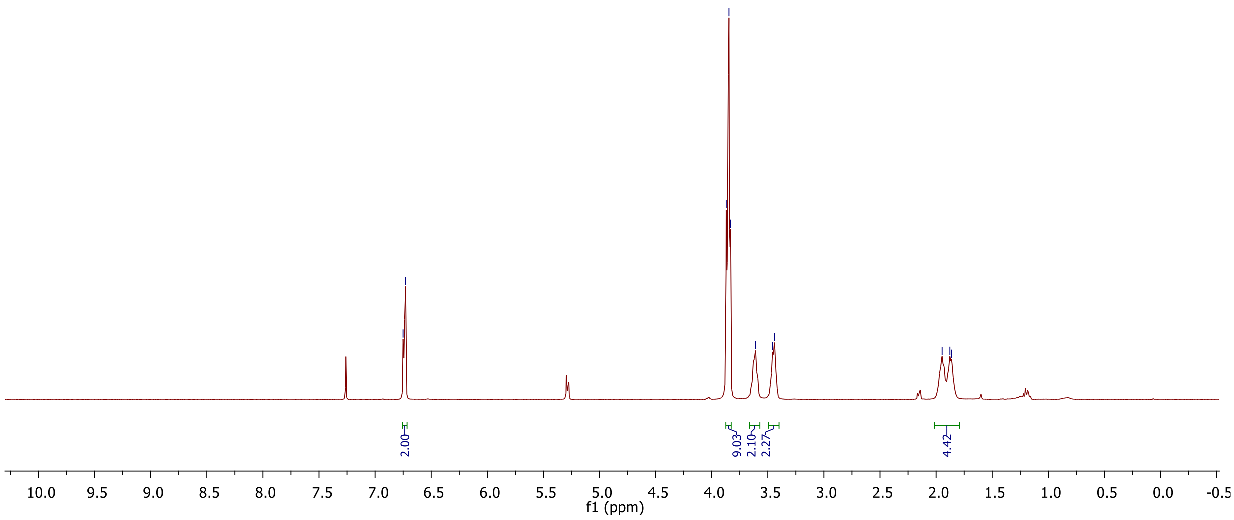

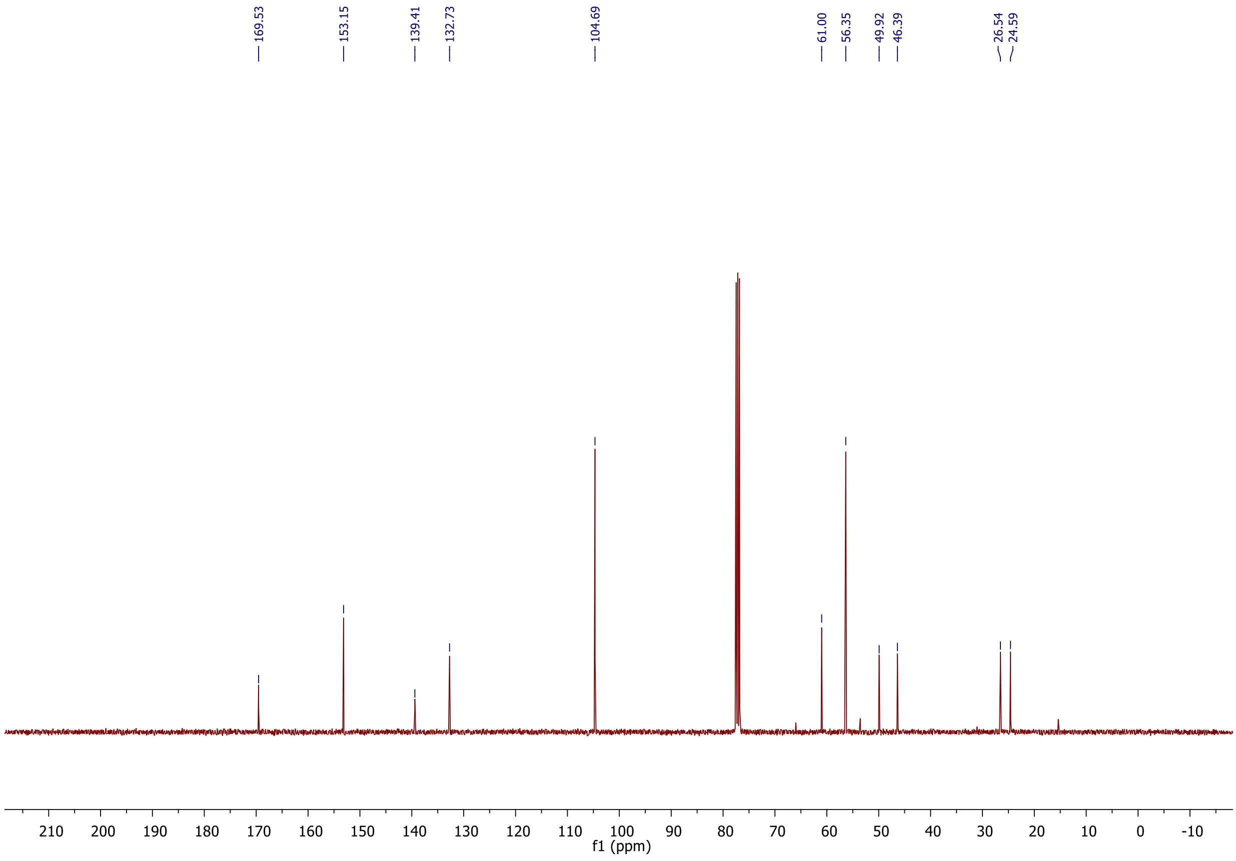

(2-Bromo-5-methoxyphenyl)(pyrrolidin-1-yl)methanone (3m)

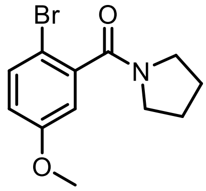

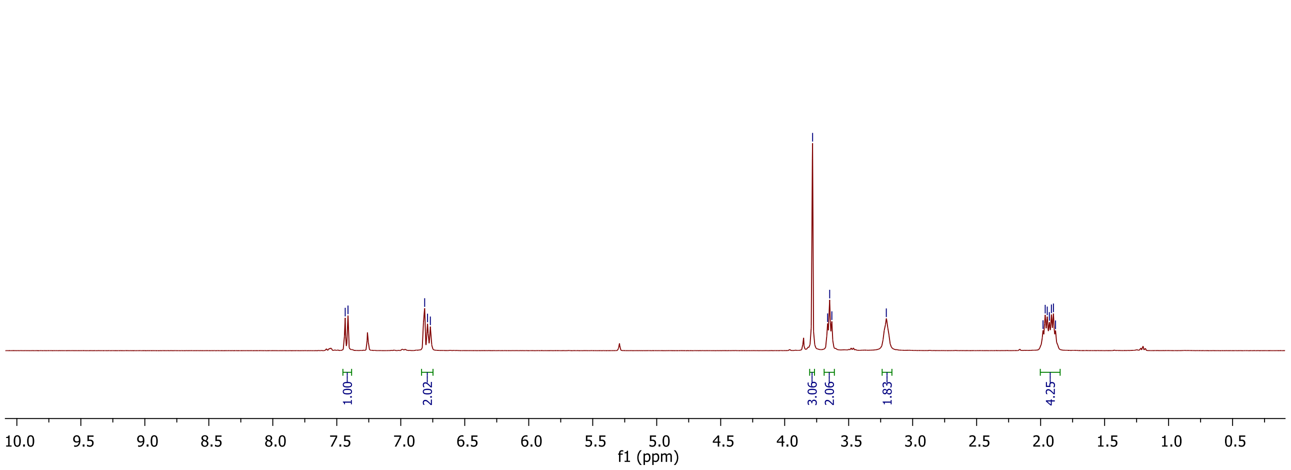

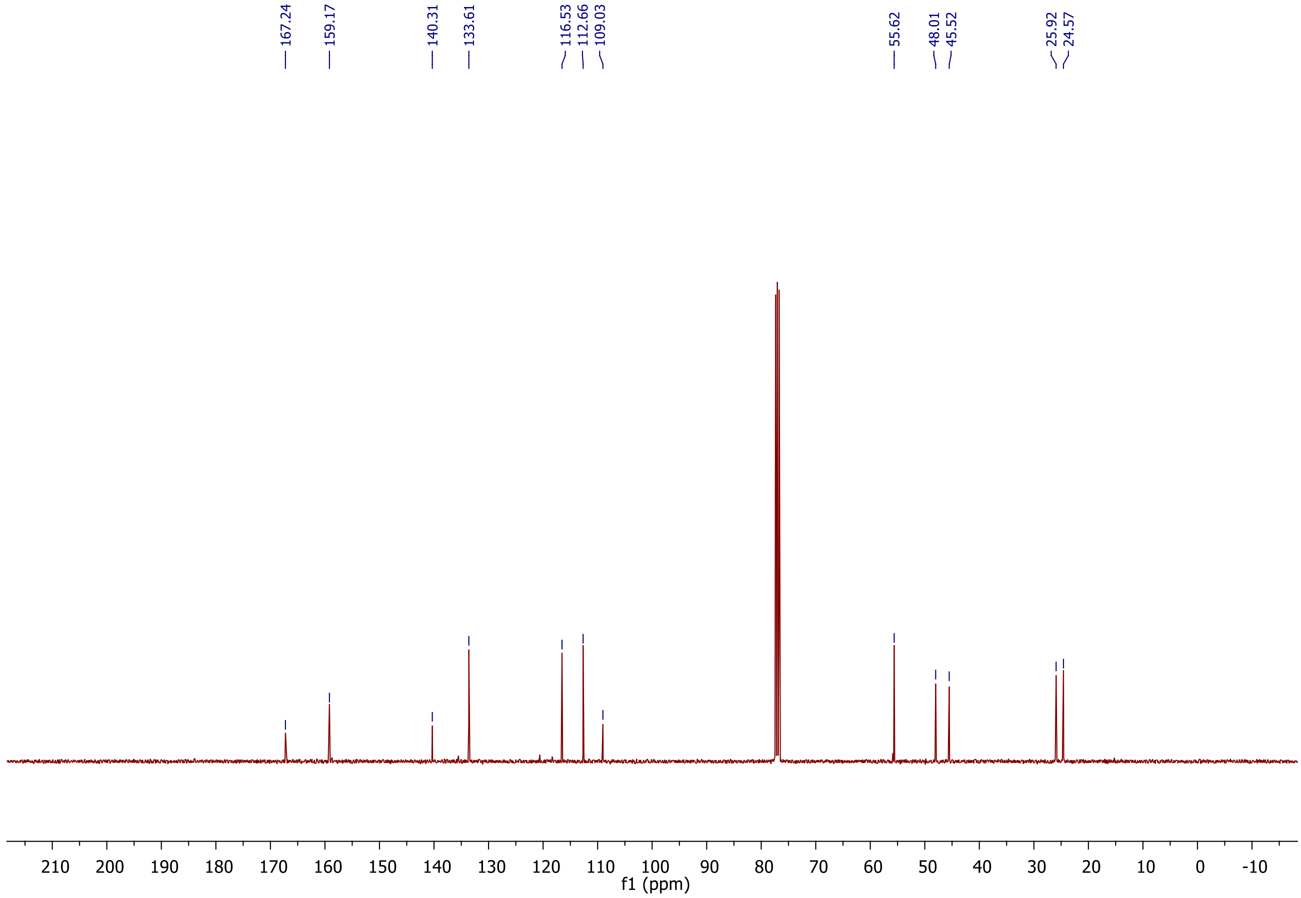

(3-Chlorophenyl)(pyrrolidin-1-yl)methanone (3n)

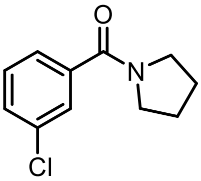

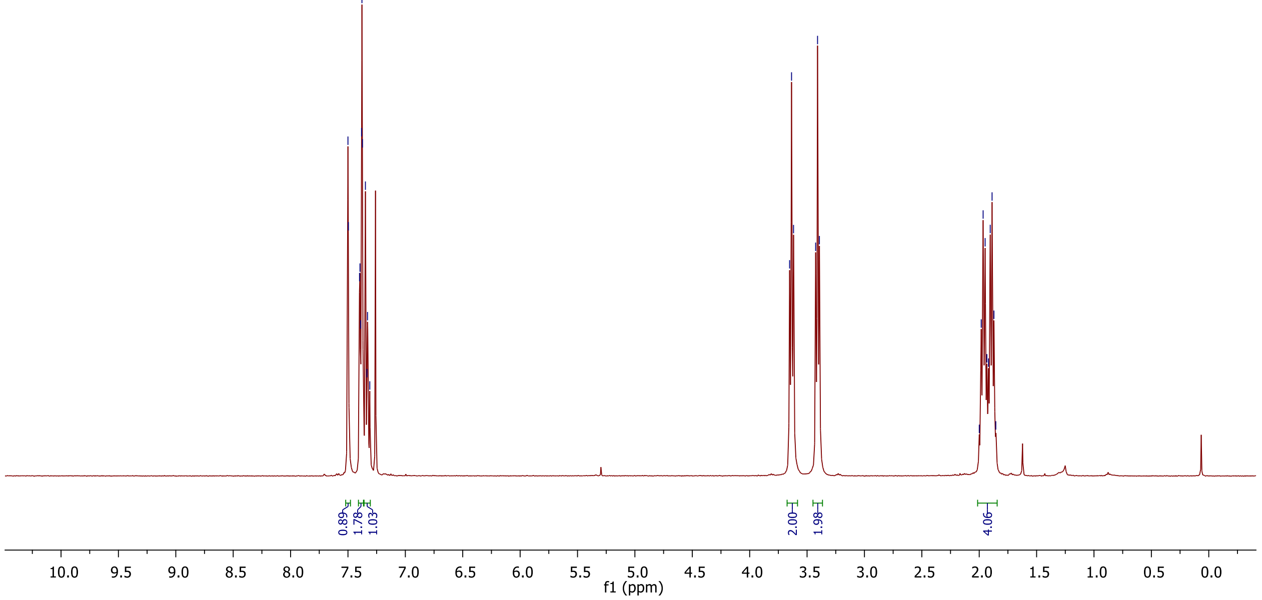

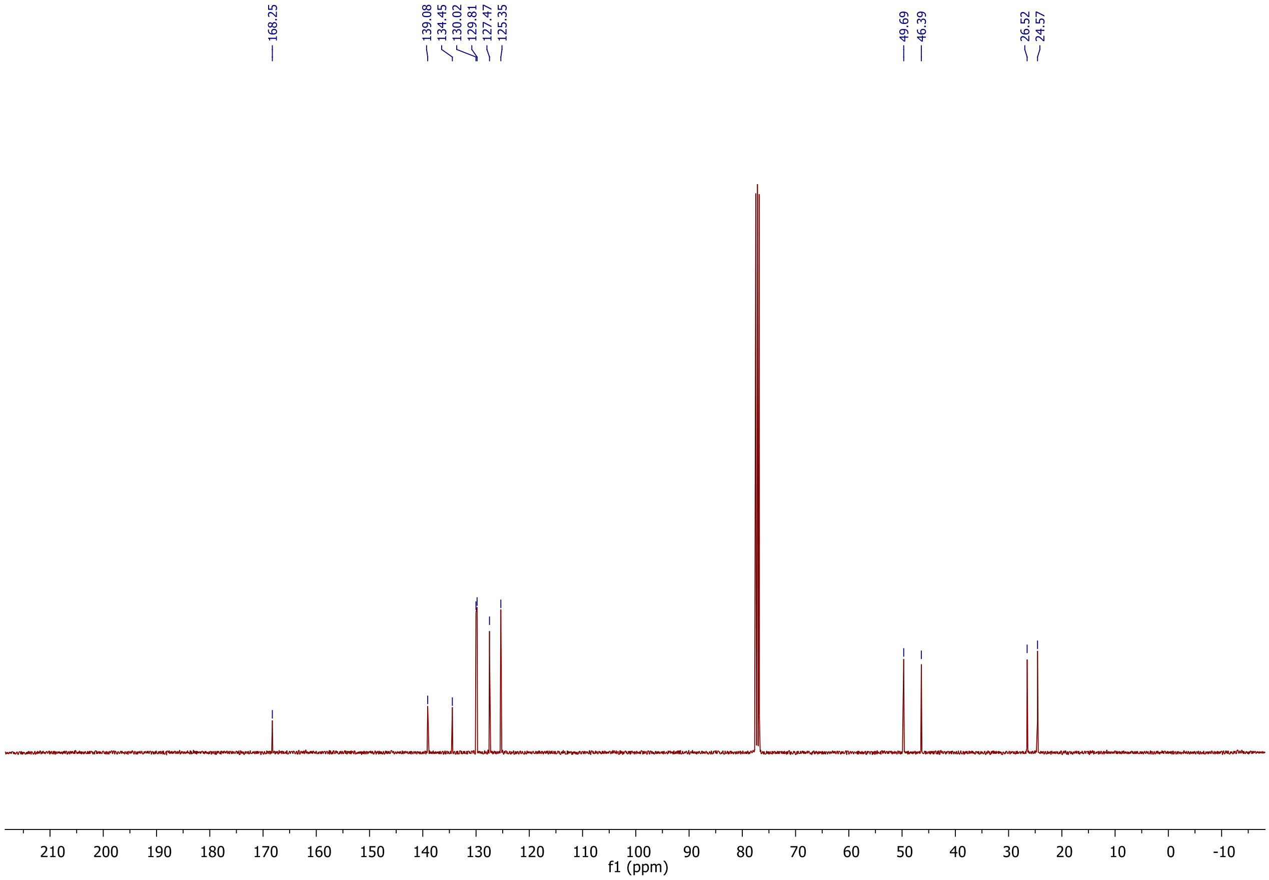

(2,3-Dihydro-1H-pyrrol-1-yl)(4-vinylphenyl)methanone (3p)

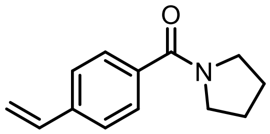

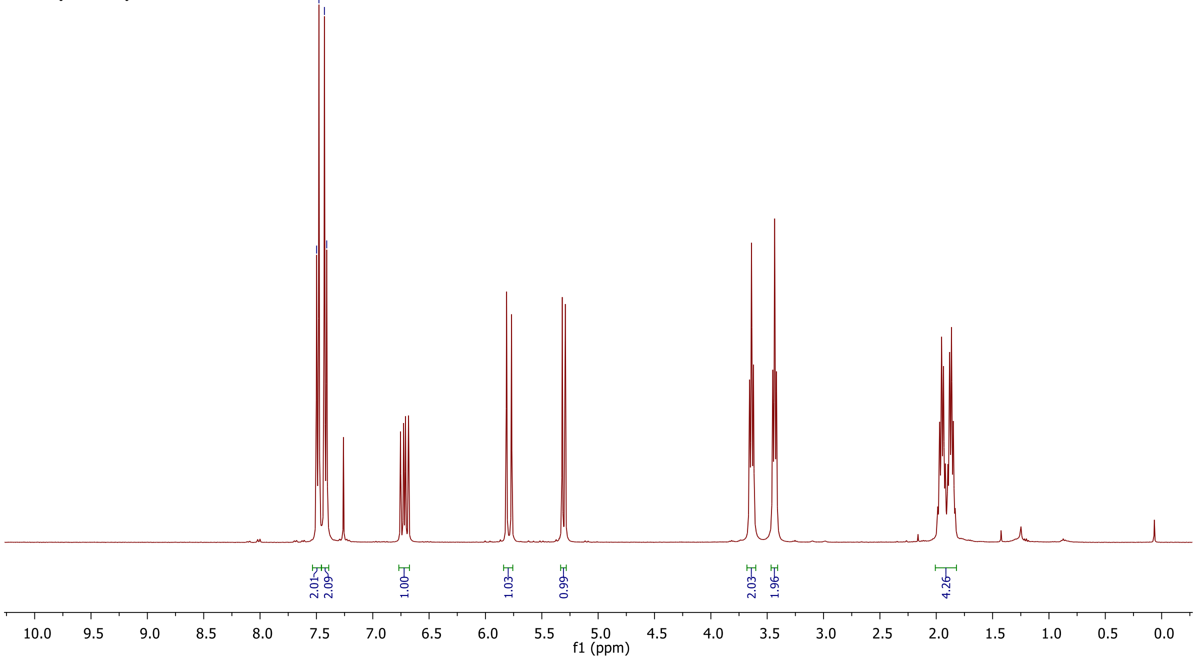

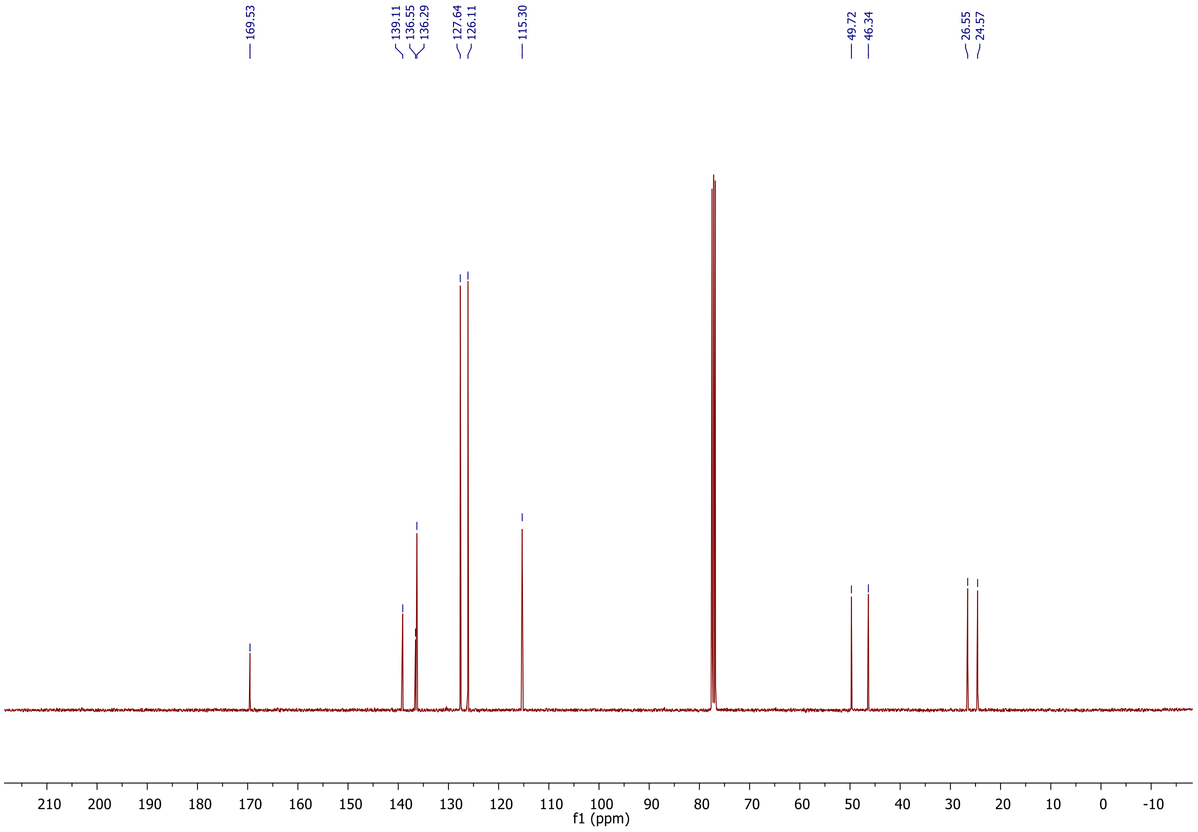

(4-(Methylthio)phenyl)(piperidin-1-yl)methanone (3q)

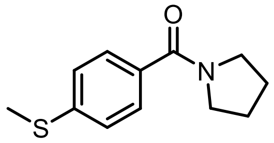

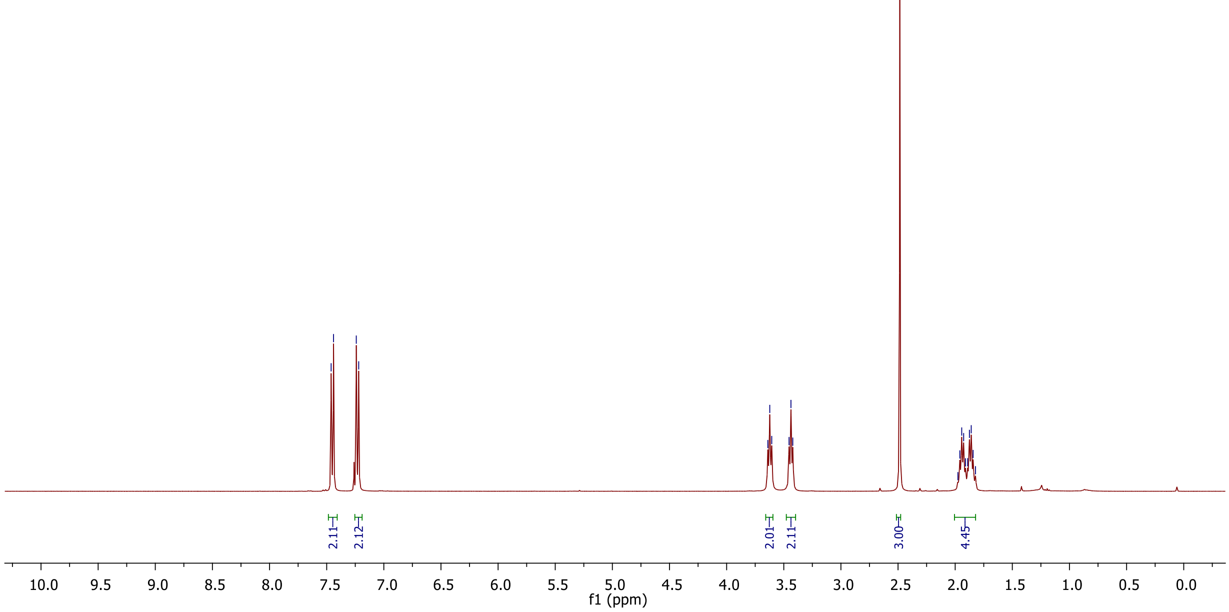

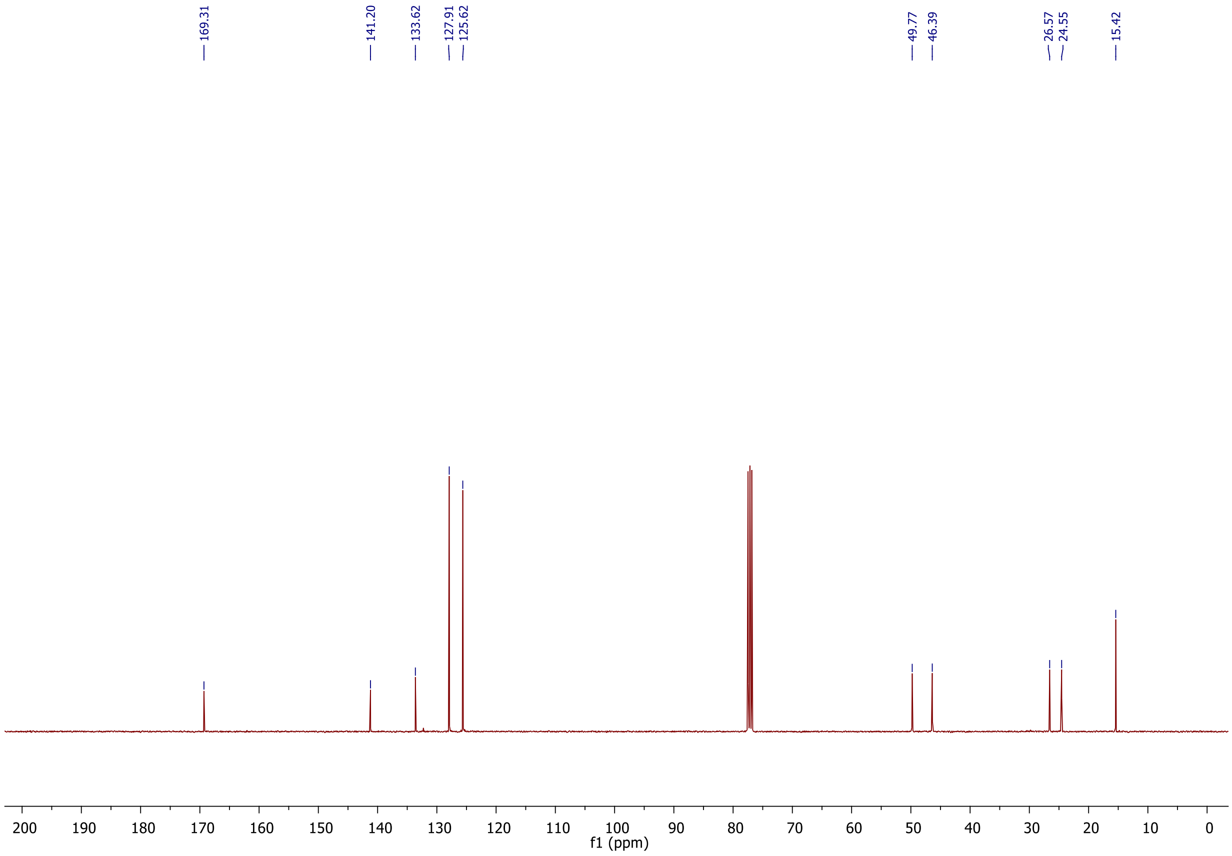

3-(Pyrrolidine-1-carbonyl)benzonitrile (3r)

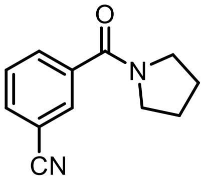

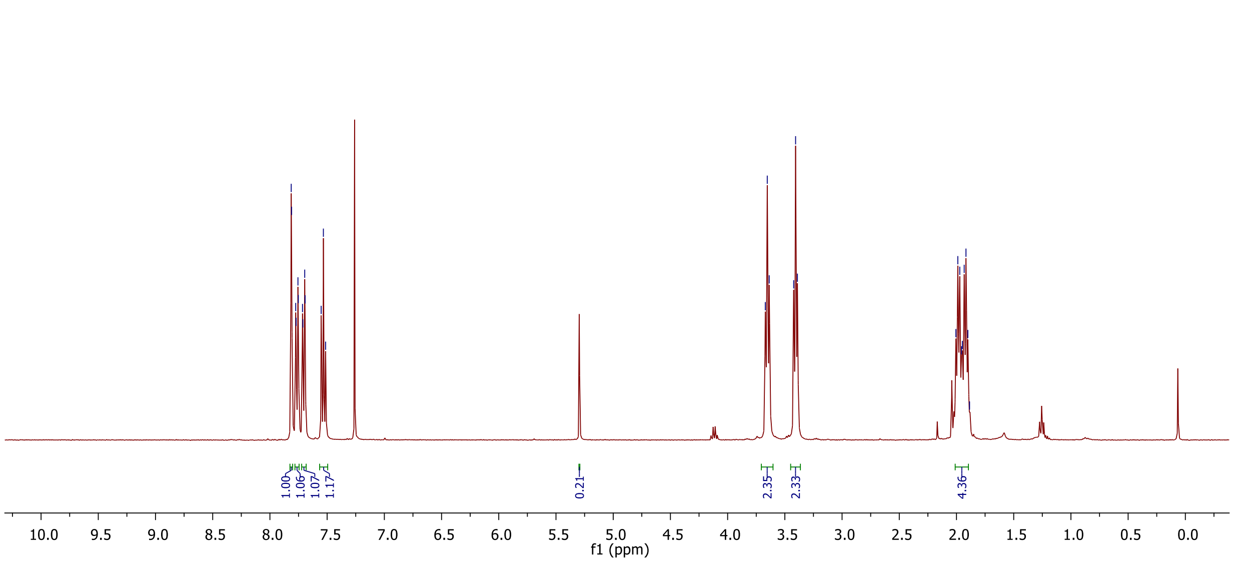

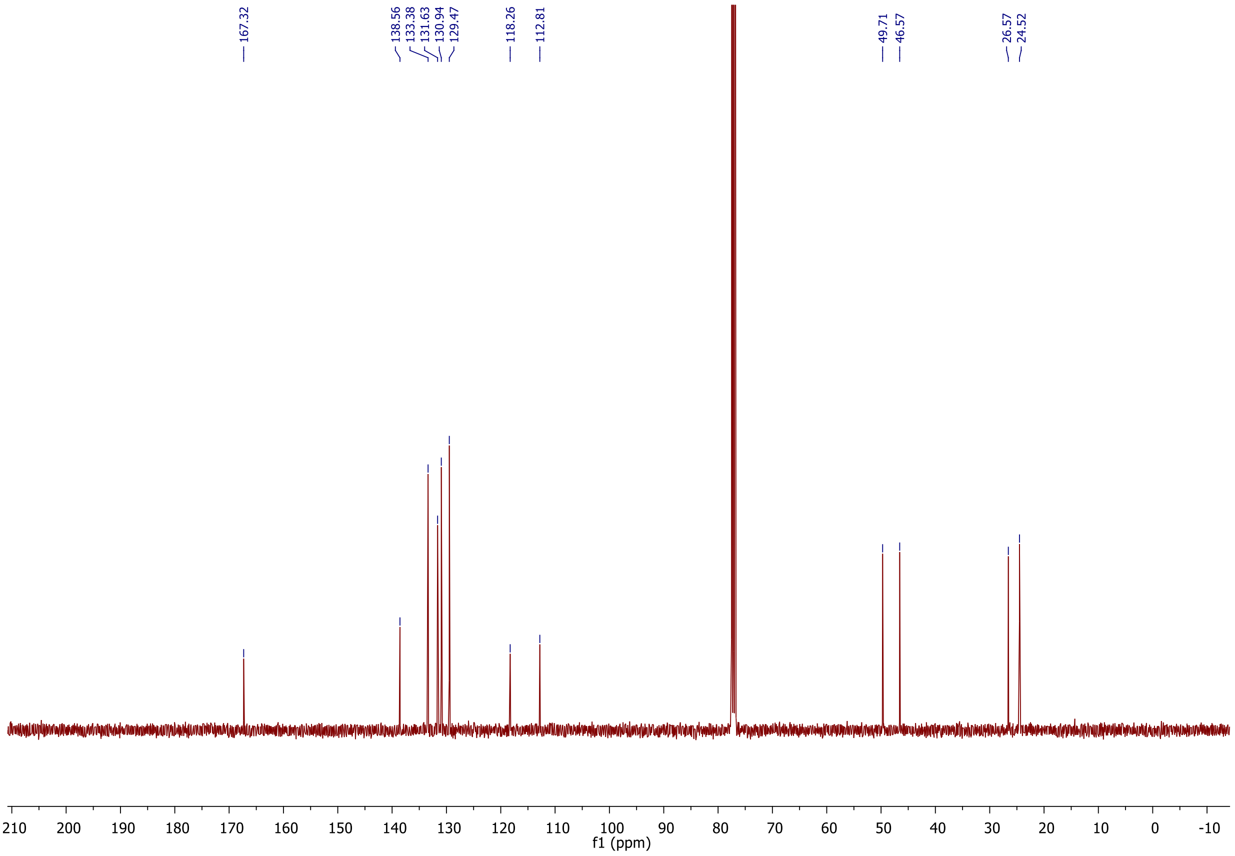

(4-Hydroxy-3-methoxyphenyl)(pyrrolidin-1-yl)methanone (precursor for 4v)

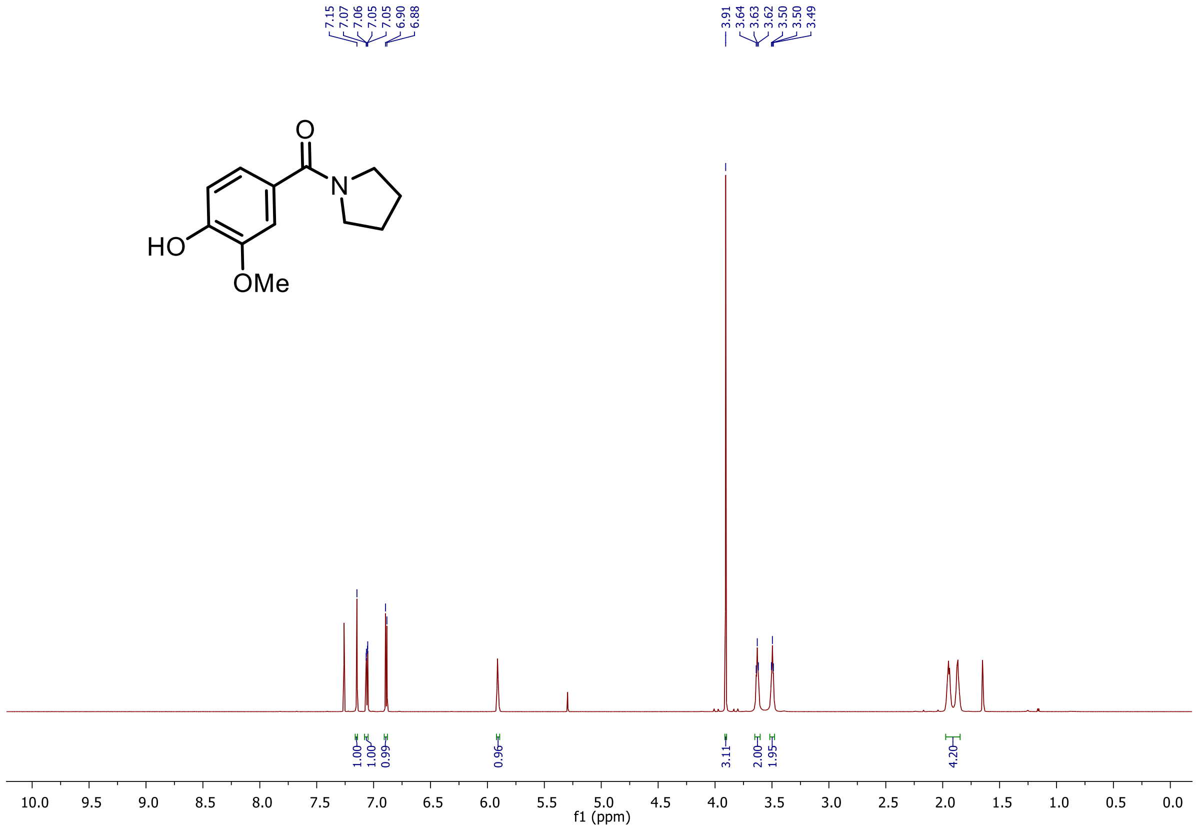

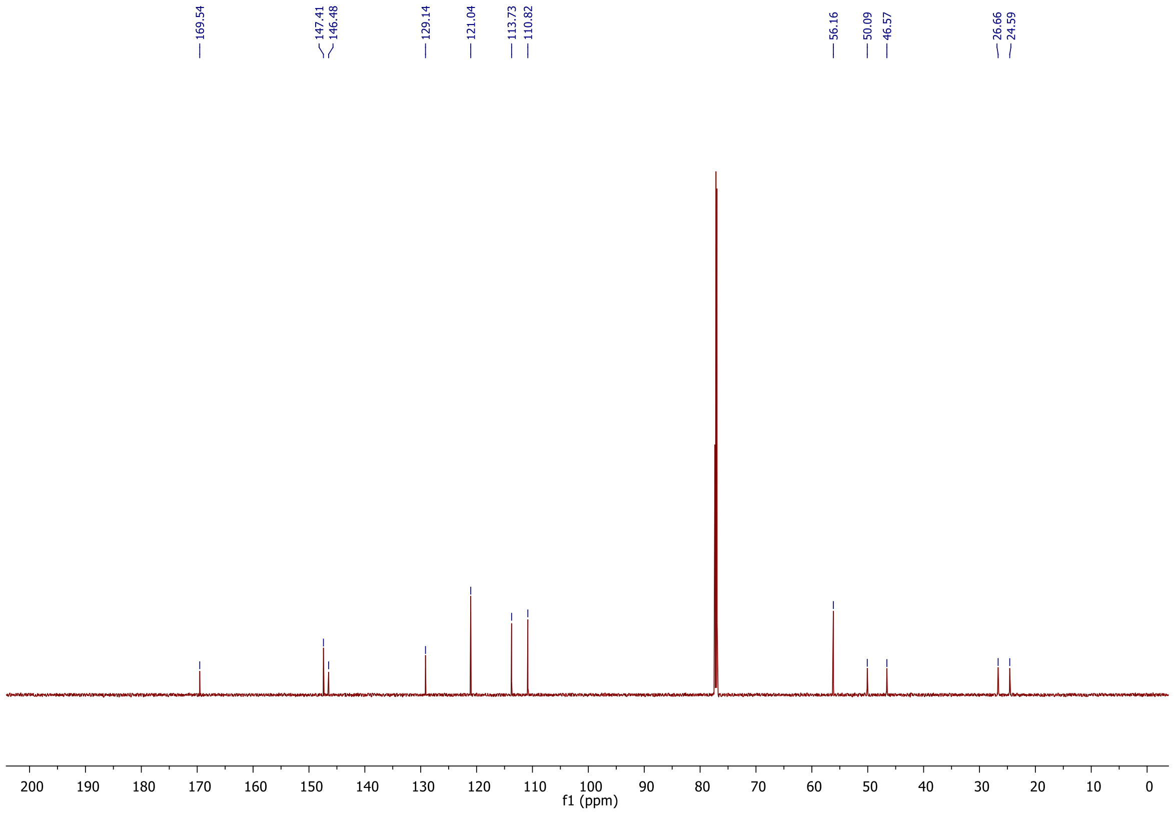

2-Methoxy-4-(pyrrolidine-1-carbonyl)phenyl 2-(3-cyano-4-isobutoxyphenyl)-4-methylthiazole-5-carboxylate (3v)

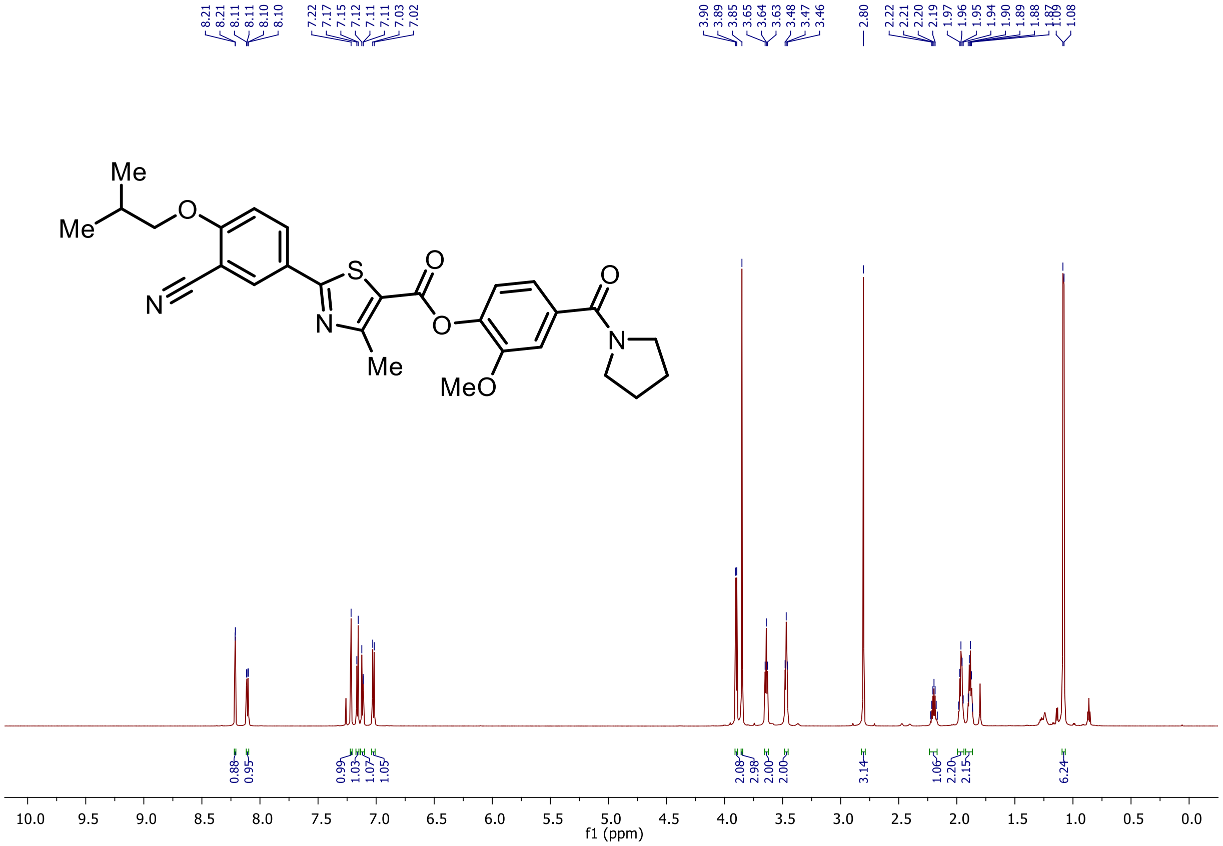

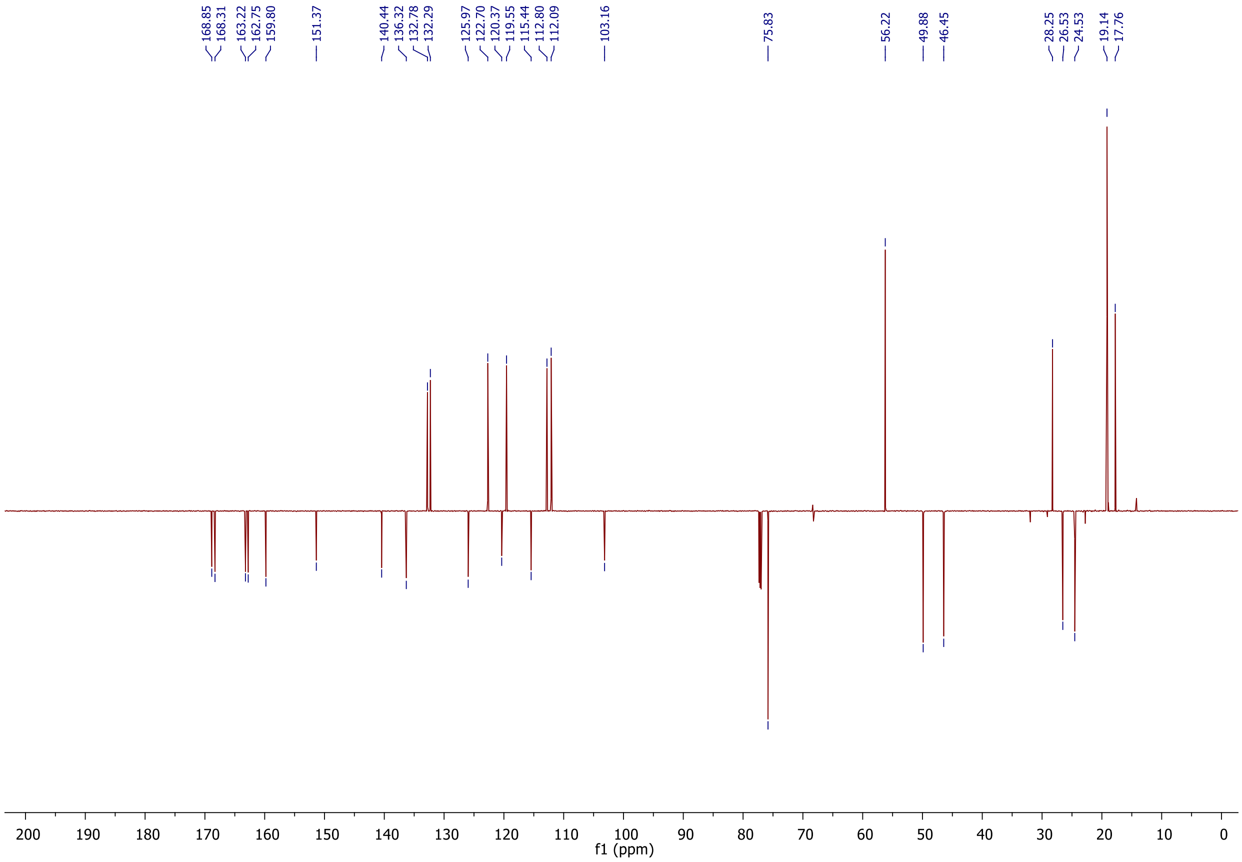

(3,4-Dihydropyridin-1(2H)-yl)(mesityl)methanone (2a)

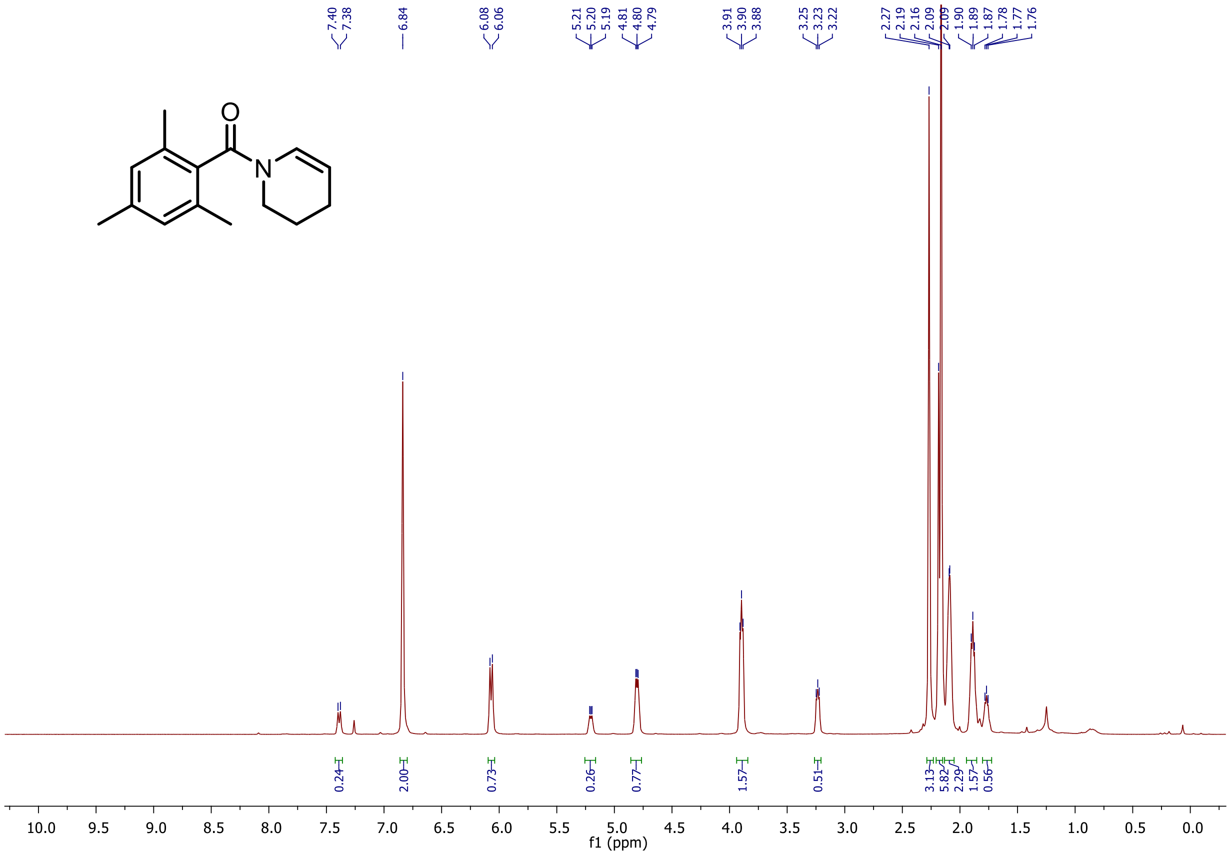

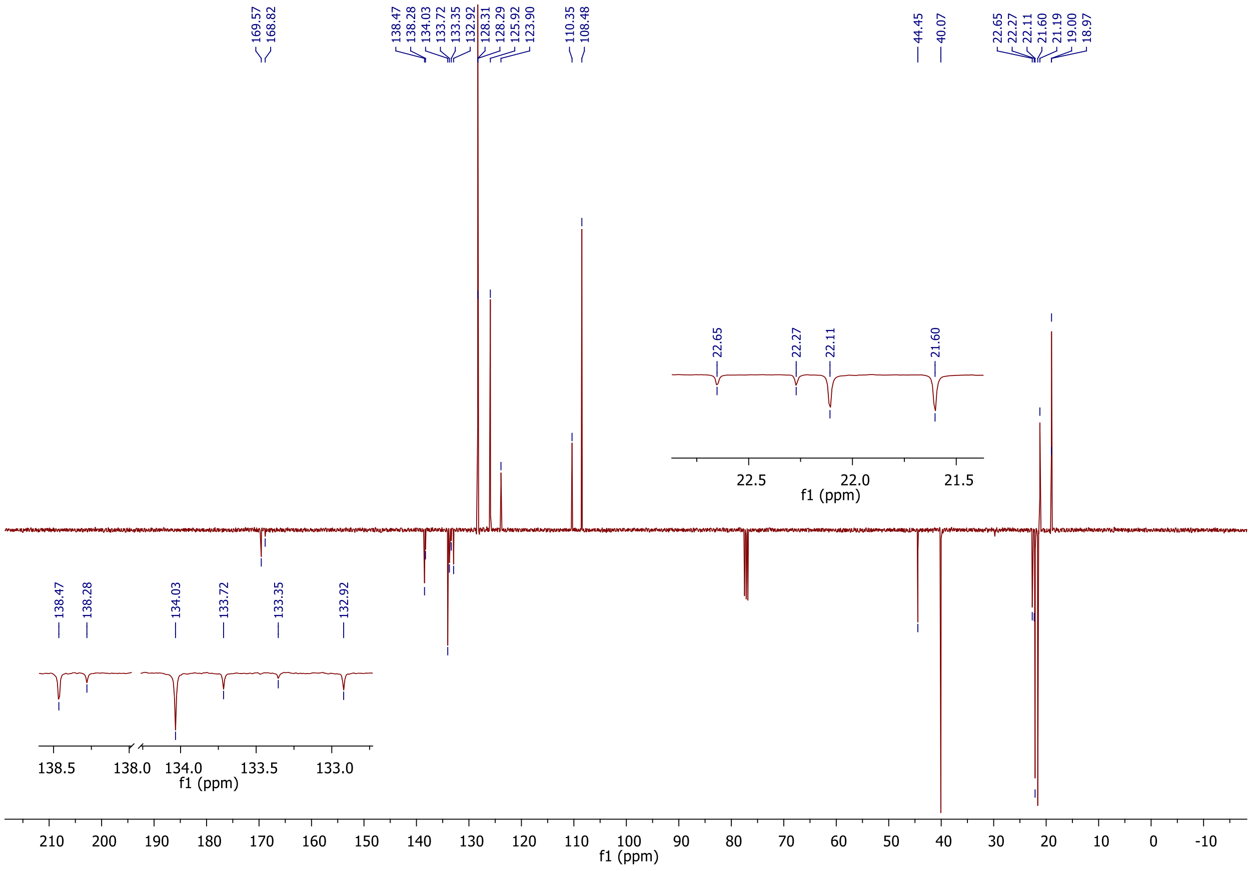

(2,3-Dihydro-1H-pyrrol-1-yl)(mesityl)methanone (2b)

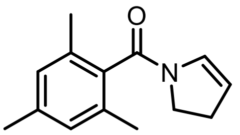

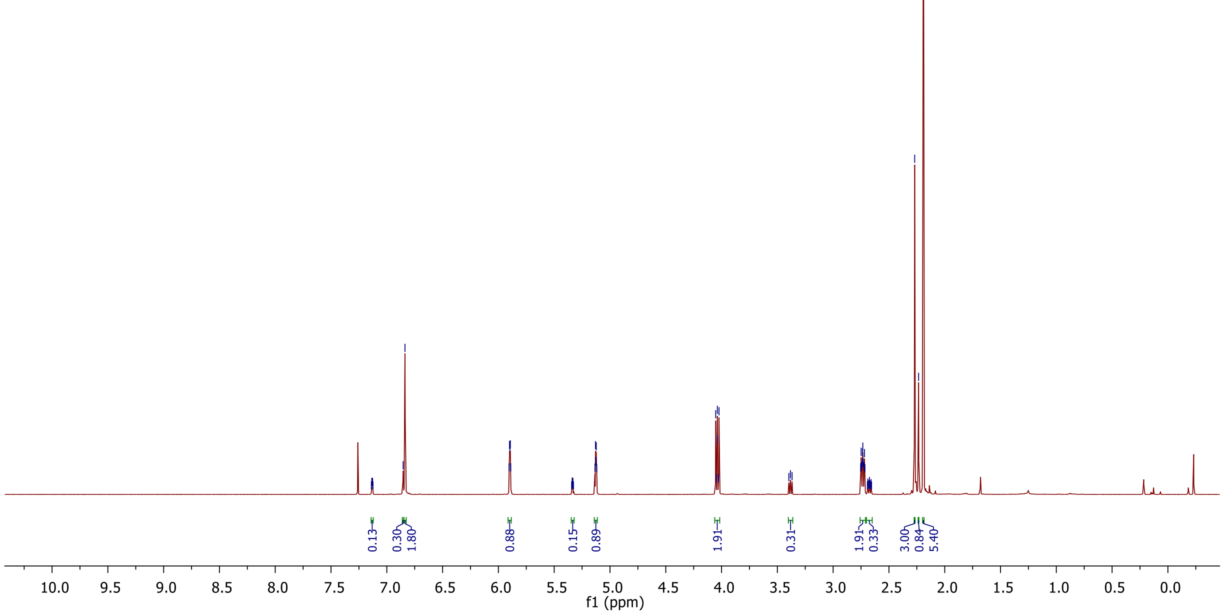

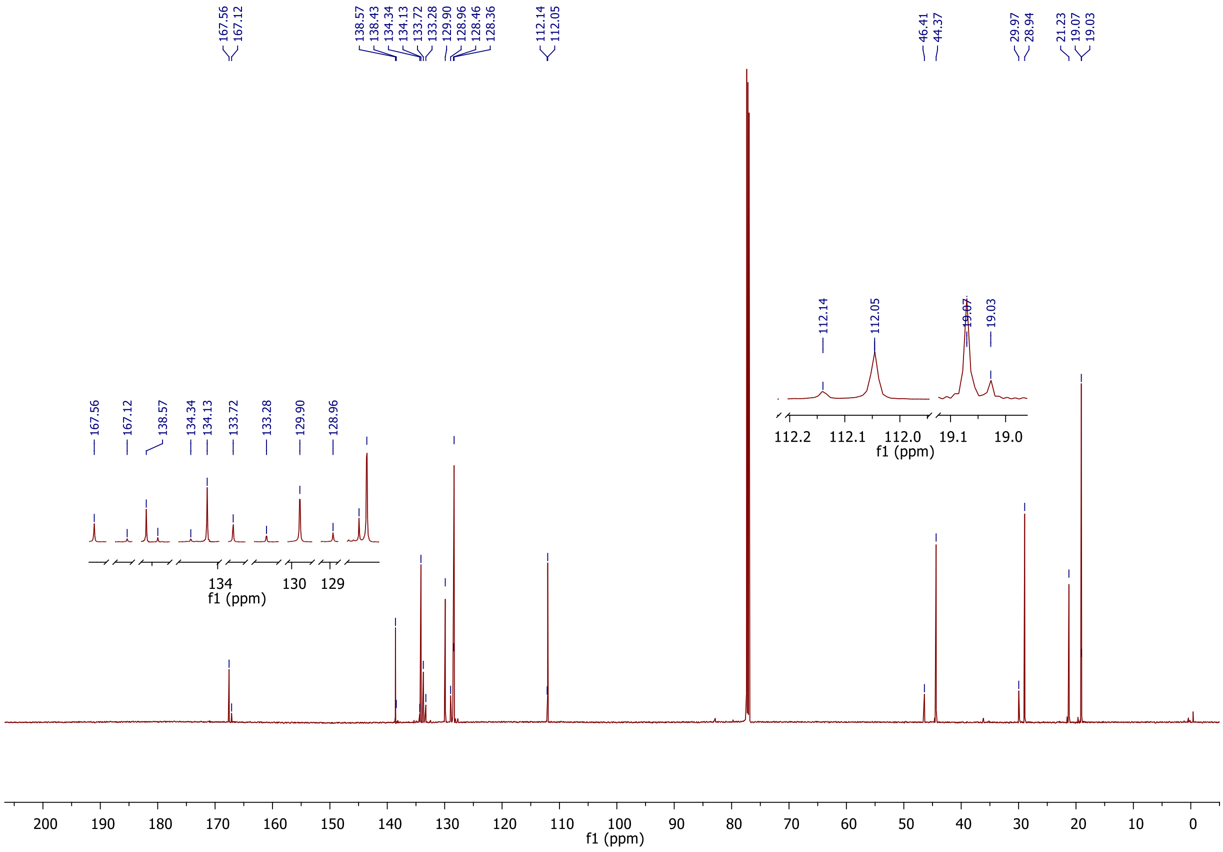

Mesityl(2,3,4,5-tetrahydro-1H-azepin-1-yl)methanone (2c)

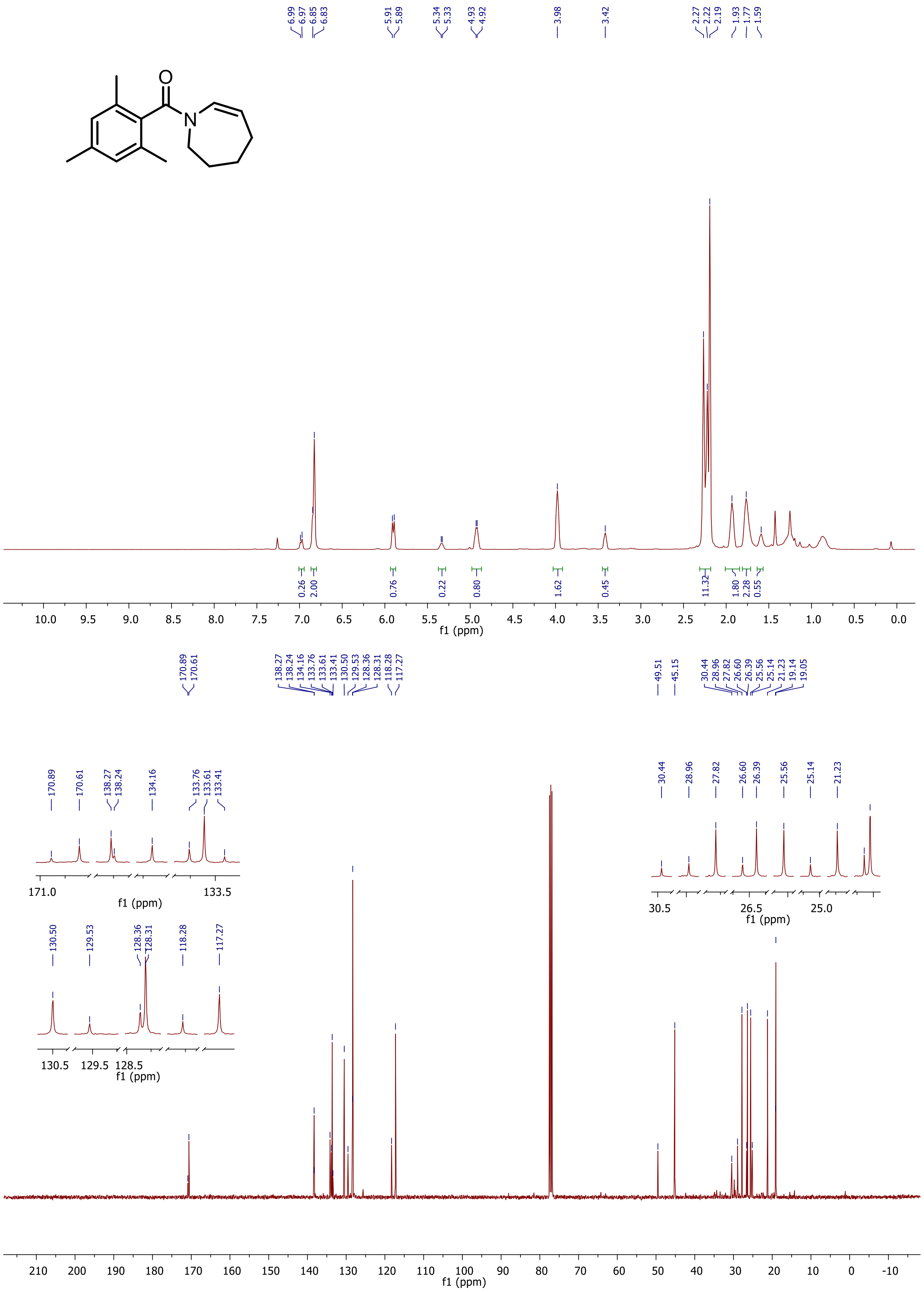

Mesityl(1,4-dioxa-8-azaspiro[4.5]dec-6-en-8-yl)methanone (2d)

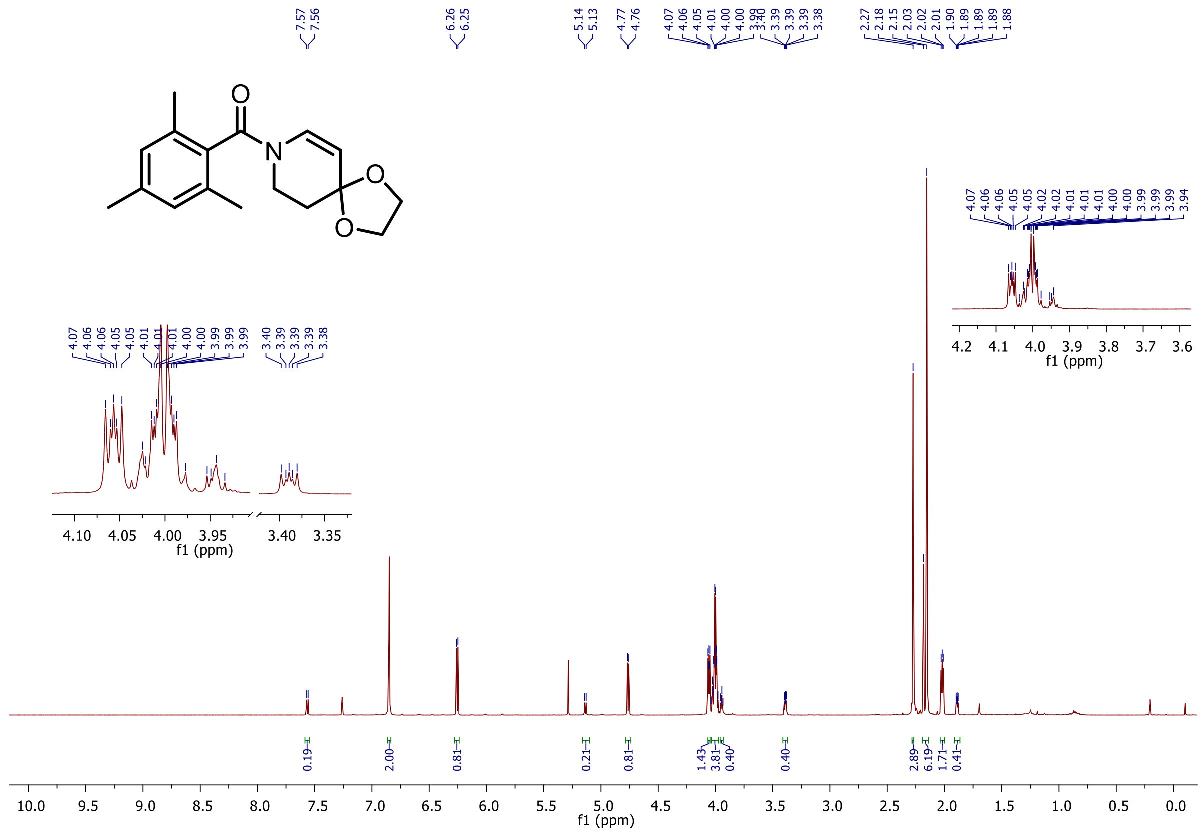

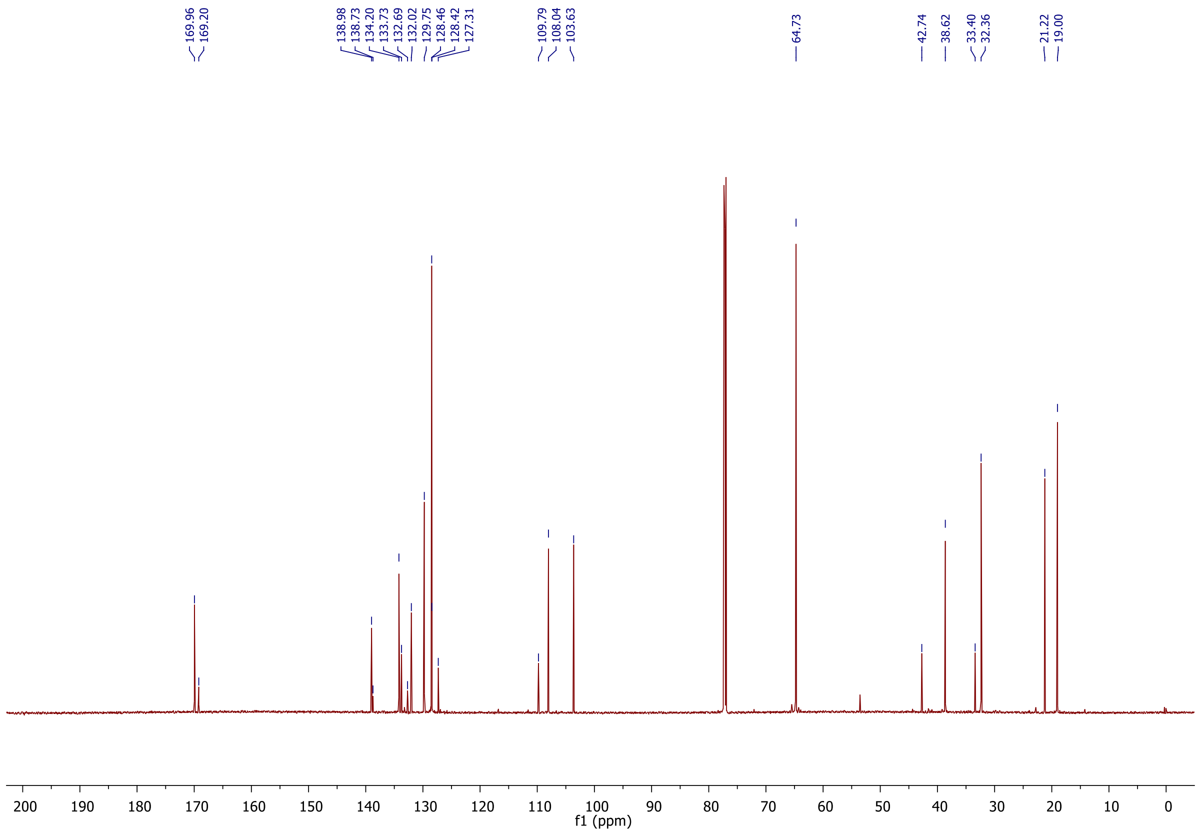

(1,4,5,6,7,7a-Hexahydro-2H-isoindol-2-yl)(mesityl)methanone (2e)

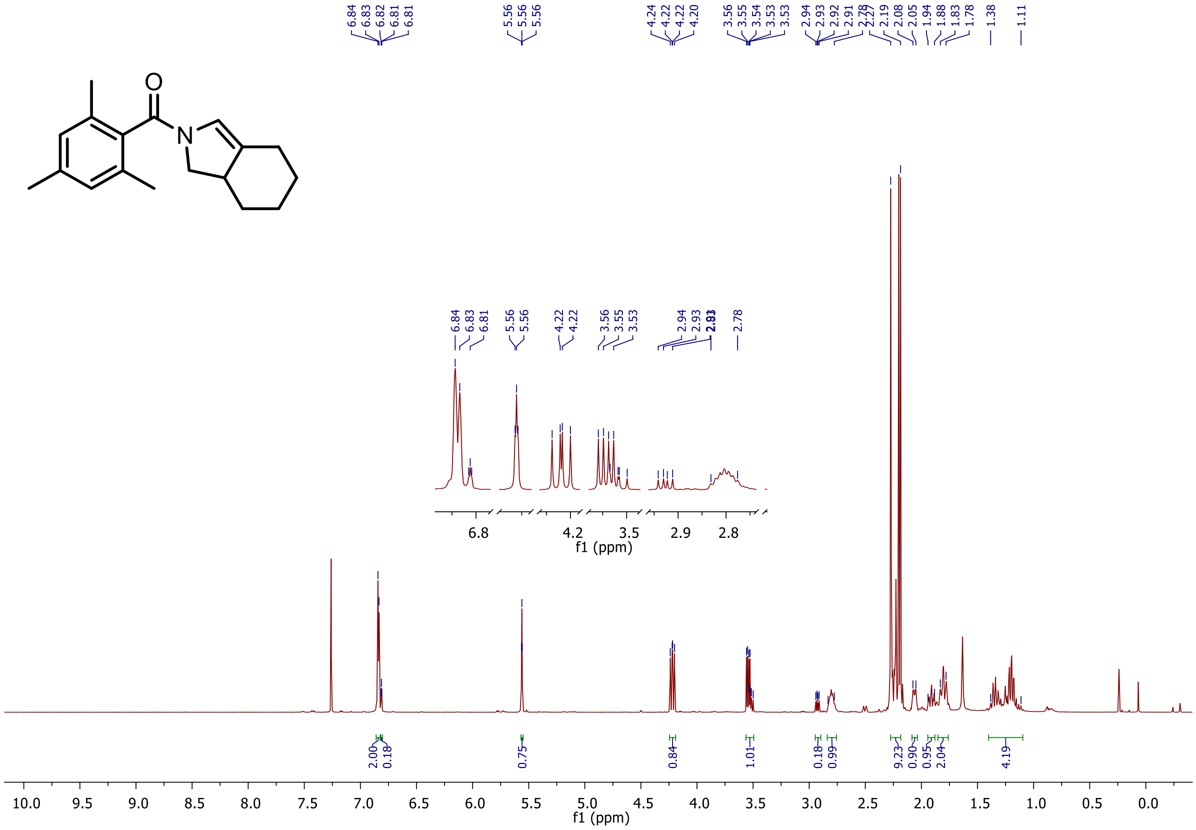

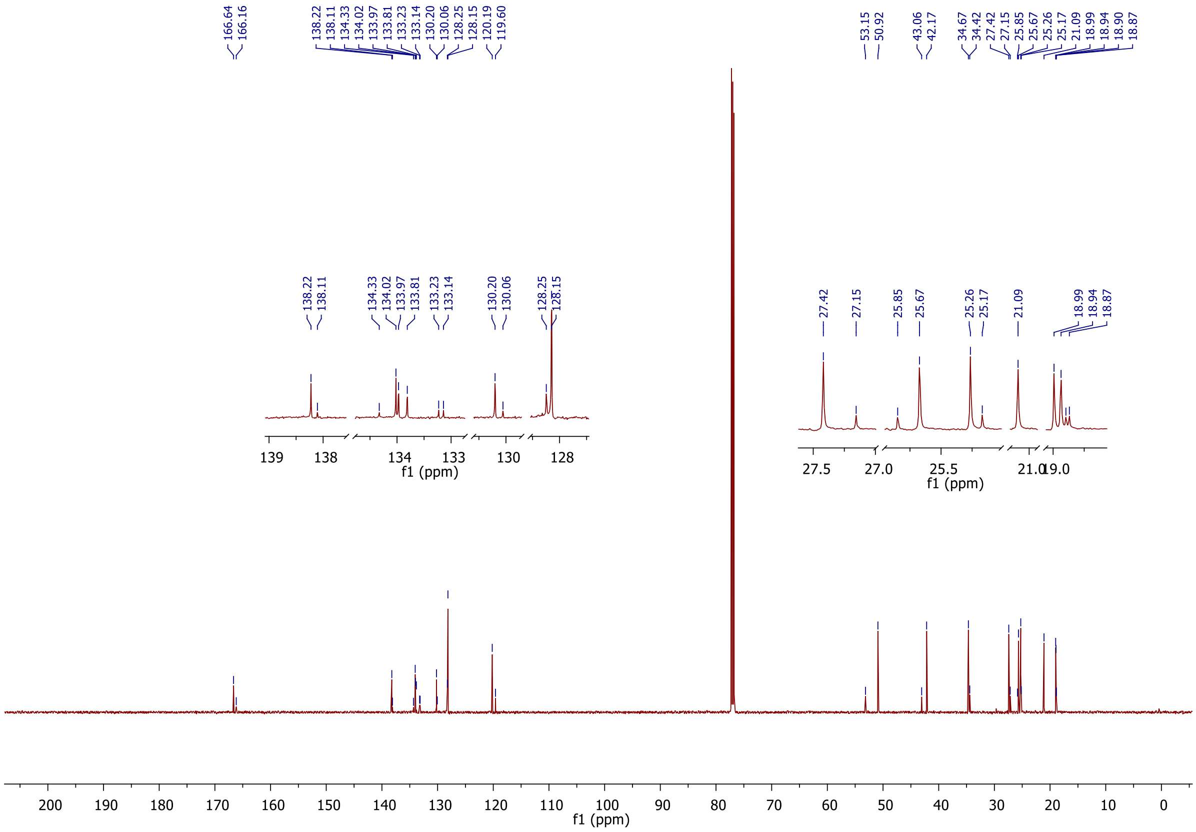

(2,3-Dihydro-4H-1,4-oxazin-4-yl)(mesityl)methanone (2f)

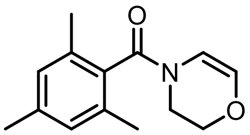

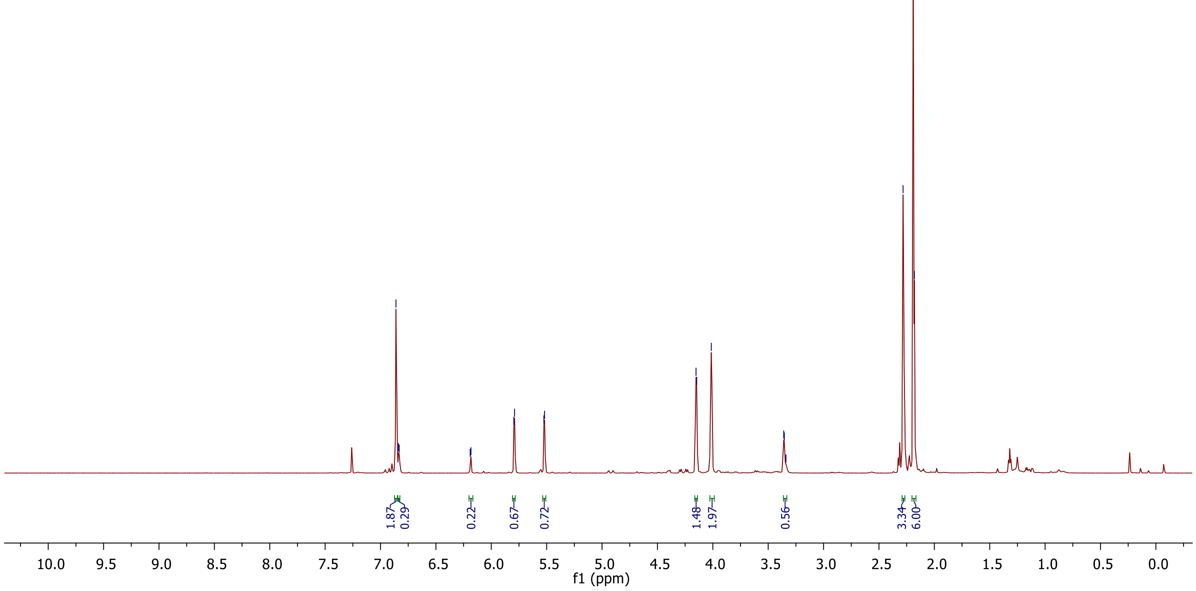

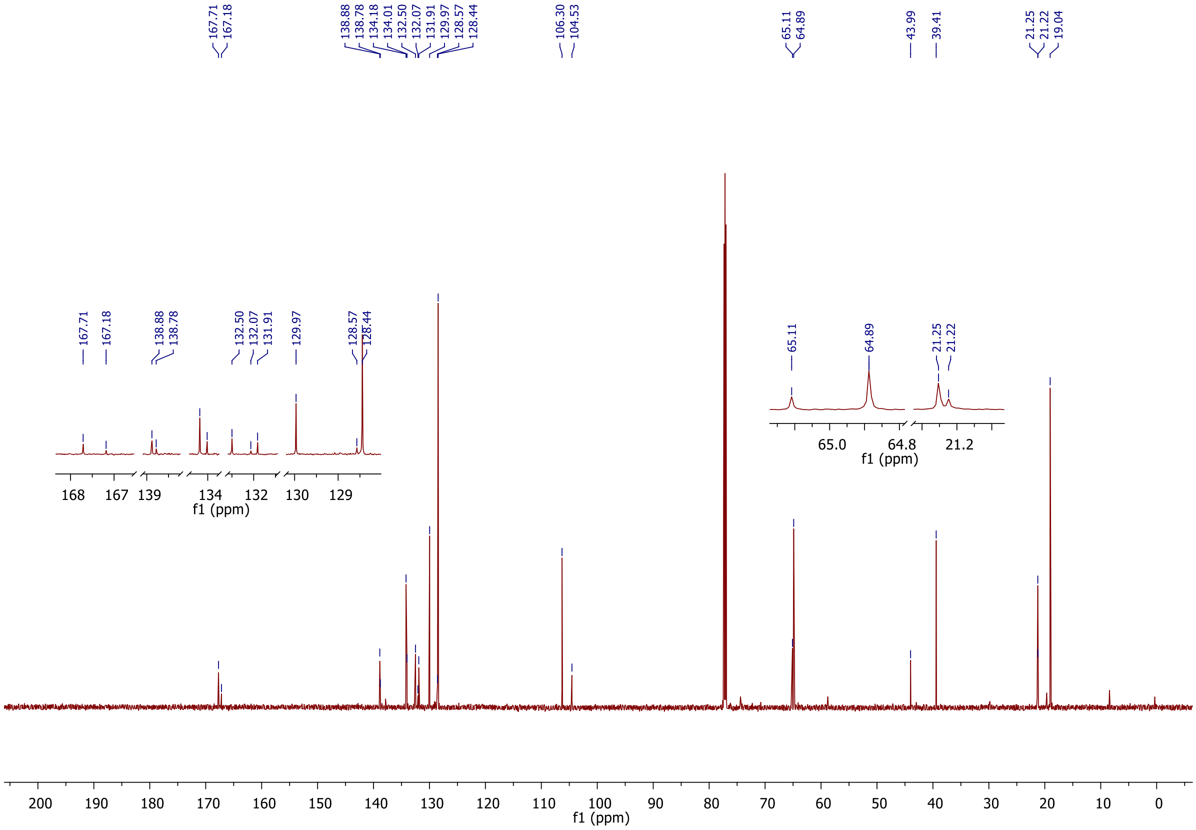

Ethyl 4-(2,4,6-trimethylbenzoyl)-3,4-dihydropyrazine-1(2H)-carboxylate (2g)

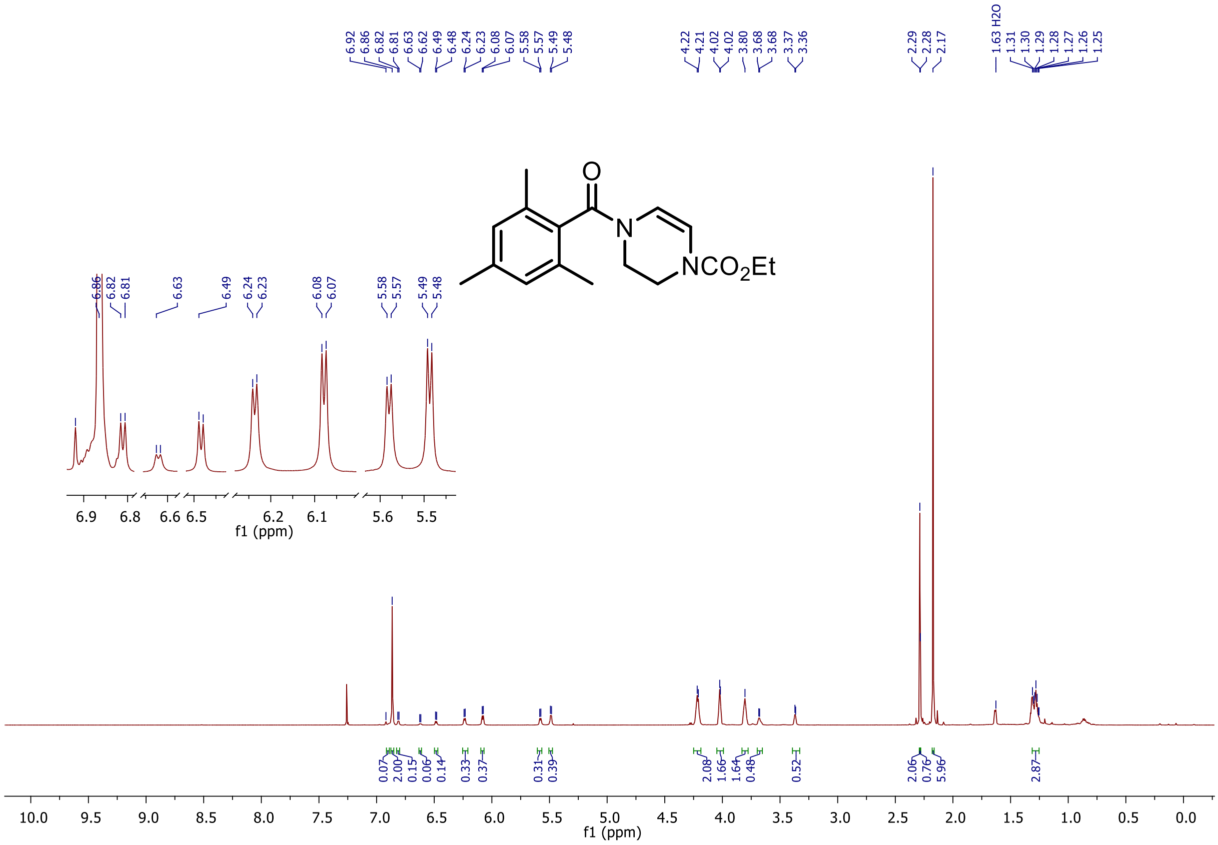

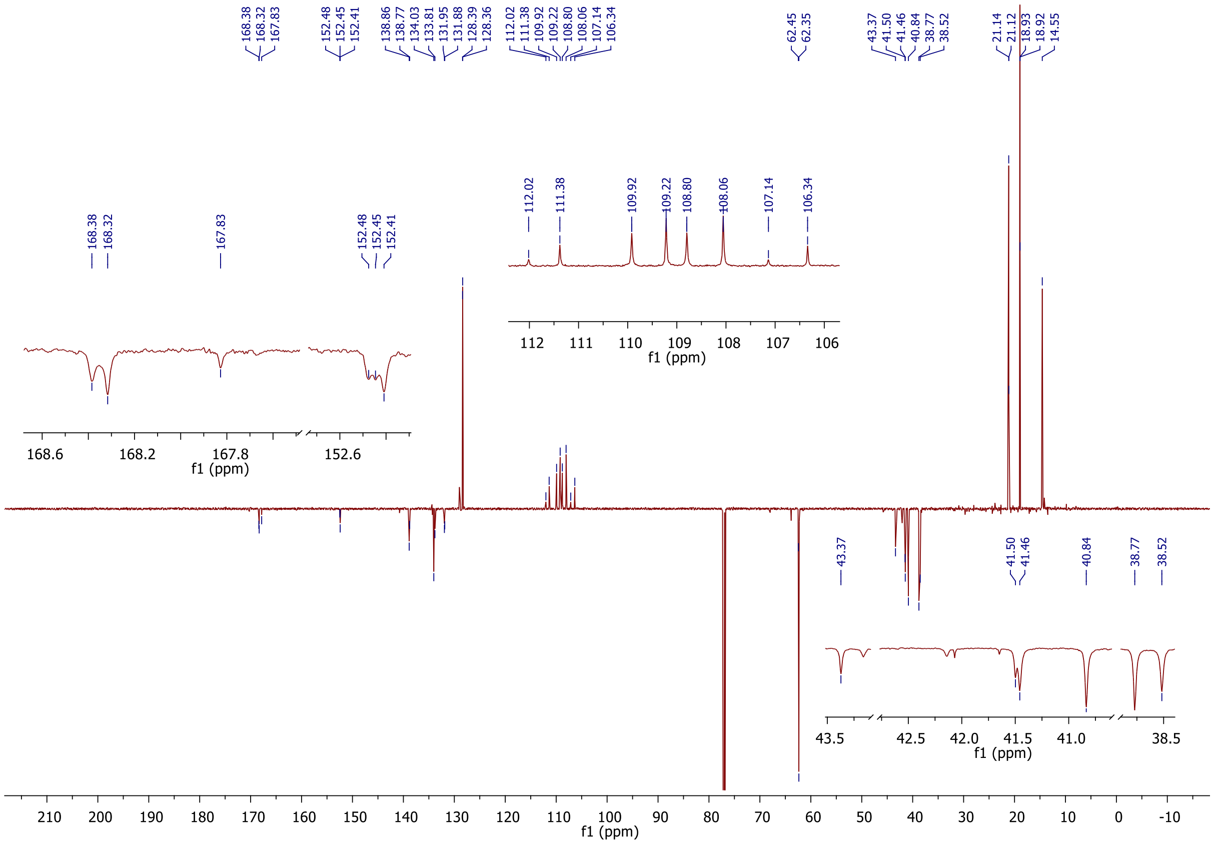

N-Ethyl-2,4,6-trimethyl-N-vinylbenzamide (2h)

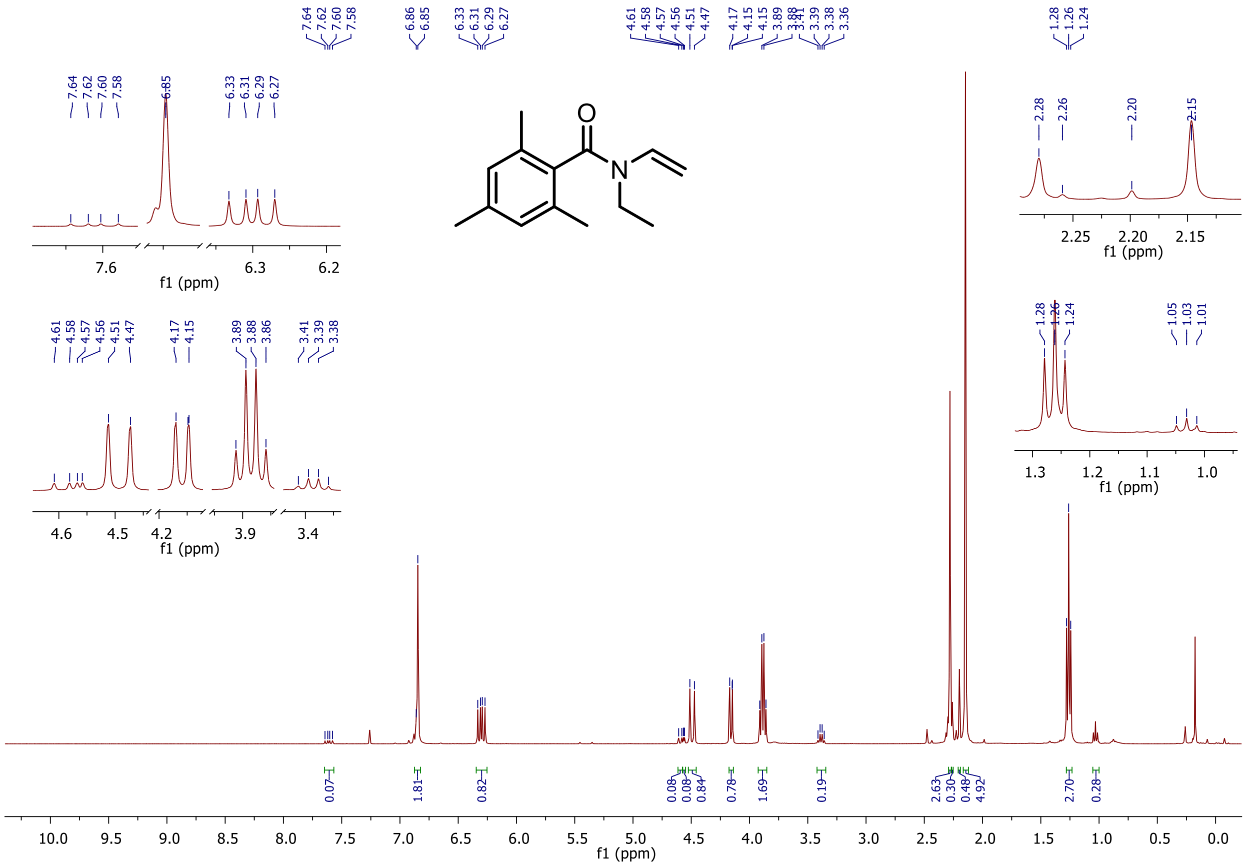

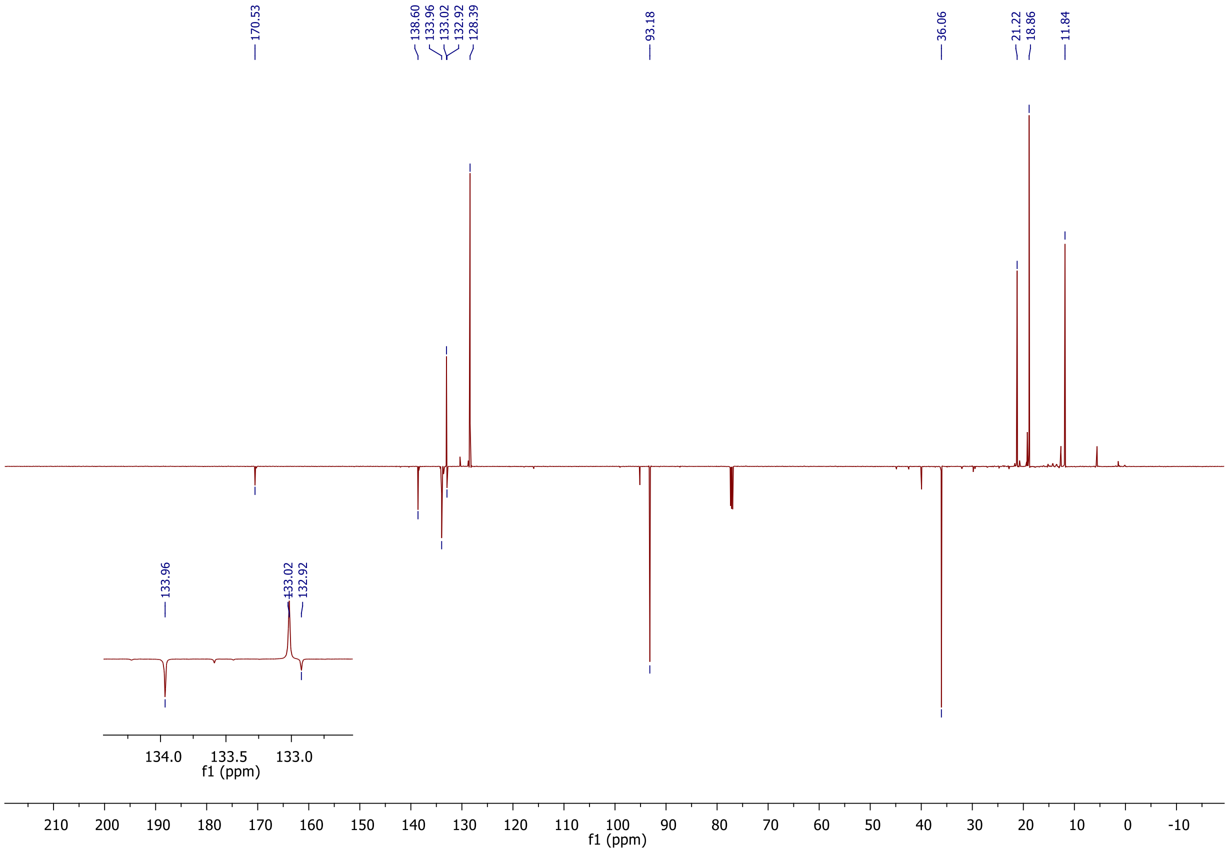

(E)-2,4,6-Trimethyl-N-(prop-1-en-1-yl)-N-propylbenzamide (2i)

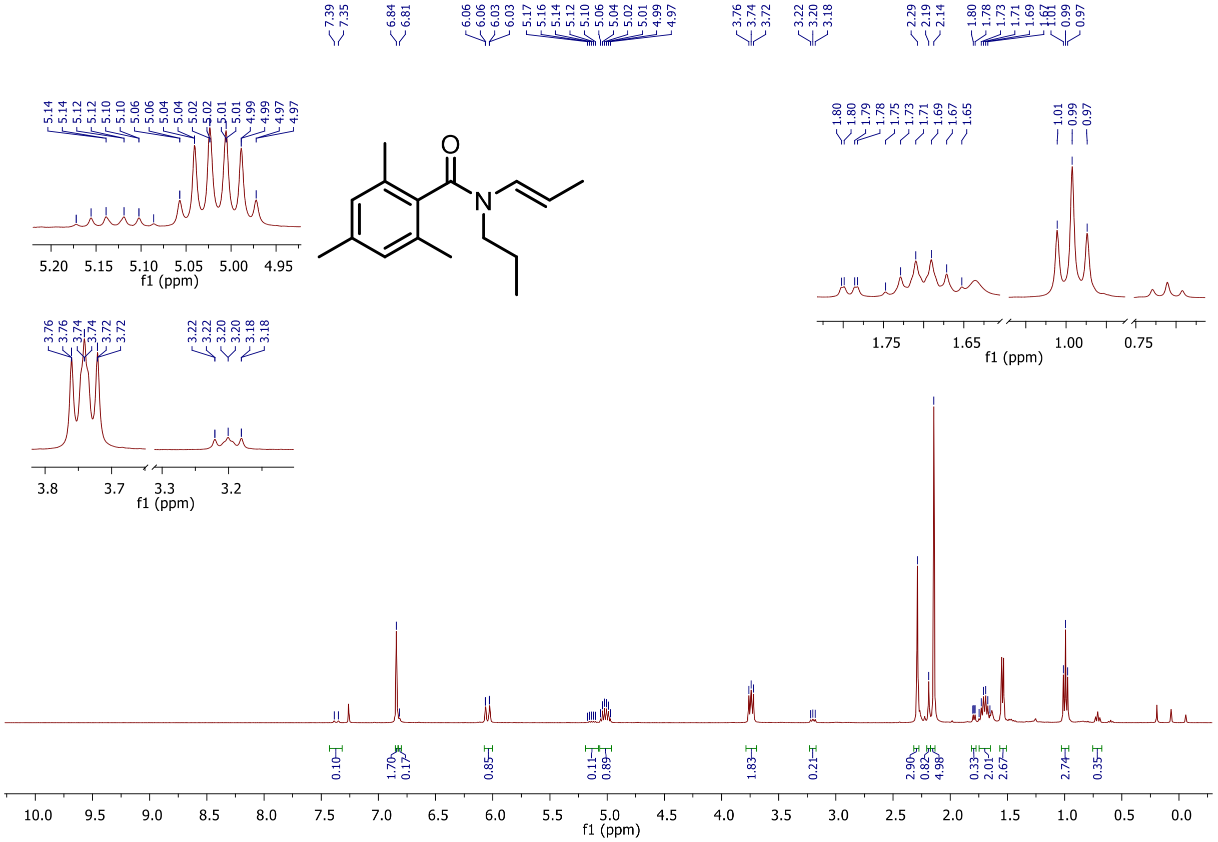

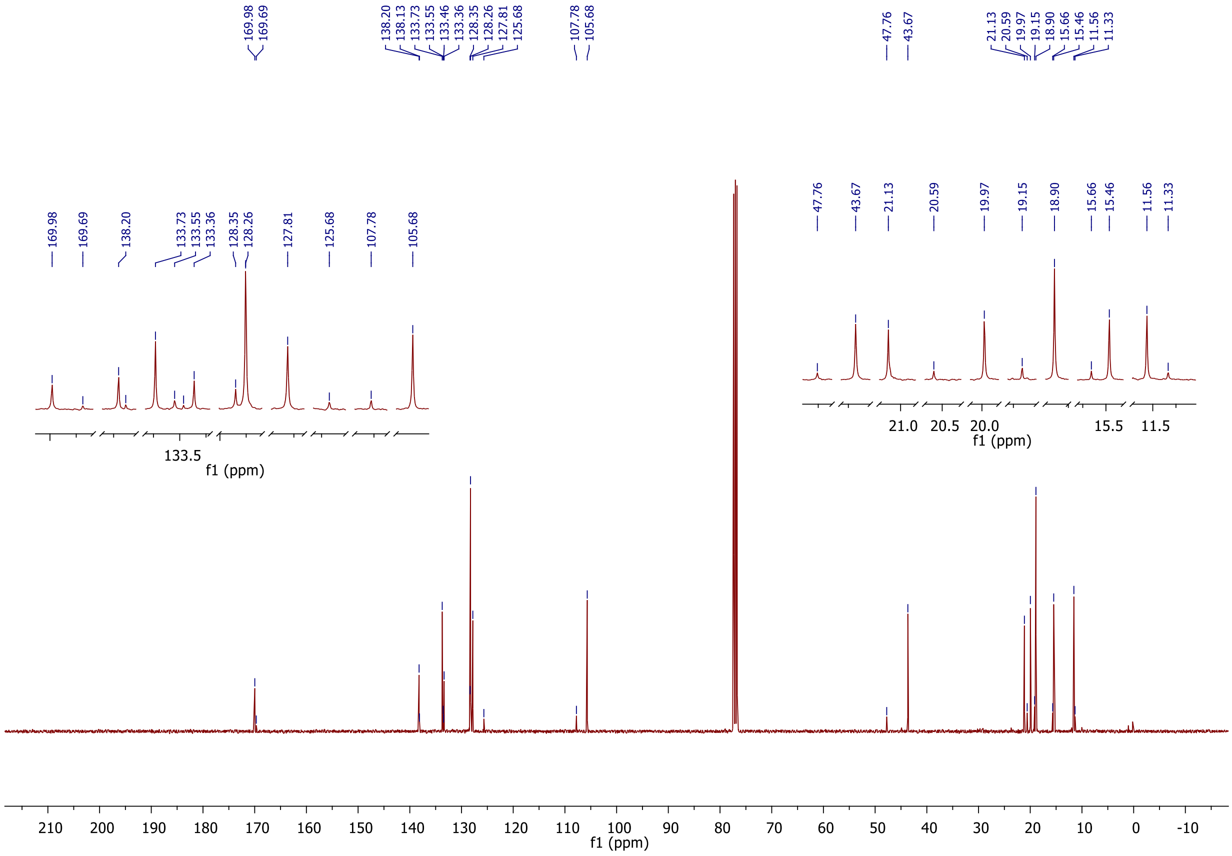

(E)-N-(But-1-en-1-yl)-N-butyl-2,4,6-trimethylbenzamide (2j)

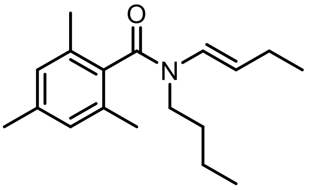

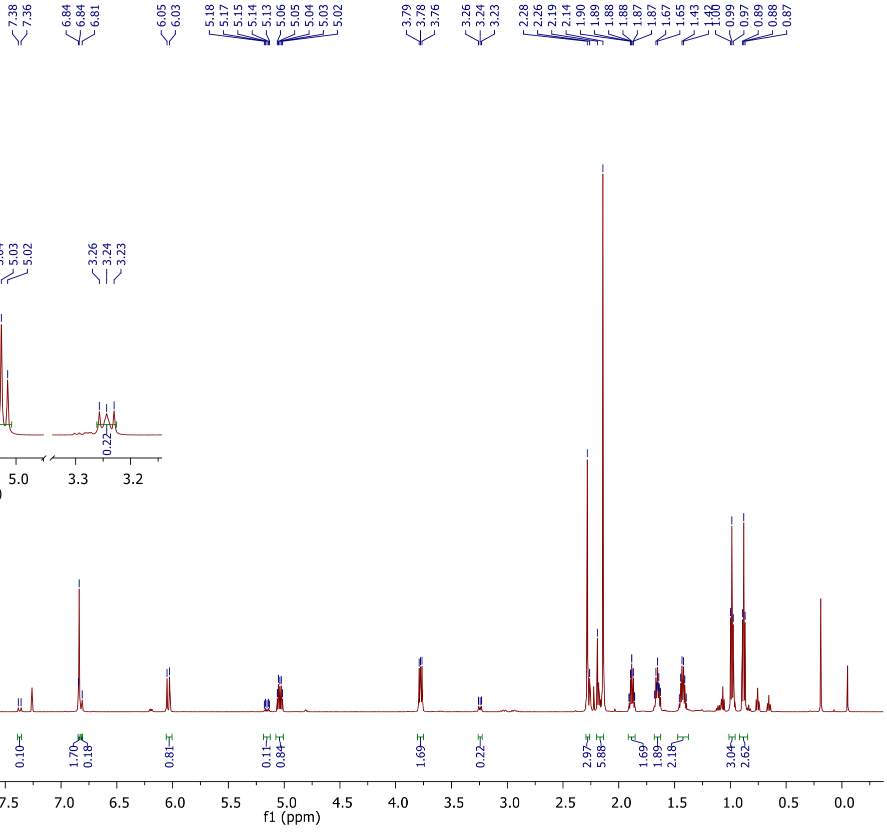

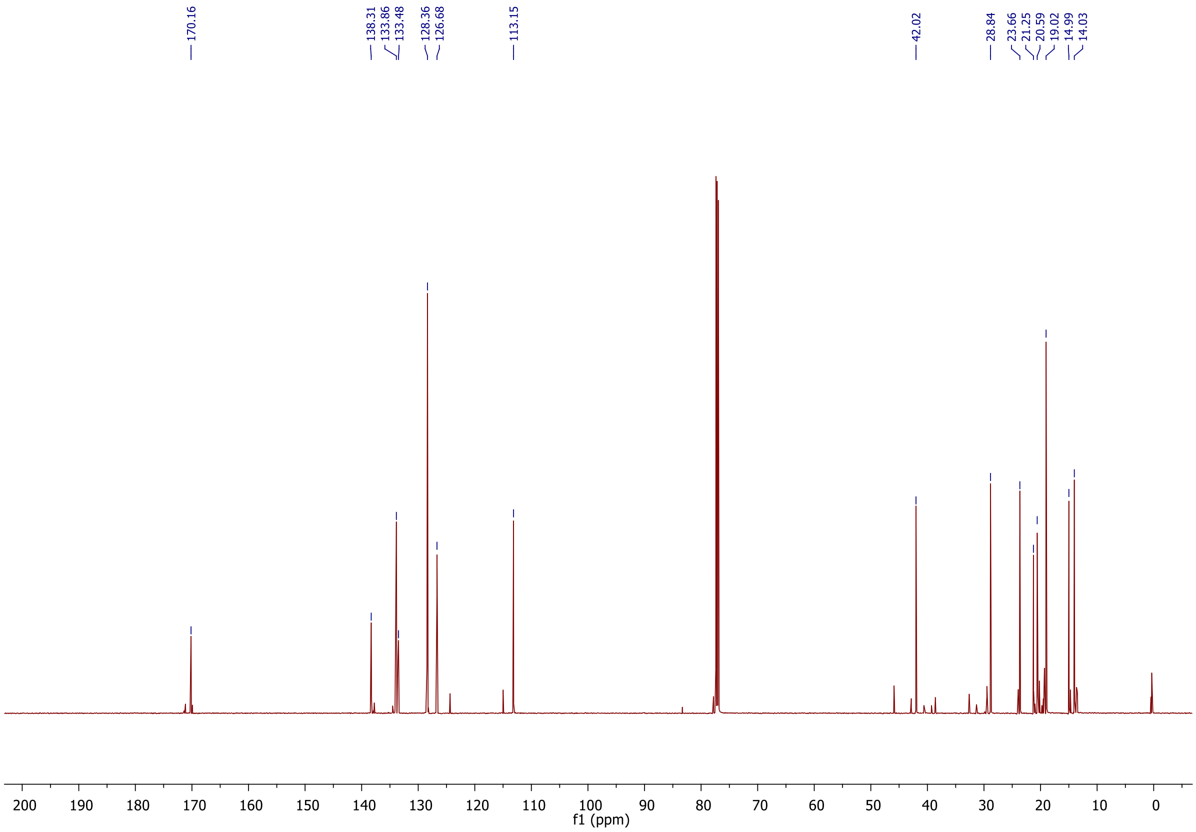

(2-Benzyl-3,4-dihydropyridin-1(2H)-yl)(mesityl)methanone (2k)

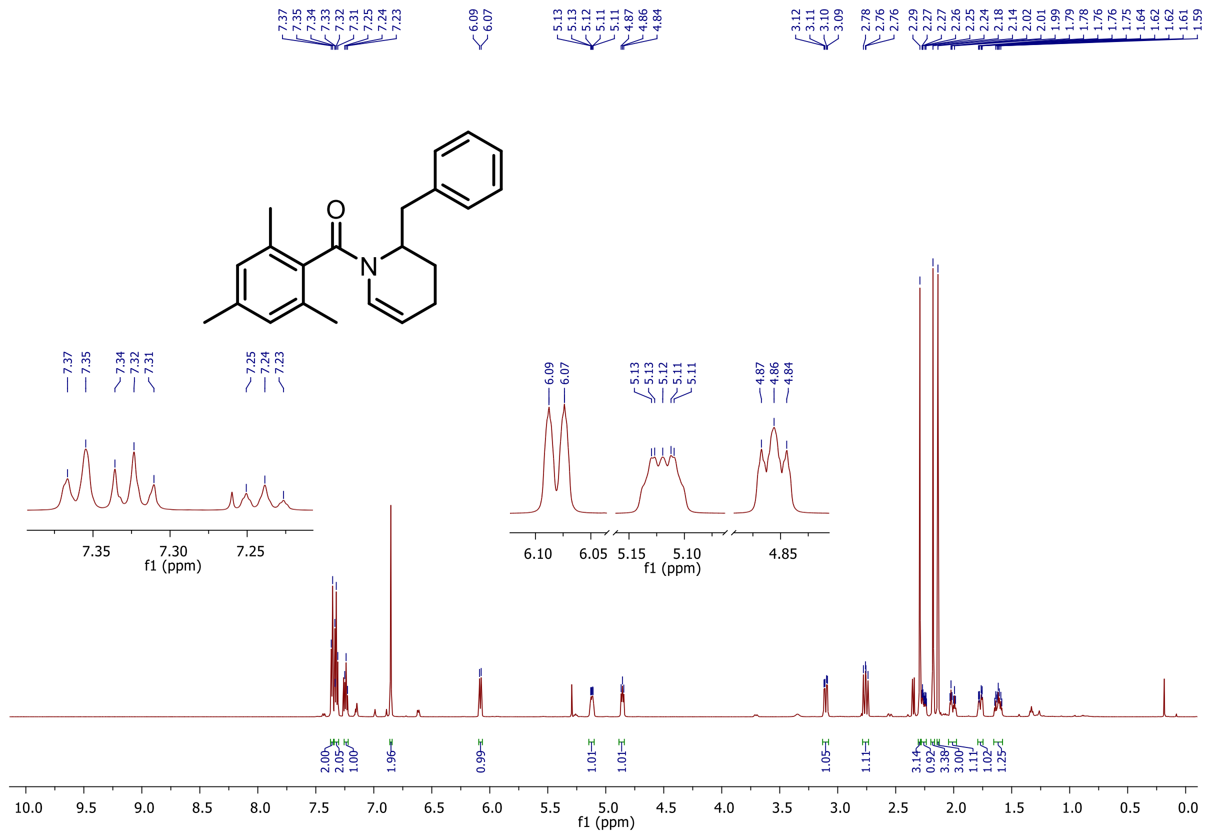

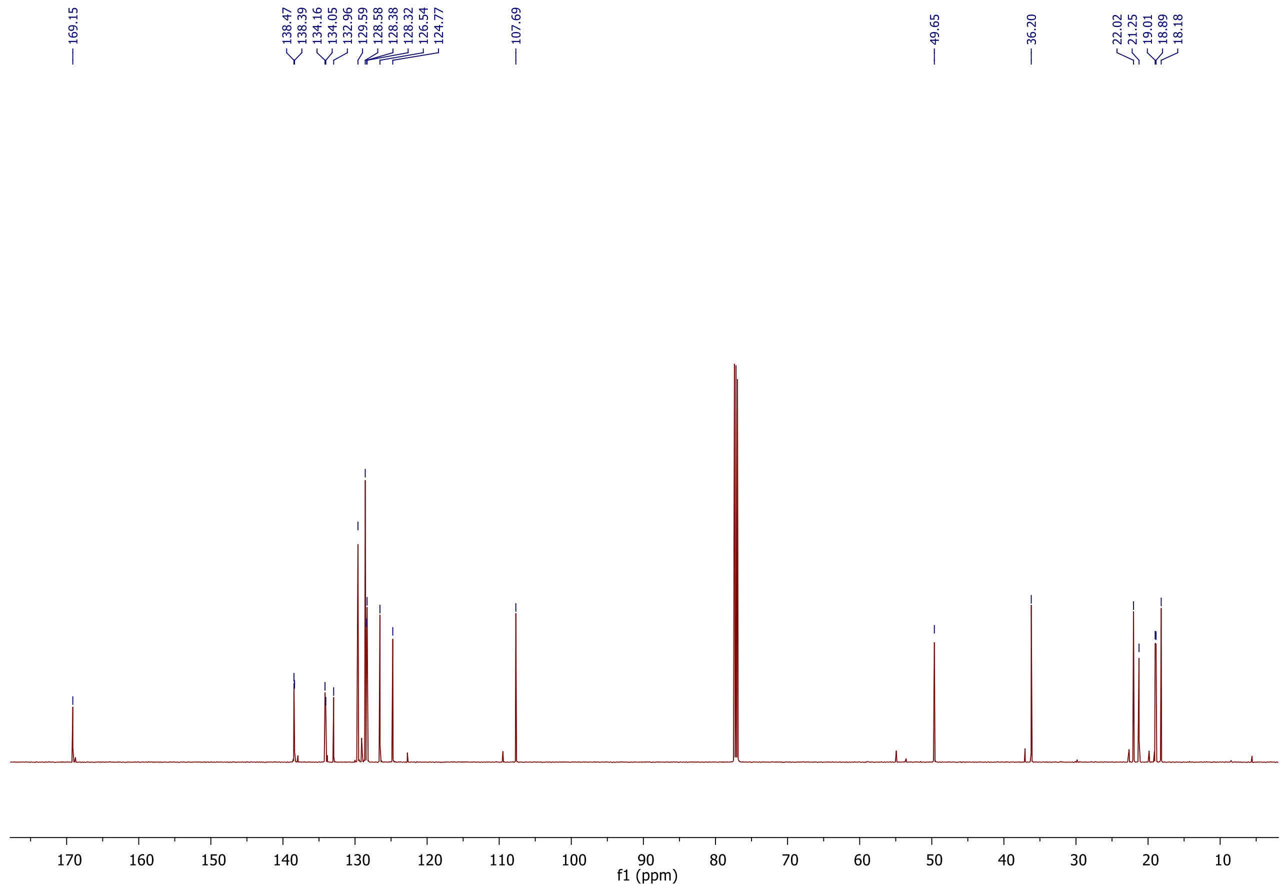

Mesityl(6-methyl-3,4-dihydropyridin-1(2H)-yl)methanone (2l)

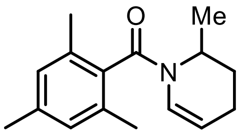

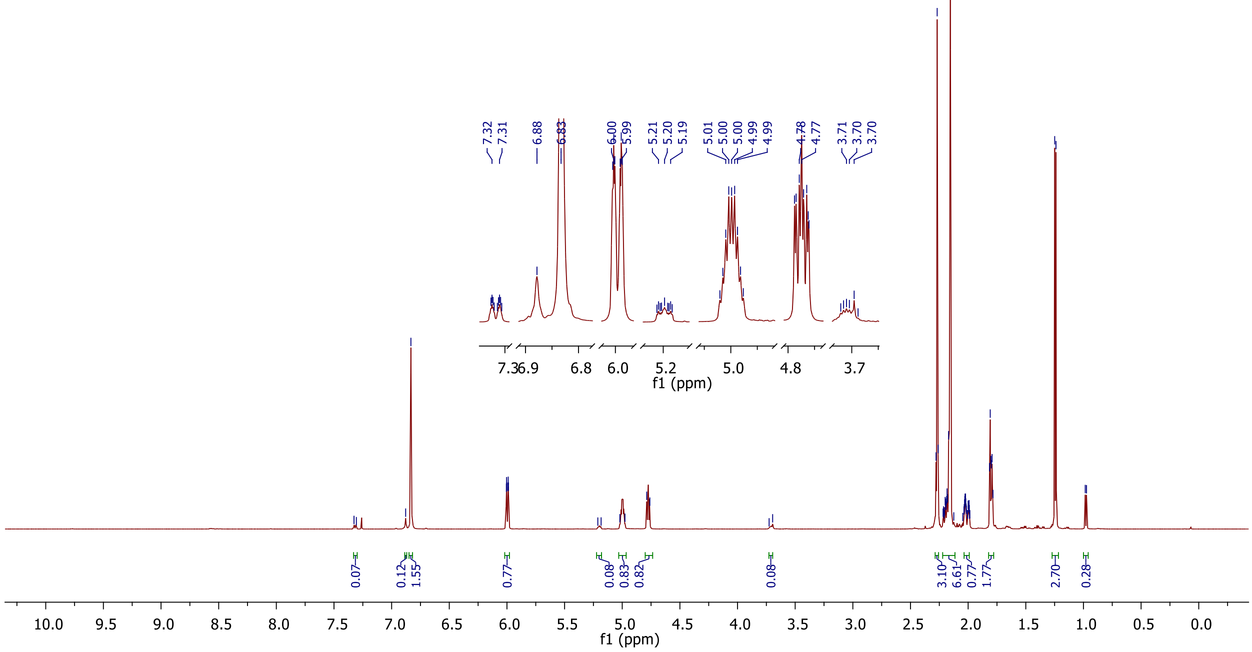

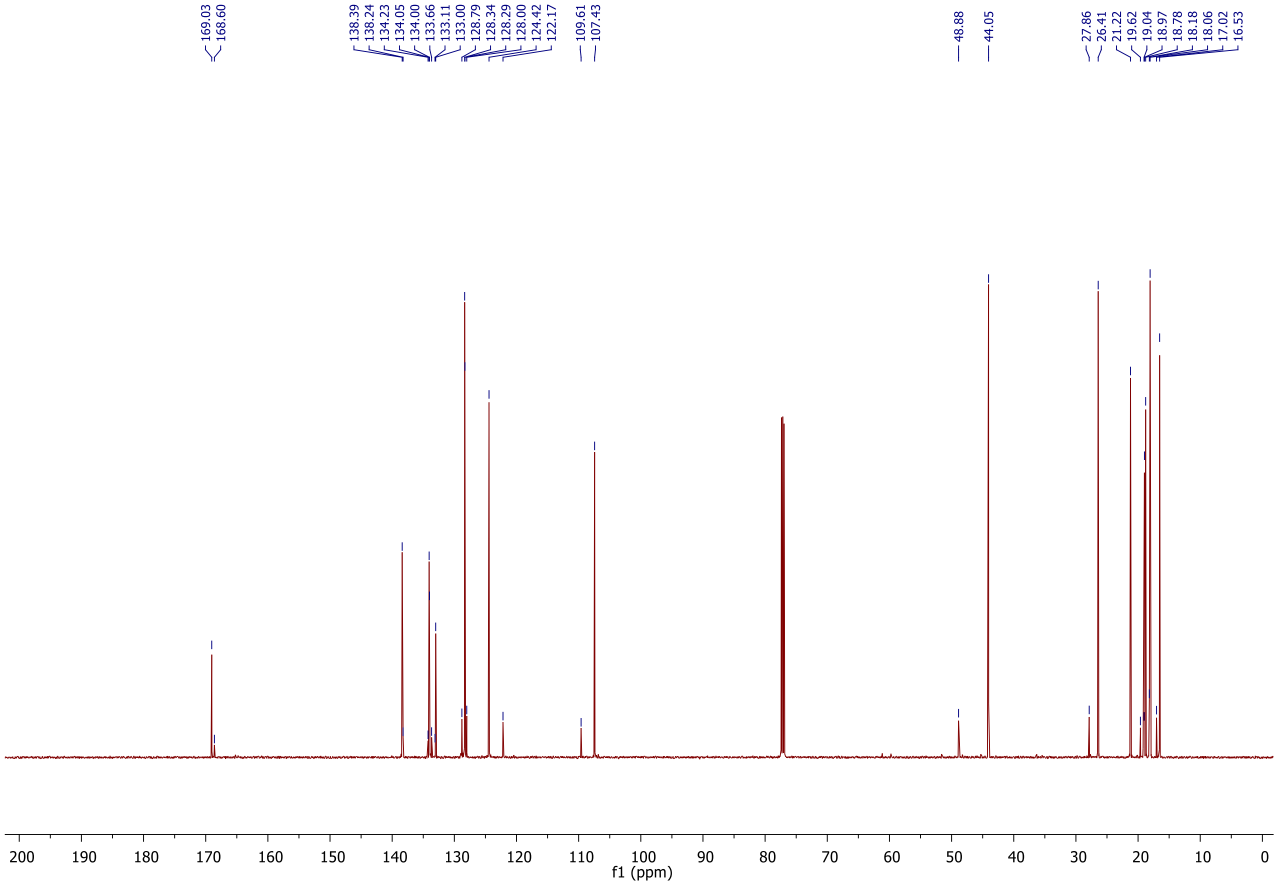

Mesityl(2-phenyl-2,3-dihydro-1H-pyrrol-1-yl)methanone (2m)

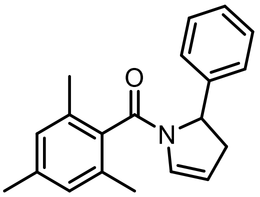

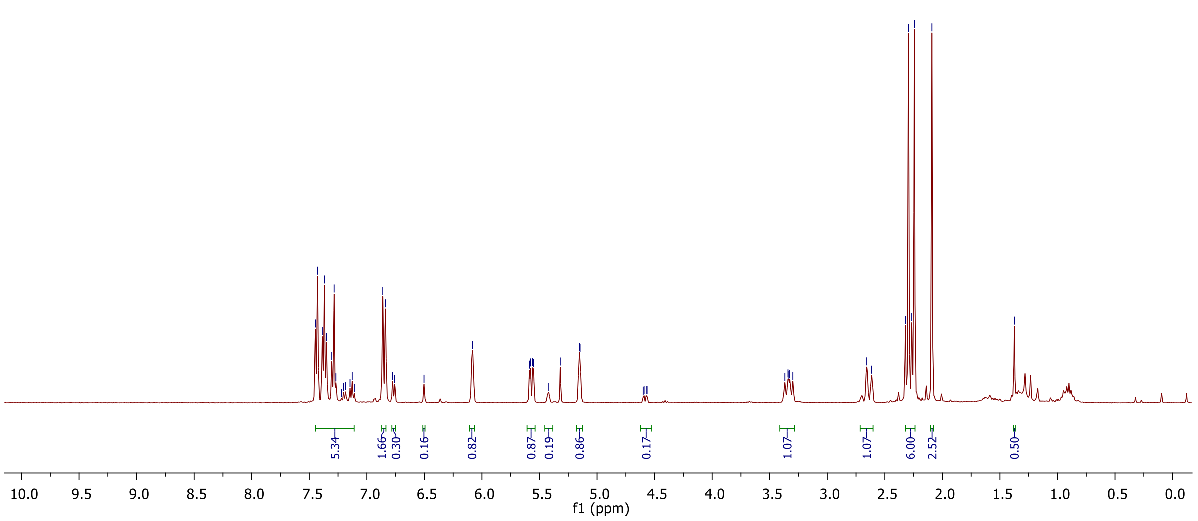

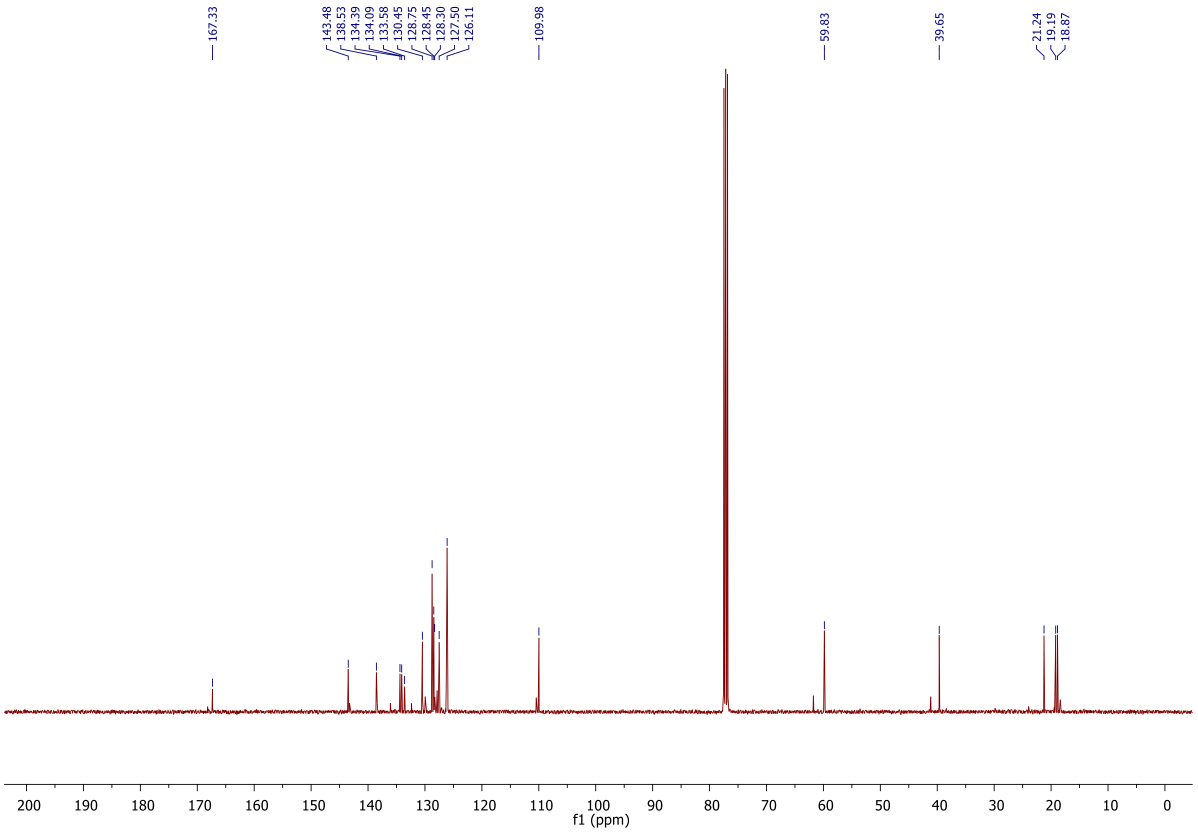

((4aS,8aR)-4a,5,6,7,8,8a-Hexahydroquinolin-1(4H)-yl)(mesityl)methanone (2n)

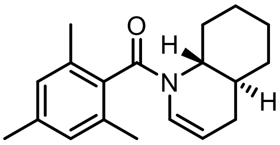

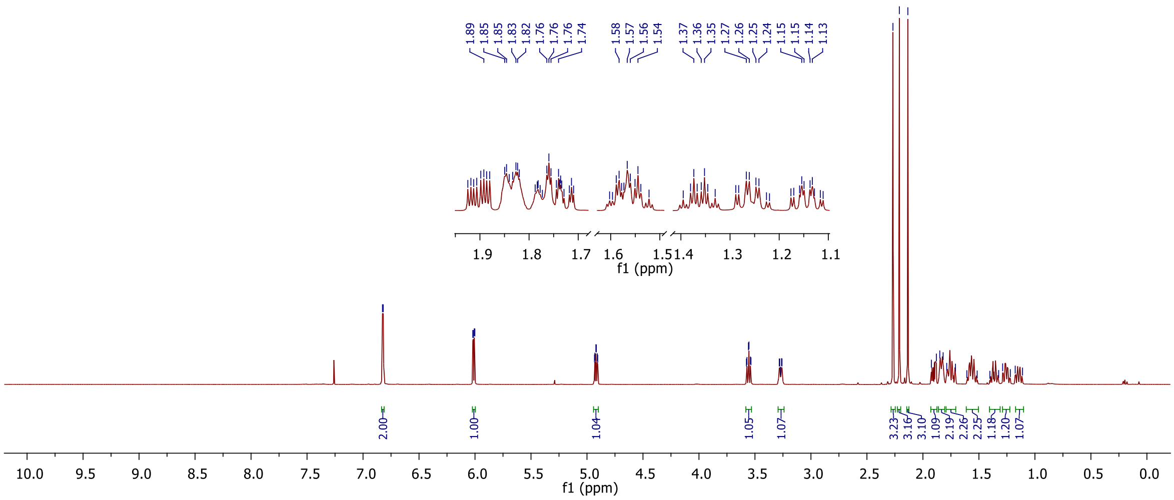

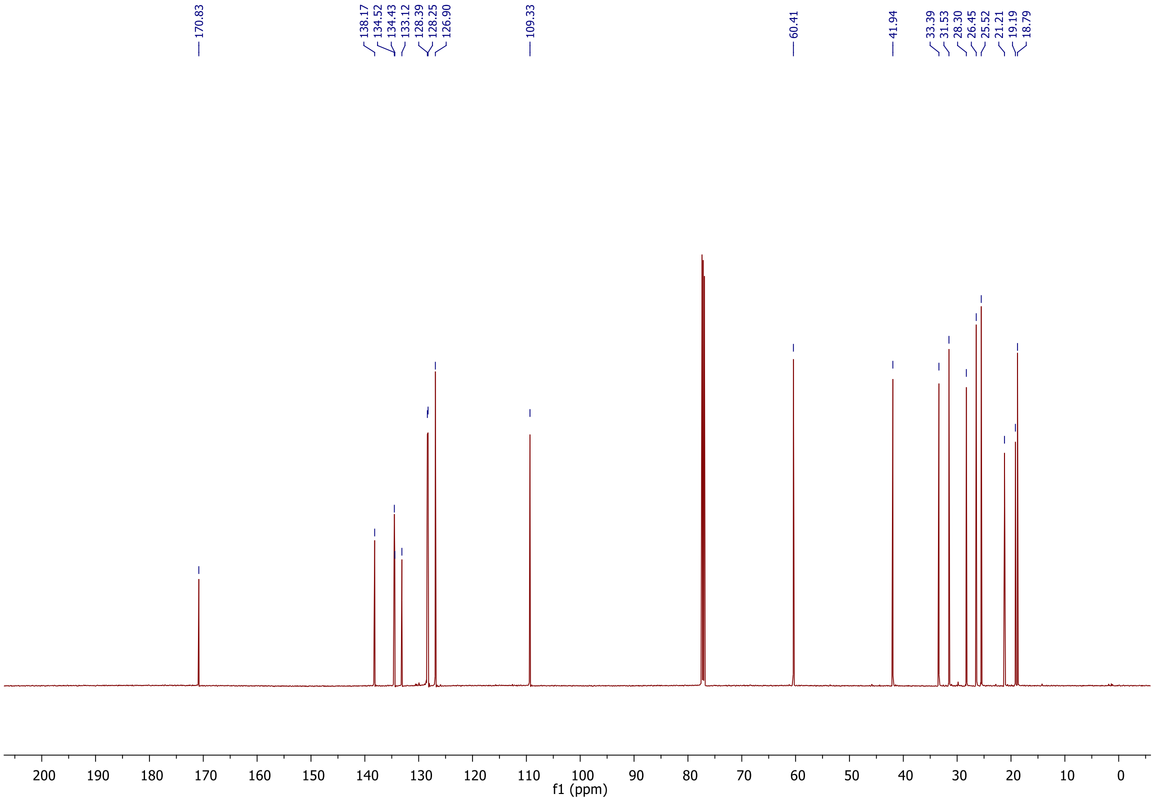

N-Cyclohexyl-2,4,6-trimethyl-N-vinylbenzamide (2o)

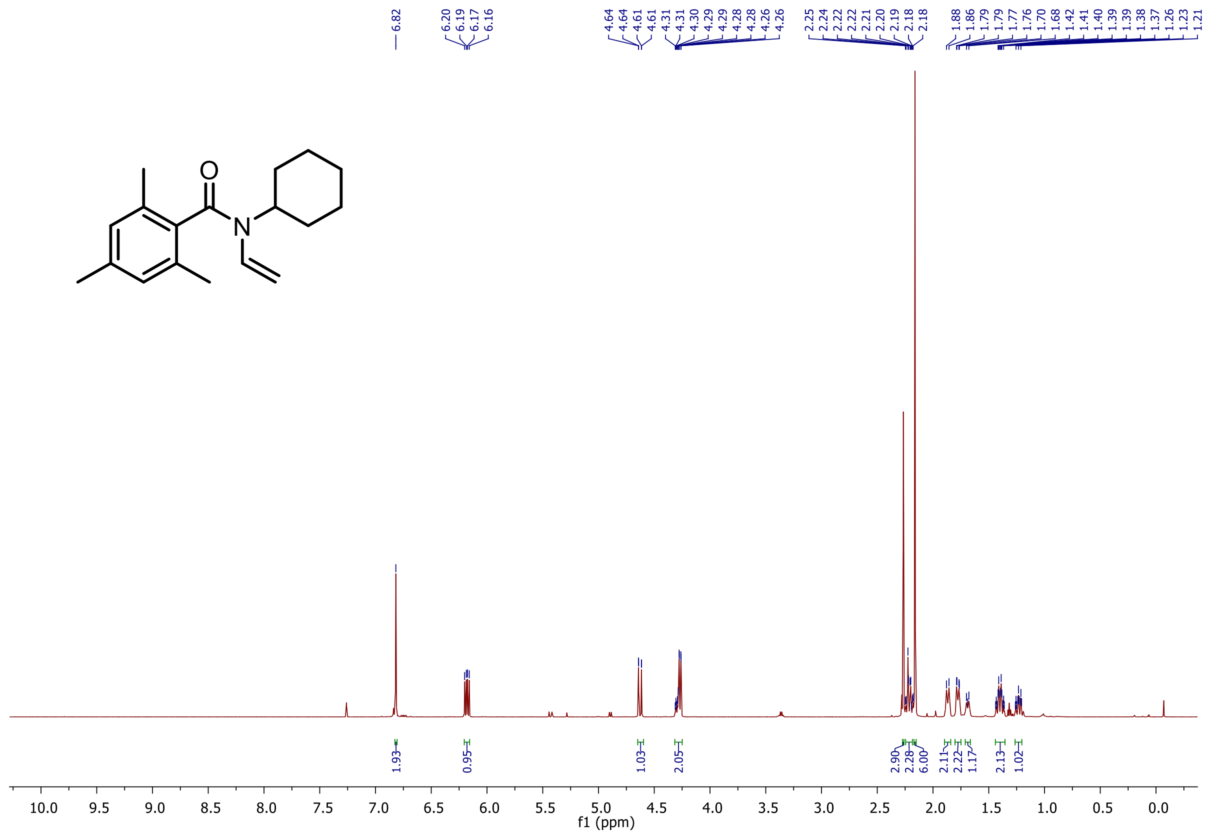

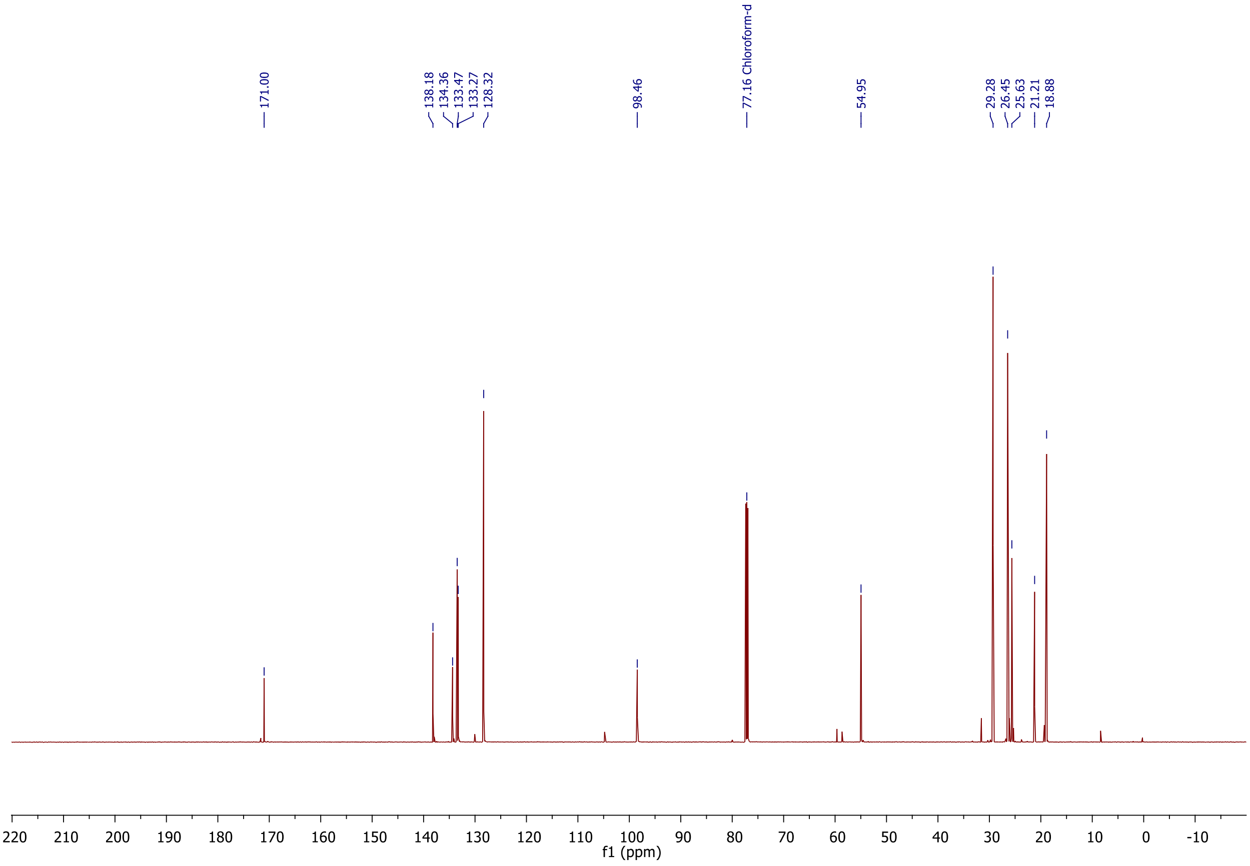

N-Butyl-2,4,6-trimethyl-N-vinylbenzamide (2p)

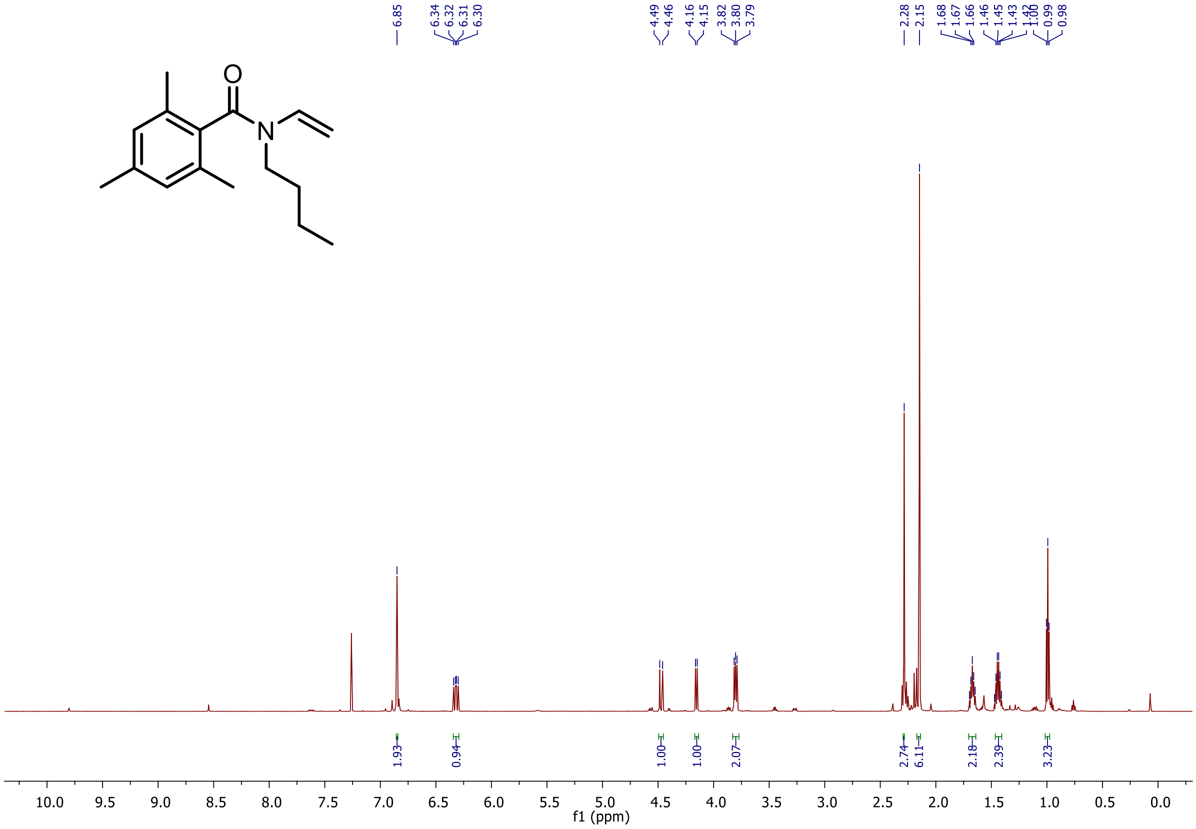

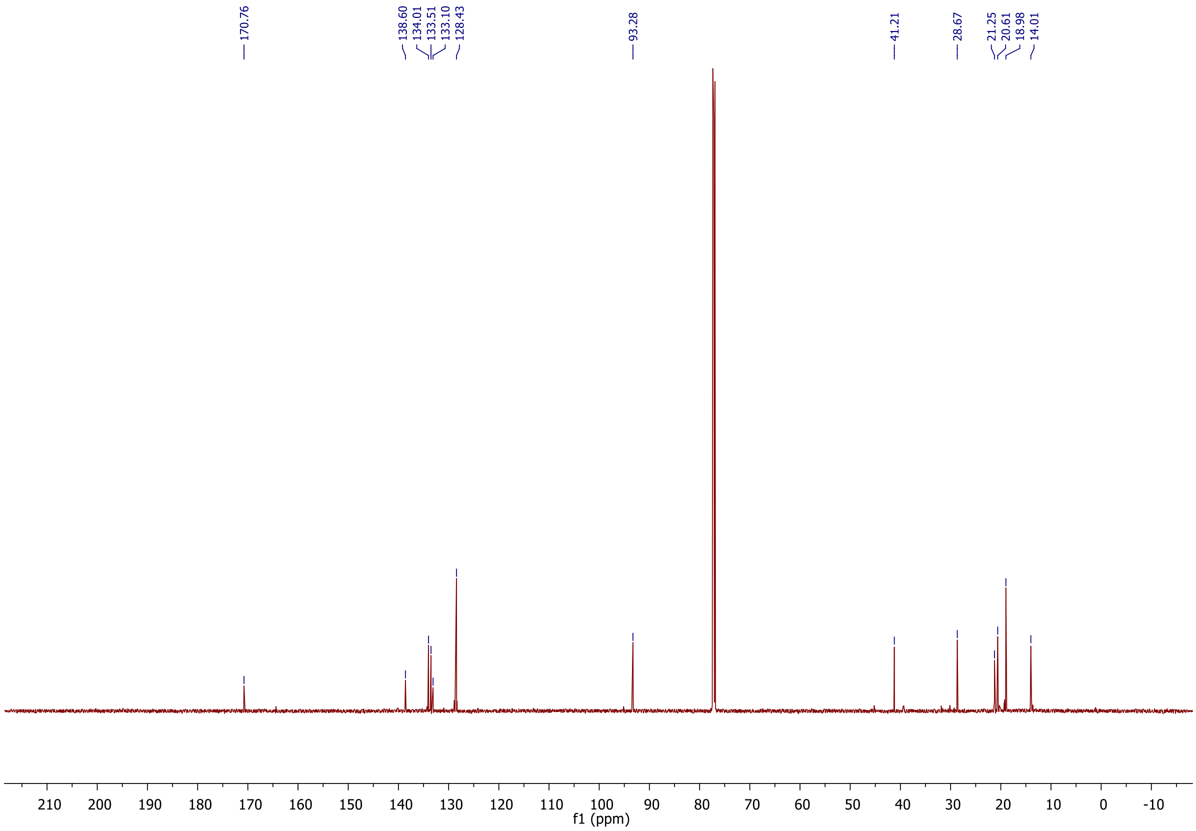

(E)-N-(But-1-en-1-yl)-N-ethyl-2,4,6-trimethylbenzamide (2p')

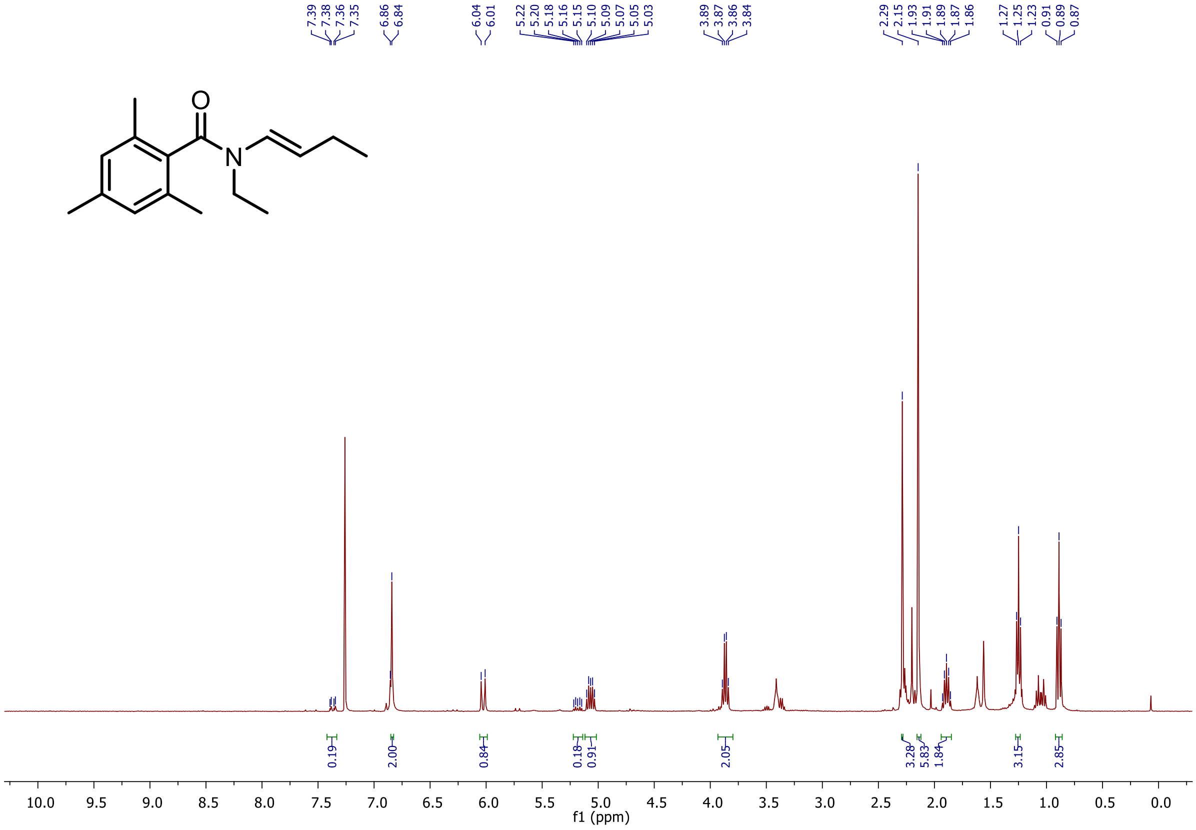

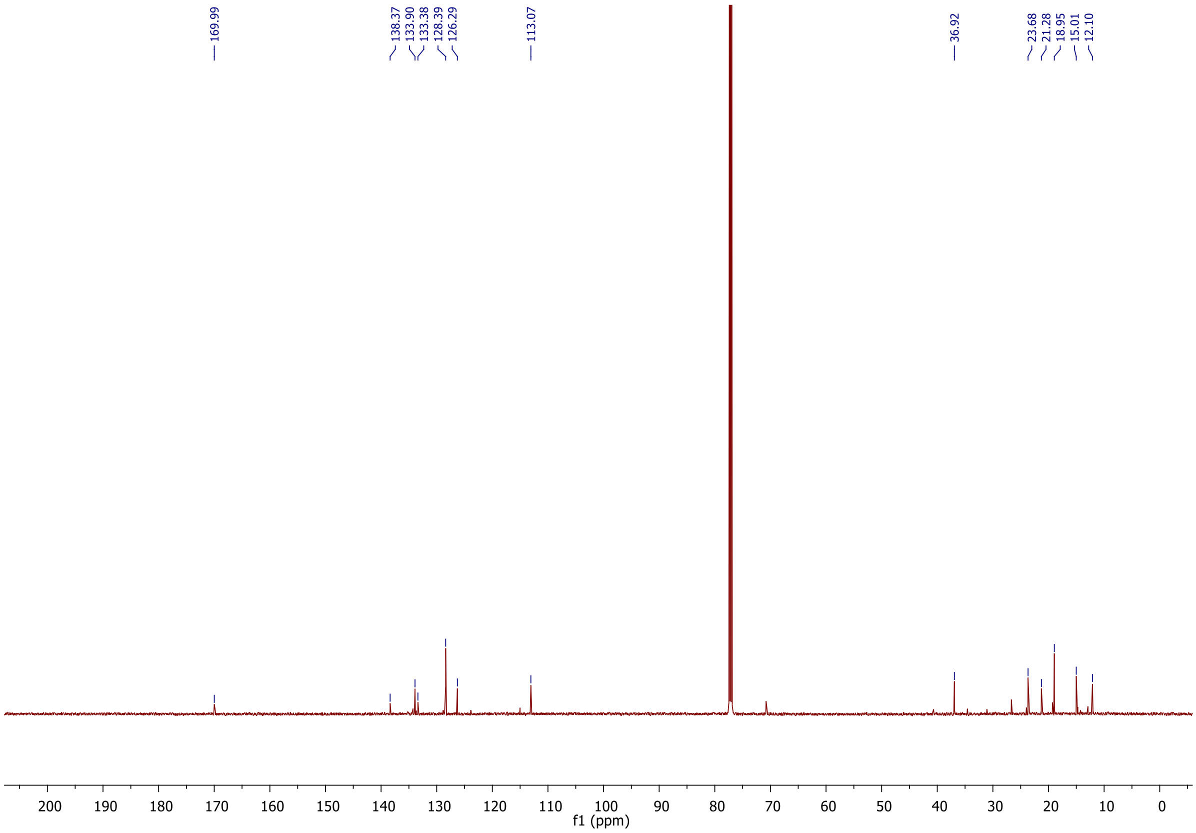

((3S,4R)-3-((Benzo[d][1,3]dioxol-5-yloxy)methyl)-4-(4-fluorophenyl)-3,4-dihydropyridin-1(2H)-yl)(mesityl)methanone (2q)

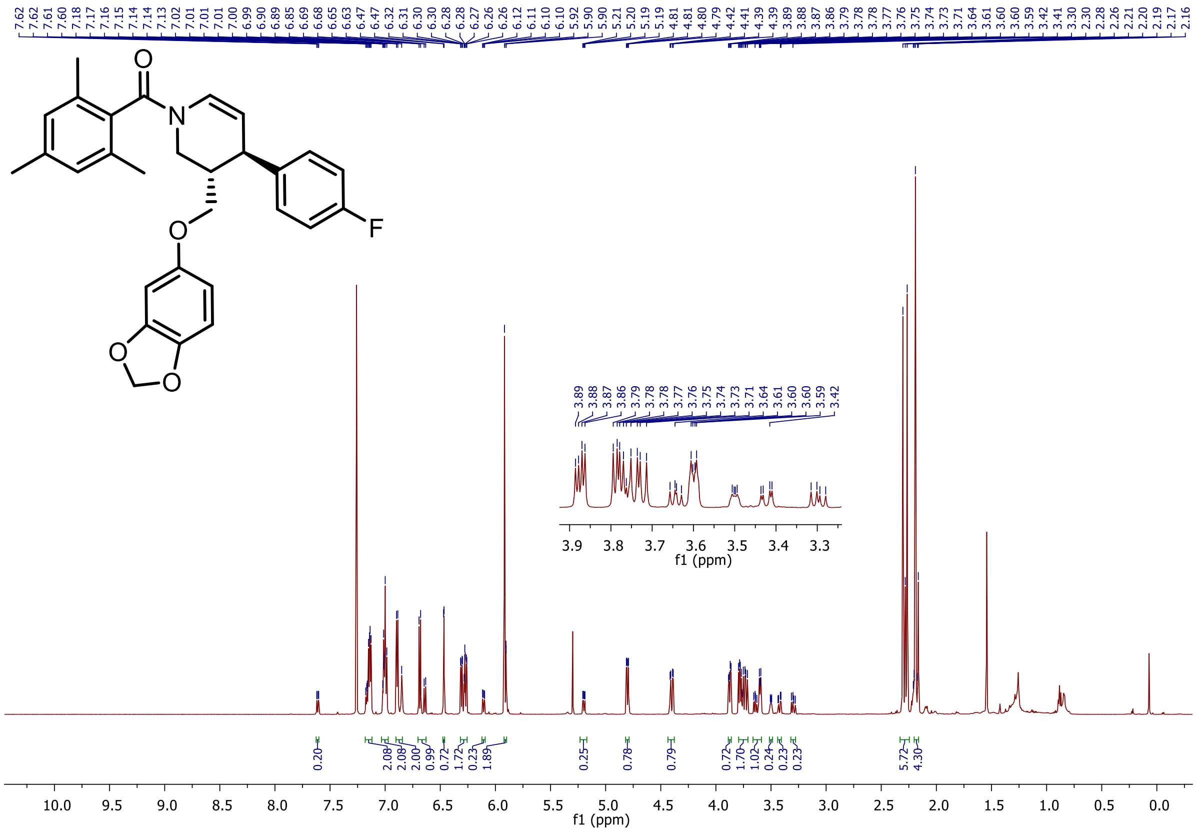

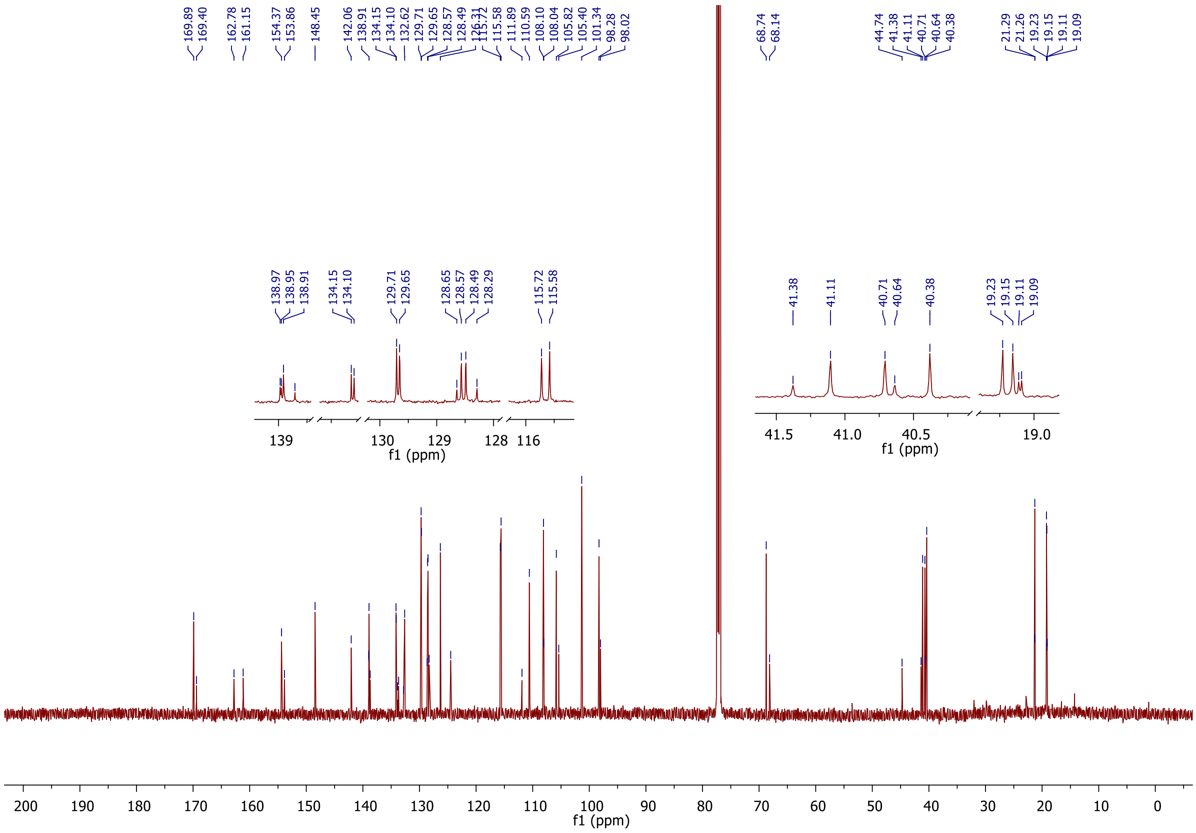

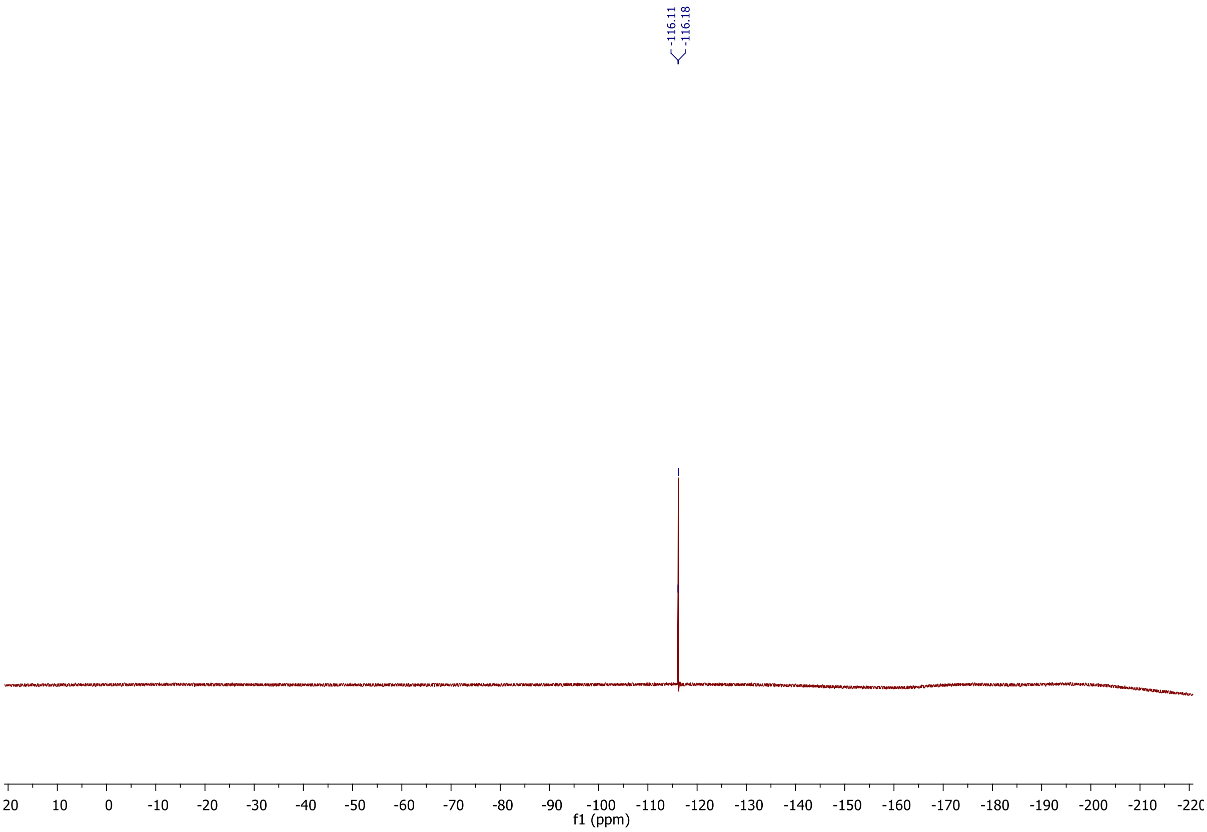

-116.11
-116.18
20 10 0 -10 -20 -30 -40 -50 -60 -70 -80 -90 -100 -110 -120 -130 -140 -150 -160 -170 -180 -190 -200 -210 -220
f1 (ppm)

(1-(3,4-Dimethoxybenzyl)-6,7-dimethoxyisoquinolin-2(1H)-yl)(mesityl)methanone (2r)

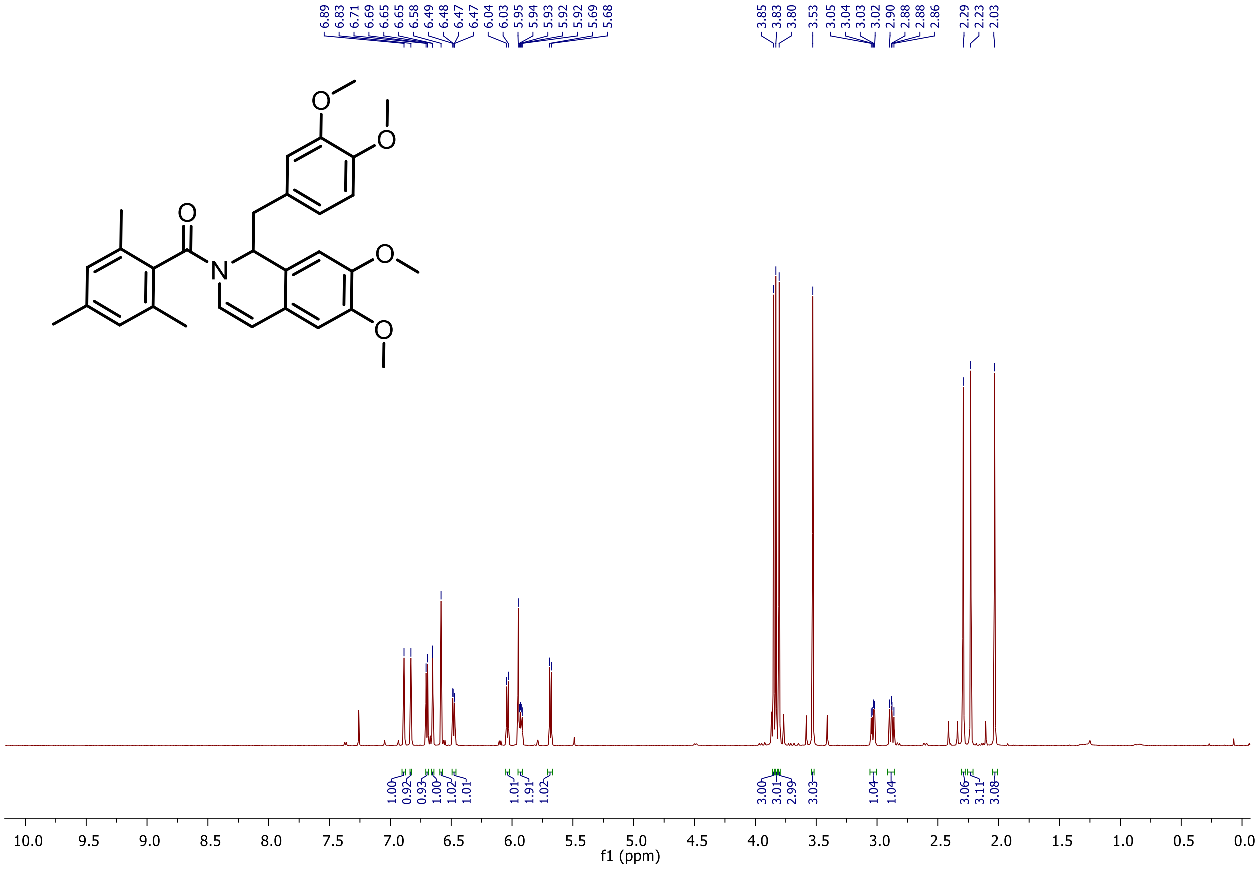

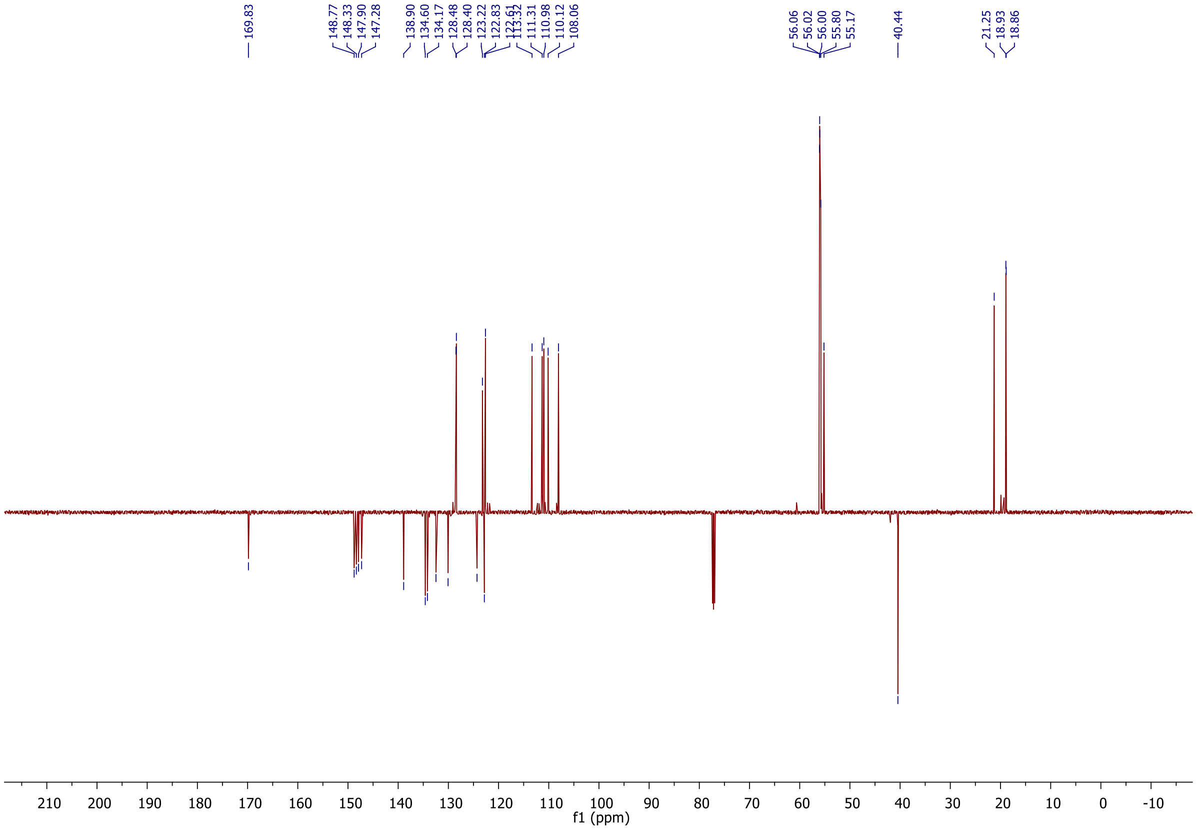

(2,3-Dihydro-1H-pyrrol-1-yl)(2,4,6-triisopropylphenyl)methanone (4a)

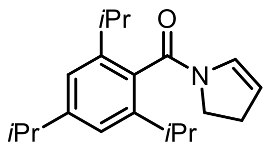

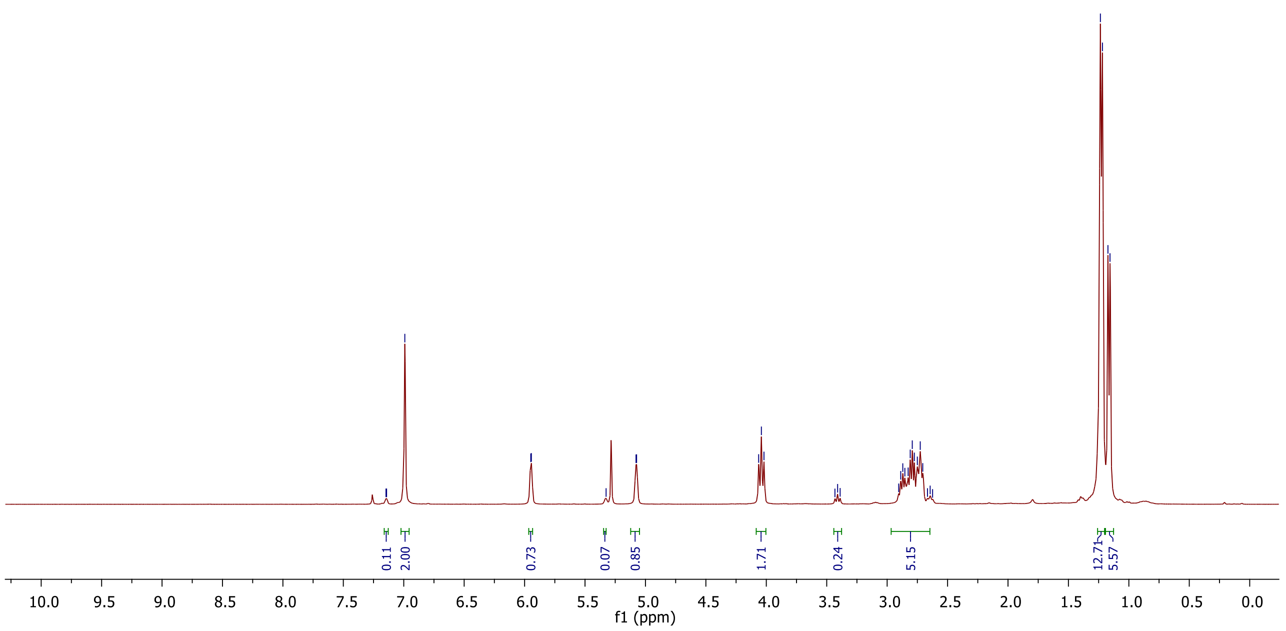

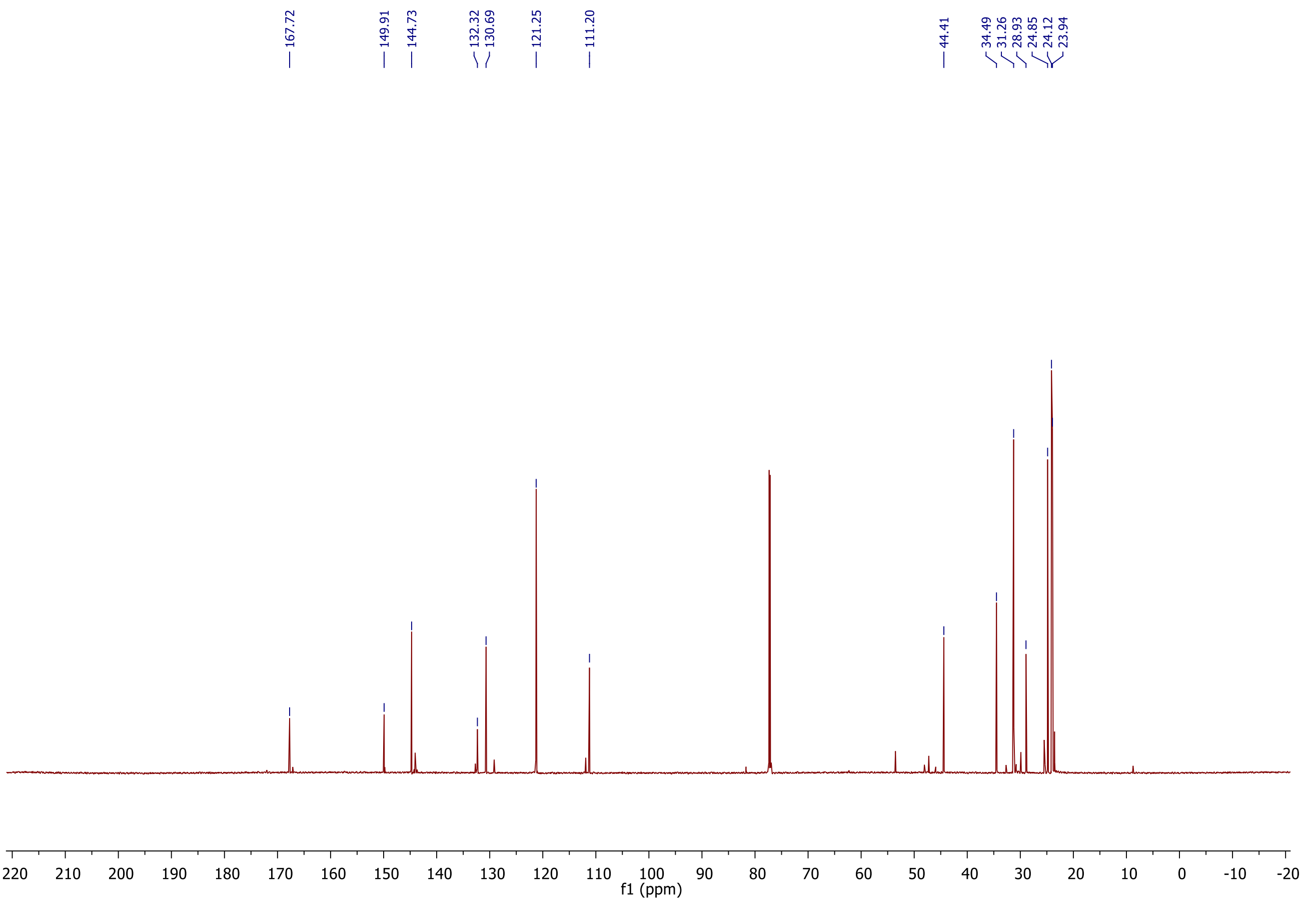

Anthracen-9-yl(2,3-dihydro-1H-pyrrol-1-yl)methanone (4b)

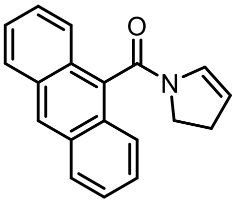

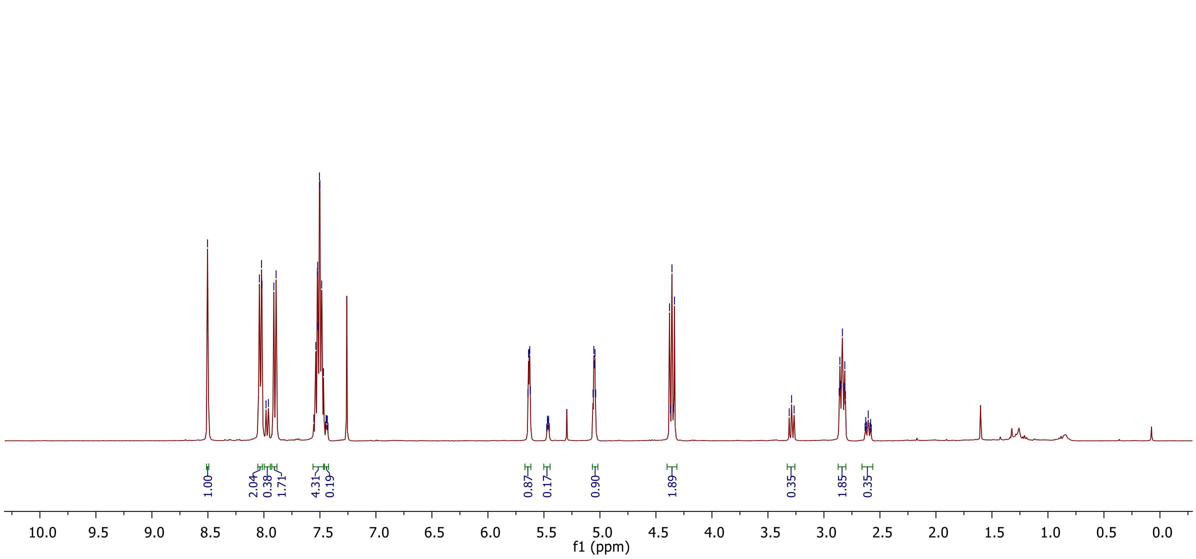

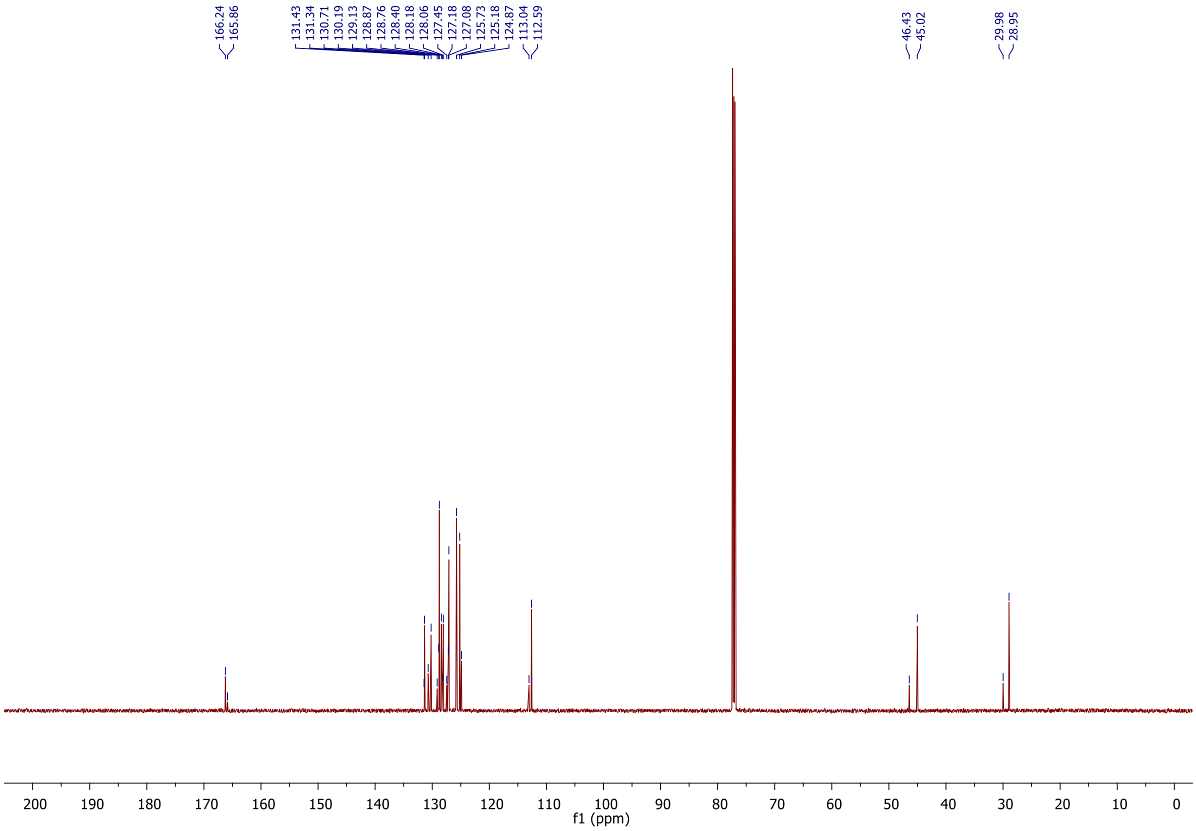

(2,3-Dihydro-1H-pyrrol-1-yl)(phenyl)methanone (4c)

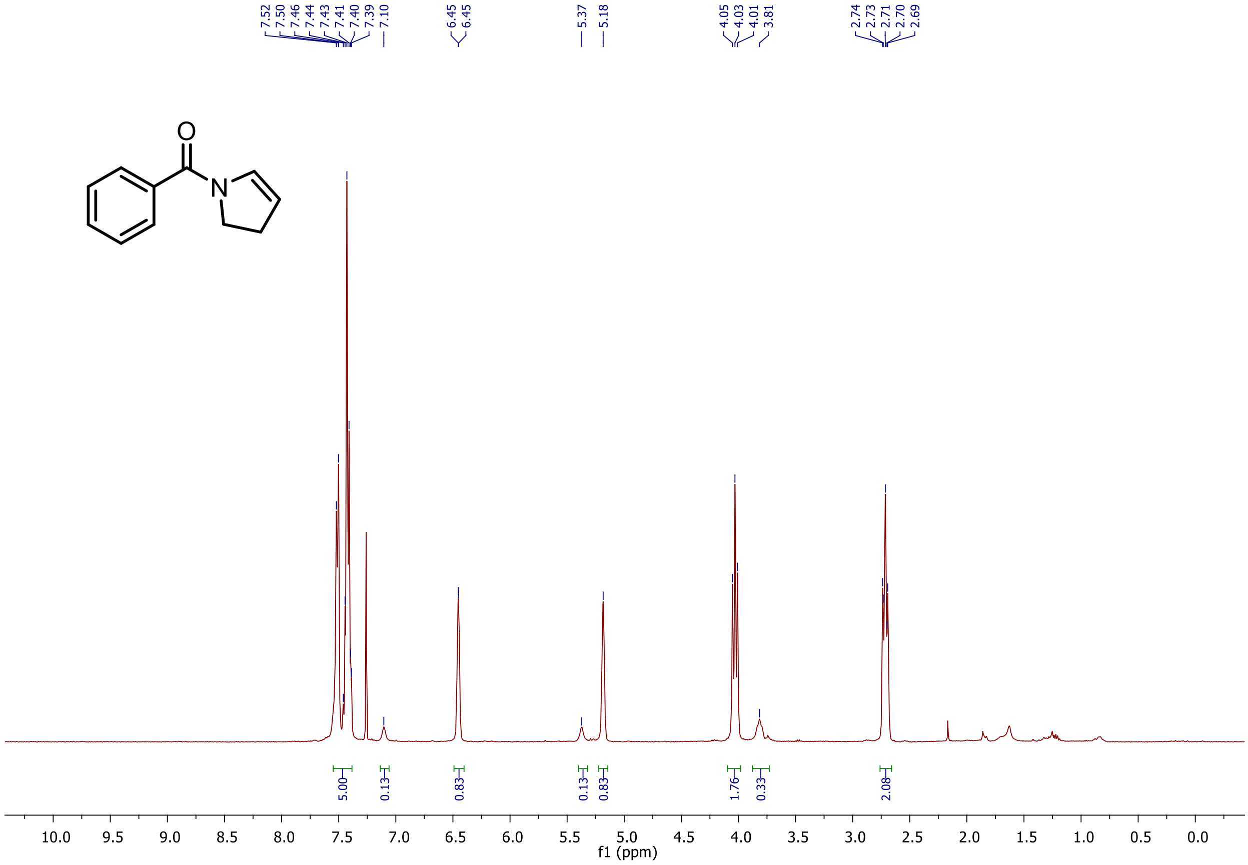

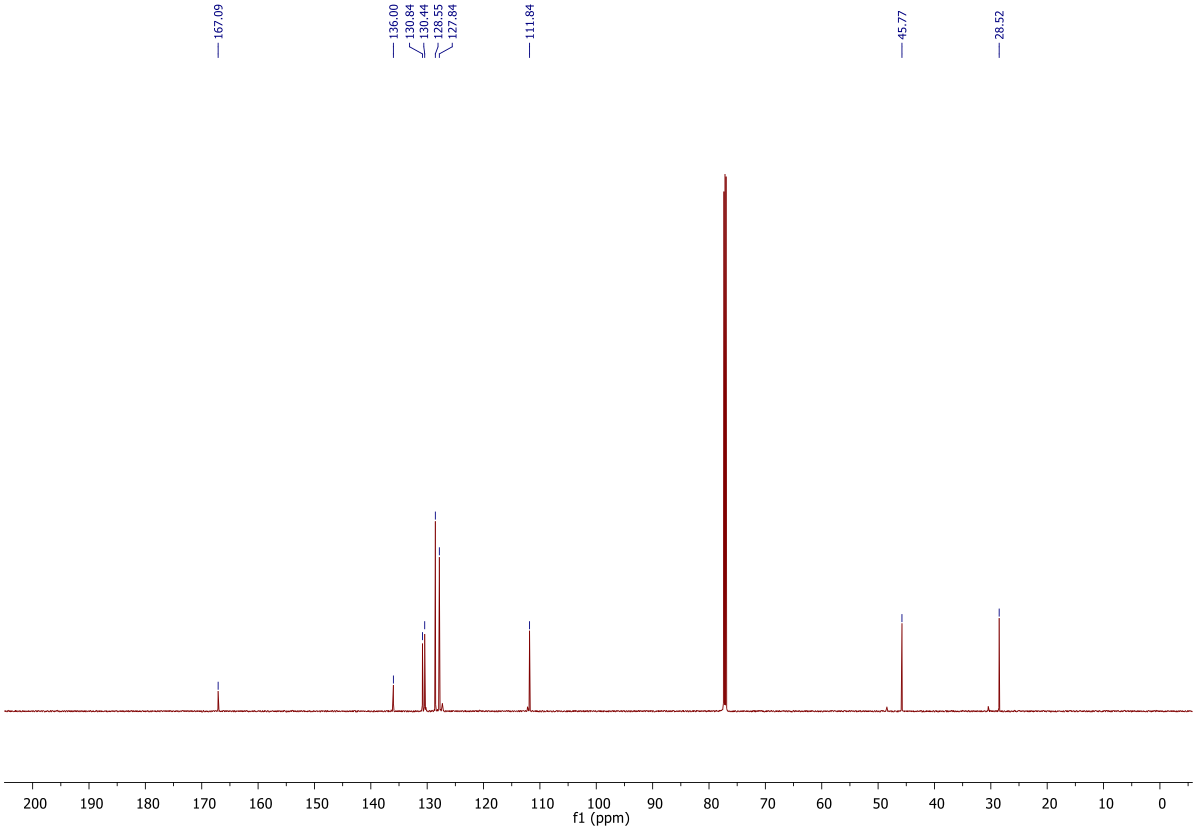

(3,4-Dihydropyridin-1(2H)-yl)(o-tolyl)methanone (4e)

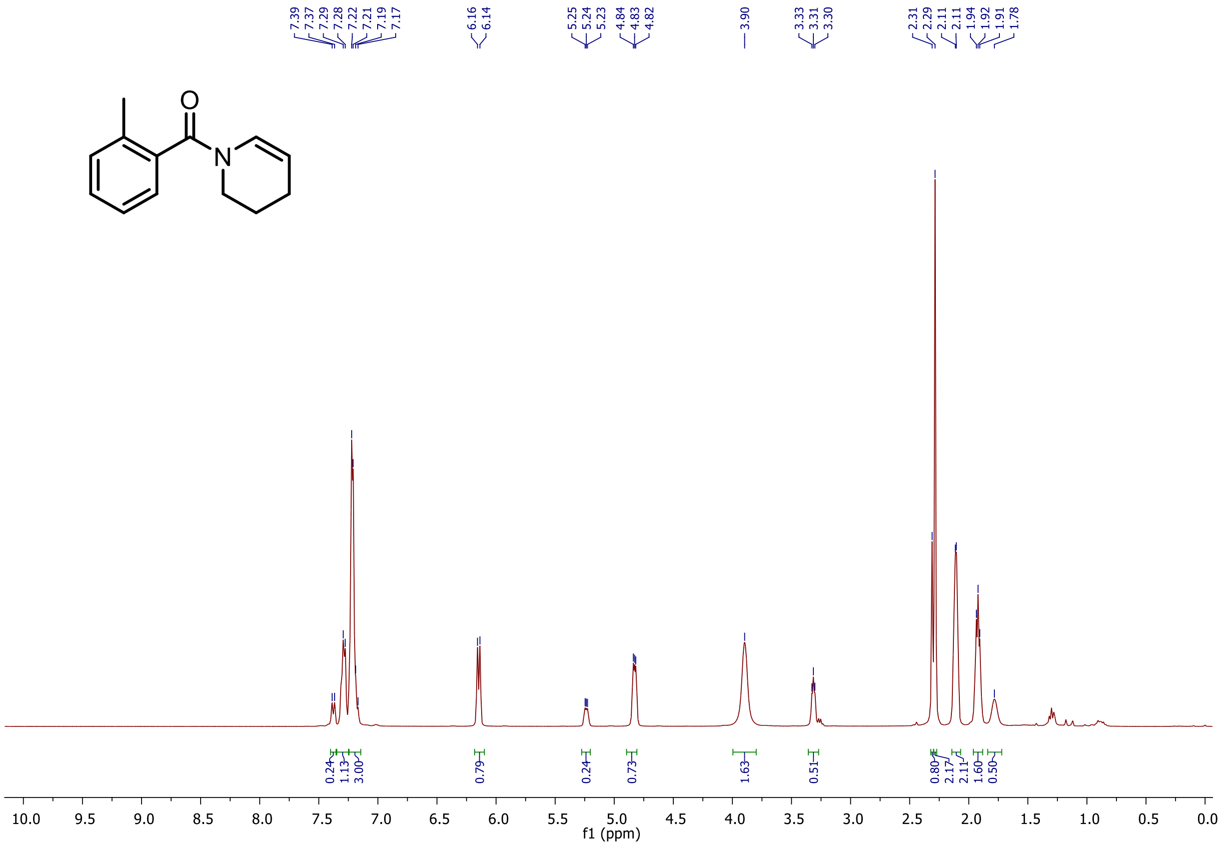

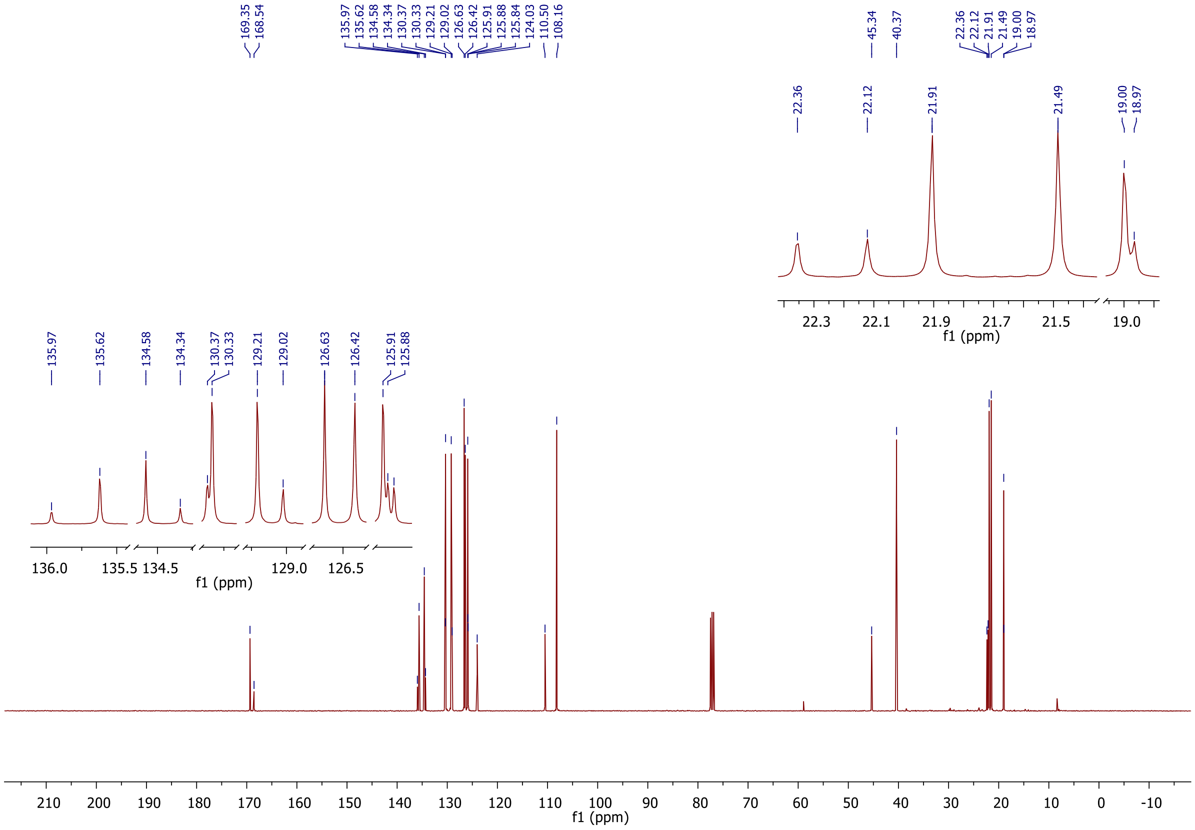

(3,4-Dihydropyridin-1(2H)-yl)(p-tolyl)methanone (4f)

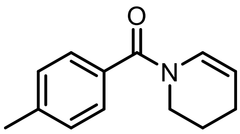

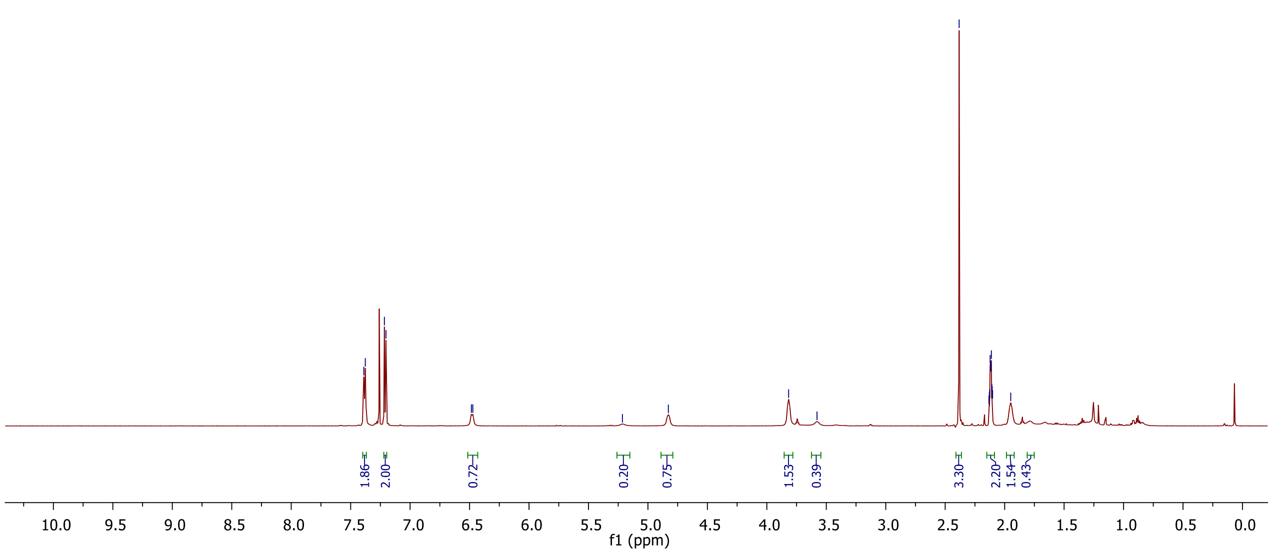

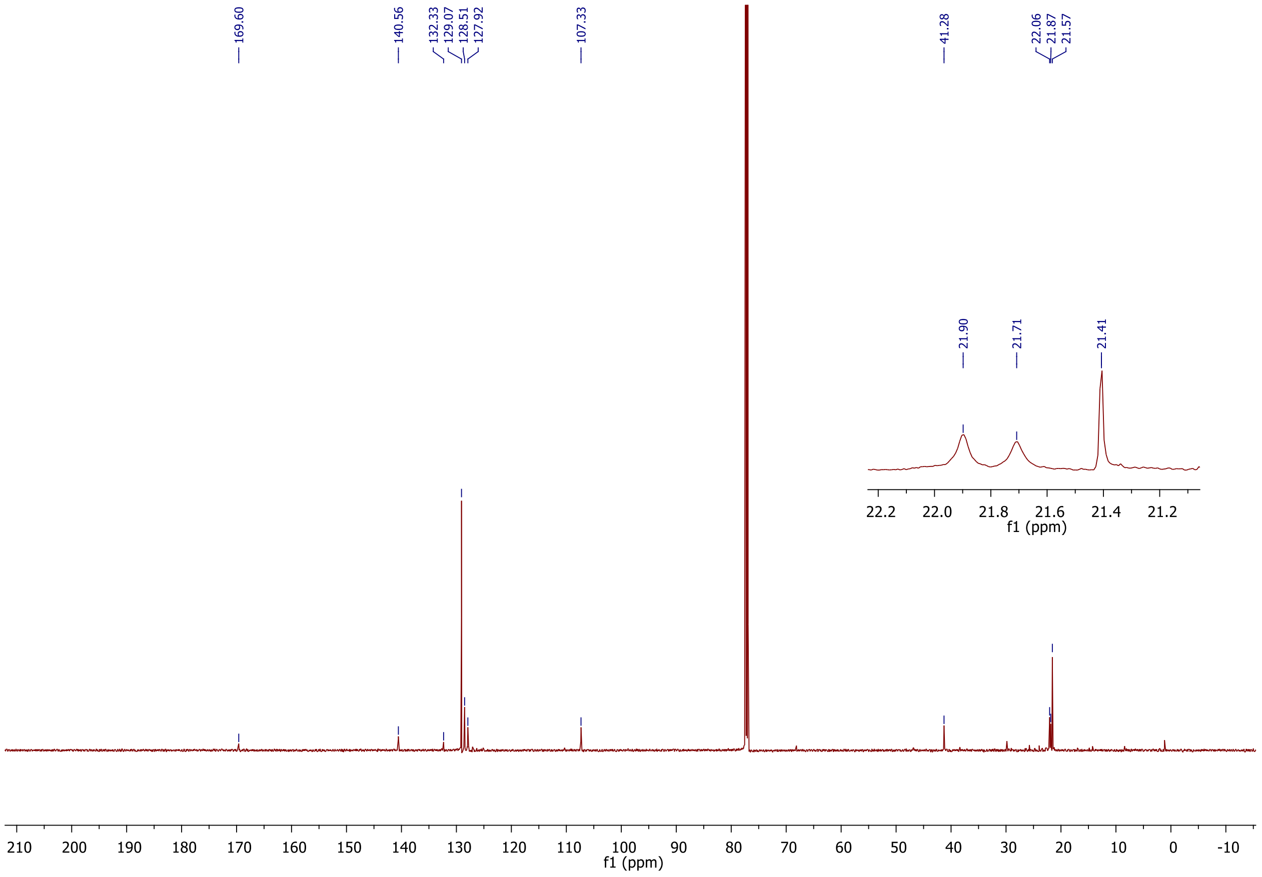

(3,4-Dihydropyridin-1(2H)-yl)(2-methoxyphenyl)methanone (4g)

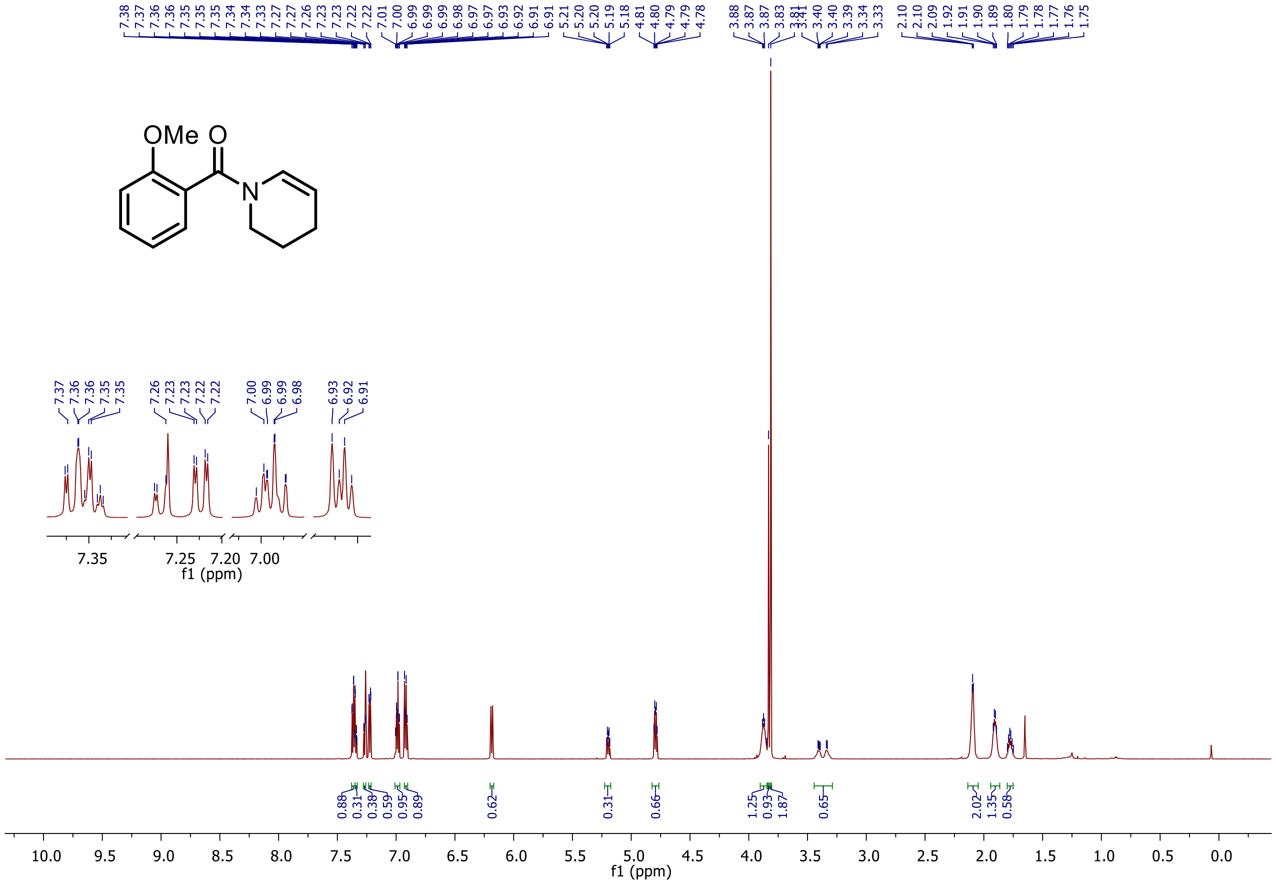

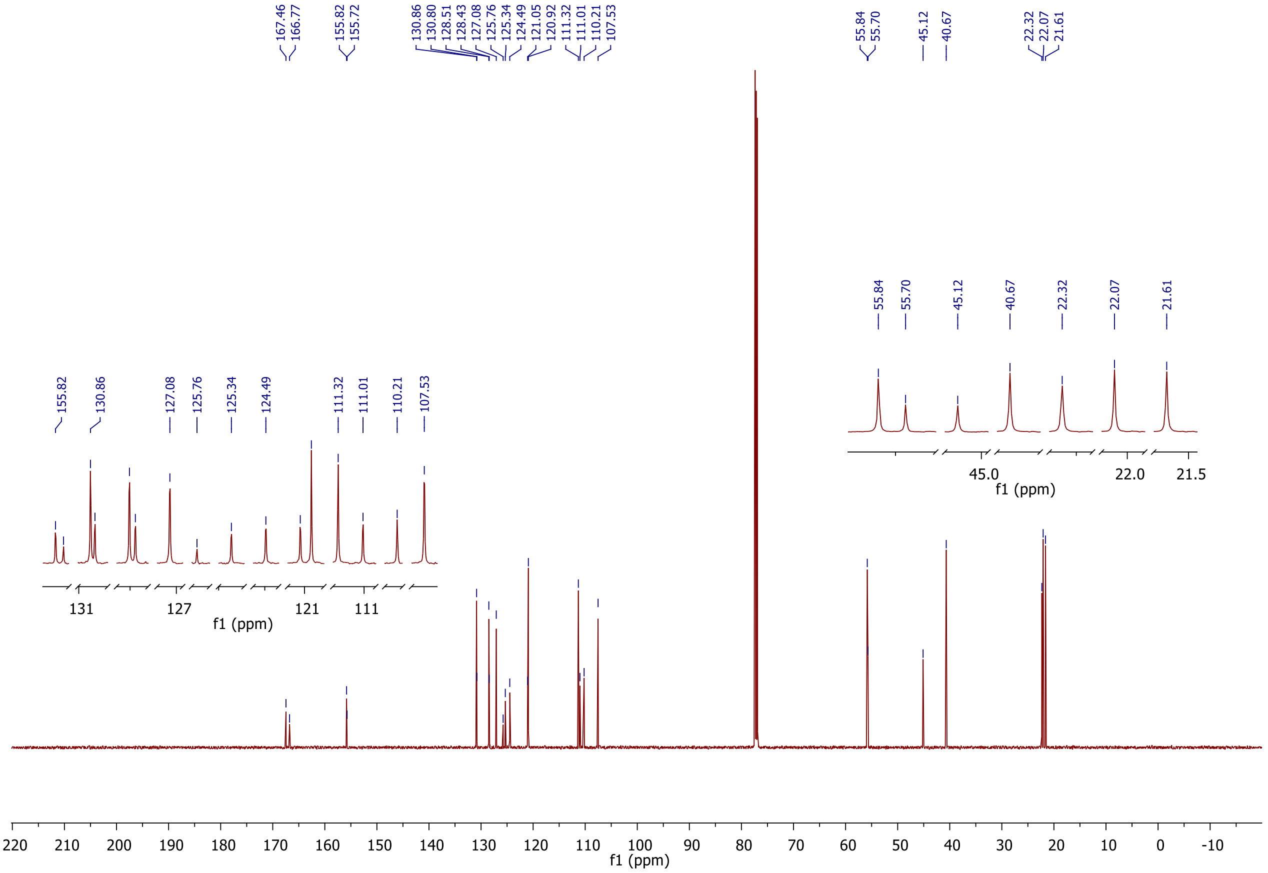

(3,4-Dihydropyridin-1(2H)-yl)(3-methoxyphenyl)methanone (4h)

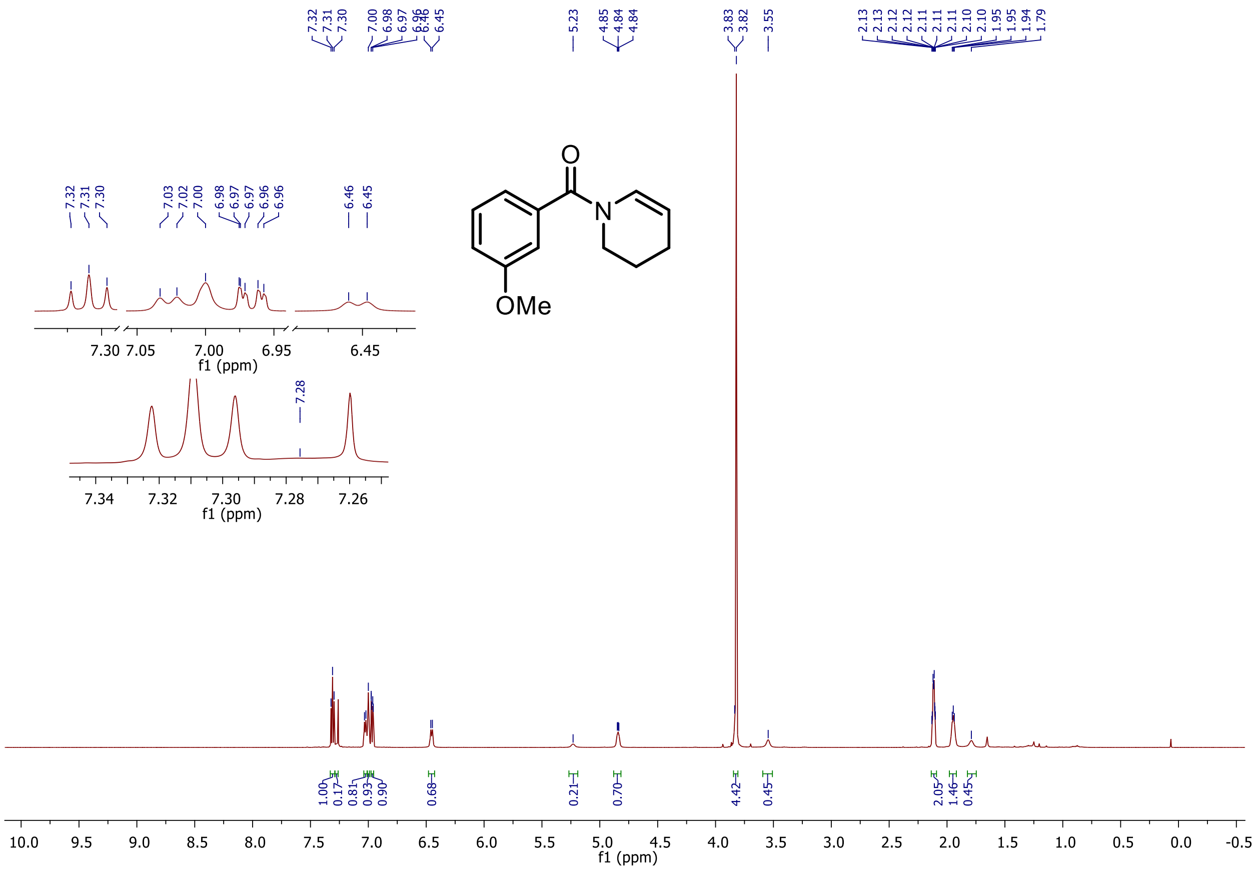

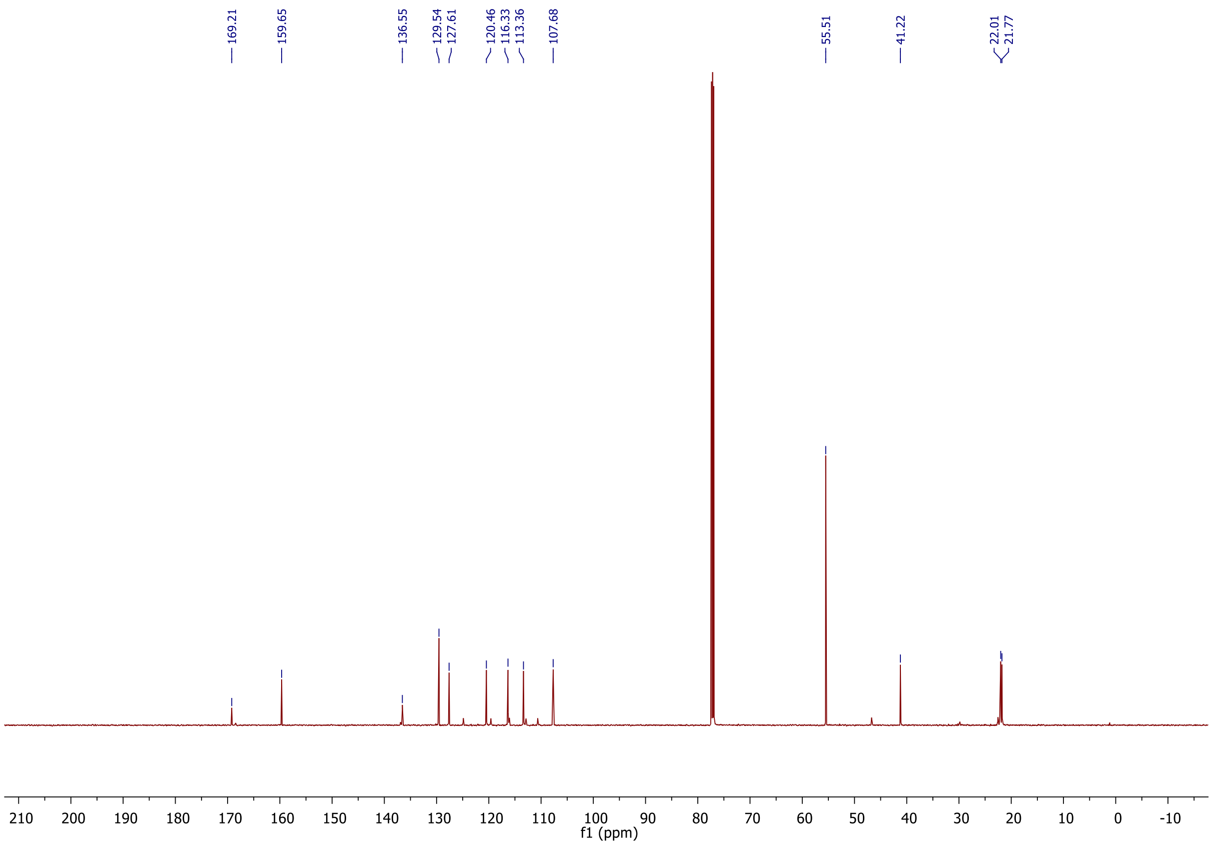

(3,4-Dihydropyridin-1(2H)-yl)(4-methoxyphenyl)methanone (4i)

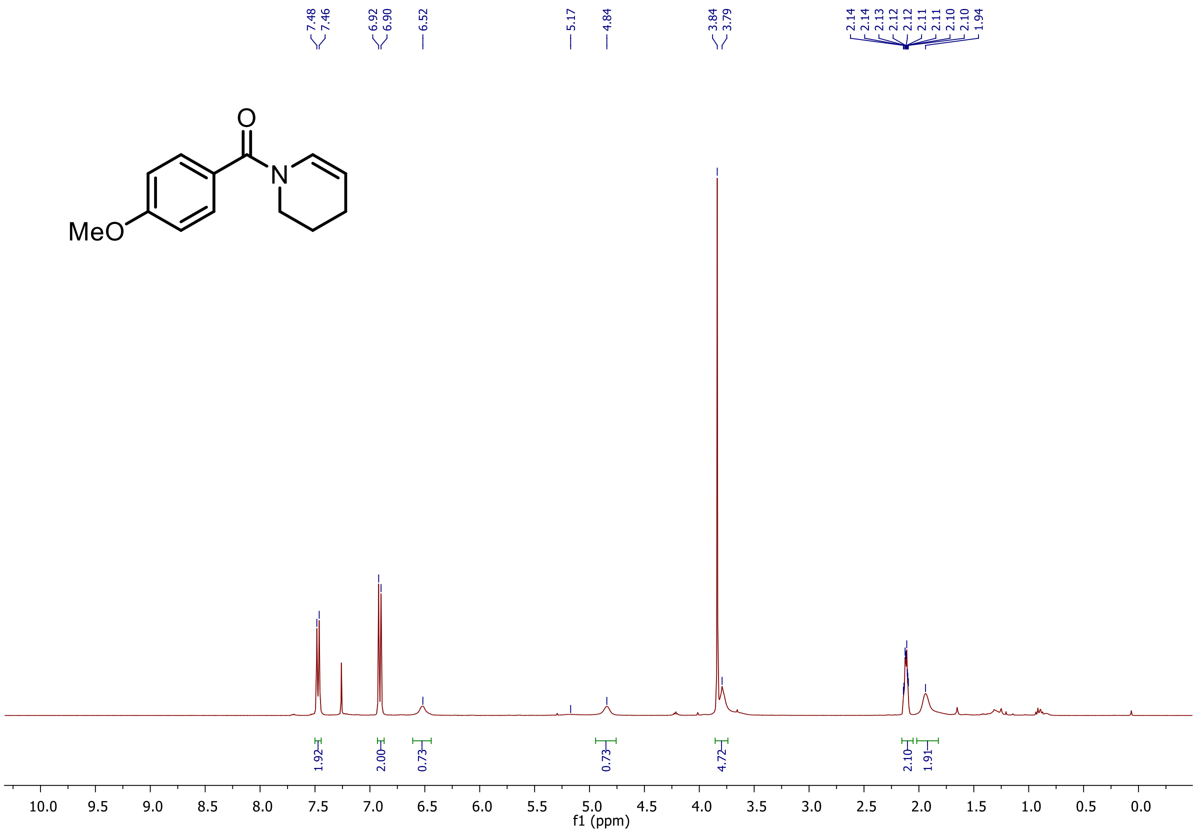

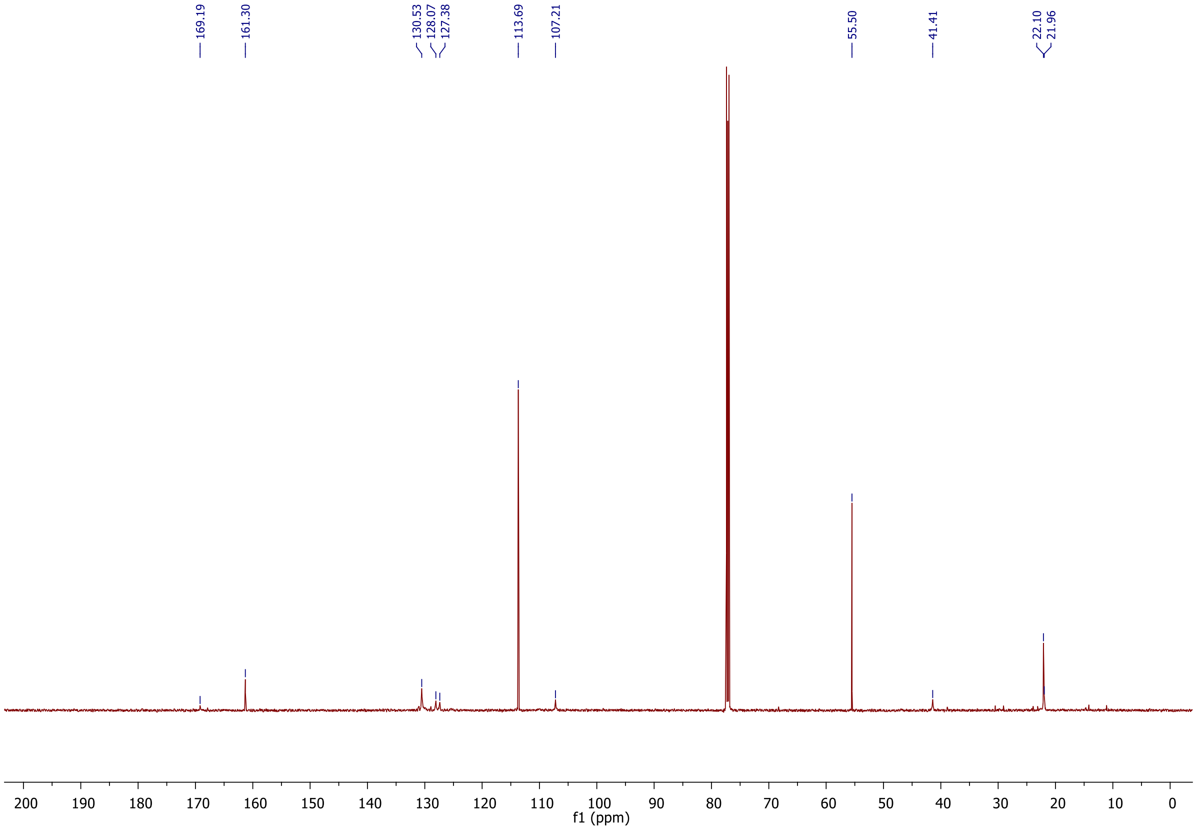

(2,3-Dihydro-1H-pyrrol-1-yl)(3,4,5-trimethoxyphenyl)methanone (4j)

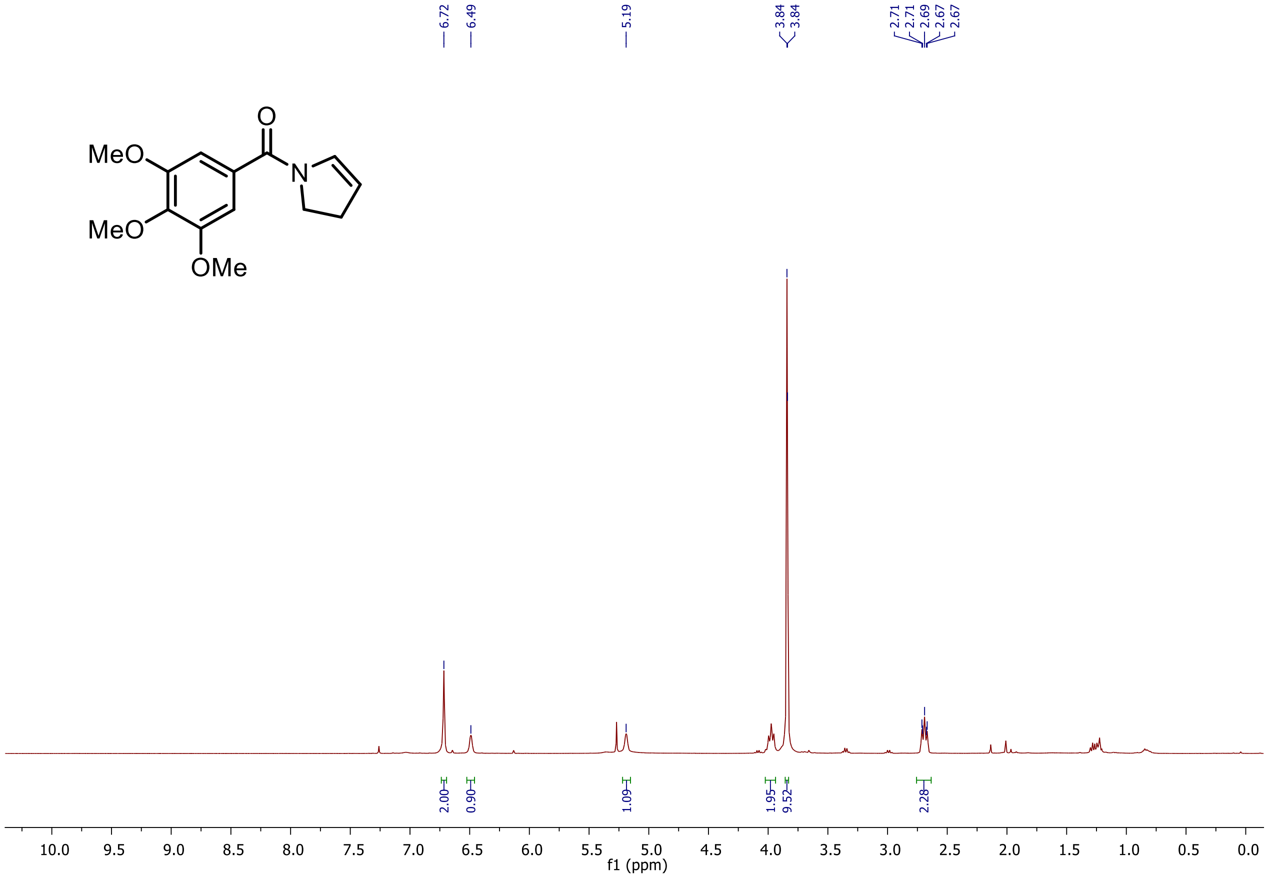

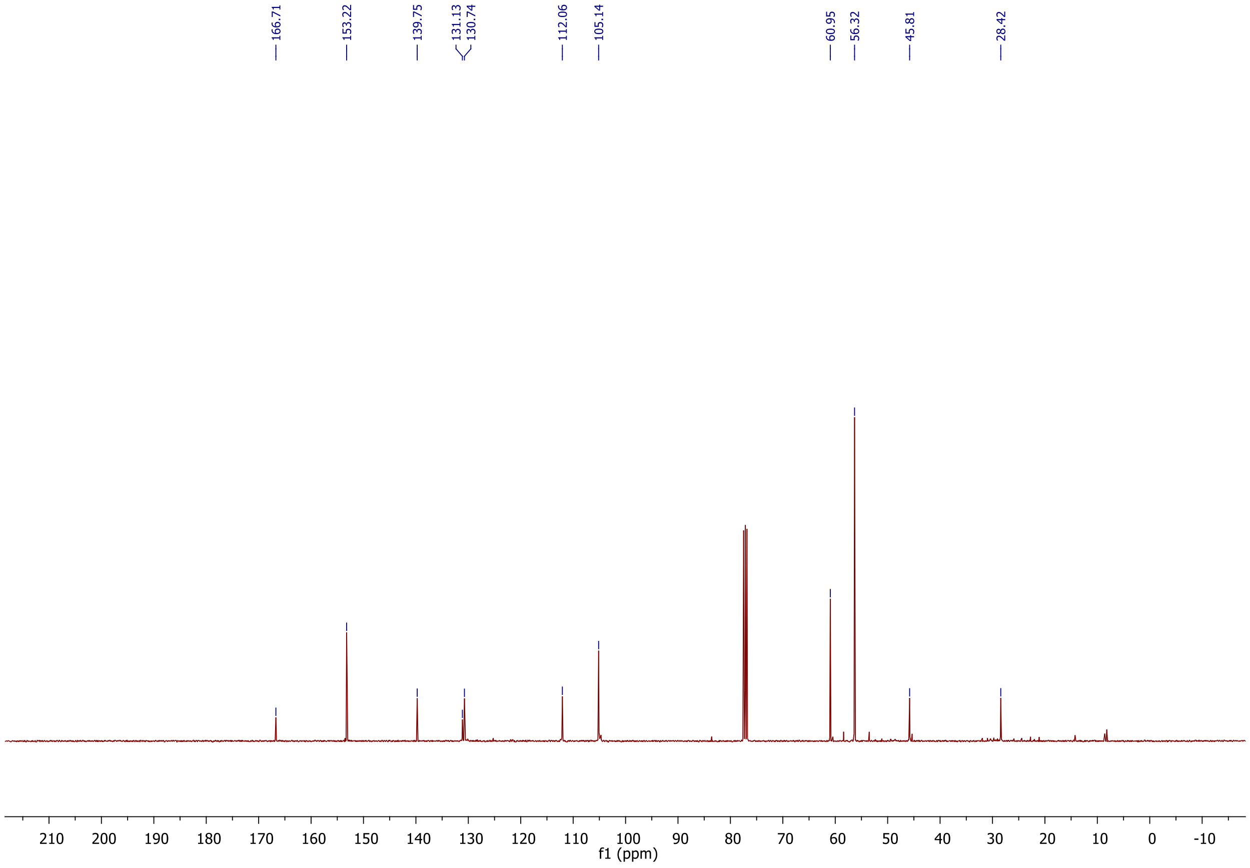

(3,4-Dihydropyridin-1(2H)-yl)(3,4,5-trimethoxyphenyl)methanone (4k)

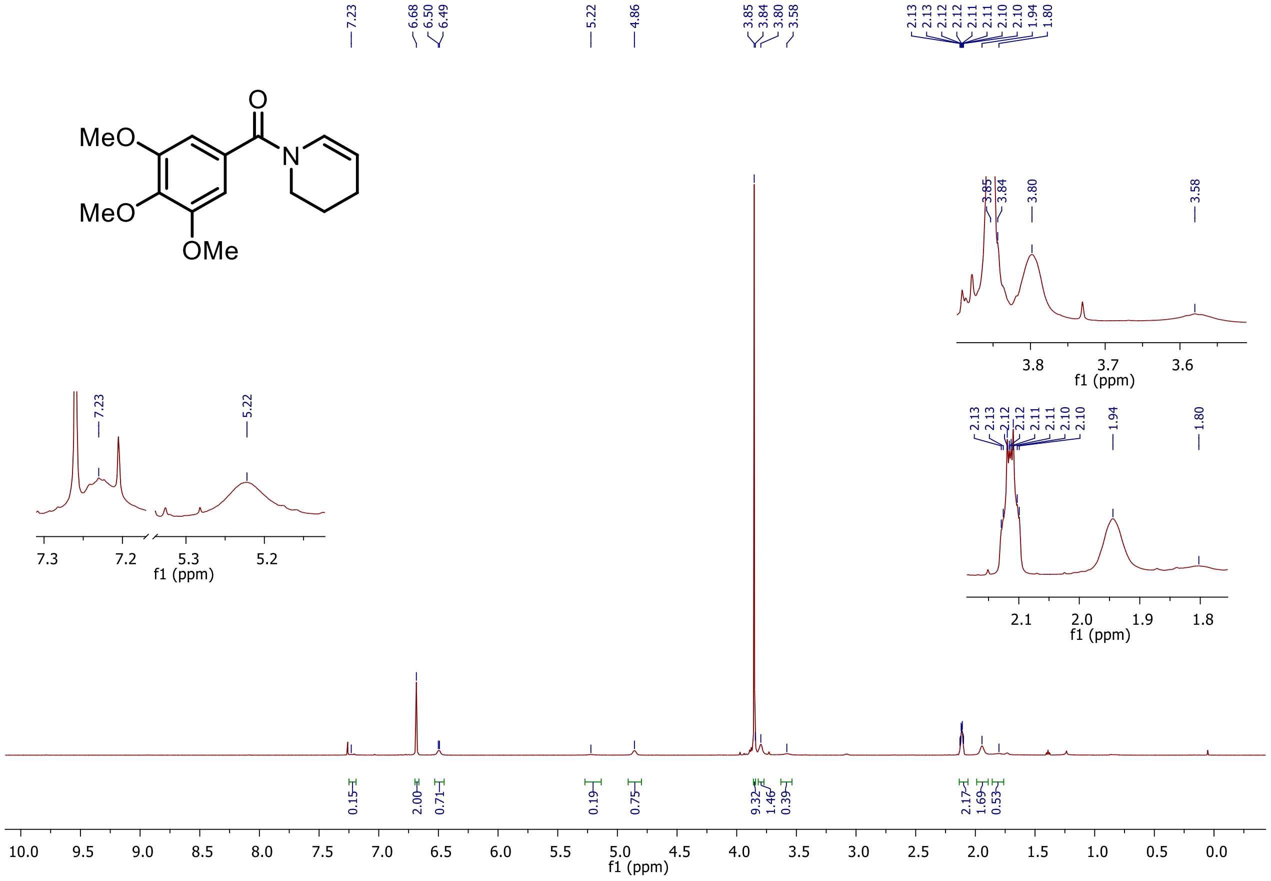

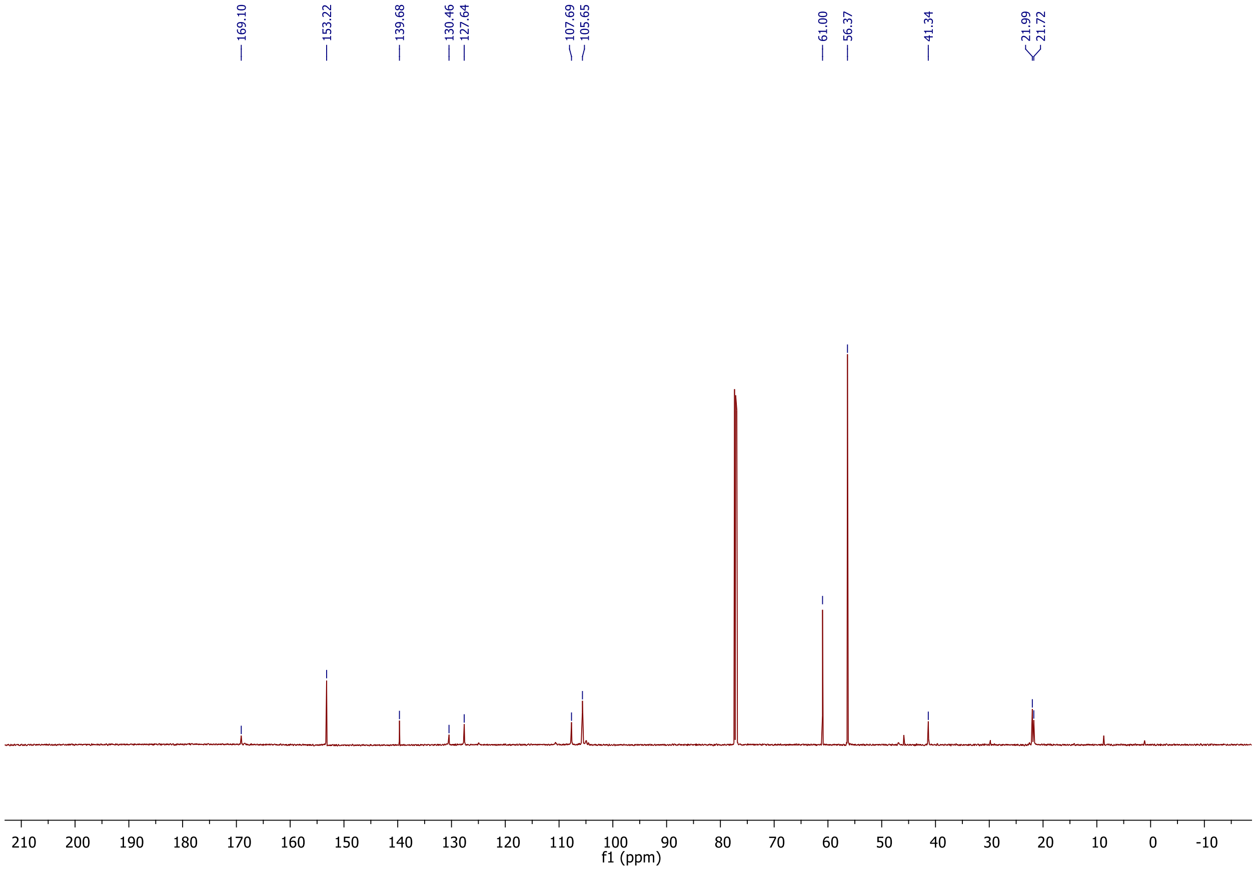

Benzo[d][1,3]dioxol-5-yl(3,4-dihydropyridin-1(2H)-yl)methanone (4l)

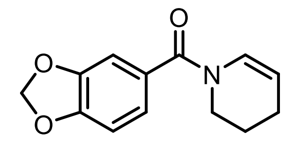

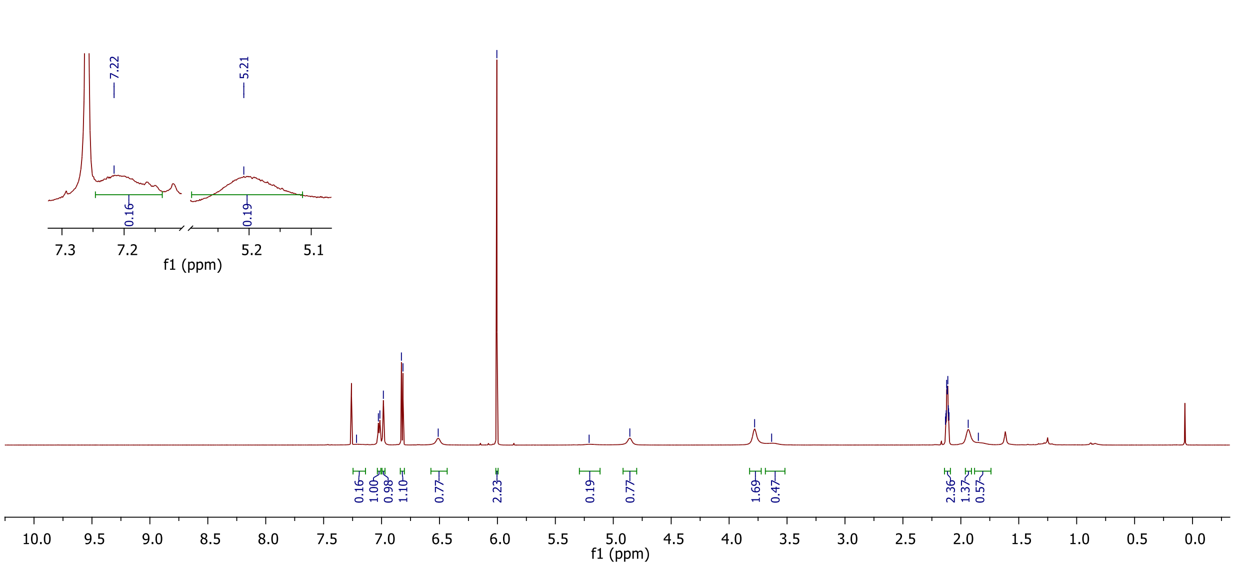

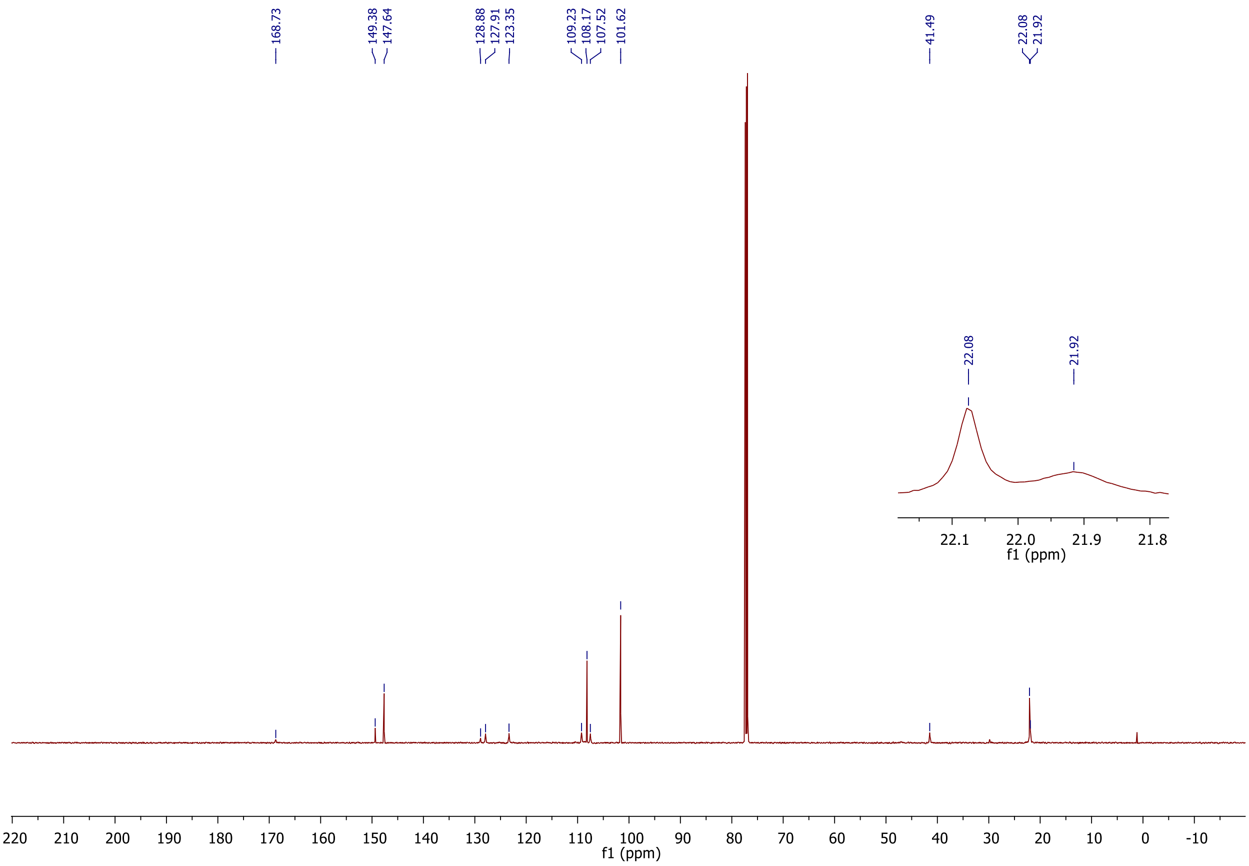

(2-Bromo-5-methoxyphenyl)(2,3-dihydro-1H-pyrrol-1-yl)methanone (4m)

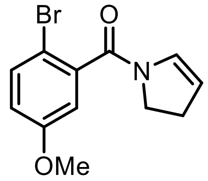

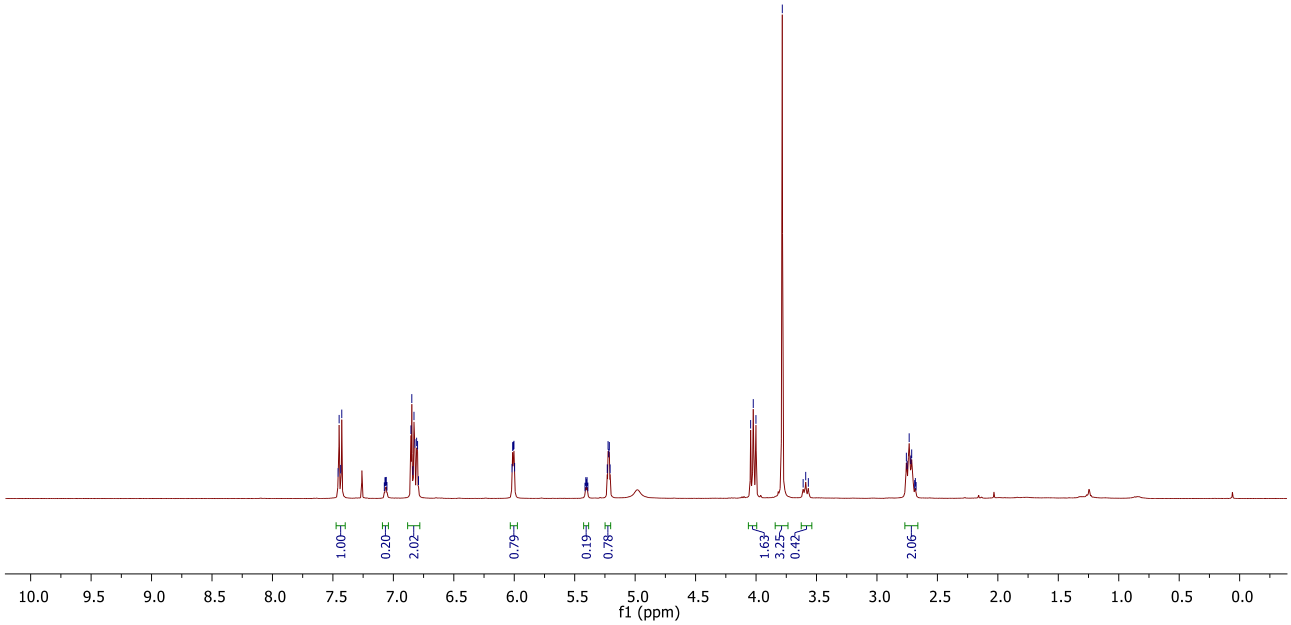

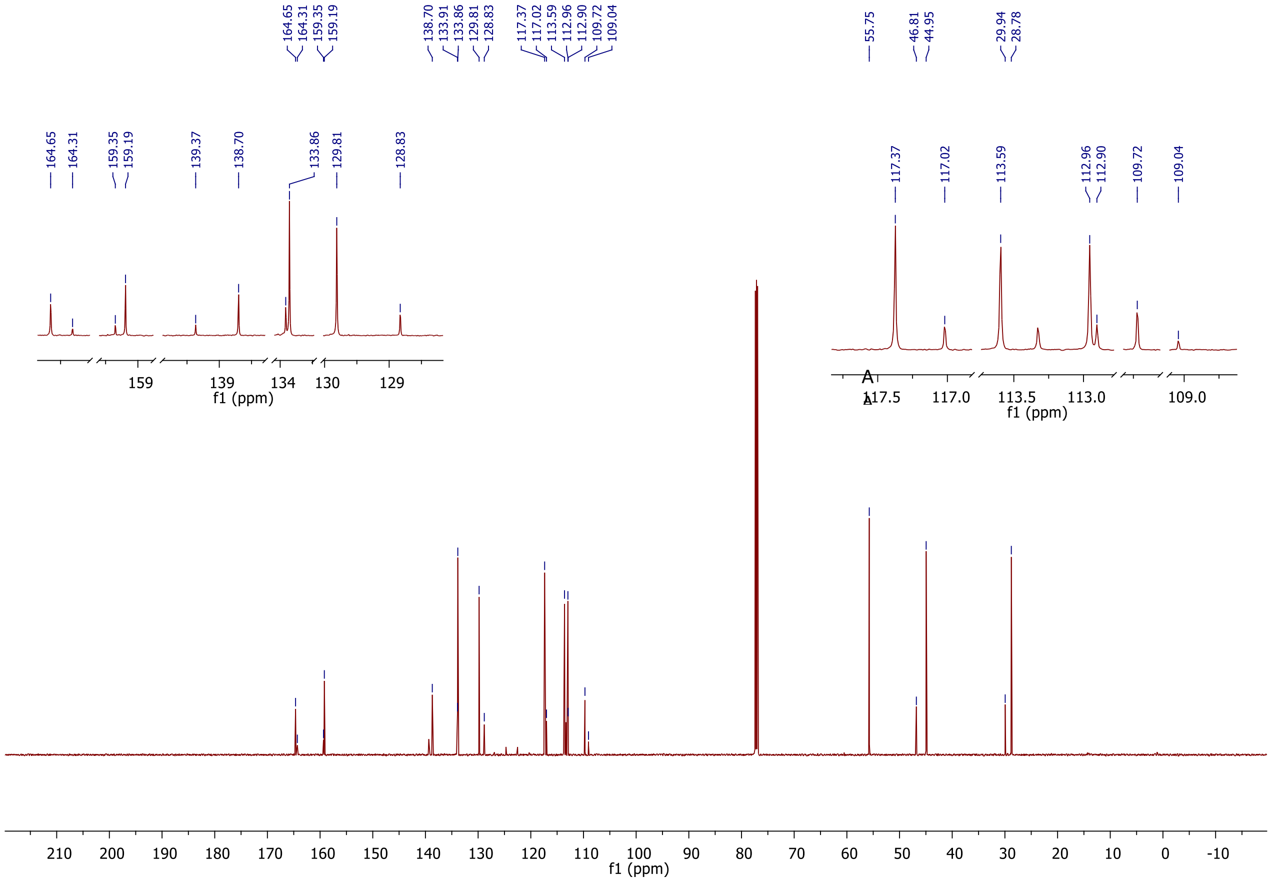

(2,3-Dihydro-1H-pyrrol-1-yl)(4-fluorophenyl)methanone (4n)

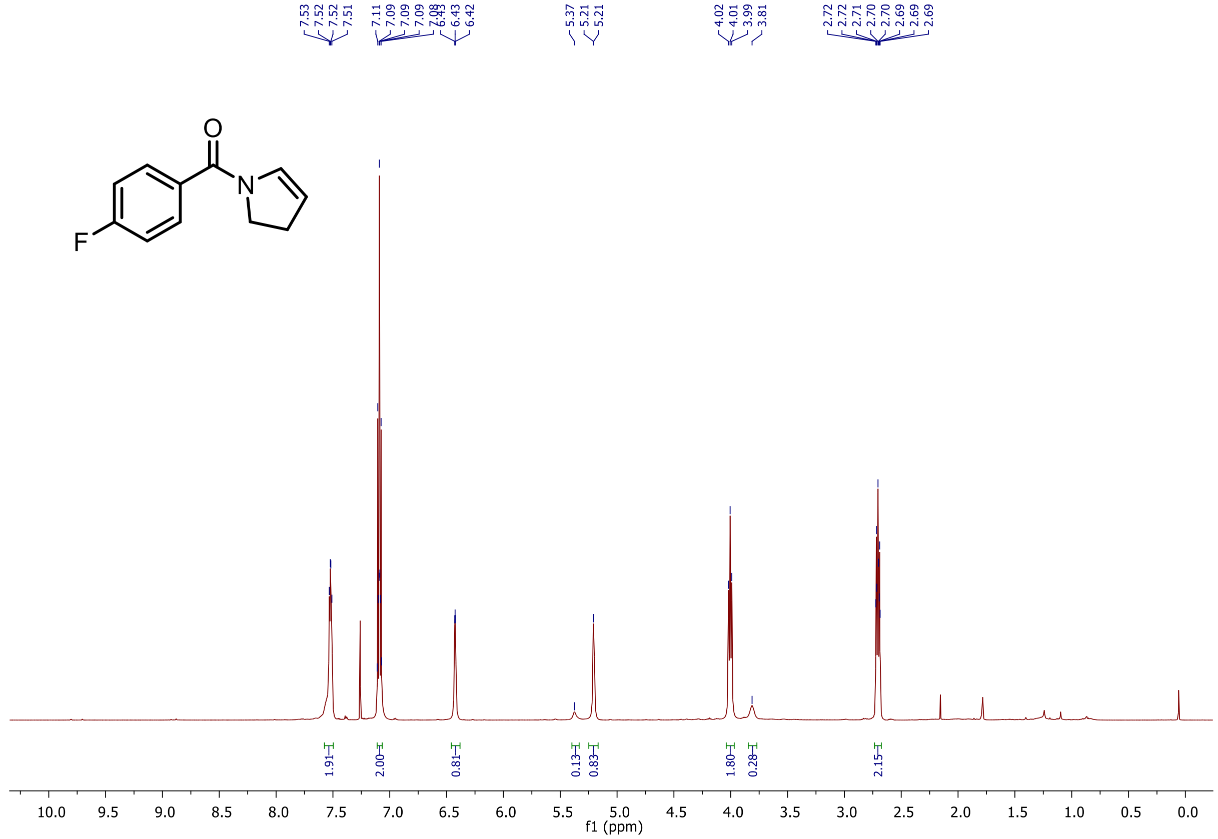

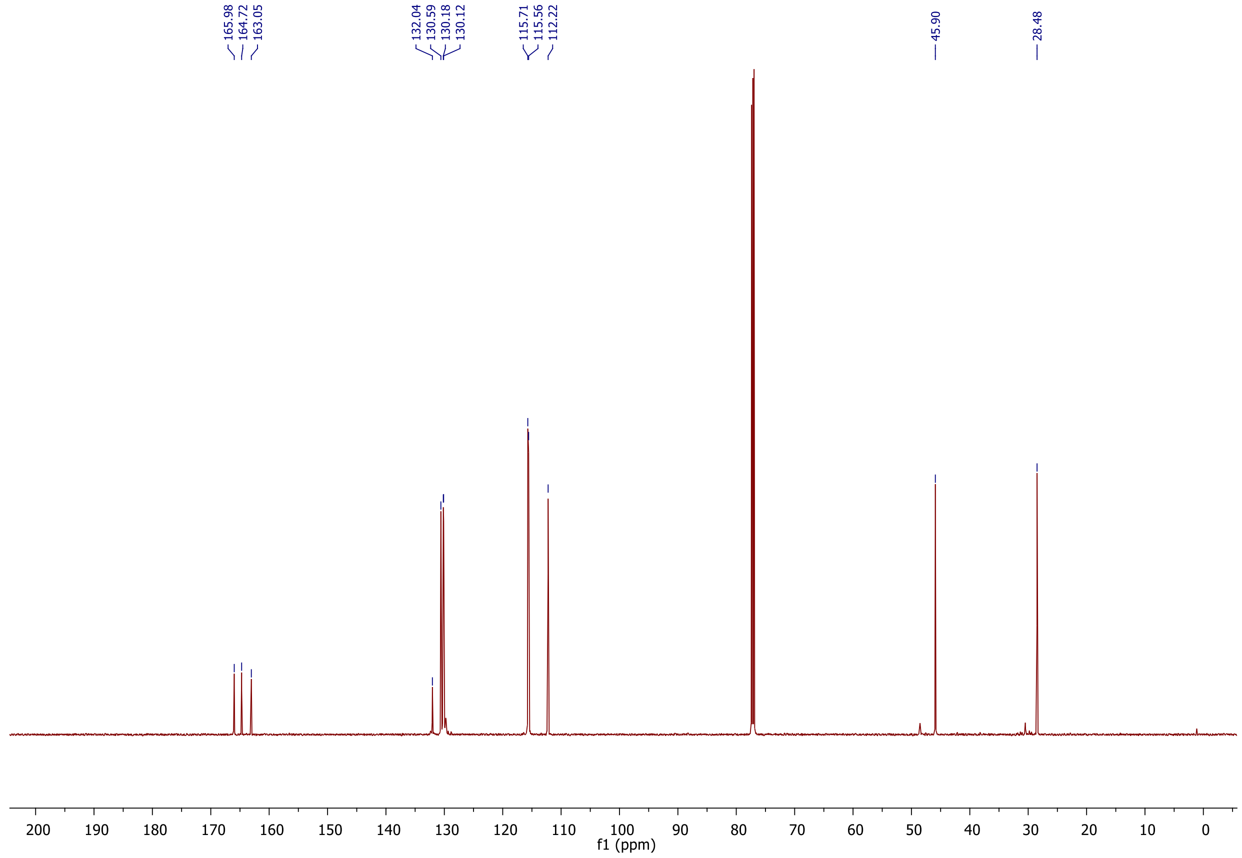

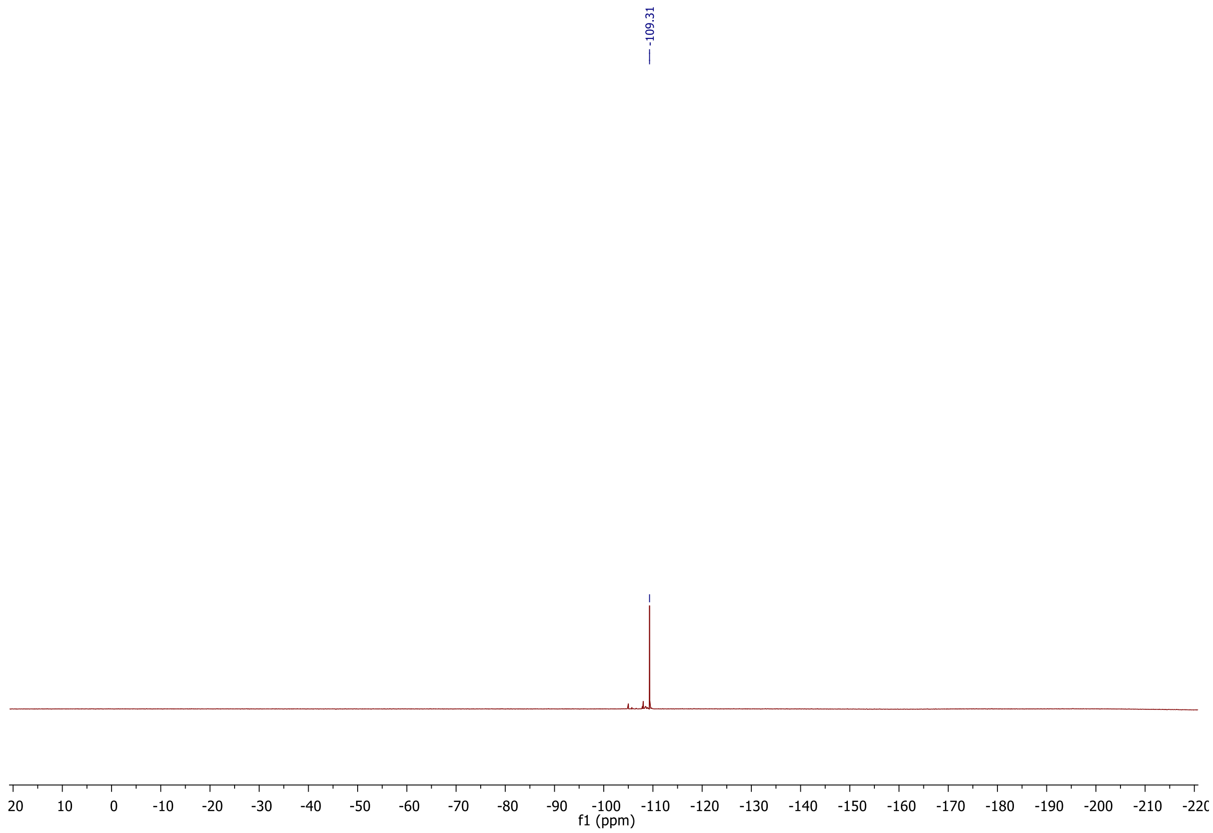
-109.31
20 10 0 -10 -20 -30 -40 -50 -60 -70 -80 -90 -100 -110 -120 -130 -140 -150 -160 -170 -180 -190 -200 -210 -220
f1 (ppm)

(3-Chlorophenyl)(2,3-dihydro-1H-pyrrol-1-yl)methanone (4o)

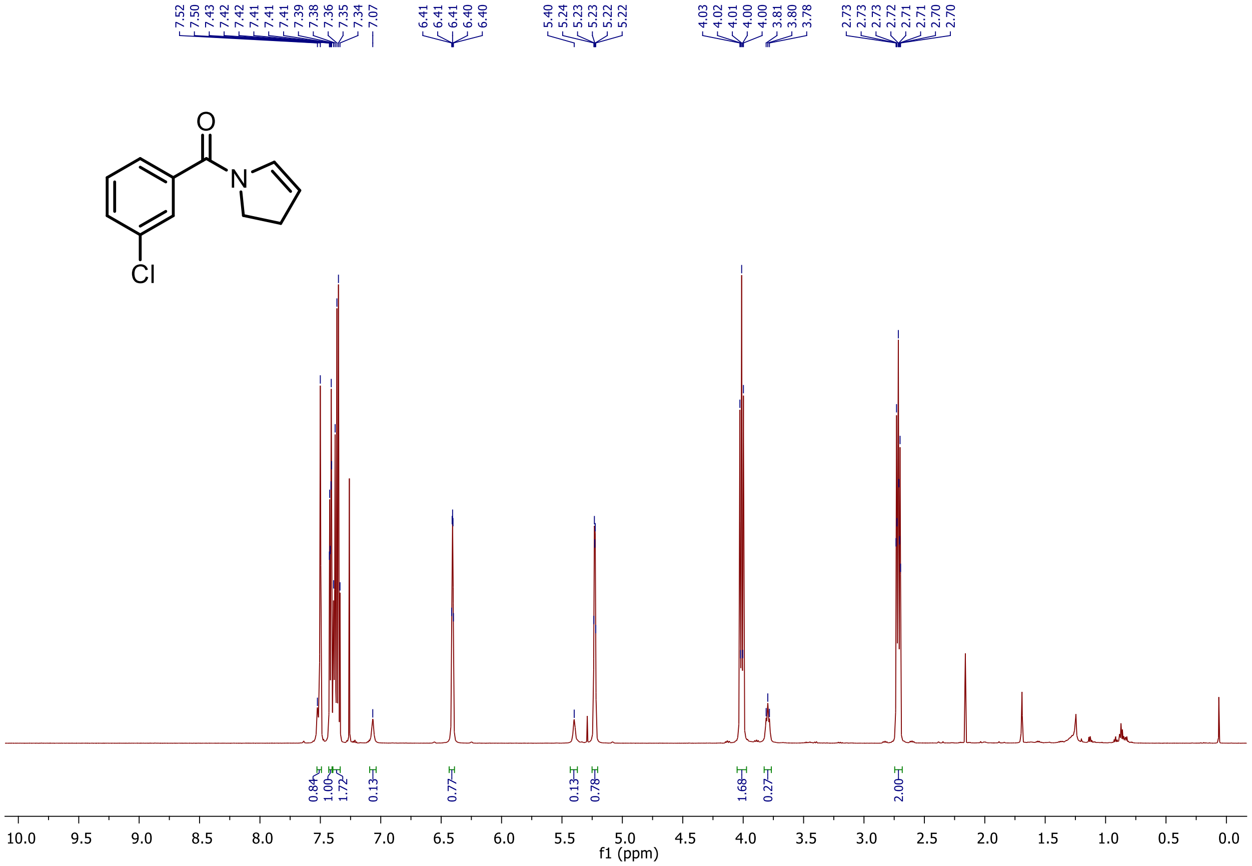

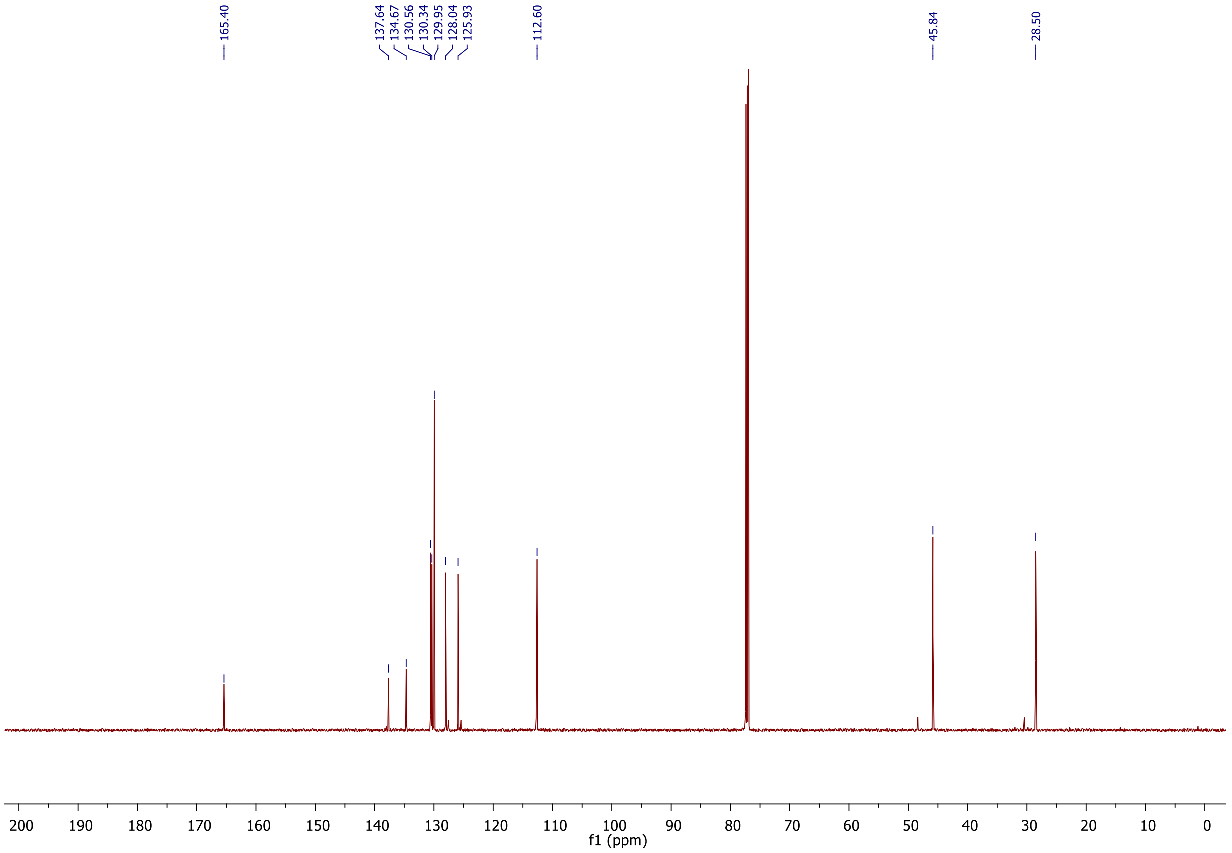

(2,3-Dihydro-1H-pyrrol-1-yl)(4-vinylphenyl)methanone (4p)

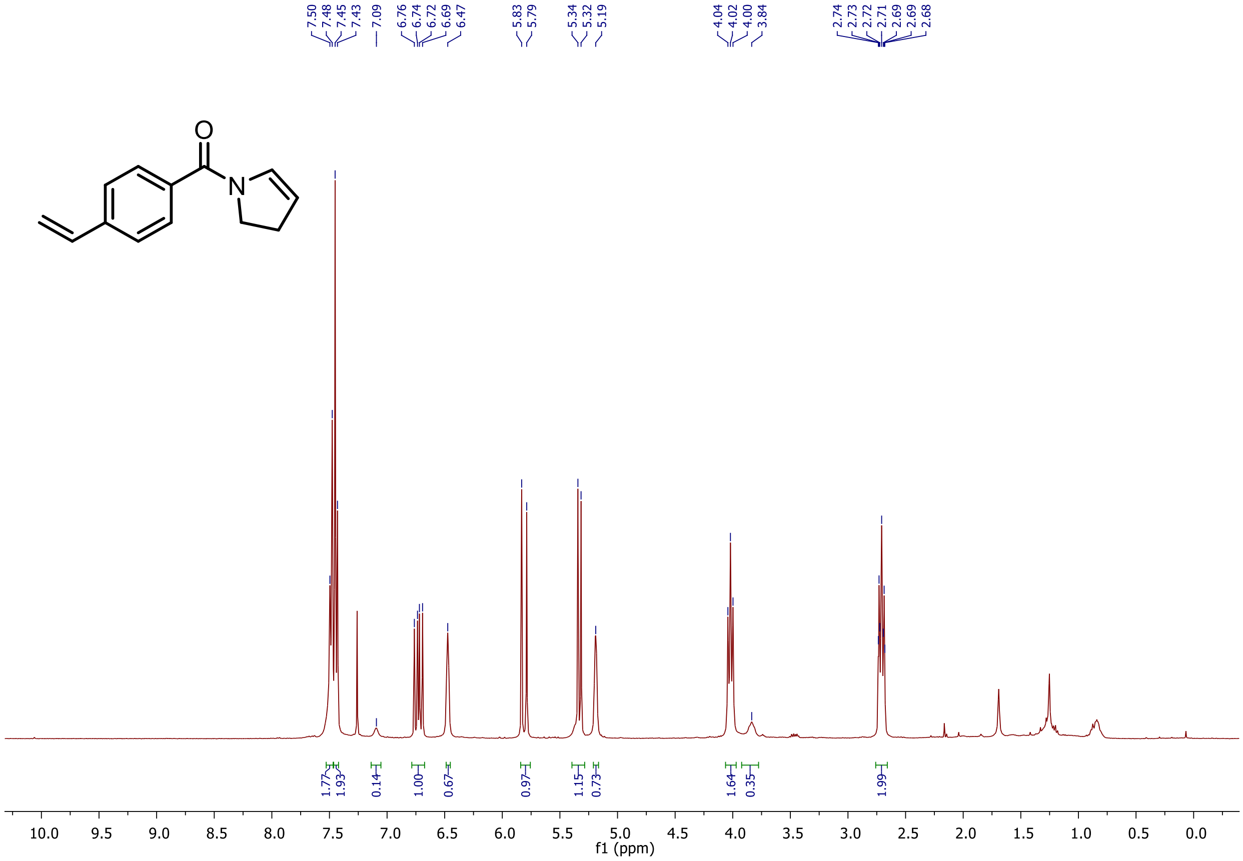

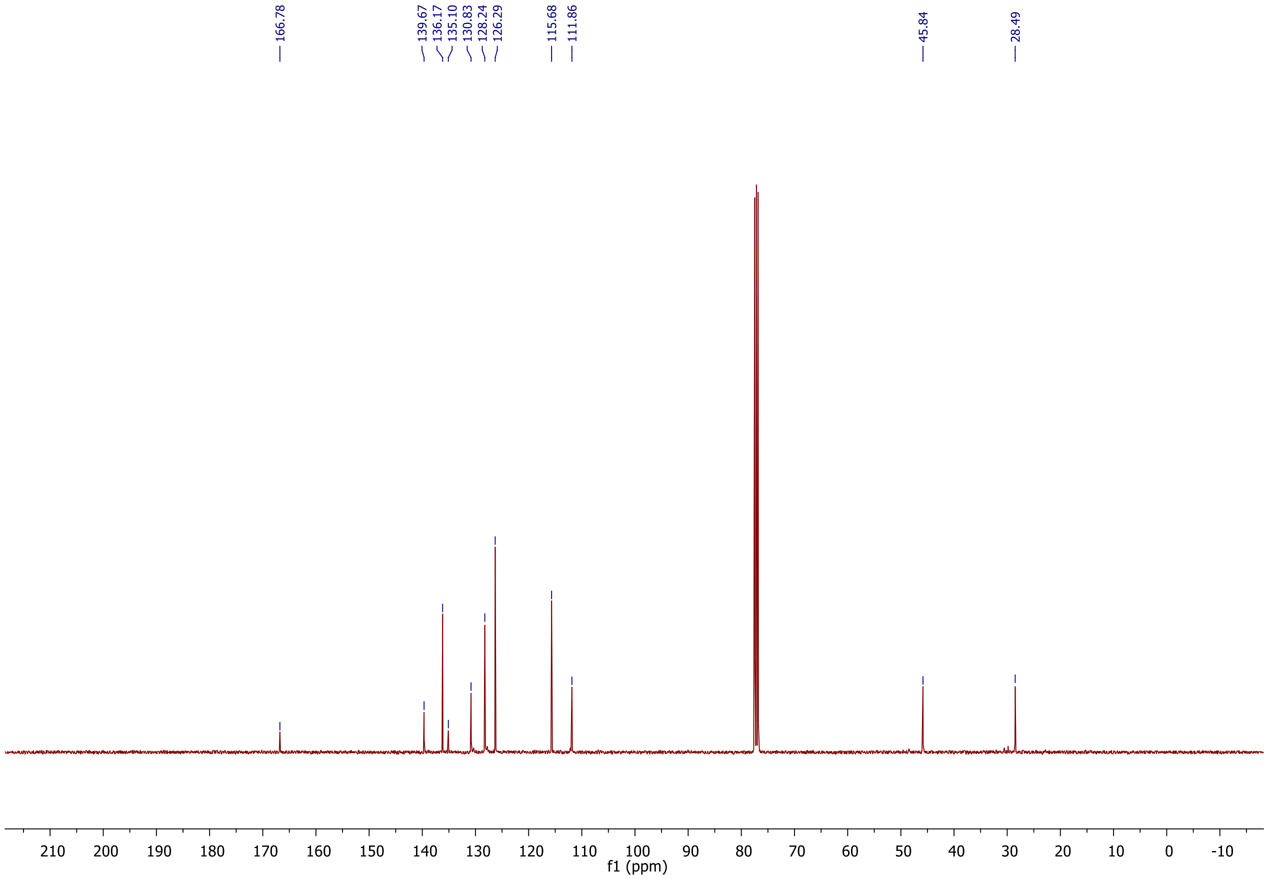

(2,3-Dihydro-1H-pyrrol-1-yl)(4-(methylthio)phenyl)methanone (4q)

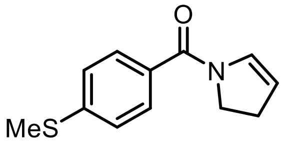

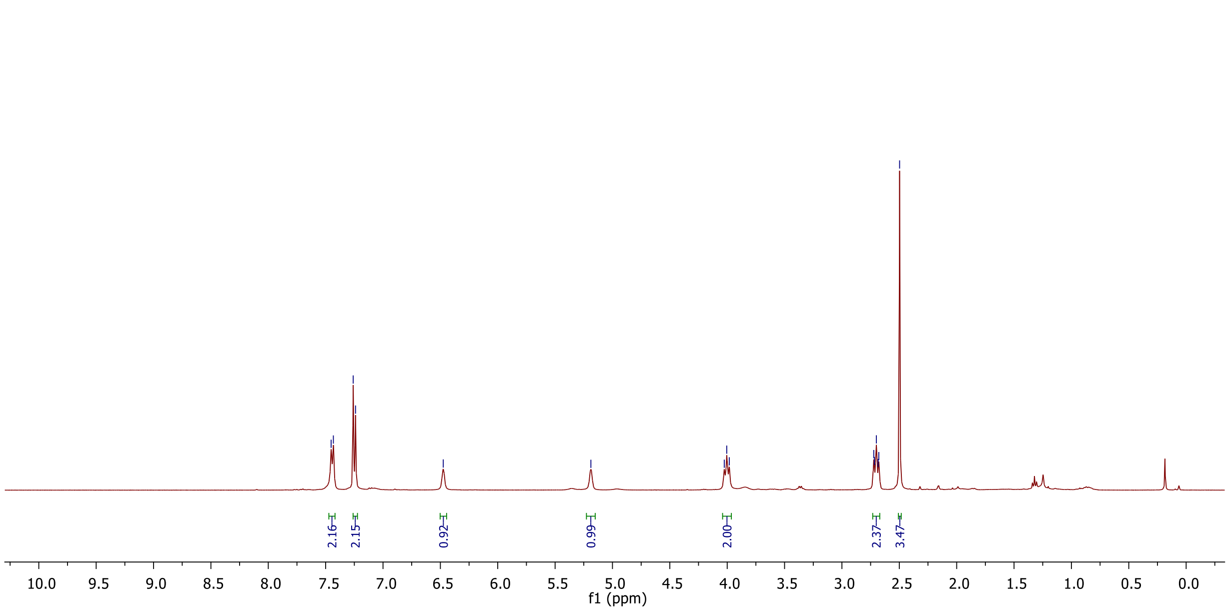

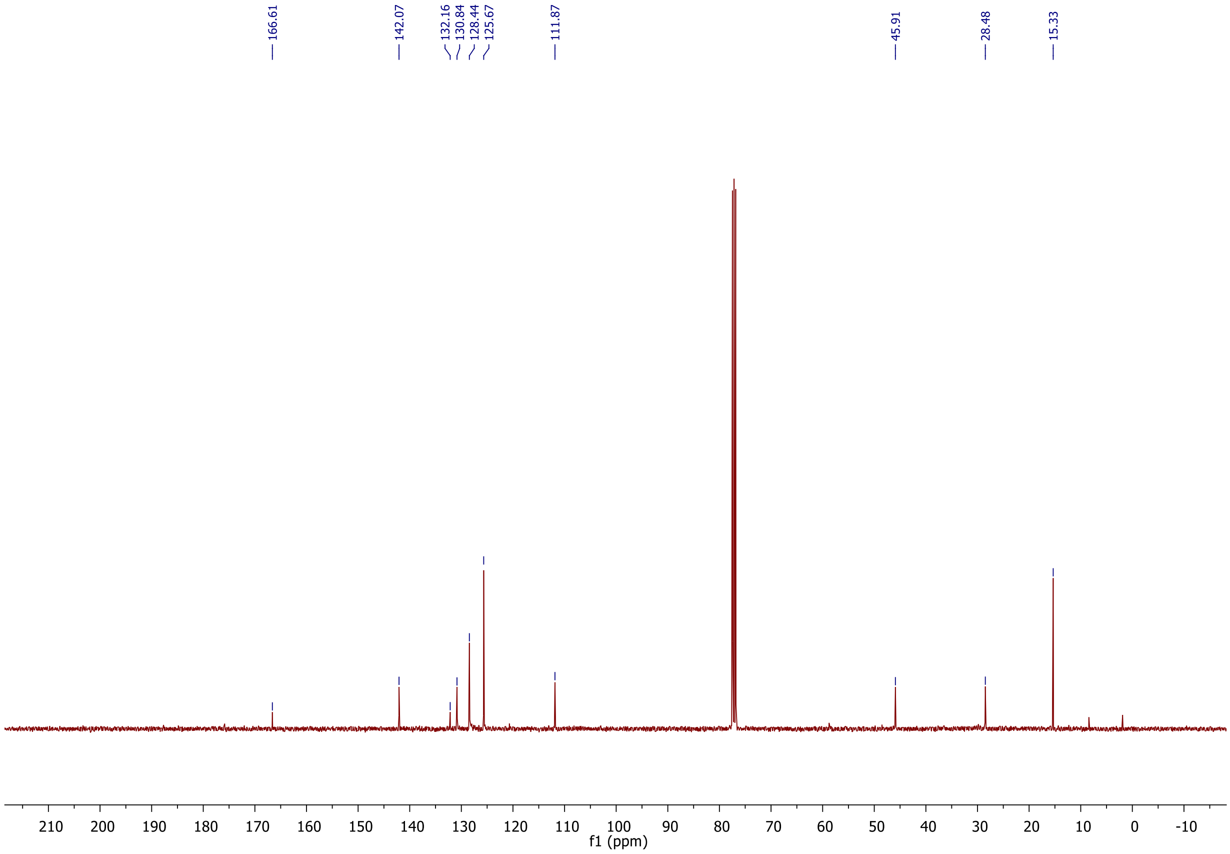

3-(2,3-Dihydro-1H-pyrrole-1-carbonyl)benzonitrile (4r)

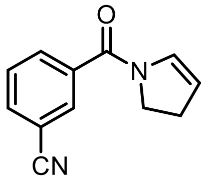

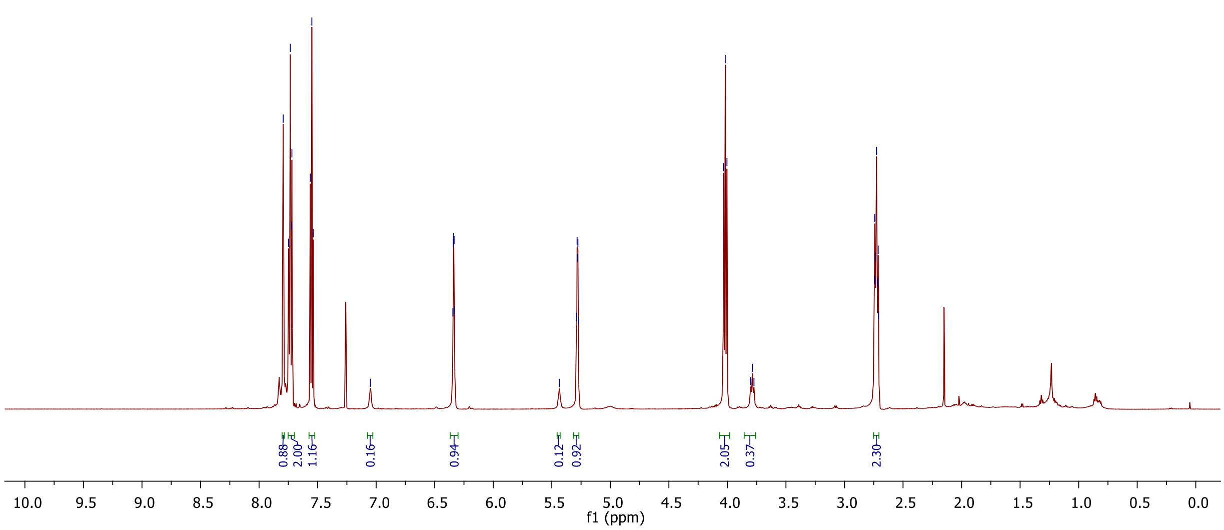

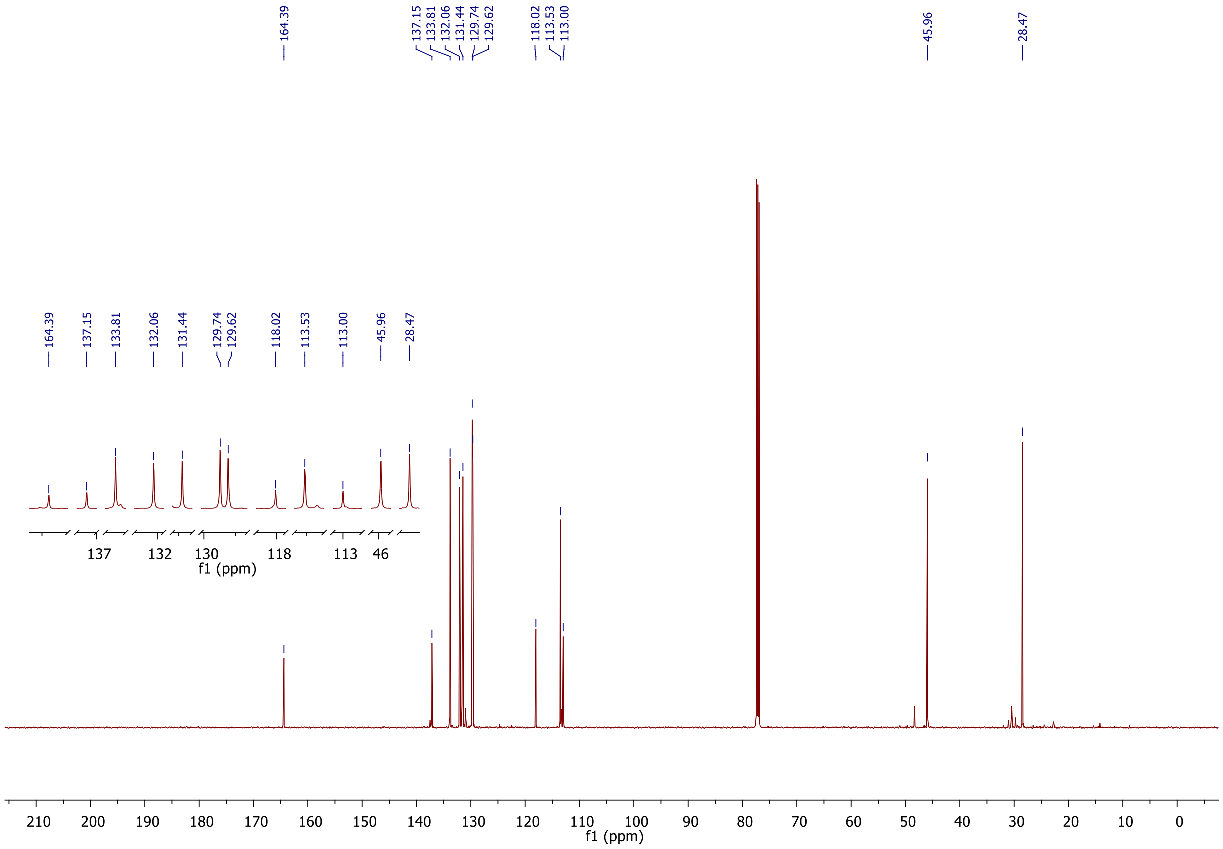

(2,3-Dihydro-1H-pyrrol-1-yl)(ferrocene)methanone (4u)

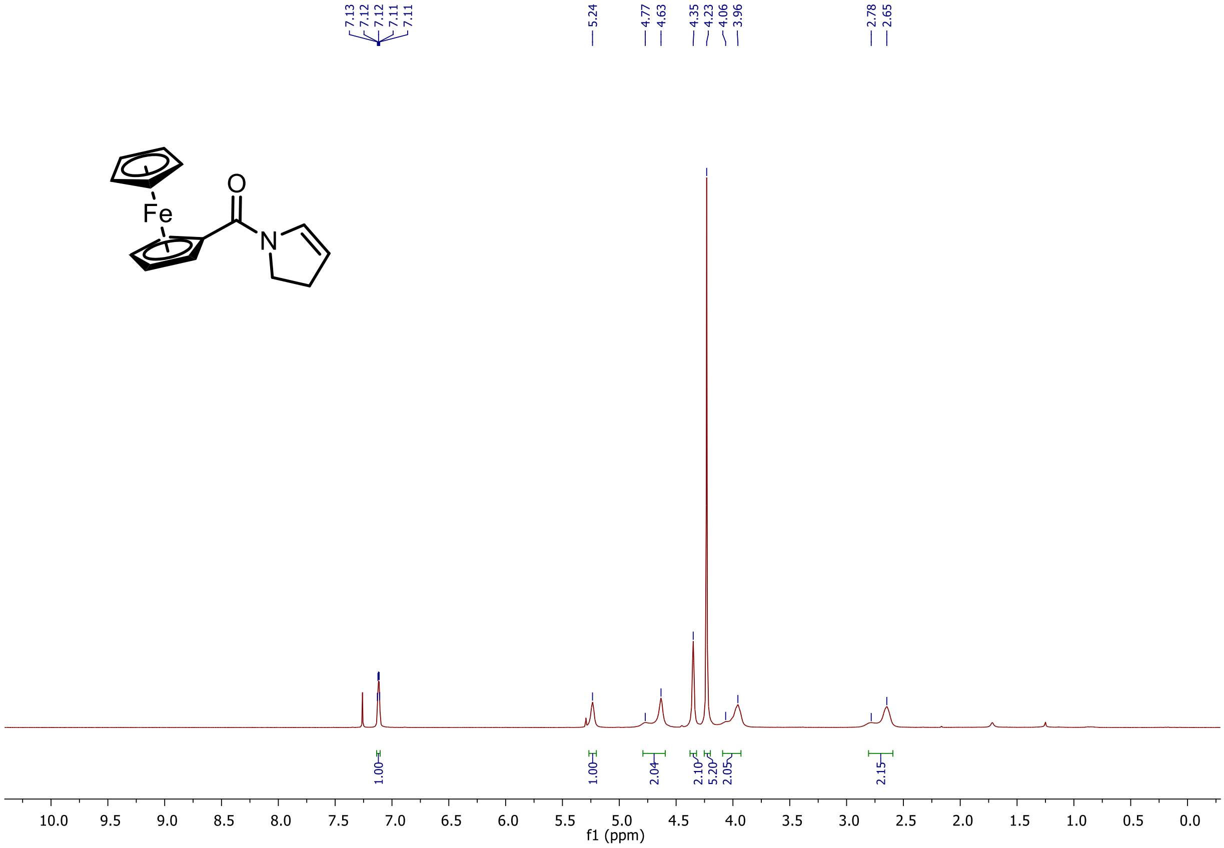

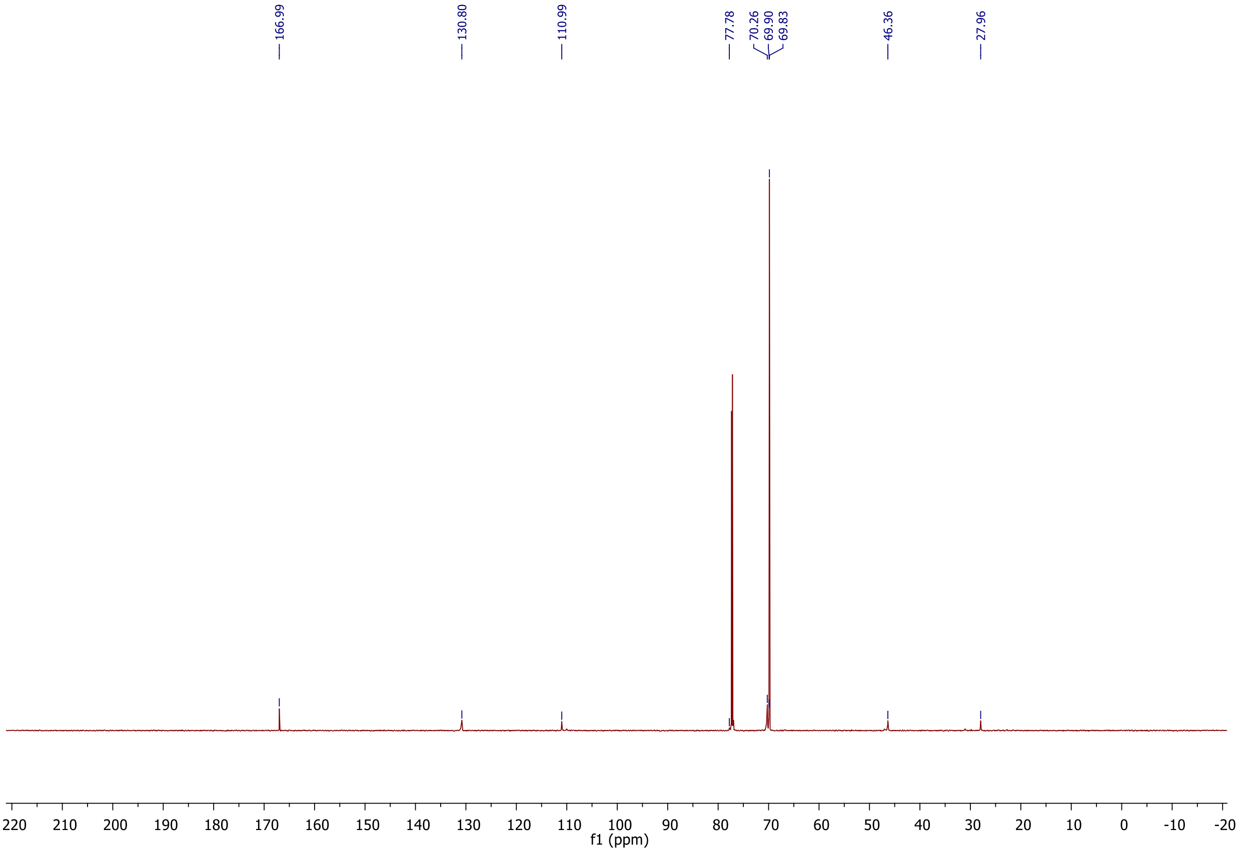

4-(2,3-Dihydro-1H-pyrrole-1-carbonyl)-2-methoxyphenyl 2-(3-cyano-4-isobutoxyphenyl)-4-methylthiazole-5-carboxylate (4v)

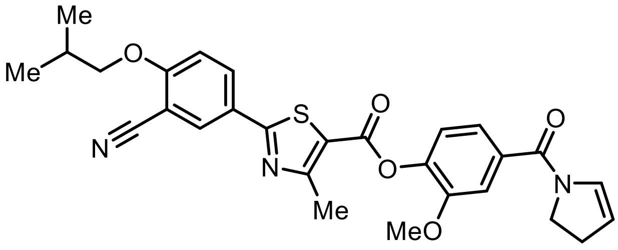

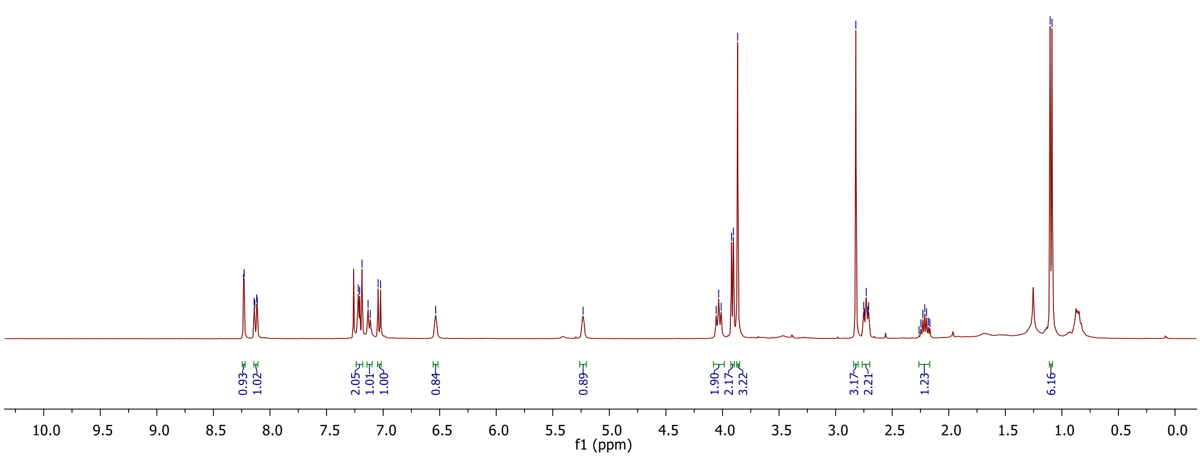

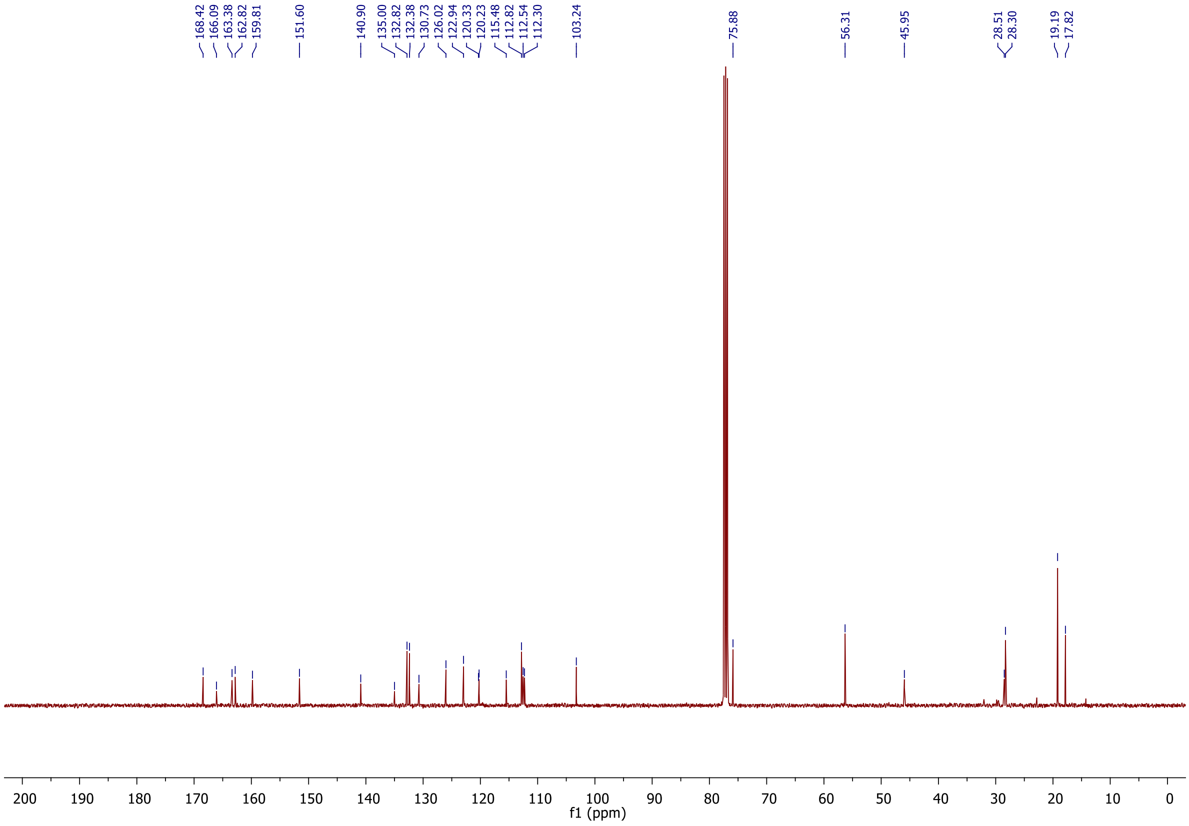

Methyl 1-benzoyl-8-oxo-2,3,3a,4,7,7a-hexahydro-1H-4,7-(epoxymethano)indole-5-carboxylate (5a)

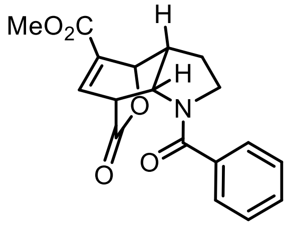

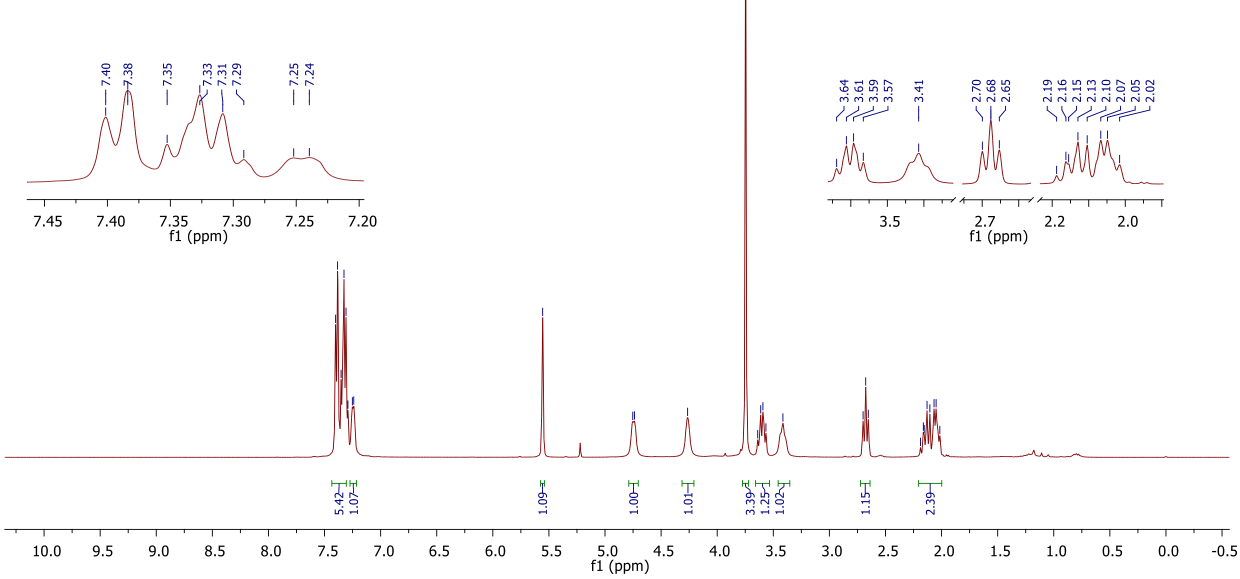

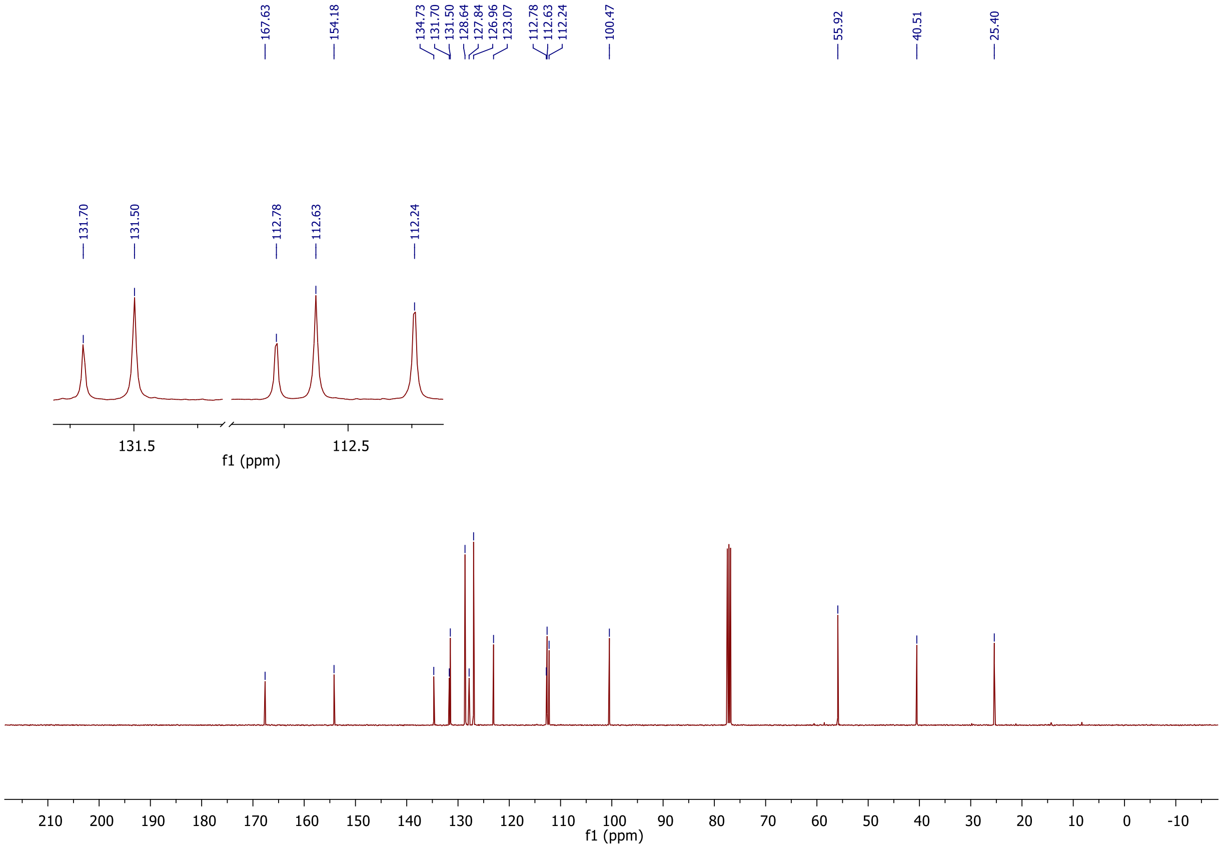

Ethyl 1-benzoyl-4-(4-chlorophenyl)-2,3-dihydro-1H-pyrrolo[3,2-c]quinoline-8-carboxylate (5b)

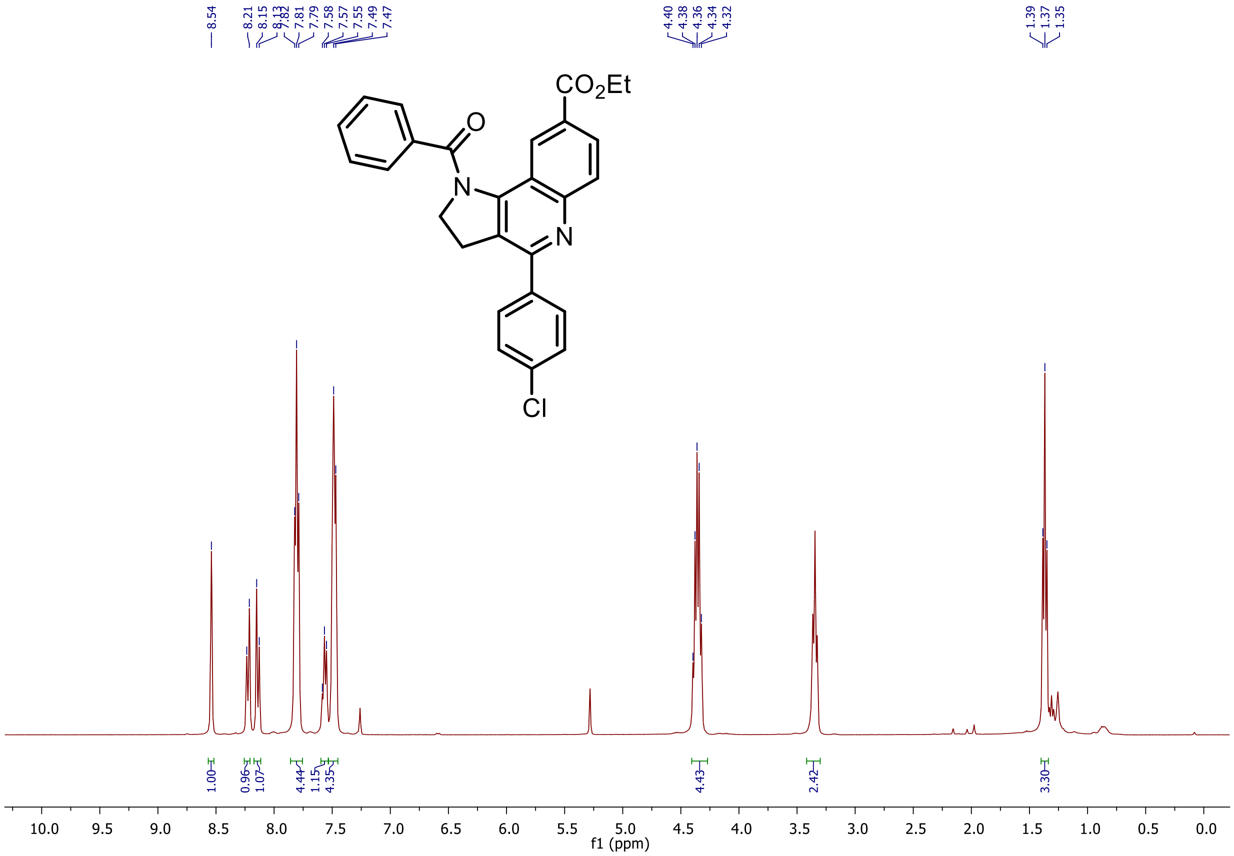

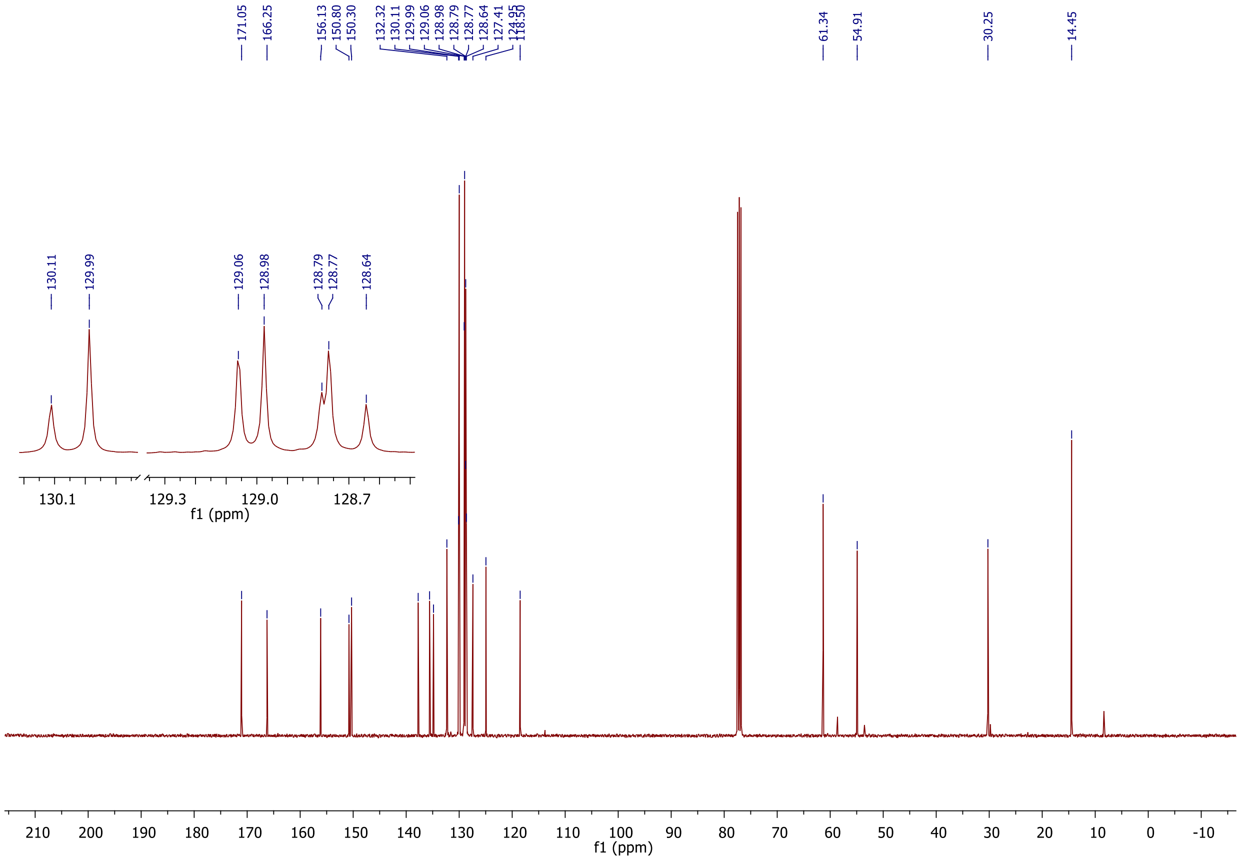

Mesityl(3,4,4a,8b-tetrahydrobenzo[3,4]cyclobuta[1,2-b]pyridin-1(2H)-yl)methanone (5c)

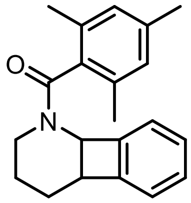

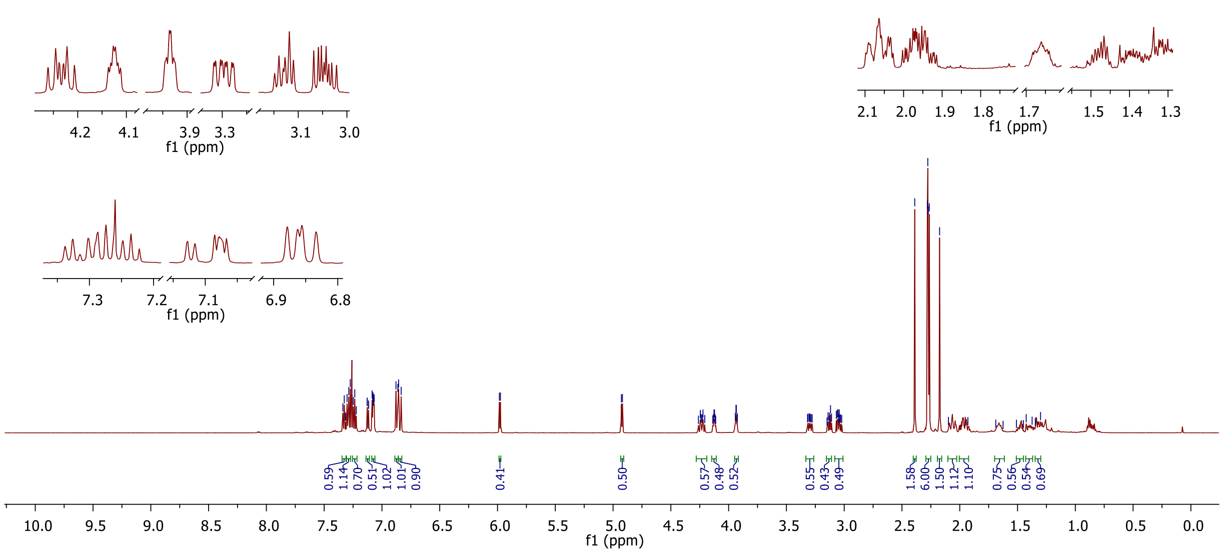

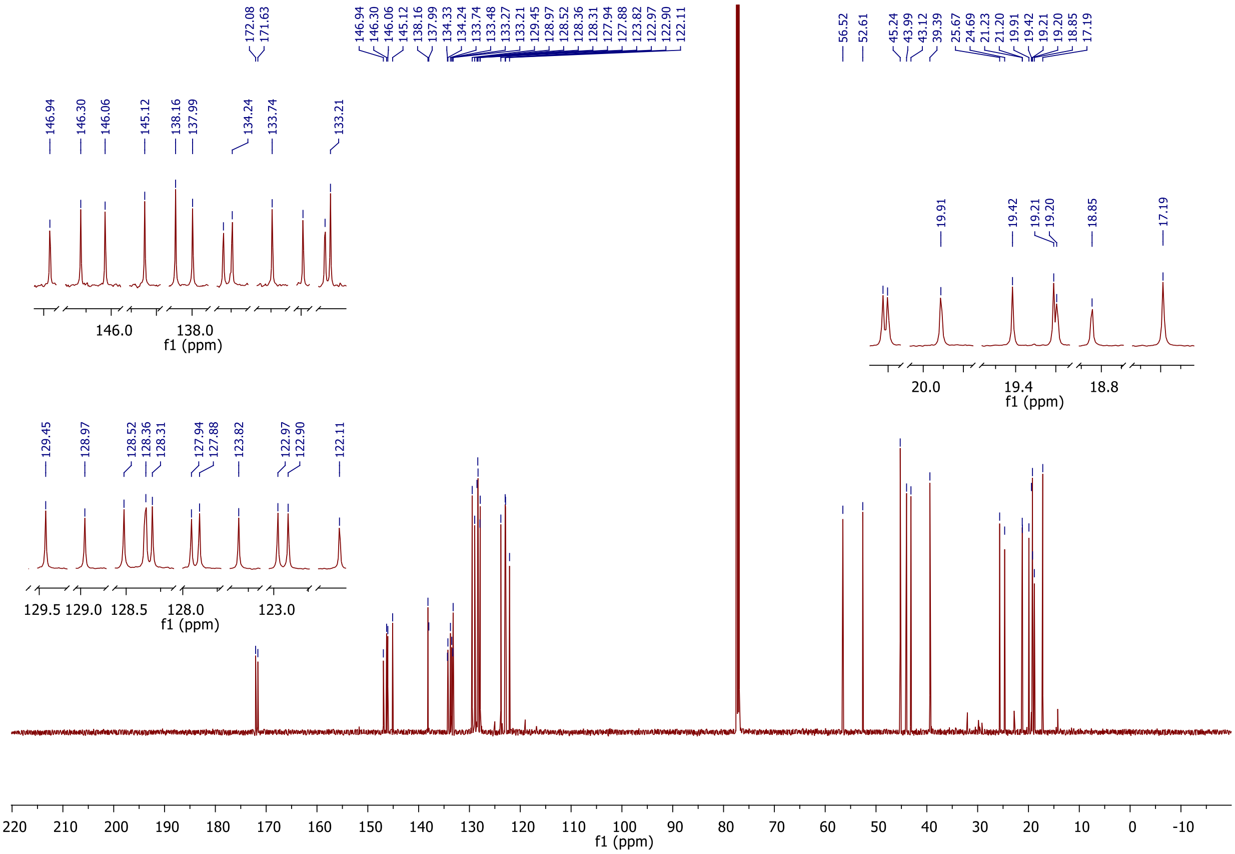

N-(2-(5-Methoxy-1H-indol-3-yl)ethyl)benzamide (5d)

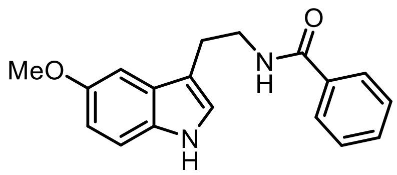

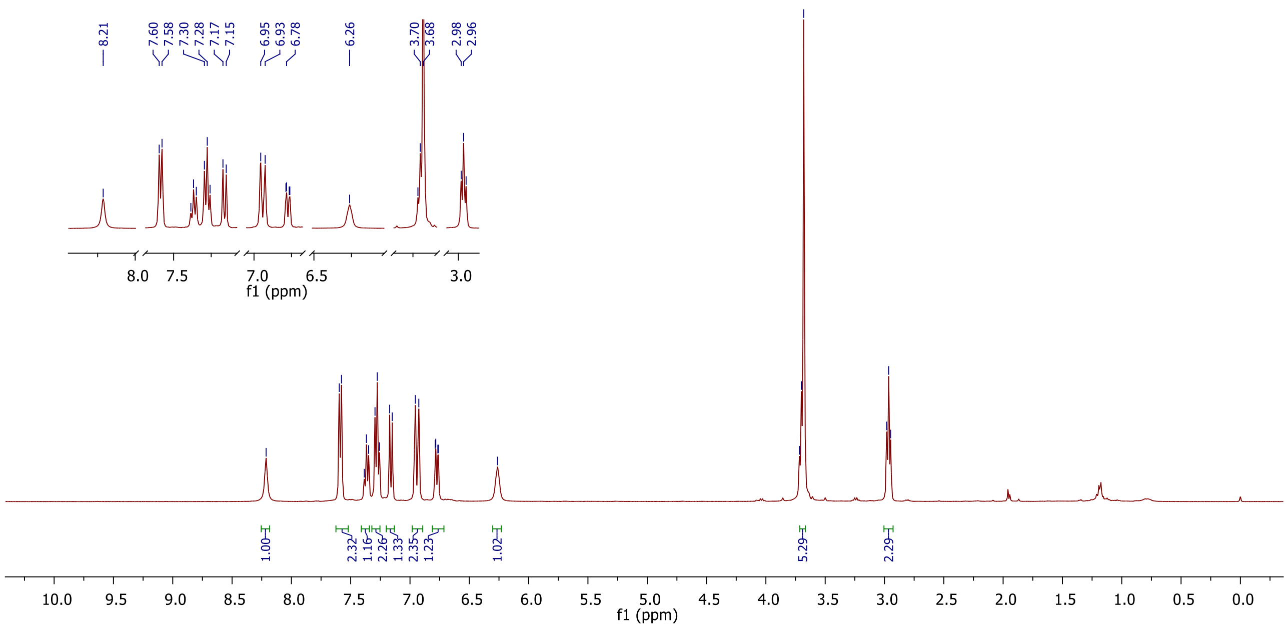

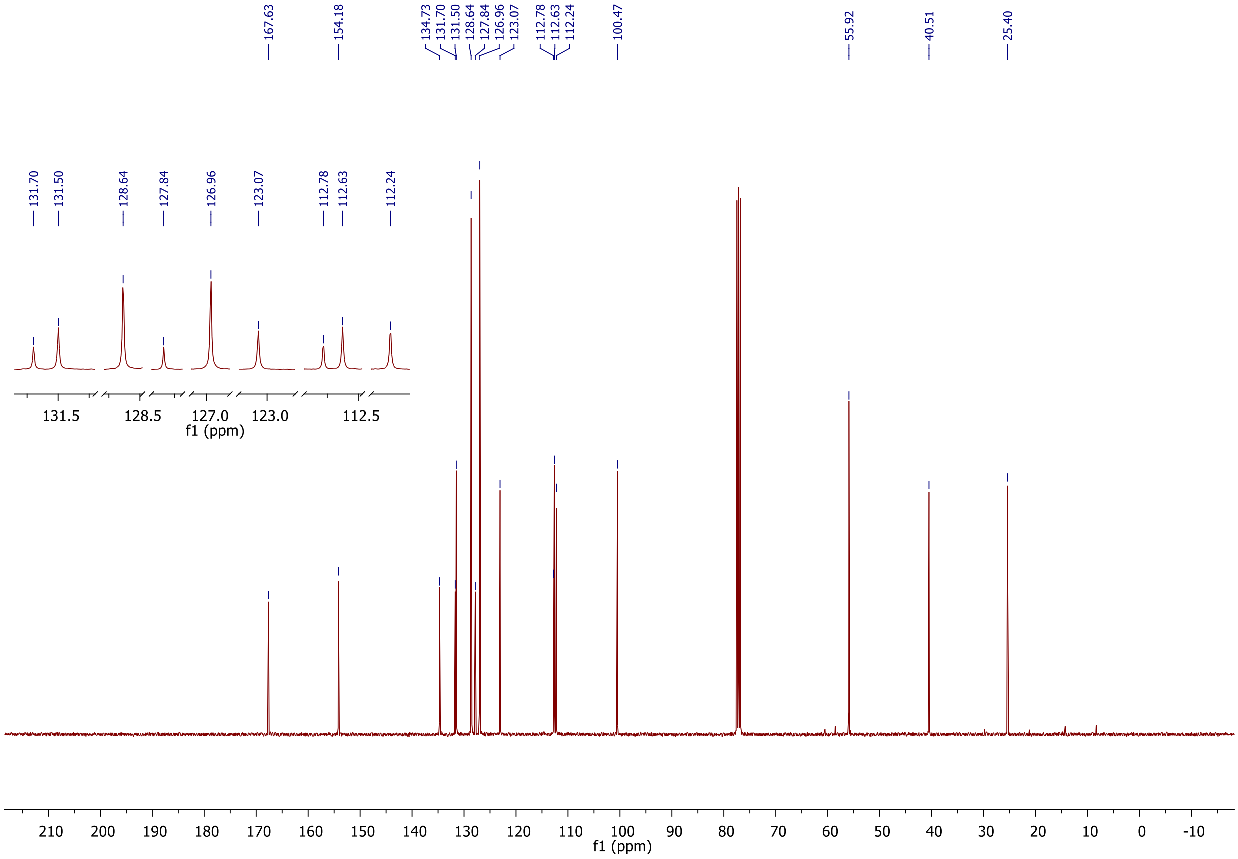

N-(3,3-Difluoropropyl)benzamide (5e)

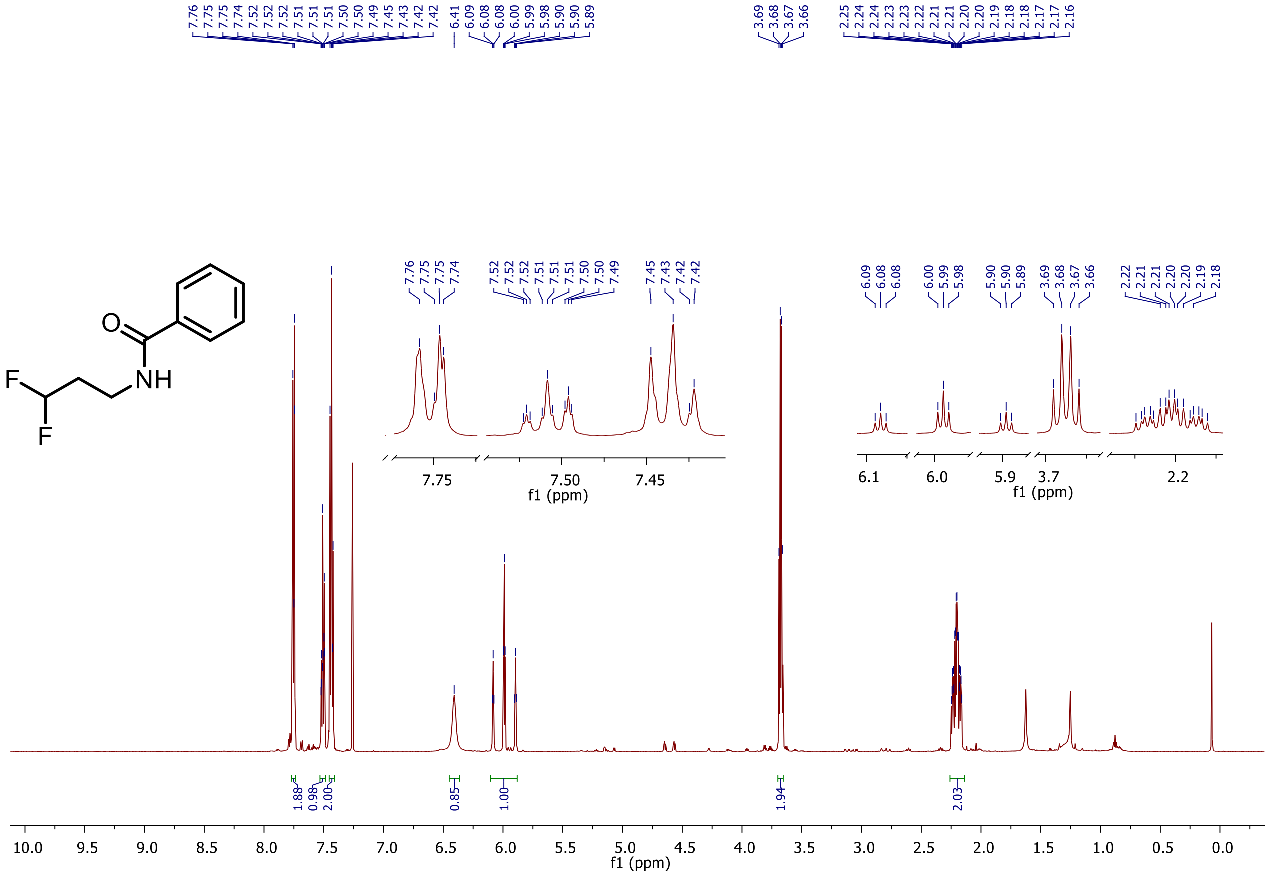

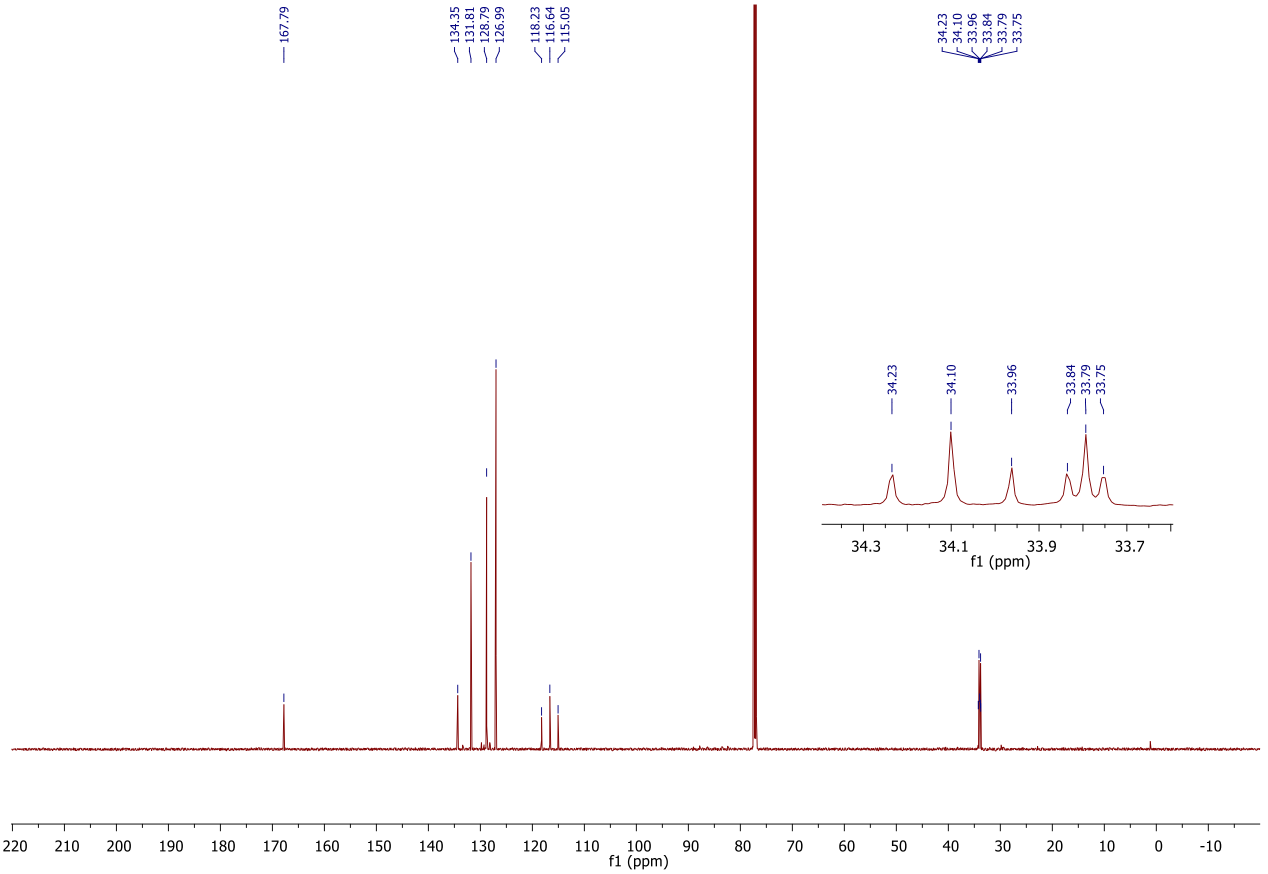

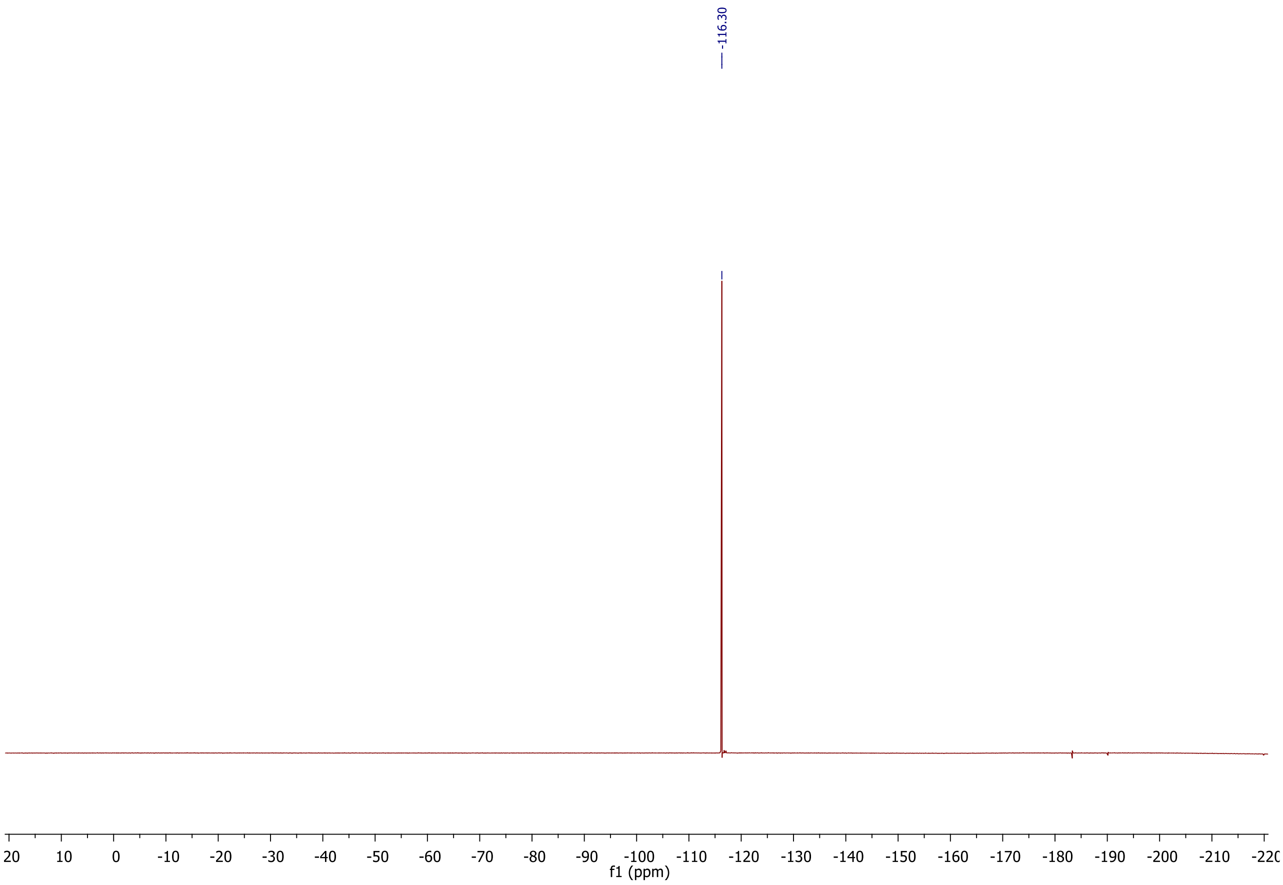
-116.30
20
10
0
-10
-20
-30
-40
-50
-60
-70
-80
-90
-100
-110
-120
-130
-140
-150
-160
-170
-180
-190
-200
-210
-220
f1 (ppm)

(R)-8-Hydroxy-7,9-dimethoxy-1,2,3,9b-tetrahydro-5H-pyrrolo[2,1-a]isoindol-5-one (5f)

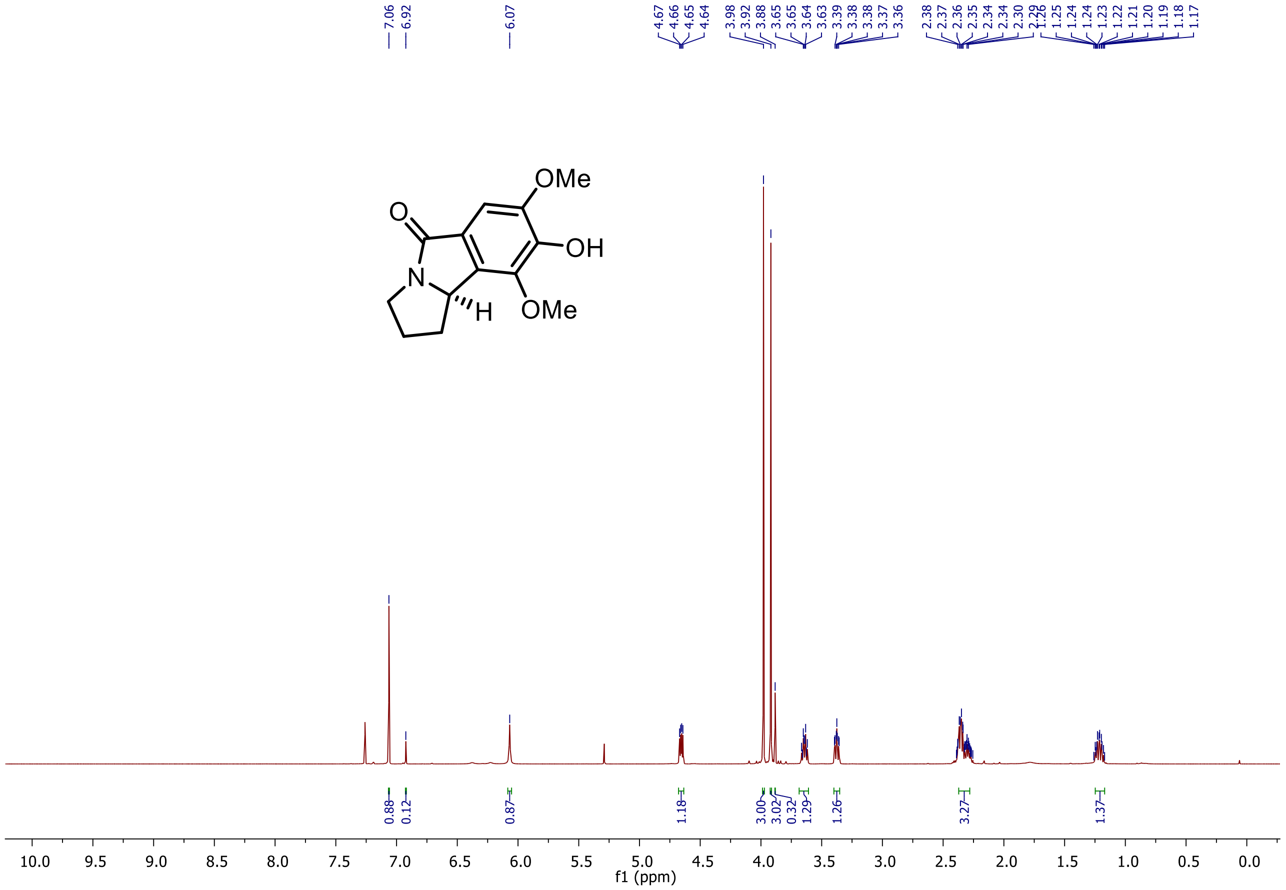

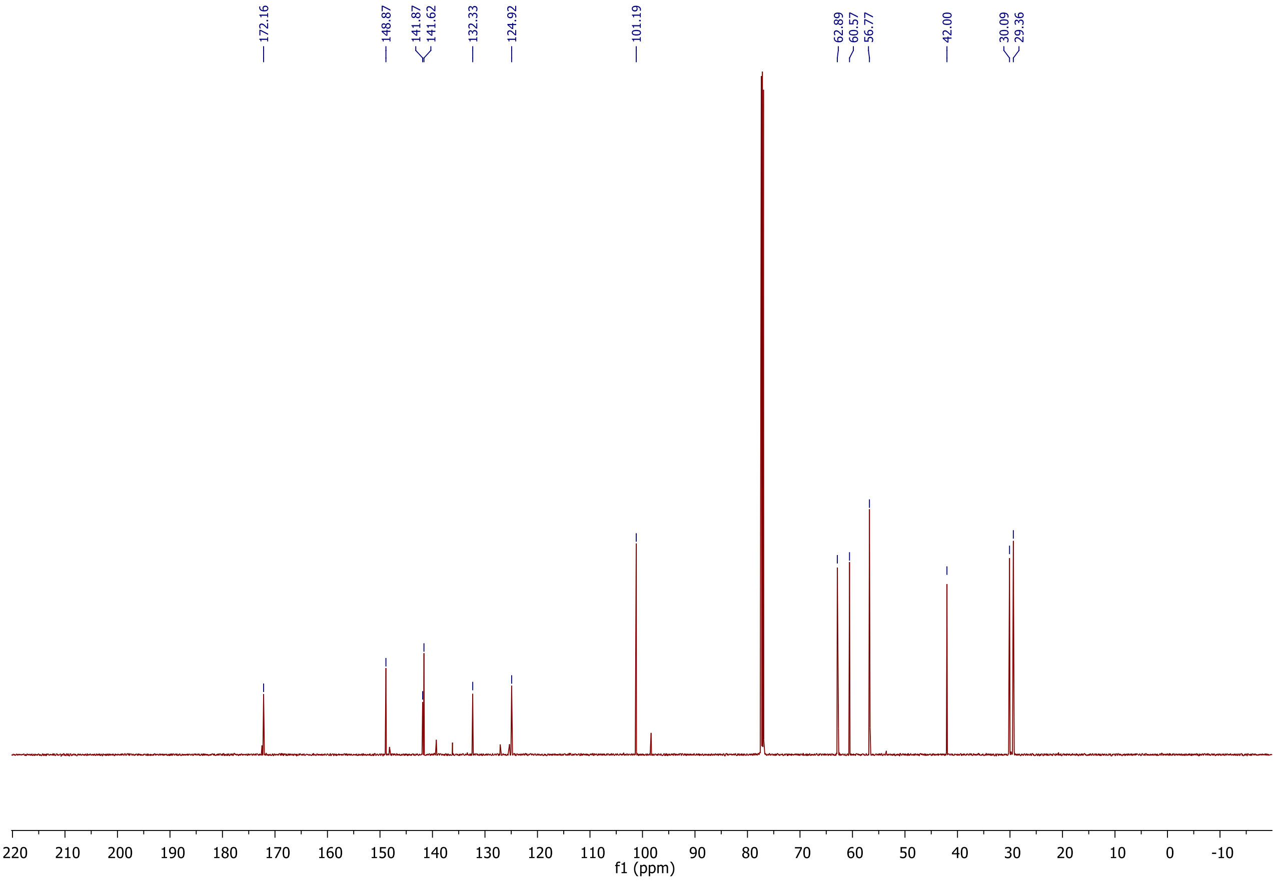

Mesityl(5-phenyl-3,4-dihydropyridin-1(2H)-yl)methanone (5g)

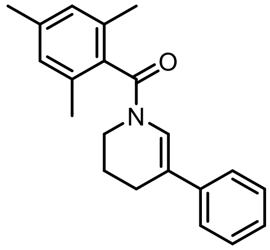

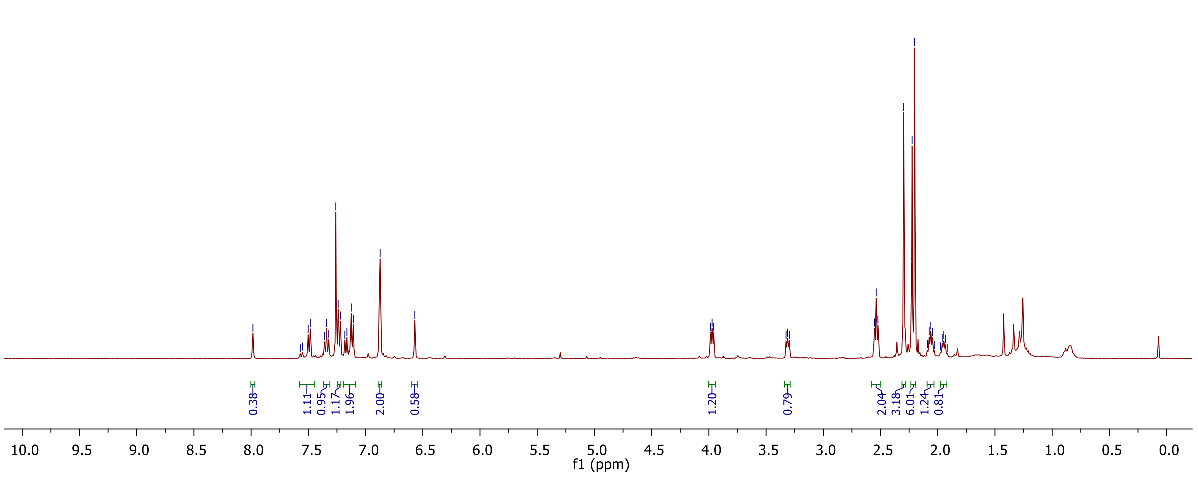

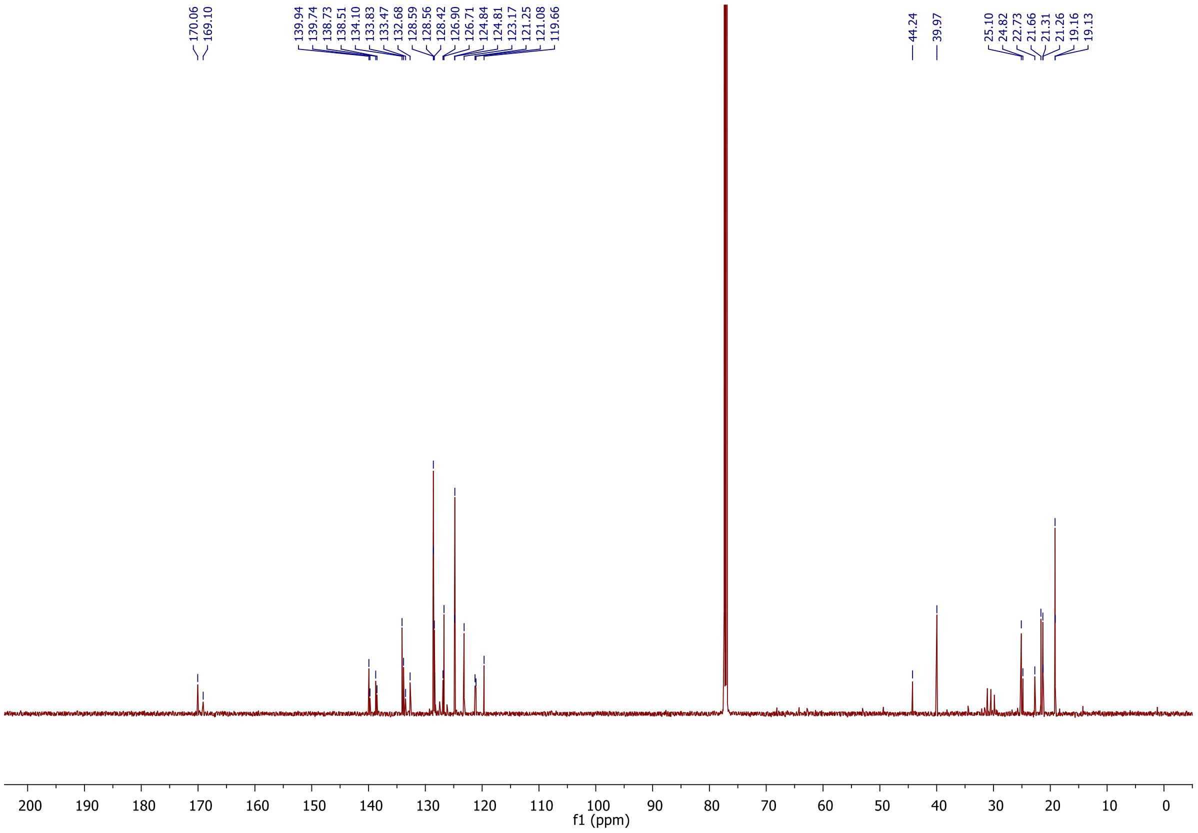

7. X-Ray Crystallographic Data

The X-ray intensity data was measured on Bruker D8 Venture diffractometer equipped with multilayer monochromator, Mo K/α INCOATEC micro focus sealed tube and Oxford cooling system. The structure was solved by *Direct Methods*. Non-hydrogen atoms were refined with *anisotropic displacement parameters*. Hydrogen atoms were inserted at calculated positions and refined with riding model. The following software was used: *Bruker SAINT software package*[i] using a narrow-frame algorithm for frame integration, *SADABS*[ii] for absorption correction, *OLEX2*[iii] for structure solution, refinement, molecular diagrams and graphical user-interface, *Shelxle*[iv] for refinement and graphical user-interface *SHELXS-2015*[v] for structure solution, *SHELXL-2015*[vi] for refinement, *Platon*[vii] for symmetry check. Experimental data and CCDC-Codes Experimental data (Available online: http://www.ccdc.cam.ac.uk/conts/retrieving.html) can be found in Table 1. Crystal data, data collection parameters, and structure refinement details are given in Tables 2 to 3. Asymmetric Unit visualized in Figure 1.

Table 1 Experimental parameter and CCDC-Code.

Sample	Machine	Source	Temp.	Detector Distance	Time/ Frame	#Frames	Frame width	CCDC
	Bruker		[K]	[mm]	[s]		[°]	
**5a**	D8	Mo	100	30	10	500	0.36	2075992

Methyl 1-benzoyl-8-oxo-2,3,3a,4,7,7a-hexahydro-1H-4,7-(epoxymethano)indole-5-carboxylate (5a)

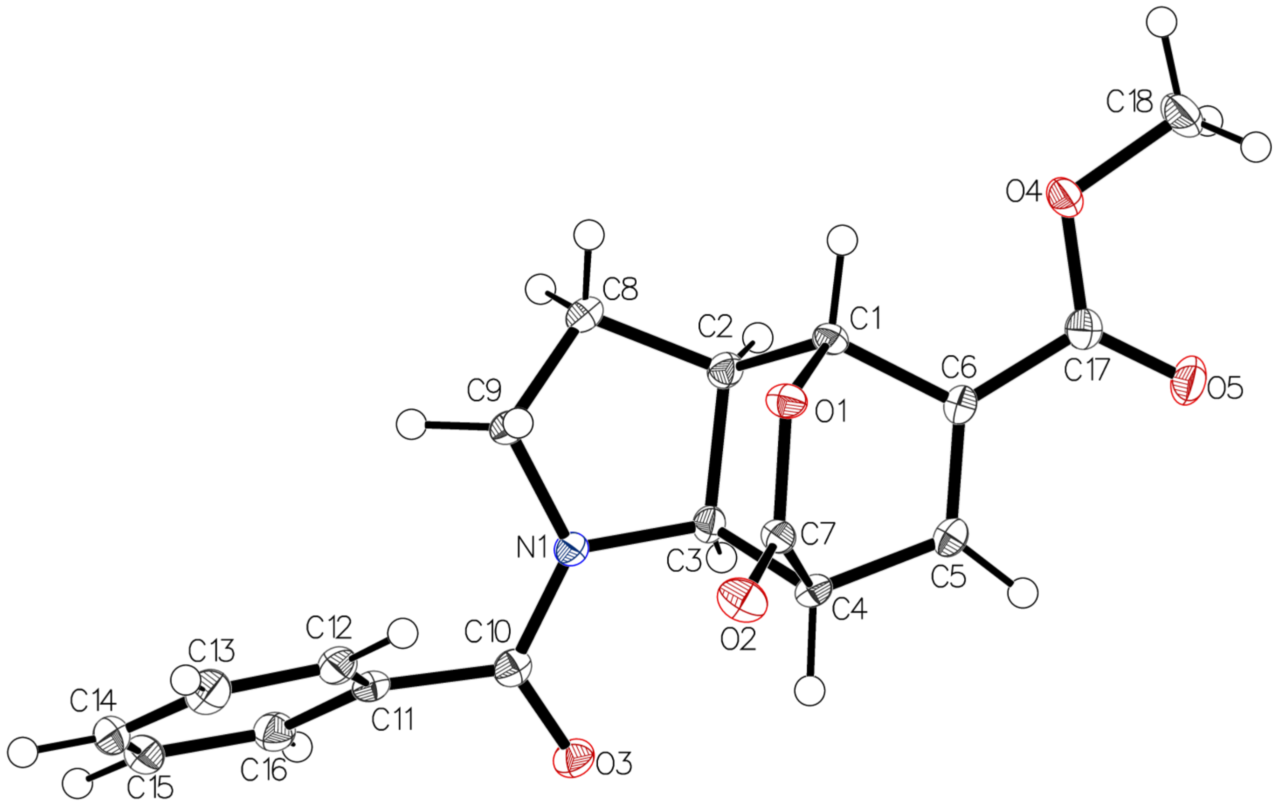

Figure 1 Crystal structure [5a] drawn with 50% displacement ellipsoid. The bond precision for C-C single bonds is 0.0318Å.

Table 2 Sample and crystal data. [5a]

Radiation [Å]	MoKα (λ = 0.71073)	Z	4	Measurement method	\f and \w scans
Crystal habit	clear colourless plate	a [Å]	10.5546(4)		
Crystal size [mm³]	0.204 × 0.085 × 0.011	b [Å]	6.4125(2)	Abs. correction type	multiscan
Empirical formula	C18H17NO5	c [Å]	22.8672(9)	Abs. correction Tmin	
Formula weight [g/mol]	327.32	α [°]	90	Abs. correction Tmax	
Temperature [K]	100.0	β [°]	91.479(2)	Density (calculated) [g/cm3]	1.405
Crystal system	Monoclinic	γ [°]	90	Absorption coefficient [mm⁻¹]	0.103
Space group	P21c	Volume [Å³]	1547.17(10)	F (000) [e⁻]	688.0

Table 3 Data collection and structure refinement. [5a]

2Θ range for data collection [°]	5.186 to 61.072	Index ranges		Goodness-of-fit on F^2	1.019
Reflections collected	11231	h	-15 ≤ h ≤ 14	Diff. peak and hole [e·Å⁻³]	0.34/-0.31
Data / restraints / parameters	4567/0/218	k	-5 ≤ k ≤ 9		
Refinement method	Direct Methods	l	-32 ≤ l ≤ 32	Function minimized	$\Sigma w (F_o^2 - F_c^2)^2$
		all data	R1 = 0.0848, wR2 = 0.1158	Weighting scheme	where
		I>2σ(I)	R1 = 0.0447, wR2 = 0.1037	$w=1/[\sigma^2(Fo^2) + (0.0720P)^2 + 204.8064P]$	$P=(F_o^2+2F_c^2)/3$

References

[i] Bruker SAINT v8.38B Copyright © 2005-2019 Bruker AXS
[ii] Sheldrick, G. M. (1996). *SADABS.* University of Göttingen, Germany.
[iii] Dolomanov, O.V., Bourhis, L.J., Gildea, R.J, Howard, J.A.K. & Puschmann, H. , OLEX2, (2009), J. Appl. Cryst. 42, 339-341
[iv] C. B. Huebschle, G. M. Sheldrick and B. Dittrich, ShelXle: a Qt graphical user interface for SHELXL, J. Appl. Cryst., 44, (2011) 1281-1284
[v] Sheldrick, G. M. (2015). *SHELXS v 2016/4* University of Göttingen, Germany.
[vi] Sheldrick, G. M. (2015). *SHELXL v 2016/4* University of Göttingen, Germany.
[vii] A. L. Spek, Acta Cryst. 2009, D65, 148-155.
